# Phosphorylation in liquid sulfur dioxide under prebiotically plausible conditions

**DOI:** 10.1038/s42004-022-00761-w

**Published:** 2022-11-03

**Authors:** Constanze Sydow, Christiane Seiband, Alexander F. Siegle, Oliver Trapp

**Affiliations:** 1 Department of Chemistry and Pharmacy, Ludwig-Maximilians-University, Butenandtstr. 5-13, 81377 Munich, Germany; 2 Max-Planck-Institute for Astronomy, Königstuhl 17, 69117 Heidelberg, Germany

**Keywords:** Origin of life, Networks and systems biology, Organic chemistry

## Abstract

In nature, organophosphates provide key functions such as information storage and transport, structural tasks, and energy transfer. Since condensations are unfavourable in water and nucleophilic attack at phosphate is kinetically inhibited, various abiogenesis hypotheses for the formation of organophosphate are discussed. Recently, the application of phosphites as phosphorylation agent showed promising results. However, elevated temperatures and additional reaction steps are required to obtain organophosphates. Here we show that in liquid sulfur dioxide, which acts as solvent and oxidant, efficient organophosphate formation is enabled. Phosphorous acid yields up to 32.6% 5′ nucleoside monophosphate, 3.6% 5′ nucleoside diphosphate, and the formation of nucleoside triphosphates and dinucleotides in a single reaction step at room temperature. In addition to the phosphorylation of organic compounds, we observed diserine formation. Thus, we suggest volcanic environments as reaction sites for biopolymer formation on Early Earth. Because of the simple recyclability of sulfur dioxide, the reaction is also interesting for synthesis chemistry.

## Abstract (web summary)

Organophosphates are essential to facilitate information storage and energy transfer in living organisms, but their prebiotic origins remain unknown. Here, the authors report a liquid sulfur dioxide-mediated phosphorylation of nucleosides from phosphorous acid in a single reaction step at room temperature.

## Introduction

Organophosphates are essential for living organisms because they enable information storage and transfer^1,2^, signal transduction^3,4^, energy transfer, compartmentalization, and participate in metabolism^5–7^. Hence, prebiotically plausible phosphorylation reactions of organic compounds are of eminent importance and interest. However, phosphorylation under Early Earth conditions faces several obstacles: condensation reactions are thermodynamically unfavourable in aqueous solution and the nucleophilic attack at the phosphorus (P) atom is kinetically inhibited because of the phosphate’s negative charge^8,9^. The low solubility of phosphate minerals such as apatite (Ca_5_(PO_4_)_3_(Cl, F, OH)), which are the dominant P source on Early Earth, further complicates organophosphate formation^2,10,11^. Nevertheless, various P(V) based pathways towards organophosphates have been investigated in recent years to circumvent these high barriers. Among these are heating of hydroxy group-containing substrates together with phosphates^2,12–14^, non-aqueous and eutectic reaction media^15–17^, mineral catalysis^18,19^, application of condensed phosphates e. g. pyrophosphate or trimetaphosphate^20–22^, use of condensation agents such as cyanamide^23^, urea^24^, carbonyl sulfide or cyanate and activated phosphates such as diamidophosphate (DAP) and amidotriphosphate (AmTP)^25–31^.

Another strategy to overcome the kinetic barrier of P(V) based phosphorylation and the low solubility of phosphate minerals is the application of water-soluble P(III) compounds as starting material^15,32,33^. In a weakly reducing anoxic environment mainly consisting of N_2_ and CO_2_ with CO, H_2_, and reduced sulfur gases as trace compounds P(III) oxidation should be extremely slow^34,35^. Consequently, coexistence of P(III) species and phosphates on the Early Earth is plausible. Various prebiotically conceivable P(III) sources have been explored in the past which can be divided into two categories: terrestrial and extraterrestrial^2^. Terrestrial sources are geothermal pools and the reduction of orthophosphate by ferrous iron^35,36^ or electrical discharges in the context of volcanic eruptions^37,38^. Extraterrestrial sources include alkyl phosphonic acids found in Murchison meteorite and Schreibersite (Fe, Ni)_3_P mainly stemming from iron meteorites or chondrites^11,39,40^. Subsequent Schreibersite corrosion in the presence of water is proposed to provide H-phosphonates among other P species^41–44^, e.g., phosphite found in early Archean marine carbonates probably originates from this process^45^.

Tetra-coordinated P(III) species, e.g., H-phosphonates, are characterized by an electrophilic P centre like their P(V) analogues^46^. However, in contrast to their analogous P(V) centres nucleophilic attacks at P(III) centres are faster because of the phosphonate’s reduced negative charge^9,46^. This property is highly beneficial to form biopolymers, e.g., the oligomerisation of RNA and DNA nucleotides^47^. Another advantage is that the oxidation of reduced P species is exothermic (~55 kJ/mol) and can deliver enough energy for the phosphorylation of an organic molecule^48^.

Consequently, previous studies with P(III) show promising phosphorylation yields. However, elevated reaction temperatures are required and the conversion stops at the nucleoside H-phosphonate (N-p^III^) stage without an additional oxidizing agent^15,32^. Since H-phosphonate diesters are more sensitive towards hydrolysis than their charged P(V) analogues, only Schwartz et al. reported the formation of nucleoside dimers bridged by a H-phosphonate unit (N-p^III^-N)^8,9,32,49^. Lönnberg introduced elemental sulfur as additional oxidant to overcome these obstacles. Unfortunately, an additional desulfurization step is necessary to convert the resulting phosphorothioate linked oligomers into the desired phosphate analogues^33^.

A P(III) based pathway to (oligomeric) organophosphates without an additional oxidation step has not yet been envisaged. Thus, we explored the possibility of a single step synthesis by the application of liquid sulfur dioxide (SO_2_) as a redox active reaction medium. At atmospheric pressure, the boiling point of SO_2_ is −10 °C and at room temperature SO_2_ is liquid above ~3 bar^50–52^. In classical synthesis chemistry liquid SO_2_ is well known as solvent and has been employed in ring opening and cyclization reactions^52,53^, polymerizations^54^, coordination chemistry^55^, the Ritter reaction, and in the alkylation and alkoxyalkylation of allylsilanes^56,57^. On the early Earth SO_2_ was provided by volcanic outgassing^58,59^. Kasting et al. estimate that the SO_2_ emission rate was about three times higher than at present because of the intensified volcanic activity on the early Earth^59^. Reconstruction of the exact conditions on early Earth is a complex task that is further complicated by the rare Hadean rock record^34,60^. Consequently, the atmospheric pressure which highly affects the surface temperature cannot be determined exactly. Discussed values for the prevailing atmospheric pressure range from ~0.01 to 100 bar^61,62^. Zahnle estimates that the nitrogen partial pressure on the early Earth was 2 to 3 bar^63^. Furthermore, it is assumed that the surface temperature was less than 273.15 K at 4.3 Ga^63,64^. Models calculate a probability of 67% for this scenario^64^. In addition to the global average, the possibility of local and temporary pressure and temperature differences has to be kept in mind. Thus, the possibility of local environments on the early Earth that tolerate the temporary existence of liquid SO_2_ is conceivable.

We have recently demonstrated amino acid condensation under prebiotically conceivable reaction conditions in liquid SO_2_ starting from low reactant loadings^65^. In addition to its hygroscopic nature that is advantageous for condensation reactions SO_2_ is capable of oxidizing P(III)^66^. Here, we show that P(III) based phosphorylation in liquid SO_2_ leads very efficiently to oligomeric organophosphates in a single reaction step. Organophosphates are observed at room temperature and even at low reactant concentrations. All canonical nucleotides are obtained in good yields and further prebiotically relevant organophosphates are accessible.

## Results

### Exploration of the reaction conditions

**Fig. 1:**
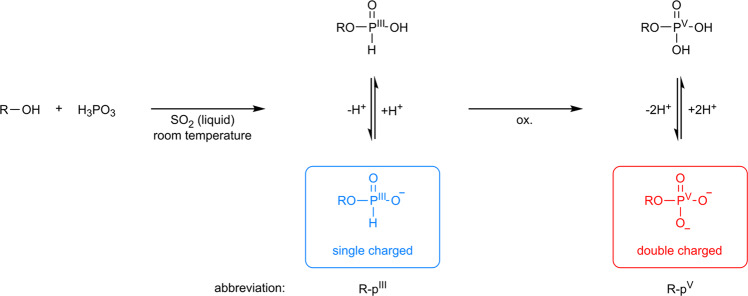
Conceptualization of the phosphorylation with H_3_PO_3_ in liquid SO_2_. Condensation of a substrate with H_3_PO_3_ in liquid SO_2_ at room temperature yields the corresponding H-phosphonate. Under the same reaction conditions the reaction medium oxidizes the H-phosphonate intermediate to the phosphate analogue.

**Fig. 2:**
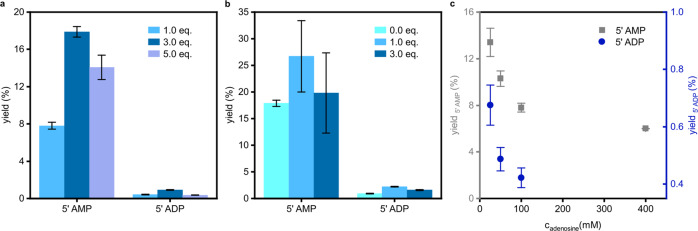
Evaluation of the reaction conditions. **a** Yields depending on H_3_PO_3_ concentration starting from A (100 mM, 1.0 eq.) after 7 d. **b** Yields depending on urea concentration starting from A (100 mM) and H_3_PO_3_ (300 mM) after 7 d. **c** Yields depending on A concentration starting from A and H_3_PO_3_ (1.0 eq.) after 7 d. Depicted are mean values, error bars refer to ± s.d. and were obtained by double determination via capillary electrophoresis (CE) (for yield calculation see supplementary information).

In continuation of our investigations on peptide formation reactions in SO_2_ we aimed to expand the scope of prebiotically plausible reactions in SO_2_ to phosphorylation reactions. In a pressure apparatus SO_2_ was condensed on a mixture of adenosine (A) (100 mM) and phosphorous acid (H_3_PO_3_) (1.0 eq.) and the reaction mixture was stirred for 7 d at room temperature (Fig. 1 and Supplementary Figs. 1, 2). Analysis of the reaction mixture by capillary electrophoresis coupled to electrospray ionization Orbitrap mass spectrometry (CE-ESI-MS) or UV detection (Supplementary Fig. 3) showed the formation of the phosphorylated products including adenosine H-phosphonate (A-p^III^) (constitution of potential isomers has not been determined; labels and abbreviations illustrate all phosphate/phosphonate binding modes and refer to the entirety of all formed isomers) (Supplementary Figs. 16, 17, Supplementary Tables 1, 2, and Supplementary Data 1)^67^. Apart from the P(III) compound, adenosine monophosphates (AMPs) (5′,3′ and 2′ AMP) and traces of cyclic adenosine monophosphate (cAMP) were detected. As expected for a non-enzymatic approach, the regioselectivity is not controlled. In addition to the monomeric compounds, adenosine diphosphate (ADP) (5′ ADP and other isomers), dinucleotide species and A dimers bridged by a phosphate group (A-p^V^-A) were obtained. The extracted ion electropherogram (EIE) of the dinucleotide species (p^V^-A-p^V^-A/A-p^V^-p^V^-A) shows a discrete peak at 9.90 min and several signals at around 12.40 min. The A units can either be bridged by pyrophosphate (A-p^V^-p^V^-A) or by phosphate linkages (p^V^-A-p^V^-A). Since phosphate nucleophiles are superior to alcohol nucleophiles, the formation of A-p^V^-p^V^-A dimers is conceivable^8^. Tandem mass spectrometry (MS/MS) spectra corroborate that A-p^V^-p^V^-A dimers are the faster migrating species (Supplementary Data 2). The signals at higher migration times were assigned to p^V^-A-p^V^-A dimers. Co-injection of a 5′-adenylic acid-3′,5′-adenosine phosphate (5′-p^V^-A-3′-p^V^-5′-A) reference confirmed this result (Supplementary Fig. 4 and Supplementary Data 1). It has to be noticed, that in contrast to the monomeric P(III) phosphorylated reaction products, no P(III) bridged nucleosides (A-p^III^-A) and P(III) phosphorylated dinucleotides (p^III^-A-p^III^-A/ A-p^III^-p^III^-A) were detected. This is in agreement with experimental data, showing that oxidation of phosphonate diesters is rapid compared to phosphonate monoesters^32,68^. Furthermore, Peyser et al. reported rapid hydrolysis of phosphonate diesters^49^. With these very promising results using liquid SO_2_ not only as solvent but also as oxidant in P(III) phosphorylation reactions we comprehensively screened the reaction conditions. At first, we varied the H_3_PO_3_ concentration. An increase in the yield of 5′ AMP and 5′ ADP was observed if an excess of H_3_PO_3_ (3.0 eq.) was applied (Fig. 2a, Supplementary Figs. 18–21, and Supplementary Tables 3, 4). Furthermore, a broadening of the reaction product spectrum was observed. In addition to the acyclic products obtained with stoichiometric amounts of H_3_PO_3_, traces of adenosine diphosphonate (A-p^III^-p^III^/p^III^-A-p^III^), mixed diphosphorylated species (A-p^V^-p^III^/p^III^-A-p^V^), adenosine triphosphate (ATP) and mixed (non-) cyclic adenosine diphosphate (p^V^-A-cp^V^/A-cp^V^-p^V^) were unambiguously detected (Supplementary Data 1). Further increase of the H_3_PO_3_ concentration (5.0 eq.) led to the formation of mixed H-phosphonate phosphate dinucleotides (A-p^III^-p^V^-A/p^III^-A-p^V^-A) in trace amounts but did not alter the product range to a greater extent (Supplementary Data 1). Rapid oxidation of mixed P(III) P(V) dinucleotides to p^V^-A-p^V^-A/A-p^V^-p^V^-A by liquid SO_2_ is presumed to protect them from hydrolysis. An interesting aspect in this context is, that oxidation rates of phosphonate diesters exceed those of phosphonate monoesters, which suggests that the oxidation could be the driving force in polymerization reactions according to studies by Lönnberg^32,33,68,69^. However, while the increase of H_3_PO_3_ (5.0 eq.) led to the formation of trace amounts of mixed dinucleotides, the 5′ AMP and 5′ ADP yields slightly decreased at the same time (Supplementary Figs. 22–25 and Supplementary Tables 5, 6). It has to be considered that product formation and hydrolysis are competing processes. Acceleration of the latter by acids is possibly the reason that yields do not further increase even when more phosphorylation agent is added^70^.

Next, we investigated whether the phosphorylation could be enhanced by the addition of urea (Fig. 2b). Urea is prebiotically plausible and well known to promote phosphorylation and other condensation reactions^17,20,65,71^. Discussed enhancement mechanisms for reactions at elevated temperature involve urea hydrolysis, formation of the activating agent cyanate and of carbamoyl phosphates, and phosphor amidates as activated intermediates^17,20,24,48,72,73^. Starting from the optimized H_3_PO_3_ concentration, we found that the phosphorylation is indeed promoted by urea. Both the addition of 1.0 eq and 3.0 eq. affected the product mixture only slightly but led to a clear increase in 5′ AMP and 5′ ADP yields (Supplementary Figs. 26–33, Supplementary Tables 7–10, and Supplementary Data 1). Yields of up to 26.7% for 5′ AMP and 2.2% for 5′ ADP were detected with a stoichiometric amount of urea. On the contrary, the analogous reaction under anoxic conditions in water provided only traces of AMP. In addition, A-p^III^ was observed (Supplementary Data 1).

Since we did not detect urea addition products and the half-life for urea decomposition is 40 years at 25 °C the above-described enhancement mechanisms seem to be unlikely for the presented phosphorylation reaction in liquid SO_2_ in the investigated time period^74^. However, urea interacts non-covalently with nucleobases in aqueous solution and the solubility of nucleosides in water is enhanced in the presence of urea^75,76^. Thus, enhancement mechanisms that are based on the physicochemical properties of urea cannot be ruled out.

Furthermore, we examined the robustness of the P(III) phosphorylation from a prebiotic point of view. Prebiotically plausible conversions require robust product formation under simple reaction conditions. In addition, extraordinary performance at low reactant concentration is essential since limited quantities are often assumed for emergence of life scenarios. Thus, we tested lower concentrations in A with stoichiometric amounts of H_3_PO_3_. Figure 2c shows that 5′ AMP and 5′ ADP yields increased at lower A concentrations (Supplementary Figs. 34–43 and Supplementary Tables 11–16). At the same time, a robust phosphorylation was observed over a wide concentration range. Although, at higher concentrations the product spectrum narrowed (Supplementary Data 1).

Phosphorylation proceeds at mild reaction temperature in liquid SO_2_. Detection of inorganic phosphate and the SO_2_ reduction products thiosulfate and dithionite shows that the reaction medium enables P(III) oxidation at the same time (Supplementary Figs. 78, 79). Reaction mixtures were colourless before the addition of SO_2_. After the reaction they had turned yellow which is possibly the result of SO_2_ reduction to elemental sulfur (Supplementary Fig. 80). Moreover, sulfate was observed which is proposed to be the product of SO_2_ disproportionation. As a result of the sulfur chemistry thiophosphonate/-phosphate analoga of A-p^III^ and A-p^V^-p^III^/p^III^-A-p^V^ were detected as side products in the reaction (Supplementary Data 1).

### Time dependence and reaction pathways of the phosphorylation

**Fig. 3:**
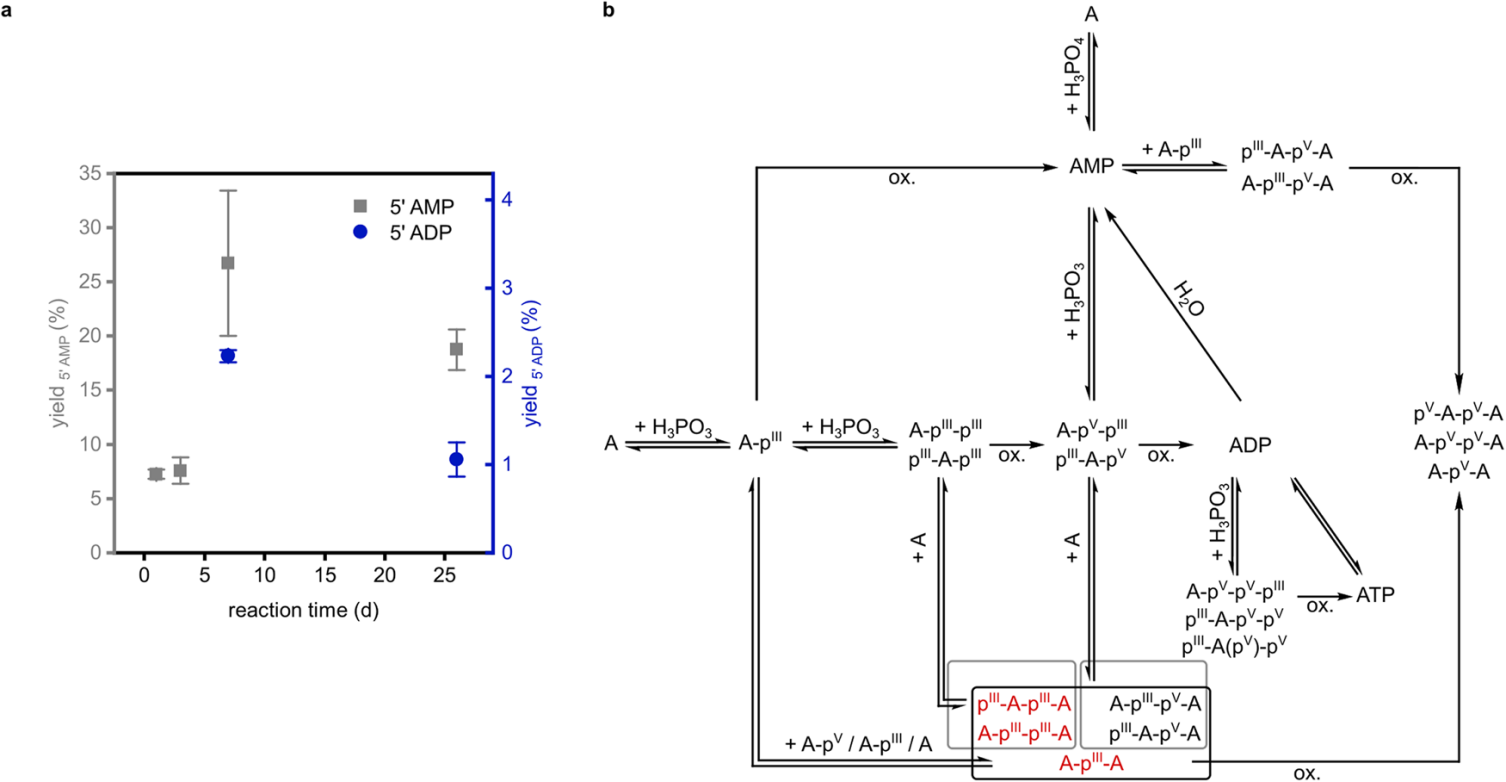
Reaction time dependence and reaction pathways of the phosphorylation. **a** Yields depending on the reaction time starting from A (100 mM), H_3_PO_3_ (3.0 eq.) and urea (1.0 eq.). Depicted are mean values, error bars refer to ± s.d and were obtained by double determination via CE (for yield calculation see supplementary information). **b** Proposed reaction pathways for phosphorylated compounds. Constitution of potential isomers has not been determined. Labels refer to the entirety of all observed isomers (black = m/z value found; red = m/z value not detected; oxidation: ox.). For simplification and since we did not detect the A-p^V^-p^III^-p^III^/ p^III^-A-p^V^-p^III^/p^III^-A(p^V^)-p^III^ intermediate, we did not show the inverse reaction sequence (first condensation, second oxidation) for the reaction of A-p^V^-p^III^/p^III^-A-p^V^ to A-p^V^-p^V^-p^III^/p^III^-A-p^V^-p^V^/p^III^-A(p^V^)-p^V^.

Starting with the optimized reaction conditions, we studied the reaction progress over time. 7.3% 5’ AMP were already obtained after 1 d and the yield further increased within 7 d (Fig. 3a, Supplementary Figs. 44–47, and Supplementary Tables 17–20). Apart from monomeric species, (mixed) P(V) and P(III) based dimers (A-p^V^-A, p^V^-A-p^V^-A/A-p^V^-p^V^-A, p^III^-A-p^V^-A/A-p^III^-p^V^-A) and diphosphorylated species (ADP, A-p^III^-p^III^/p^III^-A-p^III^, A-p^V^-p^III^/p^III^-A-p^V^, p^V^-A-cp^V^/A-cp^V^-p^V^) were found within 1 d (Supplementary Data 1). ATP formation was detected after 7 d and we were able to quantify the 5′ ADP yield within the same period. After 26 d, decreased 5′ AMP (18.7%) and 5′ ADP (1.1%) quantities were observed and less P(III) based products were detected (Supplementary Figs. 48–51, Supplementary Tables 21–22, and Supplementary Data 1). Enhanced hydrolysis by water seems to be a conceivable reason. Another explanation for the latter observation is oxidation to the corresponding P(V) compounds over time.

We then sought to explore potential reaction sequences leading to (dimeric) nucleotides (proposed reaction pathways for phosphorylated compounds are displayed in Fig. 3b). Previously detected P(III) species suggest nucleoside condensation with H_3_PO_3_ prior to an oxidation step. As expected, replacement of H_3_PO_3_ with P(V) compounds led to inferior product formation since the nucleophilic attack at the P(V) centre is slower than at the P(III) centre because of the higher adjacent negative charge^9,49,77^. In reactions of A with phosphoric acid (H_3_PO_4_), only traces of AMP were obtained (Supplementary Data 1). A mixture consisting of A, 5′ ADP and urea showed hydrolysis to 5′ AMP (3.4%) and small amounts of 5′ ATP (0.5%), which is proposed to be formed by phosphate transfer (Supplementary Figs. 58, 59 and Supplementary Tables 27, 28). On the contrary, the reaction starting with 5′ AMP showed no product formation at all.

To further identify accessible pathways of the P(III) based reaction we investigated A nucleotides as reactants. Various ADP and ATP isomers were obtained by phosphorylation of 5′ AMP with H_3_PO_3_ in the presence of urea (Table 1). Although, yields of the 5 substituted products (5′ ADP and 5′ ATP) are smaller than of products (5′ AMP and 5′ ADP) which are accessible via the same number of coupling steps in the reaction starting with A (Supplementary Figs. 52, 53 and Supplementary Tables 23, 24). Detection of the mixed diphosphorylated species A-p^V^-p^III^/p^III^-A-p^V^ indicates that, similar to the nucleoside phosphorylation reactions, the phosphorylation of the mononucleotide proceeds via P(III) intermediates (Supplementary Data 1). Isomerization of 5′ AMP was not observed. Conversion of 5′ ADP with H_3_PO_3_ in the presence of urea led to intense hydrolysis of the diphosphate reactant (Table 1). The hydrolysis product 5′ AMP (68.8%) could be phosphorylated again (Supplementary Figs. 54–57, Supplementary Tables 25, 26). As a result, A-p^V^-p^III^/p^III^-A-p^V^ was detected. Further condensation and/or oxidation steps led to mixed triphosphorylated species (A-p^V^-p^V^-p^III^/p^III^-A-p^V^-p^V^/p^III^-A(p^V^)-p^V^), ATP, A-p^V^-p^V^-A/p^V^-A-p^V^-A and to the isomerization of 5′ ADP (Supplementary Data 1). Although, A-p^V^-p^III^-p^III^/p^III^-A-p^V^-p^III^/p^III^-A(p^V^)-p^III^ were not detected, condensation prior to oxidation is conceivable since phosphonate diester are rapidly converted to their P(V) analogues^49^.

**Table 1:** Substrates investigated in the phosphorylation reaction.

Substrate	Potential products	Products confirmed by co-injection	Yield (%)
5′ AMP^a^	**ADP**^**b, c**^	5′ ADP	5′ ADP: (2.0 ± 0.3)
	**ATP**^**c**^	5′ ATP	5′ ATP: (0.4 ± 0.2)
5′ ADP^a^	**AMP**^**c**^	5′ AMP	5′ AMP: (68.8 ± 3.7)
	**ATP**^**c**^	5′ ATP	5′ ATP: (1.1 ± 0.1)
A^a^	**AMP**^**c**^	5′/3′/2′ AMP	5′ AMP: (26.7 ± 6.7)
	**ADP**^**c**^	5′ ADP	5′ ADP: (2.2 ± 0.1)
	ATP		Traces
G^a^	**GMP**^**c**^	5′ GMP	5′ GMP: (7.1 ± 1.6)
	**GDP**^**c**^	5′ GDP	Traces
	GTP		Traces
C^a^	**CMP**^**c**^	5′ CMP	5′ CMP: (23.2 ± 2.4)
	**CDP**^**c**^	5′ CDP	5′ CDP: (3.0 ± 0.3)
	CTP		Traces
U^a^	**UMP**^**c**^	5′ UMP	5′ UMP: (29.6 ± 2.5)
	**UDP**^**c**^	5′ UDP	5′ UDP: (1.5 ± 0.0)
	UTP		Traces
dA^a^	dAMP		Traces
dG^a^	dGMP		Traces
dC^a^	**dCMP**^**c**^	5′ dCMP	5′ dCMP: (32.6 ± 5.5)
	**dCDP**^**c**^	5′ dCDP	n. d.^d^
	**dCTP**^**c**^	5′ dCTP	Traces
dT^a^	**dTMP**^**c**^	5′ dTMP	5′ dTMP: (31.7 ± 9.4)
	**dTDP**^**c**^	5′ dTDP	5′ dTDP: (3.6 ± 0.2)
	**dTTP**^**c**^	5′ dTTP	Traces
GL	**GL phosphate**^**e**^	GL-1-phosphate, GL-2-phosphate	n. d.^c^
	Cyclic GL phosphate		n. d.^c^
	GL phosphonate		n. d.^c^
	GL_2_ phosphate		n. d.^c^
	GL_2_ diphosphate		n. d.^c^
	GL_2_ triphosphate		n. d.^c^
	GL_3_ triphosphate		n. d.^c^
	Mixed (non-)cyclic GL diphosphate		n. d.^c^
	Mixed GL_2_ (non-)cyclic diphosphate		n. d.^c^
GA	GA phosphate		n. d.^c^
	GA phosphonate		n. d.^c^
	GA_2_ phosphonate		n. d.^c^
Rib	**Rib phosphate**^**e**^	Rib-5-phosphate	n. d.^c^
Sodium Lc	Lc phosphate		n. d.^c^
	Cyclic Lc phosphate		n. d.^c^
	Lc phosphonate		n. d.^c^
	Lc phosphate phosphonate		n. d.^c^
	Lc triphosphonate		n. d.^c^
	Lc_2_ phosphate		n. d.^c^
Ser	**Ser phosphate**^**e**^	O-phospho Ser	n. d.^c^
	Ser phosphonate		n. d.^c^
	Ser diphosphate		n. d.^c^
	Ser_2_ phosphate		n. d.^c^
	Ser_2_ diphosphate		n. d.^c^
	Ser triphosphate		n. d.^c^
	Ser triphosphonate		n. d.^c^

Substrates (100 mM, 1.0 eq.) were phosphorylated with H_3_PO_3_ (3.0 eq.) in the presence of urea (1.0 eq.) for 7 d. Yield errors refer to ± s.d. and were obtained by double determination via CE.

^a^Complete product mixture is displayed in Supplementary Data 1.

^b^For bold labelled products, at least one signal was assigned to a particular isomer by co-injection with a reference.

^c^CE measurement with co-injection.

^d^Not determined.

^e^CE-MS measurement with co-injection.

Finally, an investigation of potential reaction sequences showed that condensation steps are reversible under the presented reaction conditions. However, due to the reaction medium’s redox properties, oxidation steps are irreversible.

### Reaction with other nucleosides

Next, we tested the applicability of the phosphorylation to the other canonical nucleosides (Table 1). Starting with ribonucleosides under the optimized conditions, we found 5′ NMP and 5′ nucleotide diphosphate (5′ NDP) yields for the reaction of cytosine (C) and uridine (U) which are comparable to the phosphorylation of A (Supplementary Figs. 60–67 and Supplementary Tables 29–32). Furthermore, both product mixtures resembled the one observed in the reaction with A. N-p^V^-N was not detected neither for C nor for U, but in addition to the products obtained with A, mixed P(III) P(V) triphosphorylated species and in the reaction with C also mixed (non-)cyclic triphosphorylated compounds were observed (Supplementary Data 1). In analogy to phosphorylation of A, two signal sets were obtained for the dinucleotides. As in the case of A, the faster migrating species are the pyrophosphate linked dimers (N-p^V^-p^V^-N) whereas the slower ones could be assigned to dimers containing a phosphate linkage (p^V^-N-p^V^-N) (Supplementary Data 2). On the contrary to the phosphorylation of these nucleosides, the reaction with guanosine (G) yielded only 7.1% 5′ GMP and the amount of 5′ GDP was too small for quantification (Supplementary Figs. 68–70 and Supplementary Tables 33, 34). In comparison to the other ribonucleosides, a smaller product mixture was obtained, which contained non-cyclic monophosphorylated species, P(V) and P(III) derived diphosphorylated compounds, several guanosine triphosphate isomers and P(V) based dinucleotides (Supplementary Data 1). However, the latter (G-p^V^-p^V^-G/p^V^-G-p^V^-G) was only detected in trace amounts and therefore assignment of the corresponding signals by MS/MS measurements was not possible.

In the case of the deoxyribonucleosides, a significant reactivity difference was observed for purine and pyrimidine-based reactants. Reaction of both deoxyadenosine (dA) and deoxyguanosine (dG) provided only traces of monophosphorylated compounds. N-p^III^ and NMP species are the sole products (Supplementary Data 1). In contrast, yields of the reaction with pyrimidine-based deoxyribonucleosides even exceeded those of the reactions with ribonucleosides (32.6% 5′ deoxycytidine monophosphate (5′ dCMP) and 31.7% 5′ deoxythymidine monophosphate (5′ dTMP)) (Supplementary Figs. 71–77 and Supplementary Tables 35–38). Both product mixtures of deoxycytidine (dC) and deoxythymidine (dT) resembled the one derived from the reaction with A (Supplementary Data 1). In analogy to the ribonucleotide dimers, product peaks could be assigned to pyrophosphate (first signal set) and phosphate-linked species (second signal set) by MS/MS measurements (Supplementary Data 2). In liquid SO_2_ phosphorylated derivatives of all canonical nucleosides are accessible, however, product yield and chain length strongly depend on the particular nucleoside.

Reactions starting from (deoxy-)ribonucleosides except for dA, dC and dG showed the formation of thiophosphonate/-phosphate side products of N-p^III^ and/or mixed diphosphorylated compounds (N-p^V^-p^III^/p^III^-N-p^V^) (Supplementary Data 1).

### Phosphorylation of prebiotically relevant substrates

After nucleotide formation, we explored the application of the presented phosphorylation in a broader prebiotic context. Apart from nucleotide formation, the phosphorylation of sugars, alcohols, carboxylic and amino acids is essential from a prebiotic point of view, since the corresponding products participate in metabolic cycles or are essential for the formation of amphiphiles^5,7^. Thus, we selected glycerol (GL), glyceraldehyde (GA), D-ribose (Rib), sodium L-lactate (Lc), and L-serine (Ser) as test substrates. By phosphorylation in liquid SO_2_, we were able to obtain the acyclic monophosphates of all model compounds (Table 1, Supplementary Data 1). Co-injection of reference compounds confirmed the presence of both GL monophosphates, O-phospho-Ser (**2**), and the formation of Rib-5-phosphate among other constitutional isomers. Cyclic monophosphates were only detected in the reactions with GL and Lc. Furthermore, H-phosphonates were observed for all substrates except Rib. Consequently, reaction sequences already proposed for the nucleoside phosphorylation seem also conceivable for these substrates. Different P-containing dimers were obtained from the phosphorylation of GL, GA, Lc, and Ser. However, GA is the sole substrate that showed the formation of H-phosphonate diester-linked substrate units (GA-p^III^-GA). Furthermore, glyceric acid was observed as a result of GA oxidation. GL and Ser were the only substrates that yielded dimers such as p^V^-GL-p^V^-GL/GL-p^V^-p^V^-GL. The longest observed oligomer was GL_3_ triphosphate. In addition, up to triphosphorylated substrates were detected in the reactions of Lc (triphosphonate) and Ser (triphosphonate and -phosphate).

**Fig. 4:**
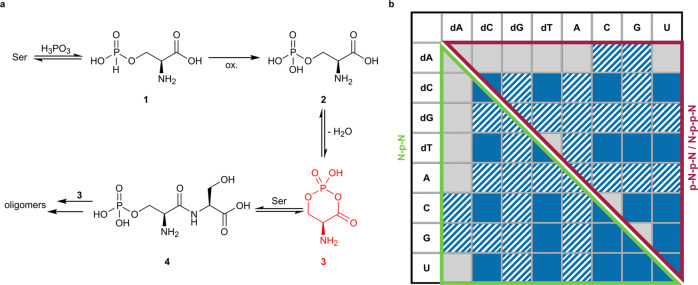
Peptide formation starting from Ser and reaction of nucleoside mixture. **a** Proposed mechanism for phosphate mediated peptide formation starting from Ser based on the mechanism for trimetaphosphate mediated amino acid coupling suggested by Rabinowitz et al. (black: m/z value found; red: m/z value not detected; oxidation: ox.)^80^. **b** Products of the reaction starting from a mixture containing all canonical nucleosides (each 25 mM, in total 1.0 eq.) with H_3_PO_3_ (3.0 eq.) and urea (1.0 eq.) after 7 d. The reaction mixture was analyzed by CE-MS (blue: detected, grey: not detected, hatched: several nucleoside combinations with identical m/z values are possible and consequently the signal could not be assigned unambiguously to one nucleoside pair).

Moreover, in the case of Ser the detection of Ser_2_ and Ser_2_ phosphate (**4**) are noteworthy. Peptide formation promoted by phosphates is well known in literature^78,79^. However, the effect has not been described for solutions of liquid SO_2_ yet. Previous peptide formations in this reaction medium required the presence of a metal^65^. In analogy to a proposed mechanism for amino acid condensation enhanced by trimetaphosphate, we suggest the formation of a cyclic intermediate (**3**) by intramolecular condensation of **2** prior to the nucleophilic attack of a second Ser unit and concomitant ring opening which leads to **4** (Fig. 4a)^80^.

### Phosphorylation of a nucleoside mixture

To mimic a more complex early Earth scenario, we investigated the phosphorylation of a mixture containing all canonical deoxy- and ribonucleosides. Mass spectrometric analysis of the product mixture after CE separation showed signals for many N-p^V^-N and N-p^V^-p^V^-N/p^V^-N-p^V^-N combinations. m/z values of most dC, dT, C, G, and U containing ribonucleoside, deoxyribonucleoside and mixed ribonucleoside-deoxyribonucleoside dimers could be unambiguously assigned to a single nucleoside pair (Fig. 4b and Supplementary Data 1). However, in the case of the remaining dimers identical m/z values prevented unambiguous determination of the building blocks. In analogy to the phosphorylation of single nucleosides, EIEs of all detected dinucleotides displayed two signal sets. Thus, we assume that both pyrophosphate (N-p^V^-p^V^-N) and phosphate (p^V^-N-p^V^-N) linked dimers were formed. Although, low product concentration prevented confirmation by MS/MS analysis. Analogous to the phosphorylation reactions of single nucleosides, dA showed the worst performance. Only m/z values of dimers with C and G were detected. However, there are other dimers with identical m/z values (e.g., A-dC, dG-dC and A-dG, dG-dG, A-A based dinucleotides). Consequently, we were not able to state whether dA-containing products were present. The same applies for dG and A containing products.

Apart from the RNA world hypothesis a heterogeneous RNA/DNA world scenario is discussed^81^. In such a hypothetical heterogeneous RNA/DNA world, the transformation of the observed heterogeneous dinucleotides to homogeneous oligomers over time might be a conceivable scenario since duplexes with heterogeneous backbone structure are less stable than their homogeneous analogues^82^. Consequently, heterogeneous templates prevent template product inhibition, which is well known in non-enzymatic replication with homogeneous templates^82,83^. Furthermore, heterogeneous templates prefer homogeneous substrates and Krishnamurthy et al. proposed that the sequence information of heterogeneous oligonucleotides might be heritable^83^.

## Discussion

We identified a prebiotically conceivable and efficient phosphorylation route in liquid SO_2_ which proceeds at ambient reaction temperature. Phosphorous acid as P(III) source in combination with the redox active reaction medium SO_2_ provides organo-monophosphates in a single reaction step in good yields. In addition, we show that dinucleotide species are directly accessible because hydrolysis-sensitive phosphonate esters are immediately converted to their more stable phosphate analogues. In contrast to that, previous P(III) based studies required both elevated temperatures and either an additional oxidation or desulfurization step^15,32,33^.

In the absence of steric constraints, pyrophosphate-linked dimers and a variety of phosphate bridged regioisomers were detected apart from the naturally occurring 3′-5′ dinucleotide. Previous studies showed that pyrophosphates can also be obtained by internal cleavage of an RNA strand and subsequent extension of the formed primer^84^. Application of the resulting single strand as a template in RNA polymerization can lead to canonical 3′-5′ phosphodiester linkages. However, replication is slower than with canonical templates. Based on these observations and the reduced stability of RNA single strands which contain 3′-5′ pyrophosphate linked units in the presence of Mg^2+^ Szostak et al. proposed strands with pyrophosphate linkages as temporary band-aid for cleaved strands^84^.

Recent investigations reported backbone heterogeneities because of 2′-5′ phosphate-linked units. Those linkages still allow RNA folding and thus molecular recognition and catalytic features^85^. Furthermore, similar to nucleobase-based backbone heterogeneity, the thermal stability of RNA duplexes which contain 2′-5′ linkages is decreased and as a consequence template product inhibition during the replication process is prevented^85,86^. Nevertheless, in comparison to their 3′-5′ linked analogues, 2′-5′ linkages are more sensitive towards hydrolysis, and they are extended slower^87,88^. Hence, disappearance of the 2′-5′ linkages over time and replacement by 3′-5′ linkages will be observed^88^.

Consequently, the formation of various isomers apart from canonical 3′-5′ phosphodiesters in liquid SO_2_ does not seem to be detrimental in view of the following replication steps. Instead, the presence of non-canonical linkages is suggested to be advantageous for template-directed polymerization and in the case of internal strand cleavage^84–86^.

In addition, reaction in liquid SO_2_ led to the phosphorylation of all canonical nucleosides and several biochemically relevant substrates. In the case of Ser even the formation of the corresponding dipeptide could be observed. Moreover, the approach seems to be compatible to complex scenarios for the early Earth since phosphorylation of a large reaction mixture yielded various dinucleotides. Advantageous for the emergence of life is also the reversibility of the condensation. Apart from phosphorylation, decomposition and isomerisation of organophosphates are essential processes in view of metabolic activity^48,89^. Alternatively, organophosphates could have been transferred from liquid SO_2_ to other environments which are more favourable for the evolution of the first living organisms. A rise in temperature or a decrease in pressure led to the evaporation of SO_2_. Organophosphates which were left behind in the process could then be dissolved in water and participate in metabolic activity.

In summary, liquid SO_2_ is a suitable reaction medium for organophosphate formation at ambient temperature starting with P(III). Furthermore, the enhancement of amino acid coupling by P species is of great interest from a prebiotic point of view. Conceivable scenarios for the early Earth often involve different monomers at the same reaction site and thus, pathways that do not only tolerate other monomers but even allow their incorporation into biopolymers are of great value. As a next step, cooperative interaction between the different accessible biopolymers might provide essential functions for living organisms. Apart from the prebiotic application, the reaction offers potential for classical organic synthesis because of the recyclability of SO_2_ and the use of simple reactants.

## Methods

### Phosphorylation reactions

A stainless steel apparatus (Supplementary Figs. 1, 2) was evacuated and purged with nitrogen thrice. Afterwards SO_2_ was condensed into the storage chamber at −76 °C and the valves were closed. The SO_2_ volume in the storage chamber was calculated from the weight difference between the empty and the SO_2_ filled apparatus. A or optionally another substrate (80.2 mg, 300.0 µmol, 1.0 eq.), urea (18.0 mg, 300.0 µmol, 1.0 eq.), and H_3_PO_3_ (73.8 mg, 900.0 µmol, 3.0 eq.) were placed in the reaction chamber of a stainless steel pressure apparatus (Supplementary Fig. 1). In the case of the nucleoside mixture equimolar amounts of A, C, G, U, dA, dC, dG, dT (in total 600.0 µmol, 1.0 eq.), H_3_PO_3_ (147.6 mg, 1.8 mmol, 3.0 eq.) and urea (36.0 mg, 600.0 µmol, 1.0 eq.) were inserted into the reaction chamber. The reaction chamber was evacuated and purged with nitrogen thrice. The reaction chamber was cooled to −76 °C. Valve 1 was opened and SO_2_ (3 mL) from the storage unit was condensed on to the reactants. Valve 1 was closed and the cooling was removed from the reaction side. The reaction proceeded at room temperature under stirring for 1–26 d. Afterwards the storage unit was cooled to −76 °C. Valve 1 was opened and the solvent was condensed into the storage unit to enable its reuse in up to four more reactions. The product mixture was dried *in vacuo* and was stored at −20 °C.

### CE-MS/MS analysis

CE-MS analyses were performed on an Agilent CE 7100 which was coupled to a Thermo Scientific Orbitrap Q Exactive Plus mass spectrometer. The customized sheath-flow interface is described elsewhere^67^. Samples were diluted to a nucleoside concentration of 5 mM referring to the initial amount of one nucleoside. A separation method from Rodríguez-Gonzalo et al. was adapted to analyze the product mixtures^90^. Electrophoretic separations were performed on bare fused silica (BFS) capillaries (*l* = 80 cm) in the positive polarity mode at 25 °C by applying 25 kV to the CE inlet. 30 mM NH_4_FA (pH 9.5) was used as background electrolyte (BGE). The outer polyimide coating of the capillaries was removed at the MS end prior to first use. New capillaries were flushed with deionised water, aqueous NaOH (0.1 M), deionised water (each 5 min) and BGE (3 min). Between measurements capillaries were conditioned with deionised water, aqueous NaOH (0.1 M), deionised water (each 2 min) and BGE (3 min). Samples were injected pressure driven (30 mbar for 10 s). An electrospray for the MS analysis was established by providing a sheath liquid consisting of deionised water and isopropanol (1:1) with 0.05% v/v formic acid (3 µL/min) and applying a voltage of −4.5 kV to the stainless steel emitter. Analytes were detected in negative mode with a resolution of 140,000 in a mass range of either m/z 50 to 750 or m/z 80 to 1200. For MS analysis, the temperature of the ion transfer capillary was set to 140 °C and the S-lens RF level was adjusted to 50. For further analysis of dinucleotide sequences, data-dependent MS/MS analysis with inclusion lists was performed. For dinucleotide fragmentation, a normalised collision energy of 30% was applied. The resolution of MS/MS spectra was adjusted to 17,500. Thermo Xcalibur software 4.1 was used for data evaluation.

### Nucleotide quantification

Nucleotides were quantified by CE measurements. CE analyses were performed on an Agilent CE 7100. Samples were diluted to 0.2–1 mM referring to the initial nucleoside concentration. Product mixtures were analysed on BFS capillaries (l = 80 cm, length to detector: 71.5 cm) in the positive polarity mode (Supplementary Fig. 3). Separations were performed at 25 °C with 30 mM ammonium formate (NH_4_FA) (pH 9.5) as BGE by applying 30 kV to the CE inlet. Conditioning of new capillaries with deionised water, aqueous NaOH (0.1 M), deionised water (each 5 min), and BGE (each 3 min) ensured proper analyses. Between separation runs, capillaries were conditioned with deionised water, aqueous NaOH (0.1 M), deionised water (each 2 min), and BGE (3 min). Samples were injected pressure driven (30 mbar for 10 s) and analytes were detected at 254 nm. Mono-, di- and triphosphate calibration curves were recorded in triplicates (Supplementary Figs. 5–15).

### Identification of inorganic sulfur compounds

Identification of inorganic sulfur compounds was accomplished by CE analyses. Measurements were performed with an UV-active BGE consisting of 30 mM bis(2-hydroxyethyl)amino-tris(hydroxymethyl)methan (BIS-TRIS) and 20 mM salicylic acid (pH 6)^44^. Separations were performed at 25 °C on BFS capillaries (*l* = 80 cm, length to detector: 71.5 cm) by applying −30 kV to the CE inlet. Analyses were conducted pressure assisted (40 mbar). New capillaries were conditioned with deionised water, aqueous NaOH (0.1 M), deionised water (each 5 min) and BGE (3 min). Samples were injected pressure driven (20 mbar for 10 s) and analytes were detected indirectly at 214 nm. CE electropherograms were evaluated using CEval 0.6 g^91^ and plotted with OriginPro 2018G.

## Supplementary information

Trapp_PR File

Supplementary Information

Description of Additional Supplementary Files

Supplementary Data 1

Supplementary Data 2

## Data Availability

The main data that support the findings of this study are available in the Supplementary information files (Supplementary information, Supplementary Data 1 and 2). Extra data are available from the corresponding author upon reasonable request.
