# Supplementary material for: Phosphorylation in liquid sulfur dioxide under prebiotically plausible conditions
Source: Commun Chem. 2022 Nov 3;5:143. doi: 10.1038/s42004-022-00761-w (PMC9814524; doi:10.1038/s42004-022-00761-w)
Supplement: Supplementary file 2 — Supplementary Information [file 42004_2022_761_MOESM2_ESM.pdf]

Supplementary Information

Phosphorylation in liquid sulphur dioxide under prebiotically plausible conditions

Constanze Sydow[1], Christiane Seiband[1], Alexander F. Siegle[1], Oliver Trapp[*1,2]

[1] Department of Chemistry and Pharmacy, Ludwig-Maximilians-University, Butenandtstr. 5-13, 81377 Munich, Germany

[2] Max-Planck-Institute for Astronomy, Königstuhl 17, 69117 Heidelberg, Germany

[*] Correspondence: oliver.trapp@cup.uni-muenchen.de

Contents

Supplementary Methods 3
Materials 3
Pressure apparatus 3
Supplementary Note 1 5
Quantitative evaluation of phosphorylated compounds 5
Model CE electropherogram 5
Calibration plots 6
Reactions with adenosine 12
Reaction with 5’ AMP 41
Reactions with 5’ ADP 43
Reaction with cytidine 48
Reaction with uridine 51
Reaction with guanosine 54
Reaction with deoxycytidine 56
Reaction with deoxythymidine 58
Supplementary Note 2 61
Sulphur redox chemistry 61

Supplementary Methods

Materials

Chemicals were ordered from Sigma Aldrich, TCI, Alfa Aesar and abcr chemicals in analytical grade and were used without further purification. 5'-adenylic acid-3',5'-adenosine phosphate (5'-p^V-A-3'-p^V-5'-A) was obtained from metabion. Aqueous solutions were prepared with deionized water obtained from a VWR Puranity PU 15 (VWR, Leuven, Belgium). Sulphur dioxide (SO_2) N38 (99.98%) was purchased from Air Liquide Germany.

Pressure apparatus

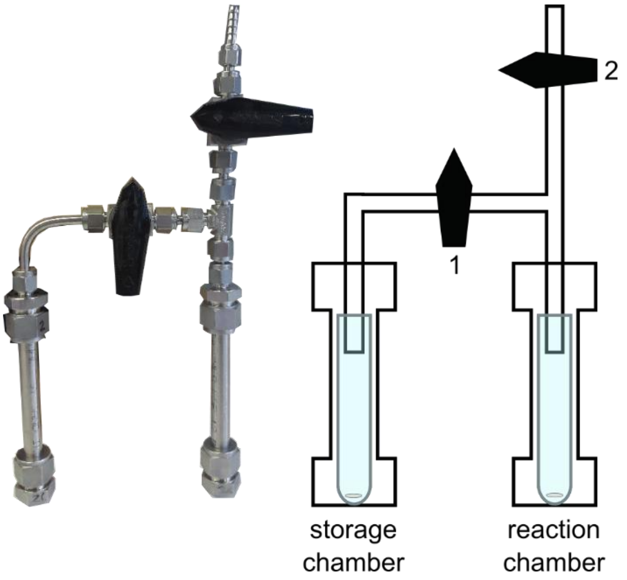

Supplementary Figure 1. Stainless steel pressure apparatus for phosphorylation reactions in liquid SO_2. subunits contain 3.5 mL test tubes and magnetic stir bars.

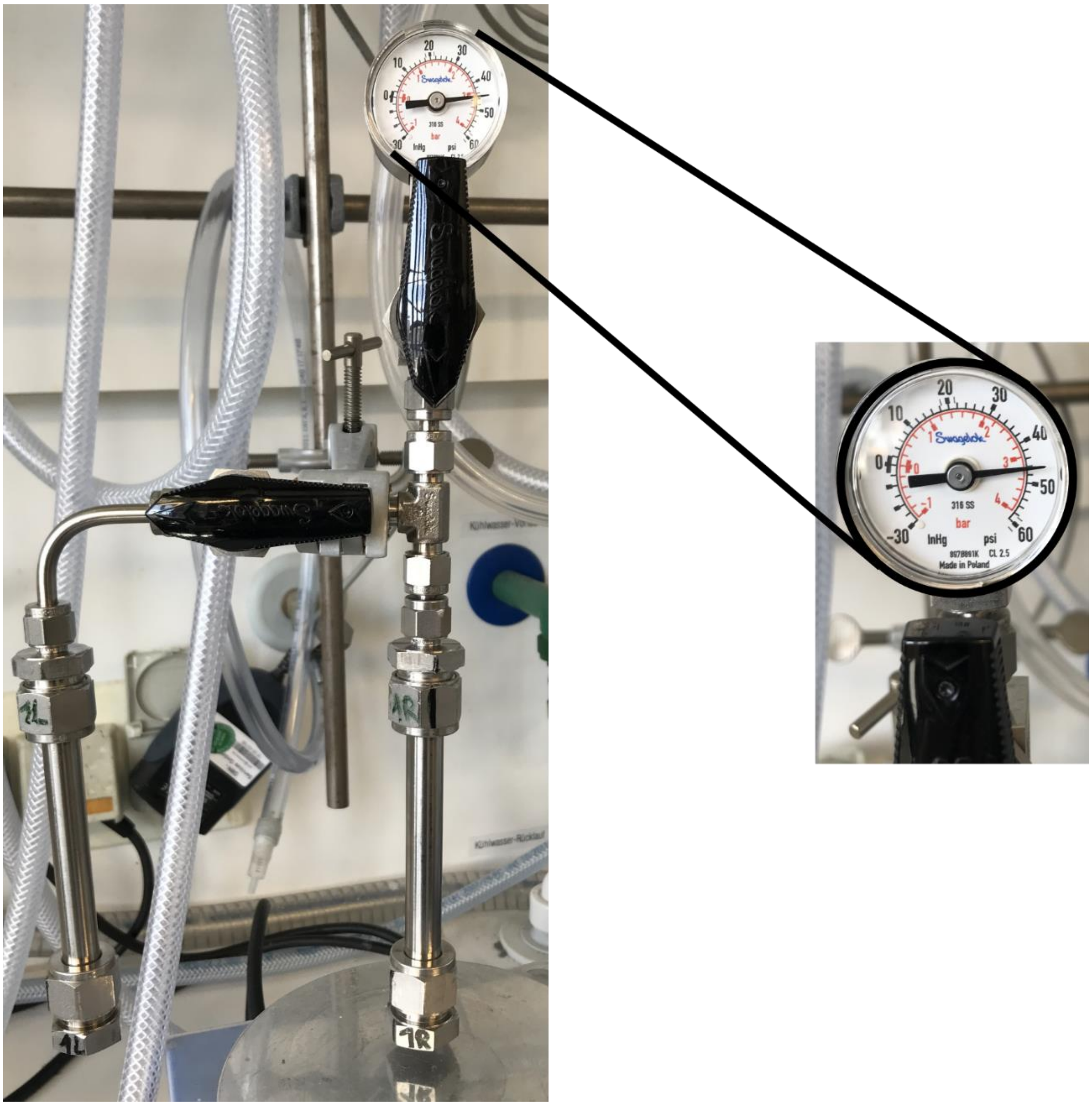

Supplementary Figure 2. Pressure in a stainless steel apparatus filled with 3.7 g SO_2 at room temperature.

Supplementary Note 1

Quantitative evaluation of phosphorylated compounds

Model CE electropherogram

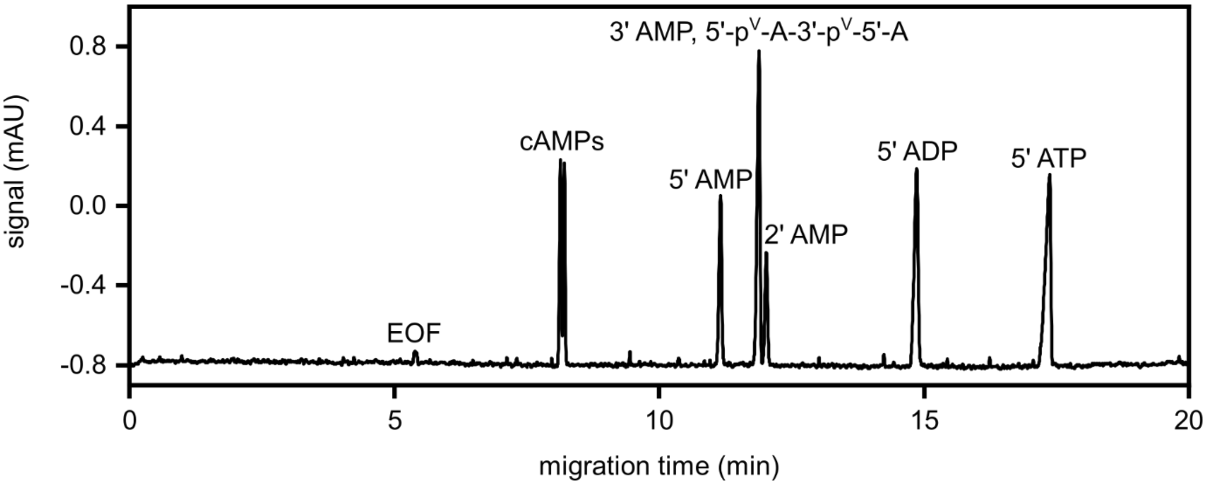

Supplementary Figure 3. Model CE electropherogram of adenosine phosphate species (each 25 µM) with labelled peaks (electroosmotic flow (EOF), cyclic adenosine monophosphates (cAMPs), 5’ adenosine monophosphate (5’ AMP), 3’ adenosine monophosphate (3’ AMP), 5’-adenylic acid-3’,5’-adenosine phosphate (5’-p^{V}-A-3’-p^{V}-5’-A), 2’ adenosine monophosphate (2’ AMP), 5’ adenosine diphosphate (5’ ADP) and 5’ adenosine triphosphate (5’ ATP). Analytes were detected at 254 nm. Conditions for the CE separation: bare fused silica (BFS) capillary (l = 80 cm, length to detector: 71.5 cm); background electrolyte (BGE): NH_4FA (30 mM, pH 9.5); CE inlet: 30 kV, pressure driven sample injection: 30 mbar for 10 s.

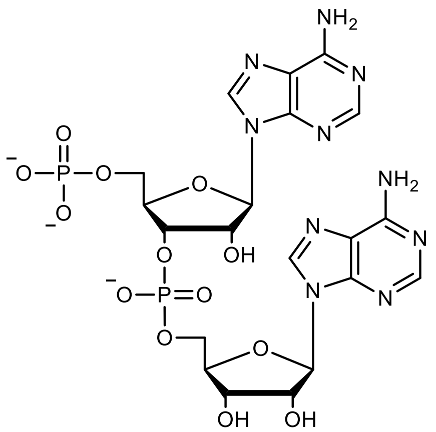

Supplementary Figure 4. Structure of 5’-adenylic acid-3’,5’-adenosine phosphate (5’-p^{V}-A-3’-p^{V}-5’-A).

Calibration plots

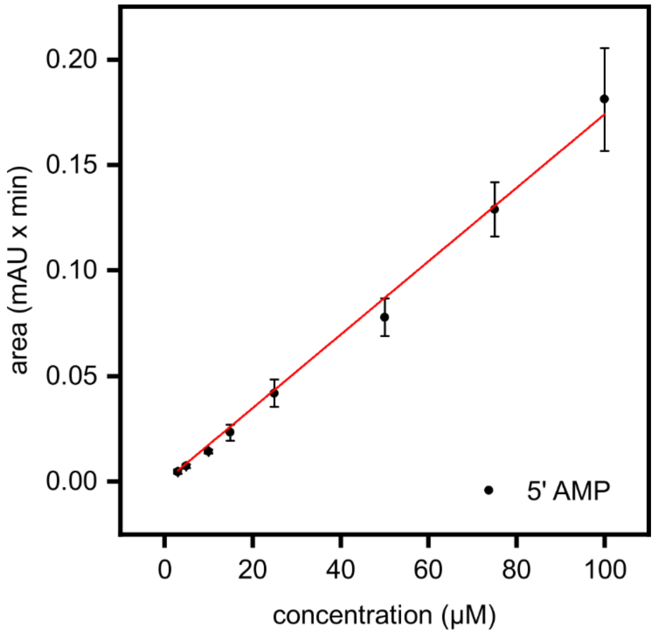

Supplementary Figure 5. Calibration plot of 5′ AMP (m = 0.00174 ± 3.42621E-5, R^2 = 0.997). 5′ AMP was detected at 254 nm. Calibration curve was recorded in triplicates. Conditions of the electrophoretic separation: BFS capillary (l = 80 cm, length to detector: 71.5 cm); BGE: NH_4FA (30 mM, pH 9.5); CE inlet: 30 kV, pressure driven sample injection: 30 mbar for 10 s, detection at 254 nm.

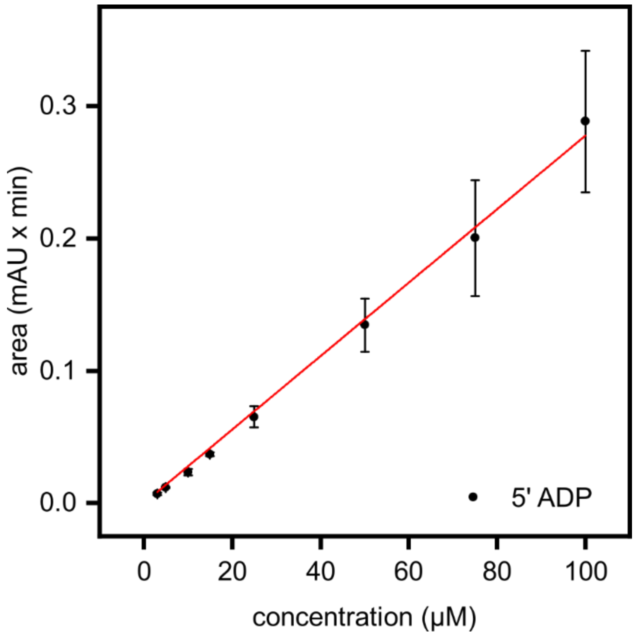

Supplementary Figure 6. Calibration plot of 5′ ADP (m = 0.00278 ± 4.41685E-5, R^2 = 0.998). 5′ ADP was detected at 254 nm. Calibration curve was recorded in triplicates. Conditions for the CE separation: BFS capillary (l = 80 cm, length to detector: 71.5 cm); BGE: NH_4FA (30 mM, pH 9.5); CE inlet: 30 kV, pressure driven sample injection: 30 mbar for 10 s, detection at 254 nm.

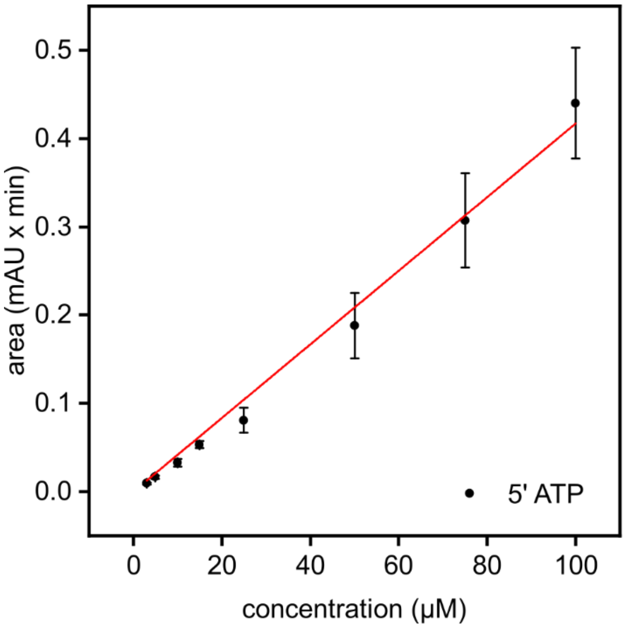

Supplementary Figure 7. Calibration plot of 5’ ATP (m = 0.00417 ± 1.14115E-4, R^2 = 0.994). 5’ ATP was detected at 254 nm. Calibration curve was recorded in triplicates. Conditions for the CE separation: BFS capillary (l = 80 cm, length to detector: 71.5 cm); BGE: NH_4FA (30 mM, pH 9.5); CE inlet: 30 kV, pressure driven sample injection: 30 mbar for 10 s, detection at 254 nm.

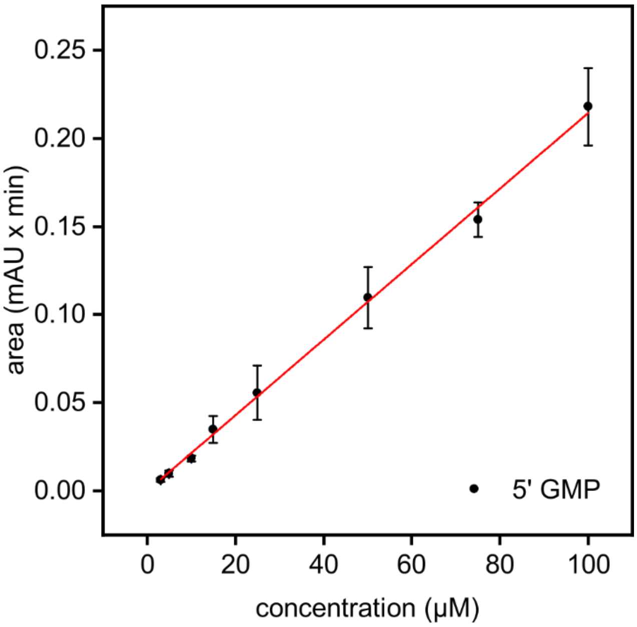

Supplementary Figure 8. Calibration plot of 5’ guanosine monophosphate (5’ GMP) (m = 0.00214 ± 2.57224E-5, R^2 = 0.998). 5’ GMP was detected at 254 nm. Calibration curve was recorded in triplicates. Conditions for the CE separation: BFS capillary (l = 80 cm, length to detector: 71.5 cm); BGE: NH_4FA (30 mM, pH 9.5); CE inlet: 30 kV, pressure driven sample injection: 30 mbar for 10 s, detection at 254 nm.

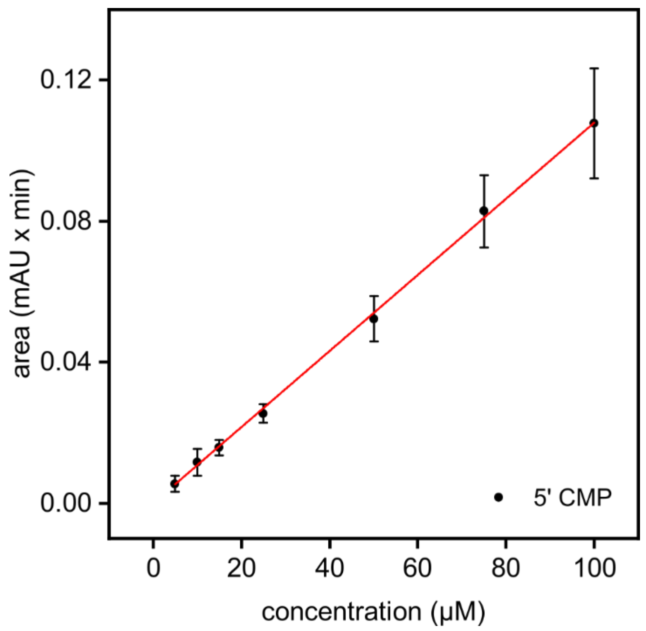

Supplementary Figure 9. Calibration plot of 5’ cytidine monophosphate (5’ CMP) (m = 0.00108 ± 9.19523E-6, R^2 = 0.999). 5’ CMP was detected at 254 nm. Calibration curve was recorded in triplicates. Conditions for the CE separation: BFS capillary (l = 80 cm, length to detector: 71.5 cm); BGE: NH_4FA (30 mM, pH 9.5); CE inlet: 30 kV, pressure driven sample injection: 30 mbar for 10 s, detection at 254 nm.

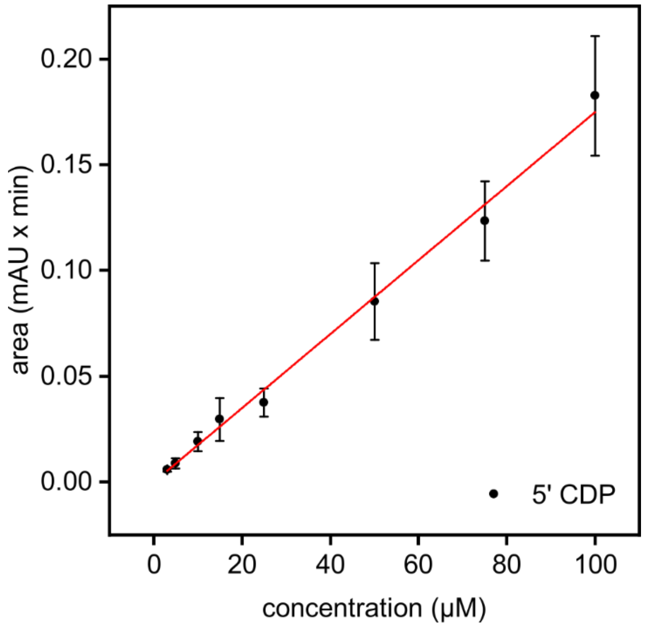

Supplementary Figure 10. Calibration plot of 5’ cytidine diphosphate (5’ CDP) (m = 0.00175 ± 3.63983E-5, R^2 = 0.996). 5’ CDP was detected at 254 nm. Calibration curve was recorded in triplicates. Conditions for the CE separation: BFS capillary (l = 80 cm, length to detector: 71.5 cm); BGE: NH_4FA (30 mM, pH 9.5); CE inlet: 30 kV, pressure driven sample injection: 30 mbar for 10 s, detection at 254 nm.

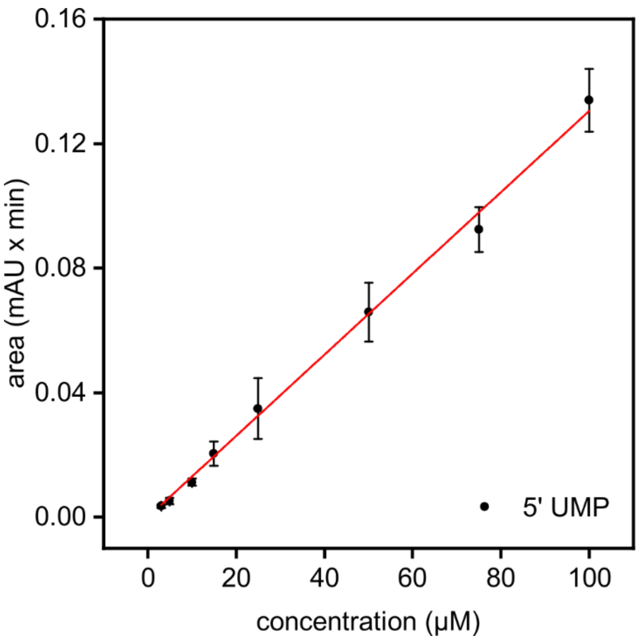

Supplementary Figure 11. Calibration plot of 5’ uridine monophosphate (5’ UMP) (m = 0.0013 ± 2.00939E-5, R^2 = 0.998). 5’ UMP was detected at 254 nm. Calibration curve was recorded in triplicates. Conditions for the CE separation: BFS capillary (l = 80 cm, length to detector: 71.5 cm); BGE: NH_4FA (30 mM, pH 9.5); CE inlet: 30 kV, pressure driven sample injection: 30 mbar for 10 s, detection at 254 nm.

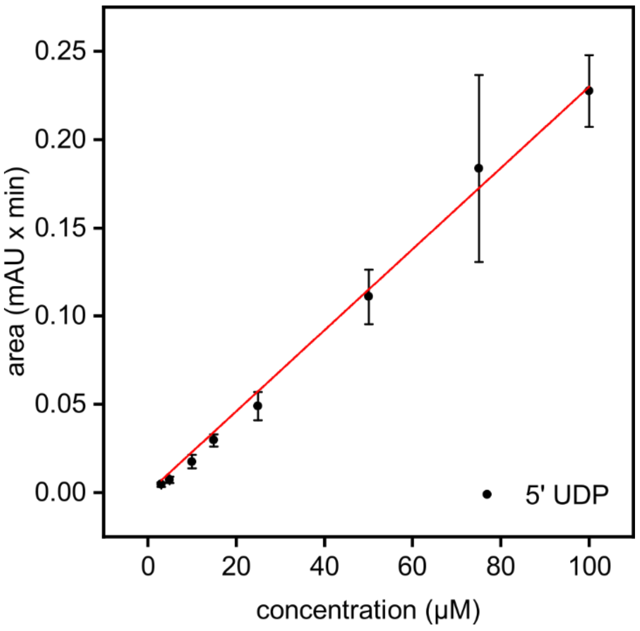

Supplementary Figure 12. Calibration plot of 5’ uridine diphosphate (5’ UDP) (m = 0.0023 ± 4.66751E-5, R^2 = 0.997). 5’ UDP was detected at 254 nm. Calibration curve was recorded in triplicates. Conditions for the CE separation: BFS capillary (l = 80 cm, length to detector: 71.5 cm); BGE: NH_4FA (30 mM, pH 9.5); CE inlet: 30 kV, pressure driven sample injection: 30 mbar for 10 s, detection at 254 nm.

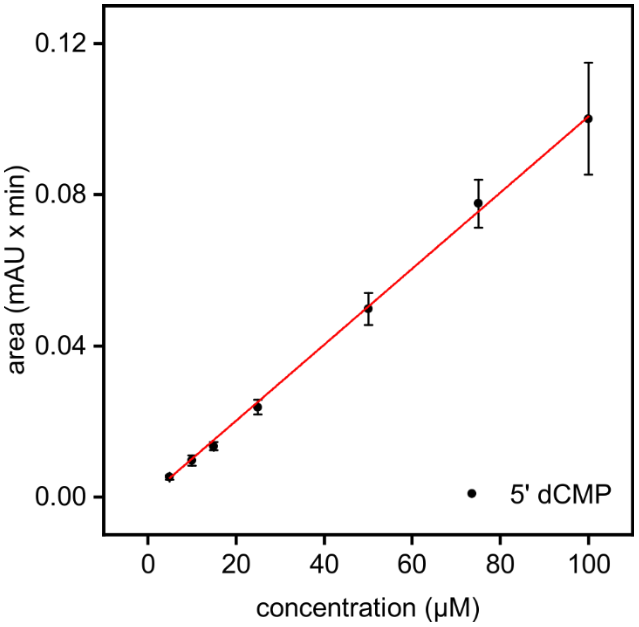

Supplementary Figure 13. Calibration plot of 5' deoxycytidine monophosphate (5' dCMP) (m = 0.00101 ± 9.44669E-6, R^2 = 0.999). 5' dCMP was detected at 254 nm. Calibration curve was recorded in triplicates. Conditions for the CE separation: BFS capillary (l = 80 cm, length to detector: 71.5 cm); BGE: NH_4FA (30 mM, pH 9.5); CE inlet: 30 kV, pressure driven sample injection: 30 mbar for 10 s, detection at 254 nm.

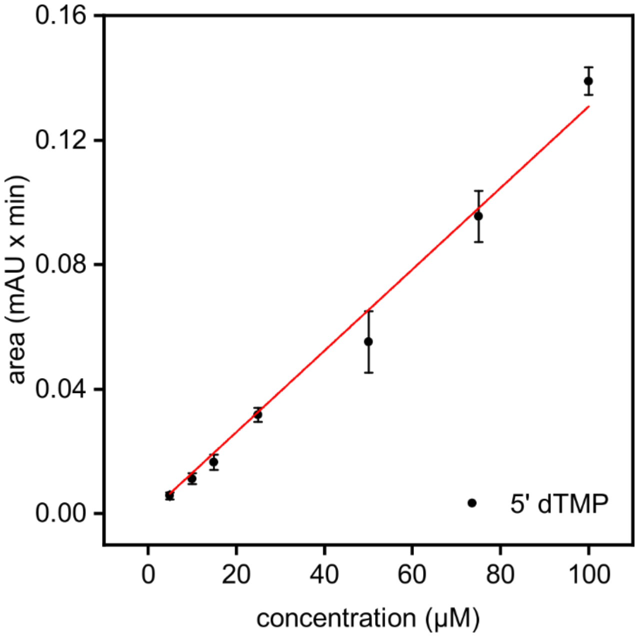

Supplementary Figure 14. Calibration plot of 5' deoxythymidine monophosphate (5' dTMP) (m = 0.00131 ± 4.08834E-5, R^2 = 0.994). 5' dTMP was detected at 254 nm. Calibration curve was recorded in triplicates. Conditions for the CE separation: BFS capillary (l = 80 cm, length to detector: 71.5 cm); BGE: NH_4FA (30 mM, pH 9.5); CE inlet: 30 kV, pressure driven sample injection: 30 mbar for 10 s, detection at 254 nm.

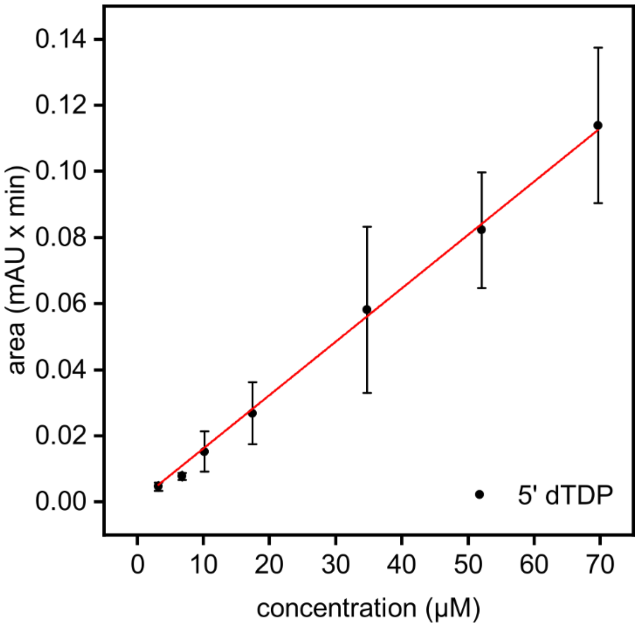

Supplementary Figure 15. Calibration plot of 5' deoxythymidine diphosphate (5' dTDP) (m = 0.00162 ± 2.08208E-5, R^2 = 0.999). 5' dTDP was detected at 254 nm. Calibration curve was recorded in triplicates. Conditions for the CE separation: BFS capillary (l = 80 cm, length to detector: 71.5 cm); BGE: NH_4FA (30 mM, pH 9.5); CE inlet: 30 kV, pressure driven sample injection: 30 mbar for 10 s, detection at 254 nm.

Reactions with adenosine

Variation of the H_3PO_3 concentration

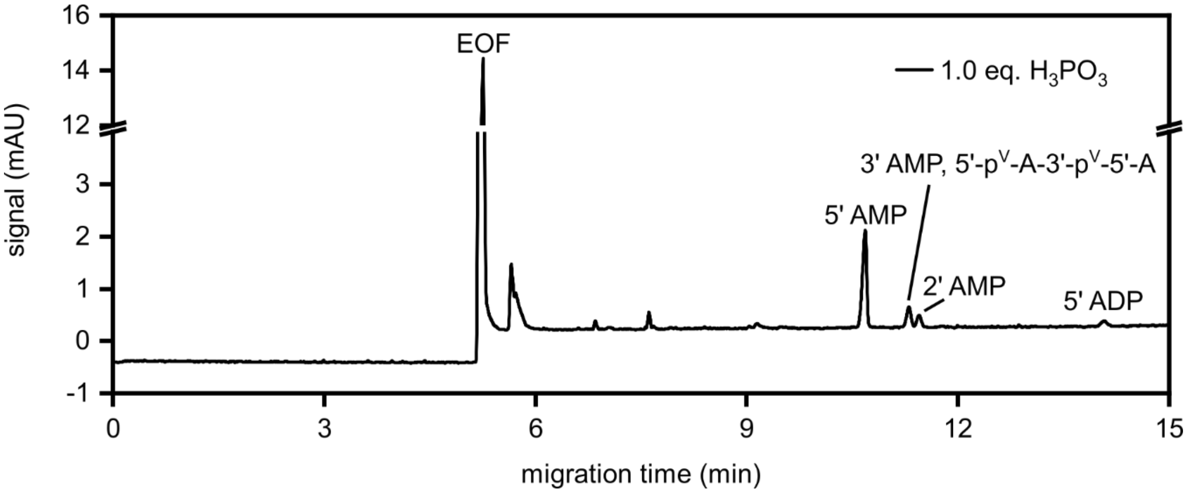

Supplementary Figure 16. First analysis run of the reaction starting from adenosine (A) (100 mM, 1.0 eq.) and H_3PO_3 (1.0 eq.) after 7 d. The sample was diluted to 1 mM referring to the initial A concentration. Conditions of the electrophoretic separation: BFS capillary (l = 80 cm, length to detector: 71.5 cm); BGE: NH_4FA (30 mM, pH 9.5); CE inlet: 30 kV, pressure driven sample injection: 30 mbar for 10 s, detection at 254 nm.

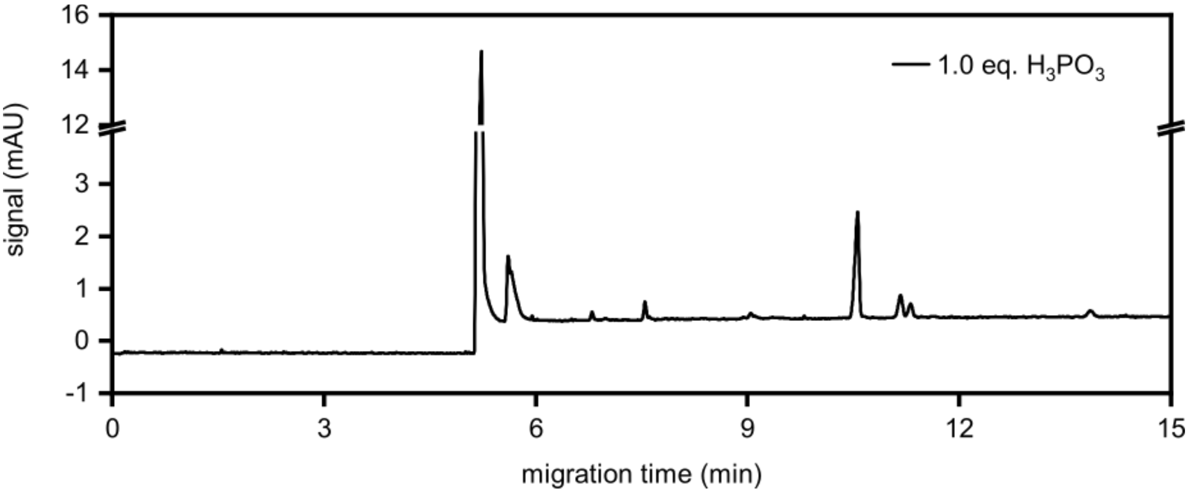

Supplementary Figure 17. Second analysis run of the reaction starting from A (100 mM, 1.0 eq.) and H_3PO_3 (1.0 eq.) after 7 d. The sample was diluted to 1 mM referring to the initial A concentration. Conditions of the electrophoretic separation: BFS capillary (l = 80 cm, length to detector: 71.5 cm); BGE: NH_4FA (30 mM, pH 9.5); CE inlet: 30 kV, pressure driven sample injection: 30 mbar for 10 s, detection at 254 nm.

Supplementary Table 1. First analysis run of the reaction starting from A (100 mM, 1.0 eq.) and H_3PO_3 (1.0 eq.) after 7 d. The sample was diluted to 1 mM referring to the initial A concentration.

Product	Integral (mAU x min)	$c_{sample\ vial}$ (µM)	$c_{reaction\ mixture}$ (mM)	Yield (%)
5’ AMP	0.1311	75.35	7.54	7.54
5’ ADP	0.0111	3.98	0.40	0.40

Supplementary Table 2. Second analysis run of the reaction starting from A (100 mM, 1.0 eq.) and H_3PO_3 (1.0 eq.) after 7 d. The sample was diluted to 1 mM referring to the initial A concentration.

Product	Integral (mAU x min)	$c_{sample\ vial}$ (µM)	$c_{reaction\ mixture}$ (mM)	Yield (%)	Ø Yield (%)
5’ AMP	0.1403	80.66	8.07	8.07	7.80 ± 0.38
5’ ADP	0.0124	4.46	0.45	0.45	0.42 ± 0.03

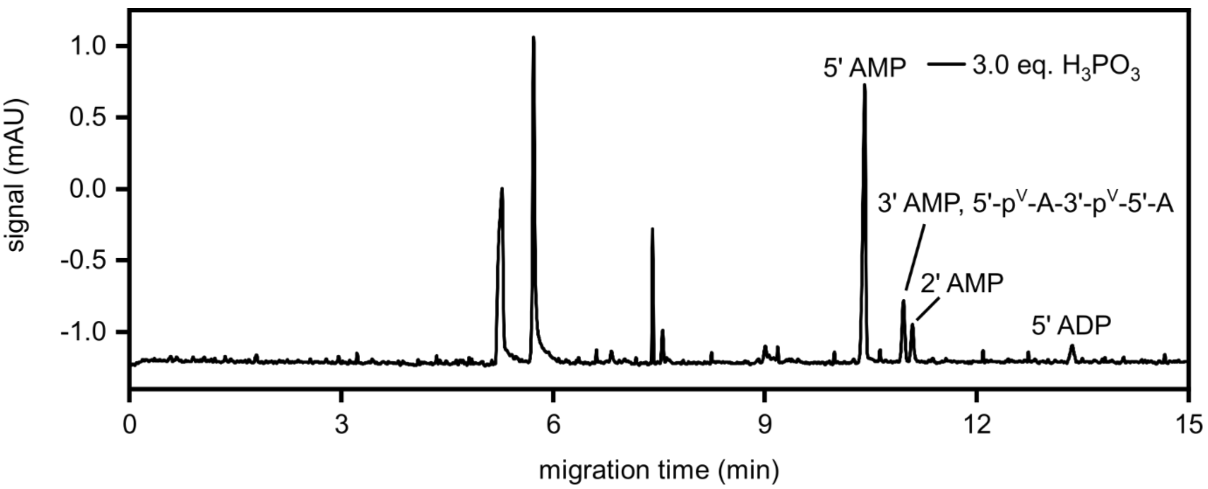

Supplementary Figure 18. First analysis run of the reaction starting from A (100 mM, 1.0 eq.) and H_3PO_3 (3.0 eq.) after 7 d. The sample was diluted to 300 µM referring to the initial A concentration for determination of the 5’ AMP yield. Conditions of the electrophoretic separation: BFS capillary (l = 80 cm, length to detector: 71.5 cm); BGE: NH_4FA (30 mM, pH 9.5); CE inlet: 30 kV, pressure driven sample injection: 30 mbar for 10 s, detection at 254 nm.

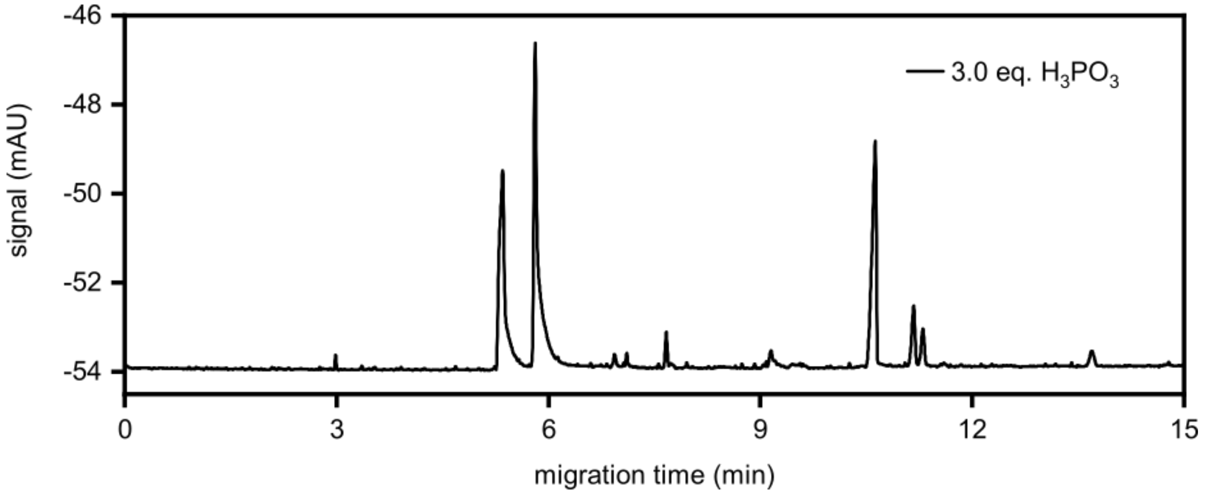

Supplementary Figure 19. First analysis run of the reaction starting from A (100 mM, 1.0 eq.) and H_3PO_3 (3.0 eq.) after 7 d. The sample was diluted to 1 mM referring to the initial A concentration for determination of the 5’ ADP yield. Conditions of the electrophoretic separation: BFS capillary (l = 80 cm, length to detector: 71.5 cm); BGE: NH_4FA (30 mM, pH 9.5); CE inlet: 30 kV, pressure driven sample injection: 30 mbar for 10 s, detection at 254 nm.

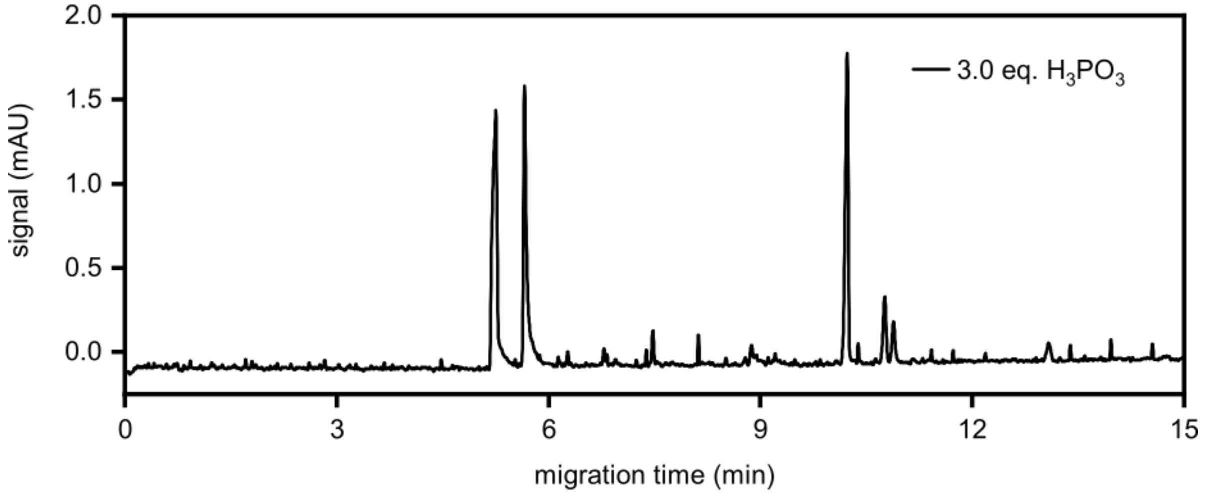

Supplementary Figure 20. Second analysis run of the reaction starting from A (100 mM, 1.0 eq.) and H_3PO_3 (3.0 eq.) after 7 d. The sample was diluted to 300 µM referring to the initial A concentration for determination of the 5' AMP yield. Conditions of the electrophoretic separation: BFS capillary (l = 80 cm, length to detector: 71.5 cm); BGE: NH_4FA (30 mM, pH 9.5); CE inlet: 30 kV, pressure driven sample injection: 30 mbar for 10 s, detection at 254 nm.

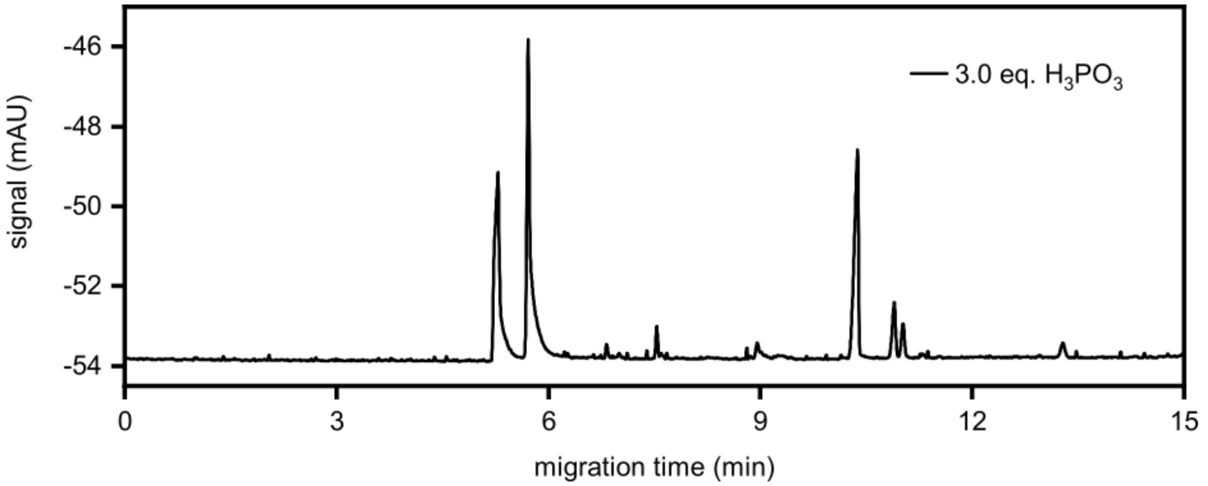

Supplementary Figure 21. Second analysis run of the reaction starting from A (100 mM, 1.0 eq.) and H_3PO_3 (3.0 eq.) after 7 d. The sample was diluted to 1 mM referring to the initial A concentration for determination of the 5' ADP yield. Conditions of the electrophoretic separation: BFS capillary (l = 80 cm, length to detector: 71.5 cm); BGE: NH_4FA (30 mM, pH 9.5); CE inlet: 30 kV, pressure driven sample injection: 30 mbar for 10 s, detection at 254 nm.

Supplementary Table 3. First analysis runs of the reaction starting from A (100 mM, 1.0 eq.) and H_3PO_3 (3.0 eq.) after 7 d. The sample was diluted to 300 µM referring to the initial A concentration for determination of the 5’ AMP yield and to 1 mM for determination of the 5’ ADP yield.

**Product**	**Integral** (mAU x min)	$c_{sample\ vial}$ (µM)	$c_{reaction\ mixture}$ (mM)	**Yield** (%)
5’ AMP	0.0955	54.87	18.29	18.29
5’ ADP	0.0245	8.81	0.88	0.88

Supplementary Table 4. Second analysis runs of the reaction starting from A (100 mM, 1.0 eq.) and H_3PO_3 (3.0 eq.) after 7 d. The sample was diluted to 300 µM referring to the initial A concentration for determination of the 5’ AMP yield and to 1 mM for determination of the 5’ ADP yield.

**Product**	**Integral** (mAU x min)	$c_{sample\ vial}$ (µM)	$c_{reaction\ mixture}$ (mM)	**Yield** (%)	**Ø Yield** (%)
5’ AMP	0.0912	52.42	17.47	17.47	17.88 ± 0.58
5’ ADP	0.0264	9.50	0.95	0.95	0.92 ± 0.05

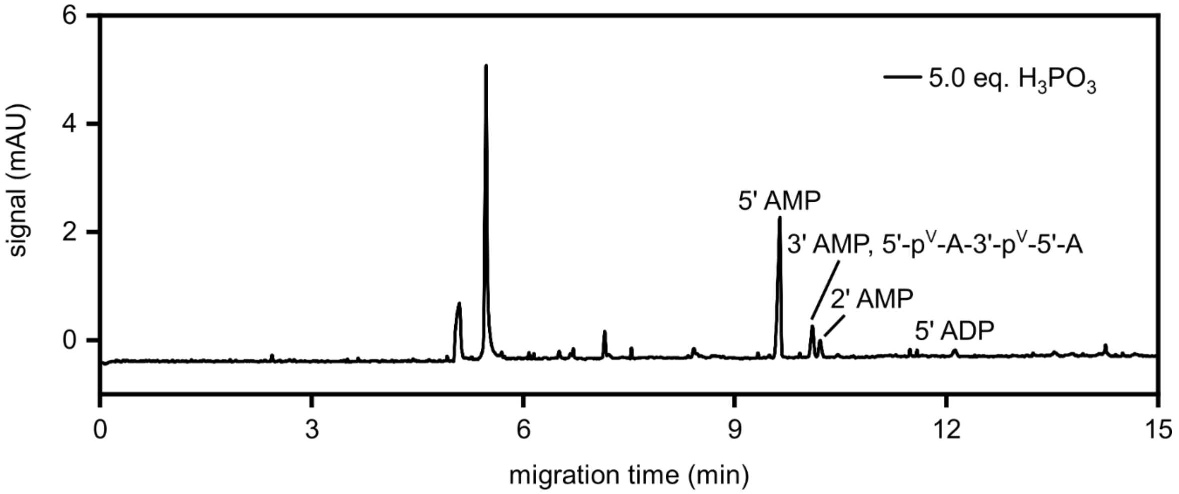

Supplementary Figure 22. First analysis run of the reaction starting from A (100 mM, 1.0 eq.) and H_3PO_3 (5.0 eq.) after 7 d. The sample was diluted to 500 µM referring to the initial A concentration for determination of the 5' AMP yield. Conditions of the electrophoretic separation: BFS capillary (l = 80 cm, length to detector: 71.5 cm); BGE: NH_4FA (30 mM, pH 9.5); CE inlet: 30 kV, pressure driven sample injection: 30 mbar for 10 s, detection at 254 nm.

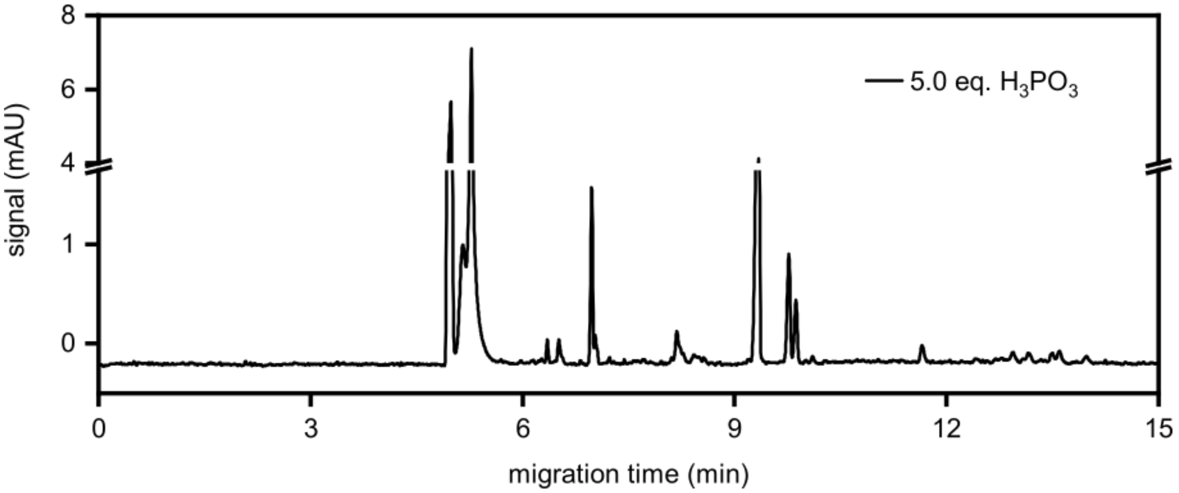

Supplementary Figure 23. First analysis run of the reaction starting from A (100 mM, 1.0 eq.) and H_3PO_3 (5.0 eq.) after 7 d. The sample was diluted to 1 mM referring to the initial A concentration for determination of the 5' ADP yield. Conditions of the electrophoretic separation: BFS capillary (l = 80 cm, length to detector: 71.5 cm); BGE: NH_4FA (30 mM, pH 9.5); CE inlet: 30 kV, pressure driven sample injection: 30 mbar for 10 s, detection at 254 nm.

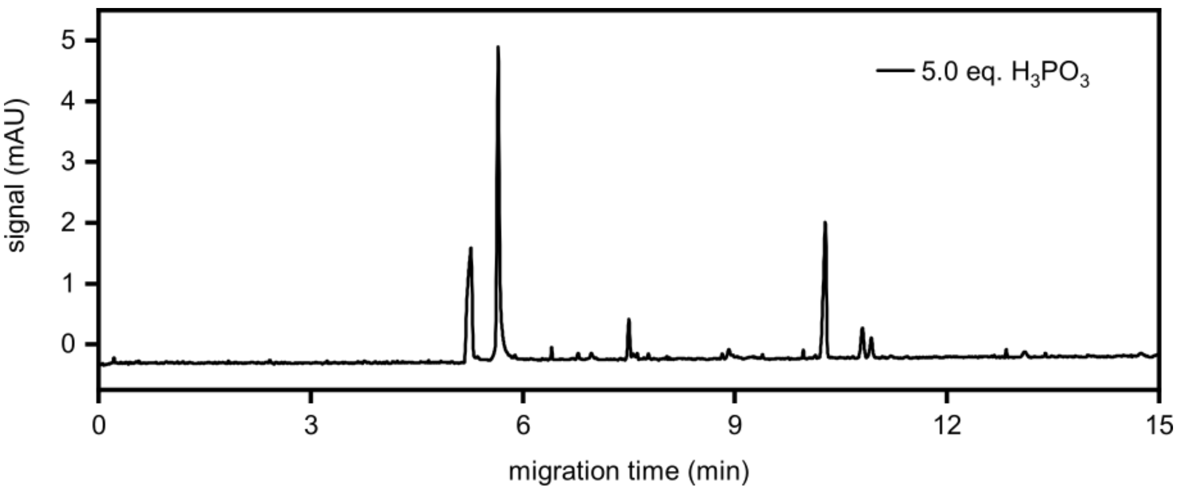

Supplementary Figure 24. Second analysis run of the reaction starting from A (100 mM, 1.0 eq.) and H_3PO_3 (5.0 eq.) after 7 d. The sample was diluted to 500 µM referring to the initial A concentration for determination of the 5' AMP yield. Conditions of the electrophoretic separation: BFS capillary (l = 80 cm, length to detector: 71.5 cm); BGE: NH_4FA (30 mM, pH 9.5); CE inlet: 30 kV, pressure driven sample injection: 30 mbar for 10 s, detection at 254 nm.

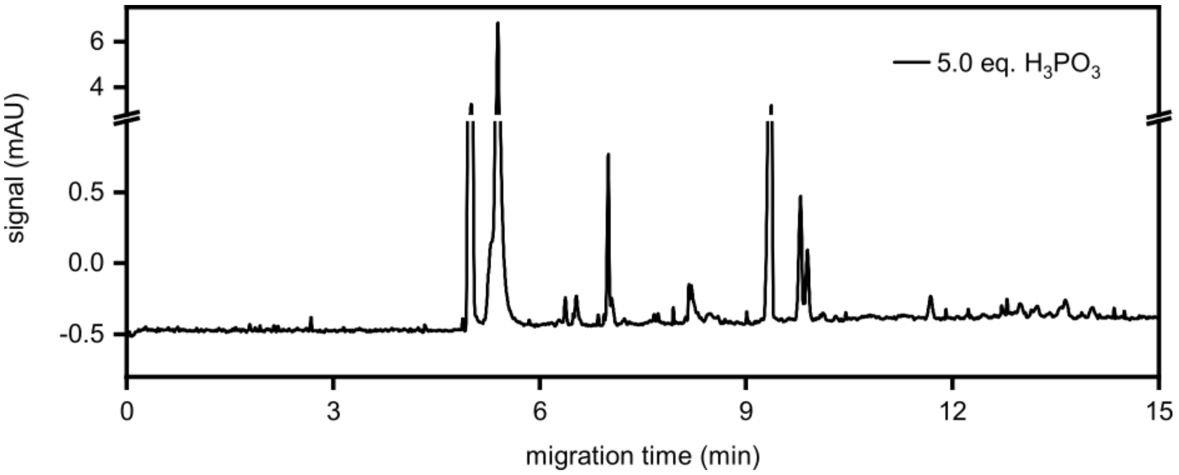

Supplementary Figure 25. Second analysis run of the reaction starting from A (100 mM, 1.0 eq.) and H_3PO_3 (5.0 eq.) after 7 d. The sample was diluted to 1 mM referring to the initial A concentration for determination of the 5' ADP yield. Conditions of the electrophoretic separation: BFS capillary (l = 80 cm, length to detector: 71.5 cm); BGE: NH_4FA (30 mM, pH 9.5); CE inlet: 30 kV, pressure driven sample injection: 30 mbar for 10 s, detection at 254 nm.

Supplementary Table 5. First analysis runs of the reaction starting from A (100 mM, 1.0 eq.) and H_3PO_3 (5.0 eq.) after 7 d. The sample was diluted to 500 µM referring to the initial A concentration for determination of the 5' AMP yield and to 1 mM for determination of the 5' ADP yield.

**Product**	**Integral** (mAU x min)	$c_{sample\ vial}$ (µM)	$c_{reaction\ mixture}$ (mM)	**Yield** (%)
5' AMP	0.1305	75.02	15.00	15.00
5' ADP	0.0103	3.72	0.37	0.37

Supplementary Table 6. Second analysis runs of the reaction starting from A (100 mM, 1.0 eq.) and H_3PO_3 (5.0 eq.) after 7 d. The sample was diluted to 500 µM referring to the initial A concentration for determination of the 5' AMP yield and to 1 mM for determination of the 5' ADP yield.

**Product**	**Integral** (mAU x min)	$c_{sample\ vial}$ (µM)	$c_{reaction\ mixture}$ (mM)	**Yield** (%)	**Ø Yield** (%)
5' AMP	0.1144	65.75	13.15	13.15	14.08 ± 1.31
5' ADP	0.0098	3.52	0.35	0.35	0.36 ± 0.01

Variation of the urea concentration

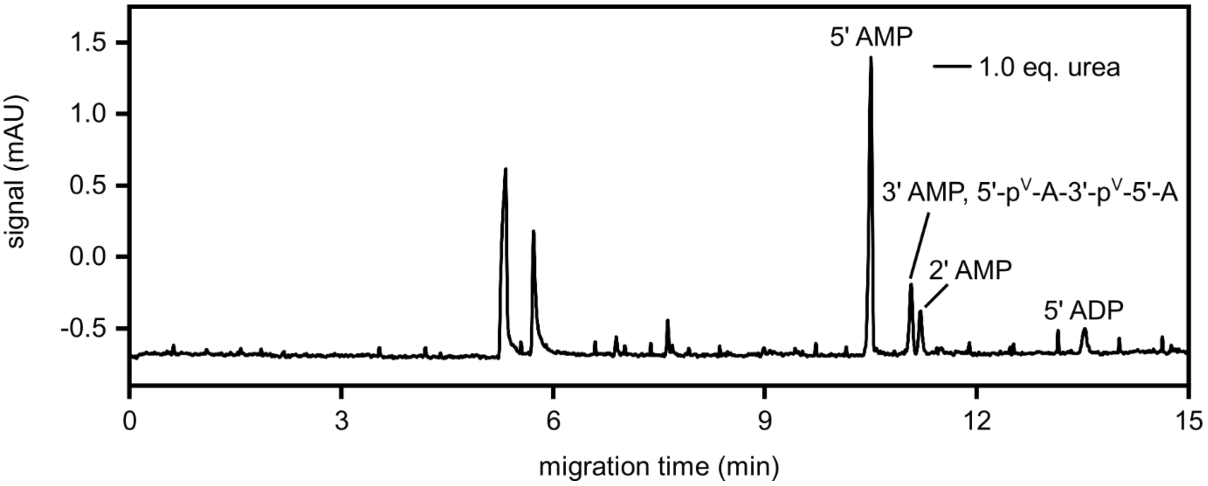

Supplementary Figure 26. First analysis run of the reaction starting from A (100 mM, 1.0 eq.), H_3PO_3 (3.0 eq.) and urea (1.0 eq.) after 7 d. The sample was diluted to 200 µM referring to the initial A concentration for determination of the 5’ AMP yield. Conditions of the electrophoretic separation: BFS capillary (l = 80 cm, length to detector: 71.5 cm); BGE: NH_4FA (30 mM, pH 9.5); CE inlet: 30 kV, pressure driven sample injection: 30 mbar for 10 s, detection at 254 nm.

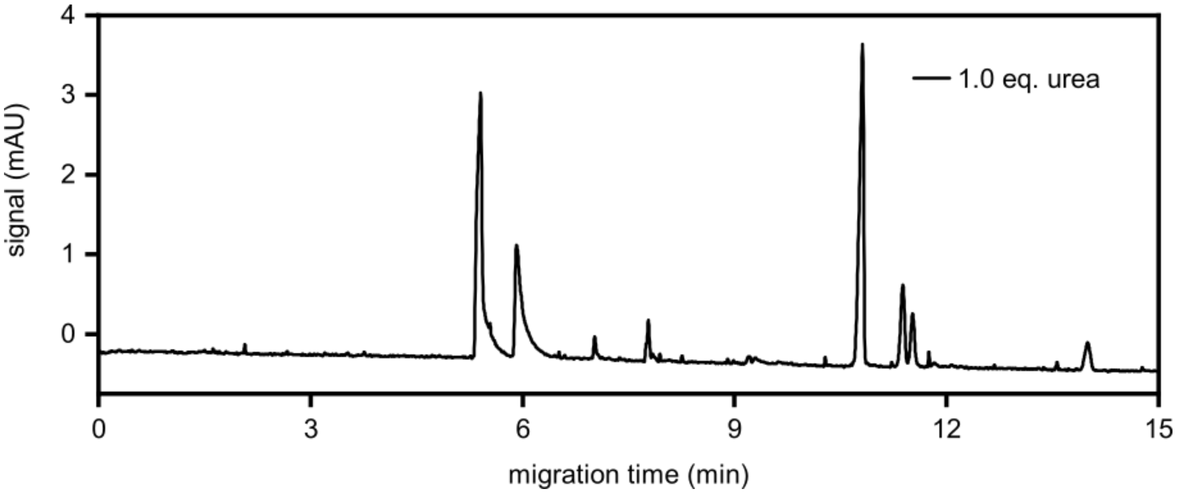

Supplementary Figure 27. First analysis run of the reaction starting from A (100 mM, 1.0 eq.), H_3PO_3 (3.0 eq.) and urea (1.0 eq.) after 7 d. The sample was diluted to 500 µM referring to the initial A concentration for determination of the 5’ ADP yield. Conditions of the electrophoretic separation: BFS capillary (l = 80 cm, length to detector: 71.5 cm); BGE: NH_4FA (30 mM, pH 9.5); CE inlet: 30 kV, pressure driven sample injection: 30 mbar for 10 s, detection at 254 nm.

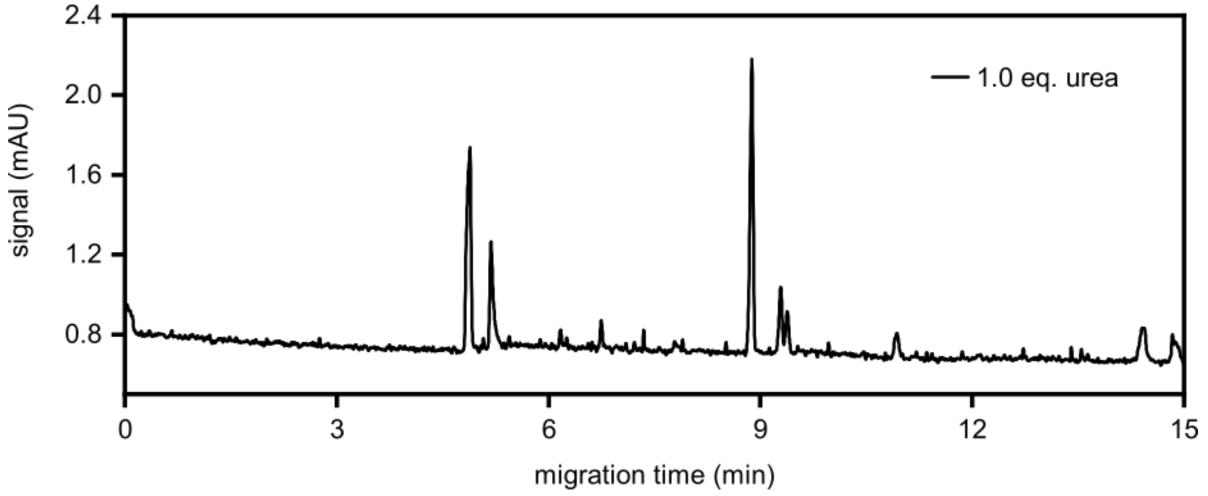

Supplementary Figure 28. Second analysis run of the reaction starting from A (100 mM, 1.0 eq.), H_3PO_3 (3.0 eq.) and urea (1.0 eq.) after 7 d. The sample was diluted to 200 µM referring to the initial A concentration for determination of the 5' AMP yield. Conditions of the electrophoretic separation: BFS capillary (l = 80 cm, length to detector: 71.5 cm); BGE: NH_4FA (30 mM, pH 9.5); CE inlet: 30 kV, pressure driven sample injection: 30 mbar for 10 s, detection at 254 nm.

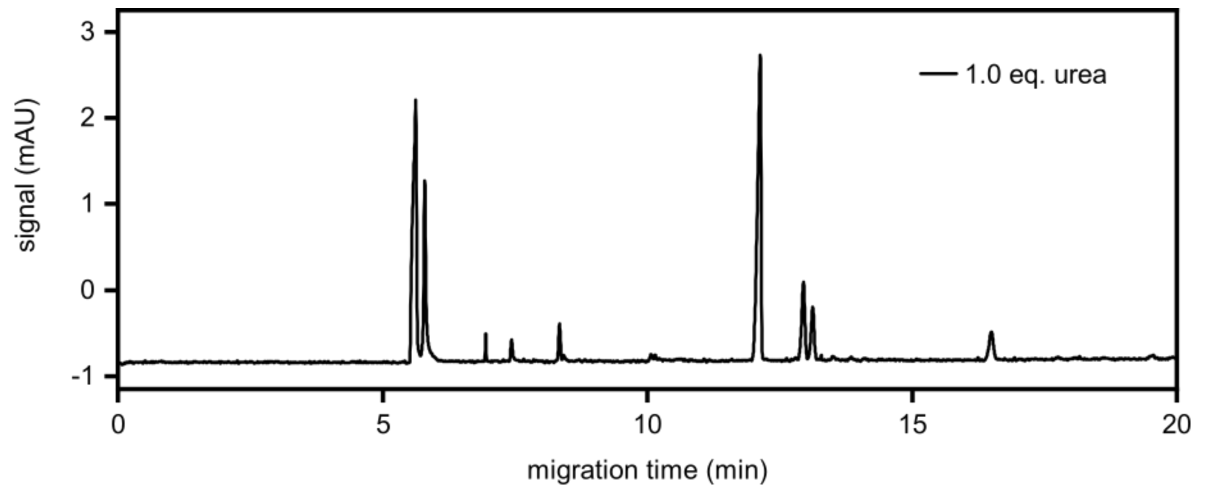

Supplementary Figure 29. Second analysis run of the reaction starting from A (100 mM, 1.0 eq.), H_3PO_3 (3.0 eq.) and urea (1.0 eq.) after 7 d. The sample was diluted to 500 µM referring to the initial A concentration for determination of the 5' ADP yield. Conditions of the electrophoretic separation: BFS capillary (l = 80 cm, length to detector: 71.5 cm); BGE: NH_4FA (30 mM, pH 9.5); CE inlet: 30 kV, pressure driven sample injection: 30 mbar for 10 s, detection at 254 nm.

Supplementary Table 7. First analysis runs of the reaction starting from A (100 mM, 1.0 eq.), H_3PO_3 (3.0 eq.) and urea (1.0 eq.) after 7 d. The sample was diluted to 200 µM referring to the initial A concentration for determination of the 5' AMP yield and to 500 µM for determination of the 5' ADP yield.

**Product**	**Integral** (mAU x min)	$c_{sample\ vial}$ (µM)	$c_{reaction\ mixture}$ (mM)	**Yield** (%)
5' AMP	0.1095	62.91	31.45	31.45
5' ADP	0.0316	11.38	2.28	2.28

Supplementary Table 8. Second analysis runs of the reaction starting from A (100 mM, 1.0 eq.), H_3PO_3 (3.0 eq.) and urea (1.0 eq.) after 7 d. The sample was diluted to 200 µM referring to the initial A concentration for determination of the 5' AMP yield and to 500 µM for determination of the 5' ADP yield.

**Product**	**Integral** (mAU x min)	$c_{sample\ vial}$ (µM)	$c_{reaction\ mixture}$ (mM)	**Yield** (%)	**Ø Yield** (%)
5' AMP	0.0764	43.90	21.95	21.95	26.70 ± 6.72
5' ADP	0.0303	10.90	2.18	2.18	2.23 ± 0.07

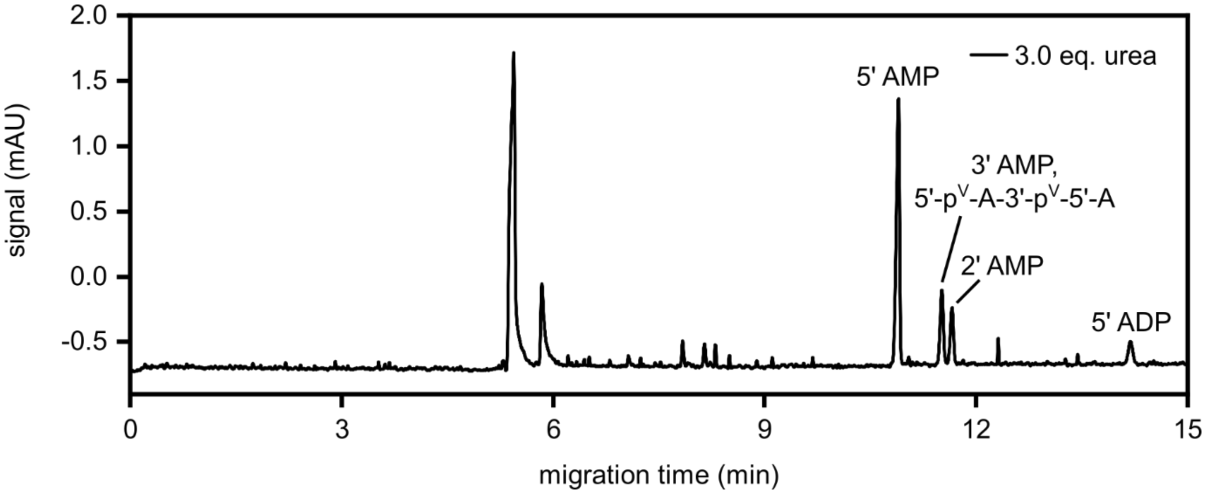

Supplementary Figure 30. First analysis run of the reaction starting from A (100 mM, 1.0 eq.), H_3PO_3 (3.0 eq.) and urea (3.0 eq.) after 7 d. The sample was diluted to 250 µM referring to the initial A concentration for determination of the 5’ AMP yield. Conditions of the electrophoretic separation: BFS capillary (l = 80 cm, length to detector: 71.5 cm); BGE: NH_4FA (30 mM, pH 9.5); CE inlet: 30 kV, pressure driven sample injection: 30 mbar for 10 s, detection at 254 nm.

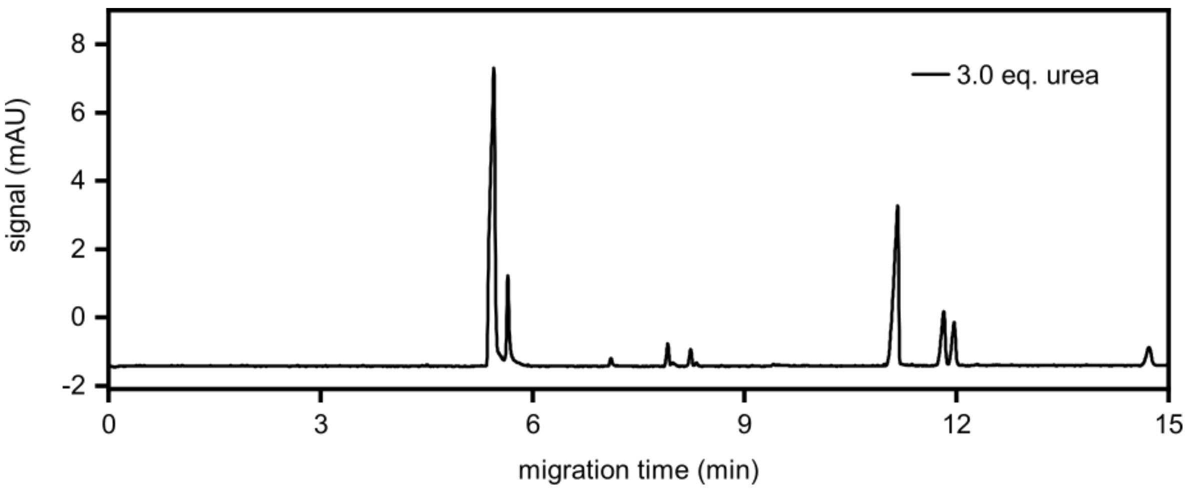

Supplementary Figure 31. First analysis run of the reaction starting from A (100 mM, 1.0 eq.), H_3PO_3 (3.0 eq.) and urea (3.0 eq.) after 7 d. The sample was diluted to 1 mM referring to the initial A concentration for determination of the 5’ ADP yield. Conditions of the electrophoretic separation: BFS capillary (l = 80 cm, length to detector: 71.5 cm); BGE: NH_4FA (30 mM, pH 9.5); CE inlet: 30 kV, pressure driven sample injection: 30 mbar for 10 s, detection at 254 nm.

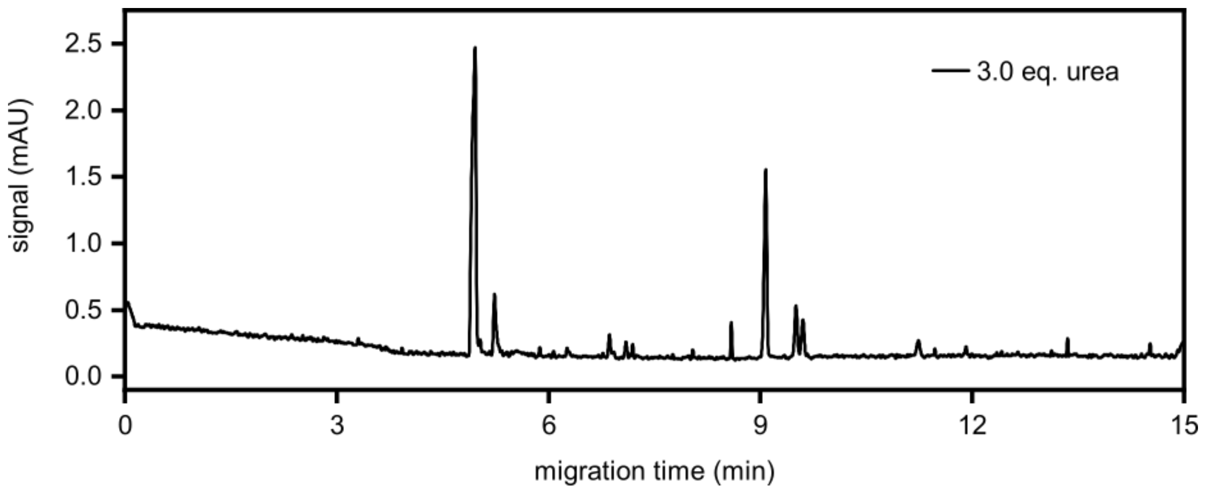

Supplementary Figure 32. Second analysis run of the reaction starting from A (100 mM, 1.0 eq.), H_3PO_3 (3.0 eq.) and urea (3.0 eq.) after 7 d. The sample was diluted to 250 µM referring to the initial A concentration for determination of the 5’ AMP yield. Conditions of the electrophoretic separation: BFS capillary (l = 80 cm, length to detector: 71.5 cm); BGE: NH_4FA (30 mM, pH 9.5); CE inlet: 30 kV, pressure driven sample injection: 30 mbar for 10 s, detection at 254 nm.

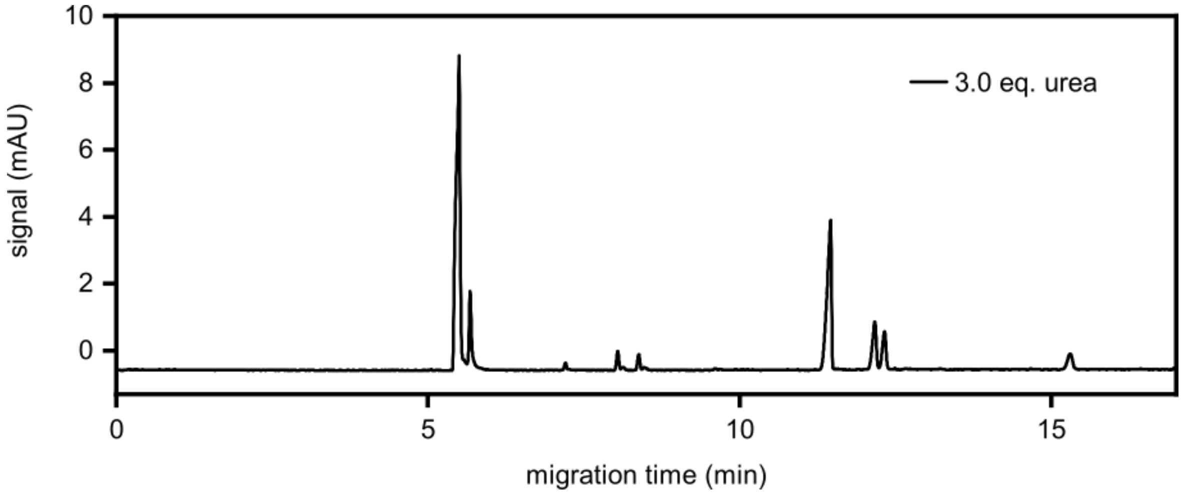

Supplementary Figure 33. Second analysis run of the reaction starting from A (100 mM, 1.0 eq.), H_3PO_3 (3.0 eq.) and urea (3.0 eq.) after 7 d. The sample was diluted to 1 mM referring to the initial A concentration for determination of the 5’ ADP yield. Conditions of the electrophoretic separation: BFS capillary (l = 80 cm, length to detector: 71.5 cm); BGE: NH_4FA (30 mM, pH 9.5); CE inlet: 30 kV, pressure driven sample injection: 30 mbar for 10 s, detection at 254 nm.

Supplementary Table 9. First analysis runs of the reaction starting from A (100 mM, 1.0 eq.), H_3PO_3 (3.0 eq.) and urea (3.0 eq.) after 7 d. The sample was diluted to 250 µM referring to the initial A concentration for determination of the 5' AMP yield and to 1 mM for determination of the 5' ADP yield.

**Product**	**Integral** (mAU x min)	$c_{sample\ vial}$ (µM)	$c_{reaction\ mixture}$ (mM)	**Yield** (%)
5' AMP	0.1093	62.84	25.14	25.14
5' ADP	0.0463	16.64	1.66	1.66

Supplementary Table 10. Second analysis runs of the reaction starting from A (100 mM, 1.0 eq.), H_3PO_3 (3.0 eq.) and urea (3.0 eq.) after 7 d. The sample was diluted to 250 µM referring to the initial A concentration for determination of the 5' AMP yield and to 1 mM for determination of the 5' ADP yield.

**Product**	**Integral** (mAU x min)	$c_{sample\ vial}$ (µM)	$c_{reaction\ mixture}$ (mM)	**Yield** (%)	**Ø Yield** (%)
5' AMP	0.0630	36.18	14.47	14.47	19.80 ± 7.54
5' ADP	0.0424	15.26	1.53	1.53	1.59 ± 0.10

Variation of the adenosine concentration

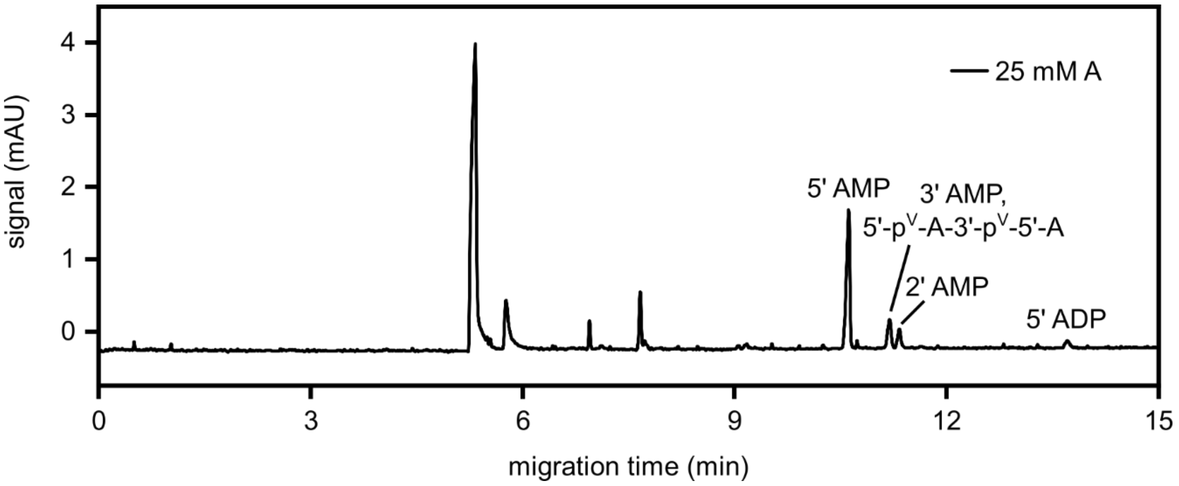

Supplementary Figure 34. First analysis run of the reaction starting from A (25 mM, 1.0 eq.) and H_3PO_3 (1.0 eq.) after 7 d. The sample was diluted to 500 µM referring to the initial A concentration for determination of the 5’ AMP yield. Conditions of the electrophoretic separation: BFS capillary (l = 80 cm, length to detector: 71.5 cm); BGE: NH_4FA (30 mM, pH 9.5); CE inlet: 30 kV, pressure driven sample injection: 30 mbar for 10 s, detection at 254 nm.

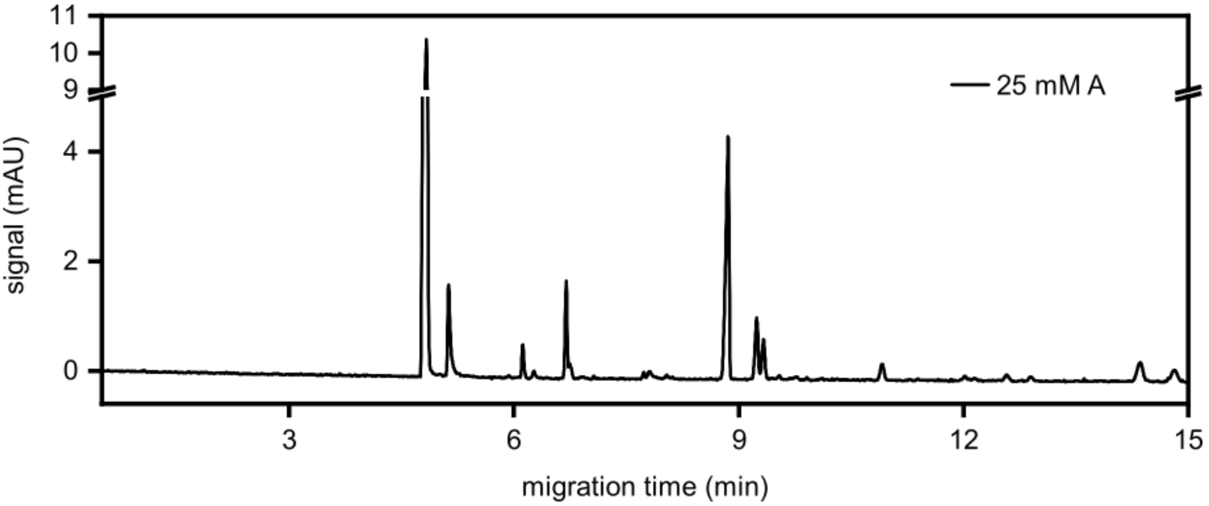

Supplementary Figure 35. First analysis run of the reaction starting from A (25 mM, 1.0 eq.) and H_3PO_3 (1.0 eq.) after 7 d. The sample was diluted to 1 mM referring to the initial A concentration for determination of the 5’ ADP yield. Conditions of the electrophoretic separation: BFS capillary (l = 80 cm, length to detector: 71.5 cm); BGE: NH_4FA (30 mM, pH 9.5); CE inlet: 30 kV, pressure driven sample injection: 30 mbar for 10 s, detection at 254 nm.

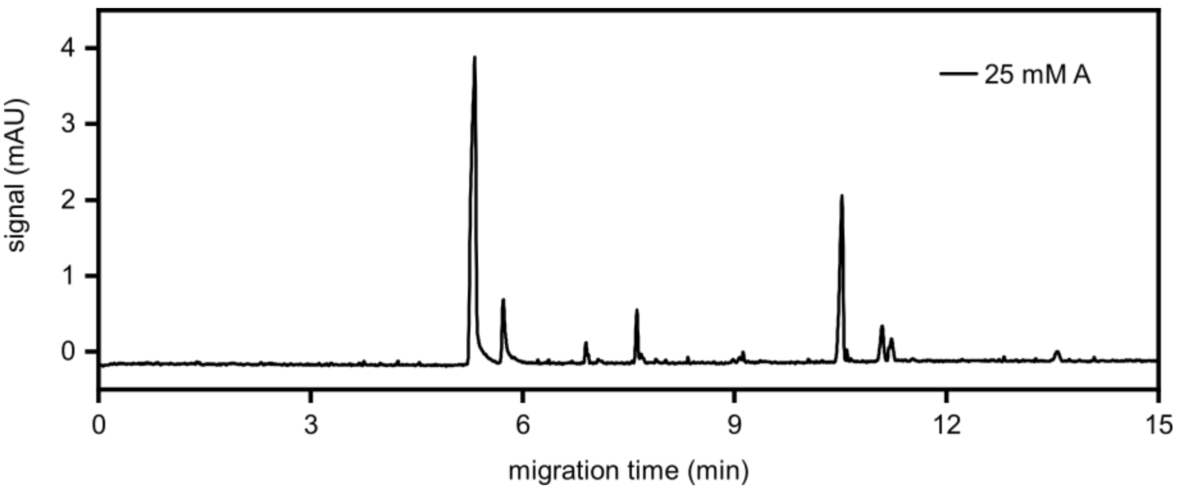

Supplementary Figure 36. Second analysis run of the reaction starting from A (25 mM, 1.0 eq.) and H_3PO_3 (1.0 eq.) after 7 d. The sample was diluted to 500 µM referring to the initial A concentration for determination of the 5’ AMP yield. Conditions of the electrophoretic separation: BFS capillary (l = 80 cm, length to detector: 71.5 cm); BGE: NH_4FA (30 mM, pH 9.5); CE inlet: 30 kV, pressure driven sample injection: 30 mbar for 10 s, detection at 254 nm.

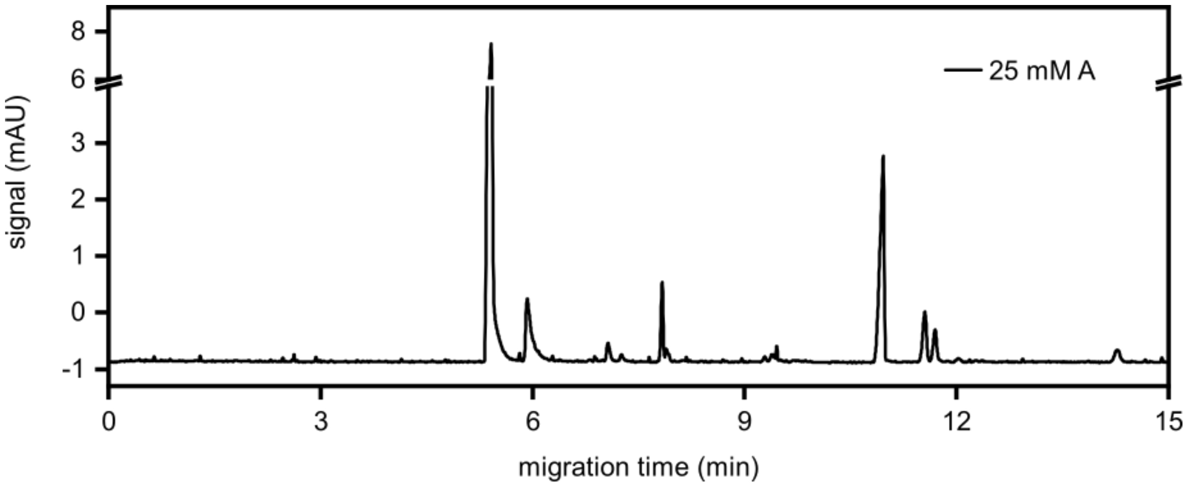

Supplementary Figure 37. Second analysis run of the reaction starting from A (25 mM, 1.0 eq.) and H_3PO_3 (1.0 eq.) after 7 d. The sample was diluted to 1 mM referring to the initial A concentration for determination of the 5’ ADP yield. Conditions of the electrophoretic separation: BFS capillary (l = 80 cm, length to detector: 71.5 cm); BGE: NH_4FA (30 mM, pH 9.5); CE inlet: 30 kV, pressure driven sample injection: 30 mbar for 10 s, detection at 254 nm.

Supplementary Table 11. First analysis runs of the reaction starting from A (25 mM, 1.0 eq.) and H_3PO_3 (1.0 eq.) after 7 d. The sample was diluted to 500 µM referring to the initial A concentration for determination of the 5’ AMP yield and to 1 mM for the determination of the 5’ ADP yield.

**Product**	**Integral** (mAU x min)	$c_{sample\ vial}$ (µM)	$c_{reaction\ mixture}$ (mM)	**Yield** (%)
5’ AMP	0.1090	62.66	3.13	12.53
5’ ADP	0.0174	6.26	0.16	0.63

Supplementary Table 12. Second analysis runs of the reaction starting from A (25 mM, 1.0 eq.) and H_3PO_3 (1.0 eq.) after 7 d. The sample was diluted to 500 µM referring to the initial A concentration for determination of the 5’ AMP yield and to 1 mM for the determination of the 5’ ADP yield.

**Product**	**Integral** (mAU x min)	$c_{sample\ vial}$ (µM)	$c_{reaction\ mixture}$ (mM)	**Yield** (%)	**Ø Yield** (%)
5’ AMP	0.1240	71.24	3.56	14.25	13.39 ± 1.21
5’ ADP	0.0201	7.25	0.18	0.72	0.68 ± 0.07

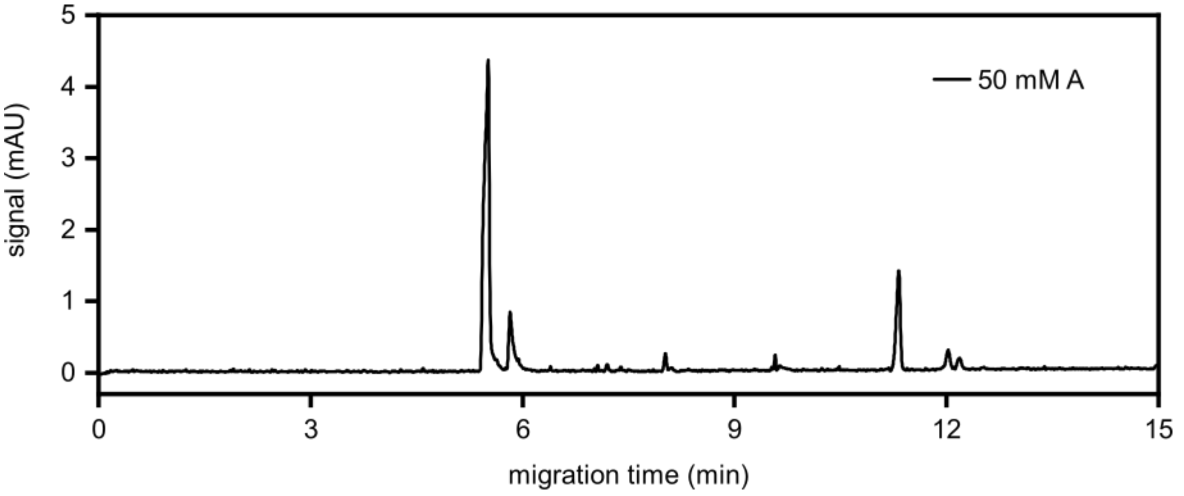

Supplementary Figure 38. First analysis run of the reaction starting from A (50 mM, 1.0 eq.) and H_3PO_3 (1.0 eq.) after 7 d. The sample was diluted to 500 µM referring to the initial A concentration for determination of the 5' AMP yield. Conditions of the electrophoretic separation: BFS capillary (l = 80 cm, length to detector: 71.5 cm); BGE: NH_4FA (30 mM, pH 9.5); CE inlet: 30 kV, pressure driven sample injection: 30 mbar for 10 s, detection at 254 nm.

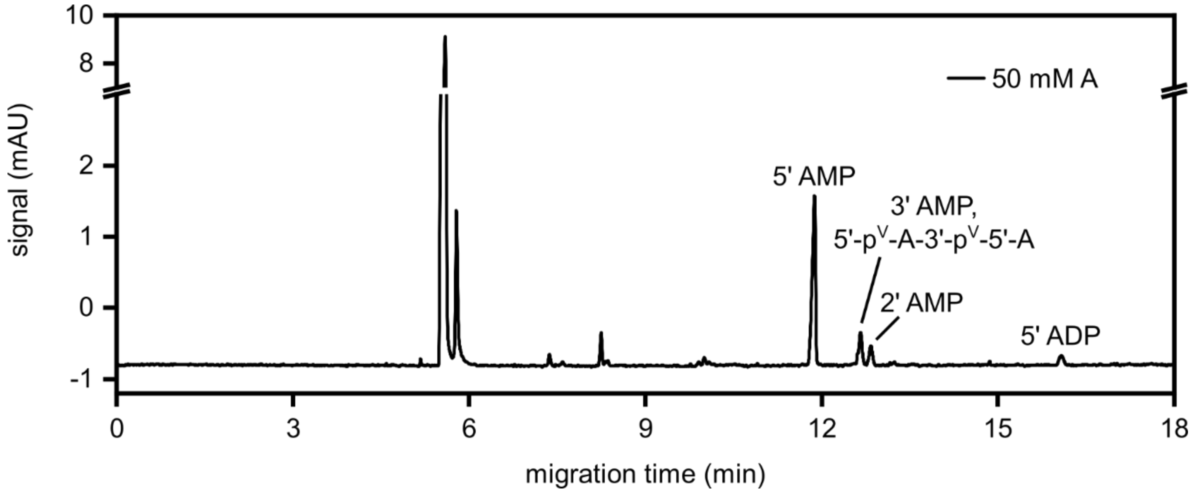

Supplementary Figure 39. First analysis run of the reaction starting from A (50 mM, 1.0 eq.) and H_3PO_3 (1.0 eq.) after 7 d. The sample was diluted to 1 mM referring to the initial A concentration for determination of the 5' ADP yield. Conditions of the electrophoretic separation: BFS capillary (l = 80 cm, length to detector: 71.5 cm); BGE: NH_4FA (30 mM, pH 9.5); CE inlet: 30 kV, pressure driven sample injection: 30 mbar for 10 s, detection at 254 nm.

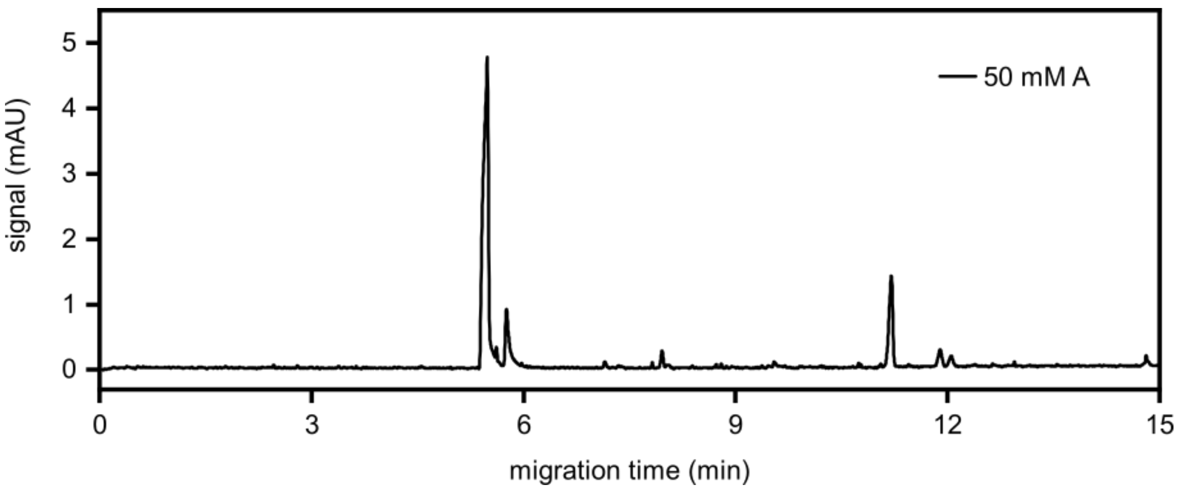

Supplementary Figure 40. Second analysis run of the reaction starting from A (50 mM, 1.0 eq.) and H_3PO_3 (1.0 eq.) after 7 d. The sample was diluted to 500 µM referring to the initial A concentration for determination of the 5’ AMP yield. Conditions of the electrophoretic separation: BFS capillary (l = 80 cm, length to detector: 71.5 cm); BGE: NH_4FA (30 mM, pH 9.5); CE inlet: 30 kV, pressure driven sample injection: 30 mbar for 10 s, detection at 254 nm.

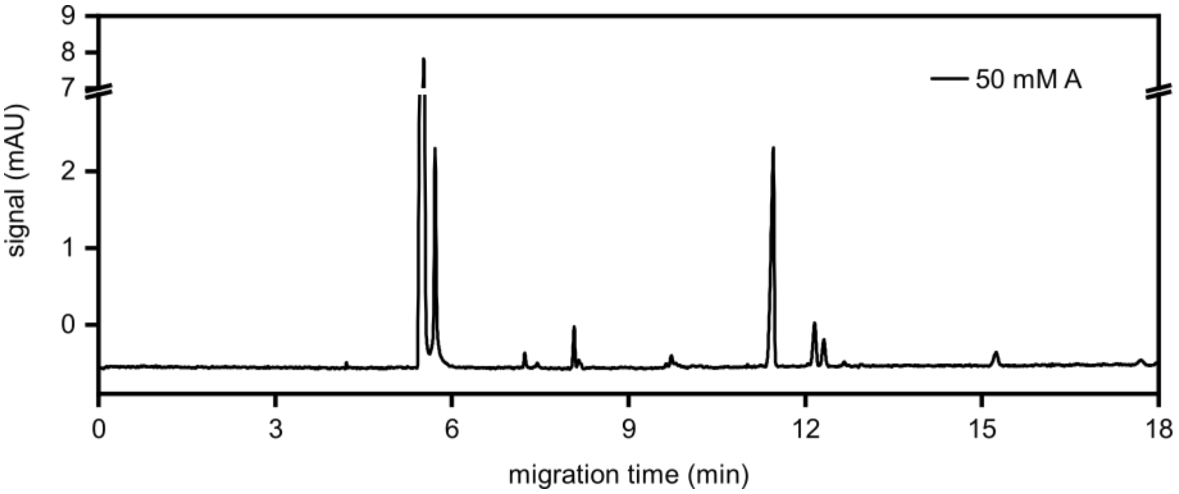

Supplementary Figure 41. Second analysis run of the reaction starting from A (50 mM, 1.0 eq.) and H_3PO_3 (1.0 eq.) after 7 d. The sample was diluted to 1 mM referring to the initial A concentration for determination of the 5’ ADP yield. Conditions of the electrophoretic separation: BFS capillary (l = 80 cm, length to detector: 71.5 cm); BGE: NH_4FA (30 mM, pH 9.5); CE inlet: 30 kV, pressure driven sample injection: 30 mbar for 10 s, detection at 254 nm.

Supplementary Table 13. First analysis runs of the reaction starting from A (50 mM, 1.0 eq.) and H_3PO_3 (1.0 eq.) after 7 d. The sample was diluted to 500 µM referring to the initial A concentration for determination of the 5′ AMP yield and to 1 mM for determination of the 5′ ADP yield.

**Product**	**Integral** (mAU x min)	$c_{sample\ vial}$ (µM)	$c_{reaction\ mixture}$ (mM)	**Yield** (%)
5′ AMP	0.0936	53.80	5.38	10.76
5′ ADP	0.0127	4.58	0.23	0.46

Supplementary Table 14. Second analysis runs of the reaction starting from A (50 mM, 1.0 eq.) and H_3PO_3 (1.0 eq.) after 7 d. The sample was diluted to 500 µM referring to the initial A concentration for determination of the 5′ AMP yield and to 1 mM for determination of the 5′ ADP yield.

**Product**	**Integral** (mAU x min)	$c_{sample\ vial}$ (µM)	$c_{reaction\ mixture}$ (mM)	**Yield** (%)	**Ø Yield** (%)
5′ AMP	0.0855	49.13	4.91	9.83	10.29 ± 0.66
5′ ADP	0.0143	5.16	0.26	0.52	0.49 ± 0.04

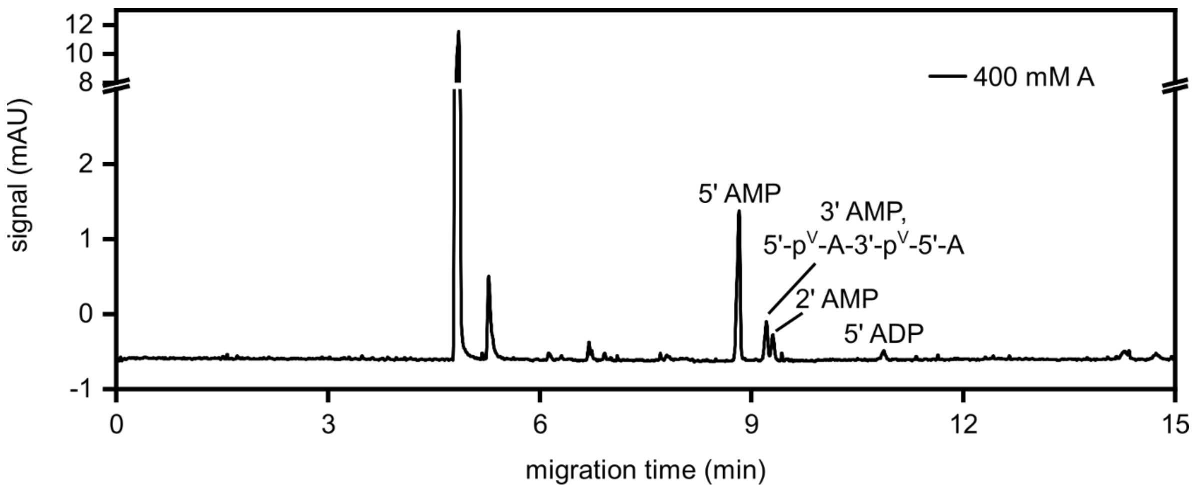

Supplementary Figure 42. First analysis run of the reaction starting from A (400 mM, 1.0 eq.) and H_3PO_3 (1.0 eq.) after 7 d. The sample was diluted to 1 mM referring to the initial A concentration for determination of the 5' AMP yield. Conditions of the electrophoretic separation: BFS capillary (l = 80 cm, length to detector: 71.5 cm); BGE: NH_4FA (30 mM, pH 9.5); CE inlet: 30 kV, pressure driven sample injection: 30 mbar for 10 s, detection at 254 nm.

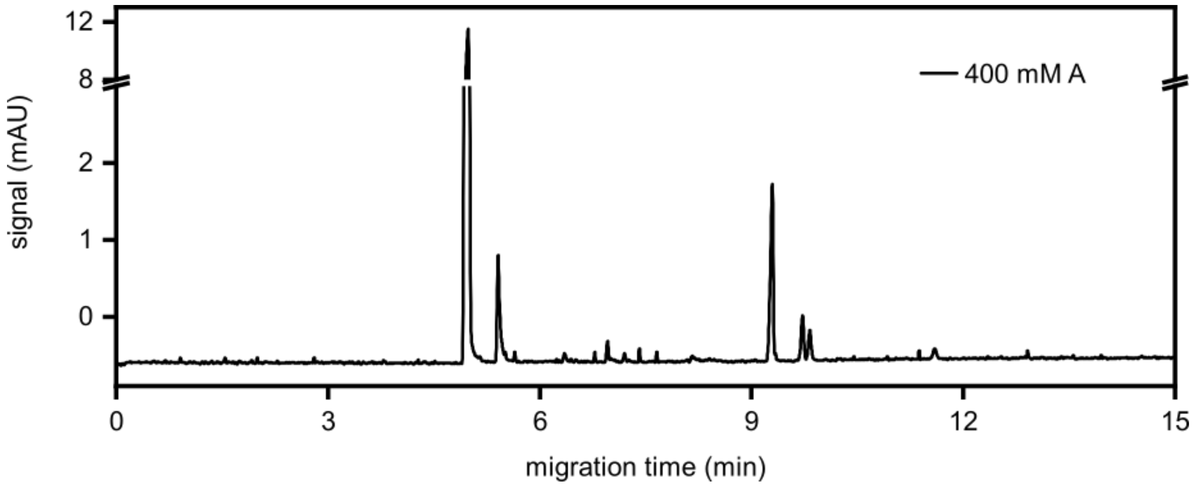

Supplementary Figure 43. Second analysis run of the reaction starting from A (400 mM, 1.0 eq.) and H_3PO_3 (1.0 eq.) after 7 d. The sample was diluted to 1 mM referring to the initial A concentration for determination of the 5' AMP yield. Conditions of the electrophoretic separation: BFS capillary (l = 80 cm, length to detector: 71.5 cm); BGE: NH_4FA (30 mM, pH 9.5); CE inlet: 30 kV, pressure driven sample injection: 30 mbar for 10 s, detection at 254 nm.

Supplementary Table 15. First analysis run of the reaction starting from A (400 mM, 1.0 eq.) and H_3PO_3 (1.0 eq.) after 7 d. The sample was diluted to 1 mM referring to the initial A concentration for determination of the 5' AMP yield.

**Product**	**Integral** (mAU x min)	$c_{sample\ vial}$ (µM)	$c_{reaction\ mixture}$ (mM)	**Yield** (%)
5' AMP	0.1043	59.94	23.97	5.99

Supplementary Table 16. Second analysis run of the reaction starting from A (400 mM, 1.0 eq.) and H_3PO_3 (1.0 eq.) after 7 d. The sample was diluted to 1 mM referring to the initial A concentration for determination of the 5' AMP yield.

**Product**	**Integral** (mAU x min)	$c_{sample\ vial}$ (µM)	$c_{reaction\ mixture}$ (mM)	**Yield** (%)	**Ø Yield** (%)
5' AMP	0.1047	60.17	24.07	6.02	6.01 ± 0.02

Time dependence of the reaction

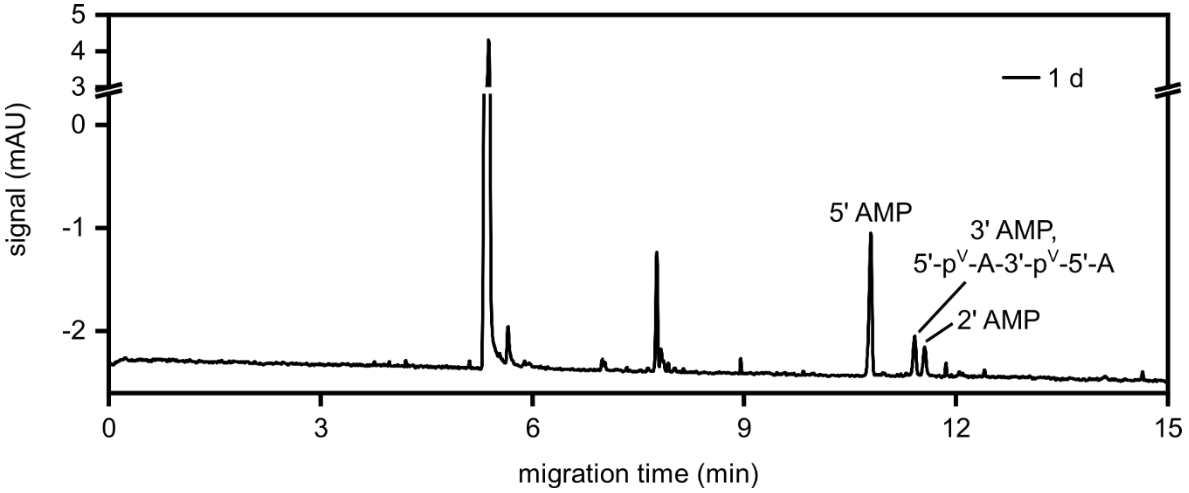

Supplementary Figure 44. First analysis run of the reaction starting from A (100 mM, 1.0 eq.), H_3PO_3 (3.0 eq.) and urea (1.0 eq.) after 1 d. The sample was diluted to 500 µM referring to the initial A concentration for determination of the 5' AMP yield. Conditions of the electrophoretic separation: BFS capillary (l = 80 cm, length to detector: 71.5 cm); BGE: NH_4FA (30 mM, pH 9.5); CE inlet: 30 kV, pressure driven sample injection: 30 mbar for 10 s, detection at 254 nm.

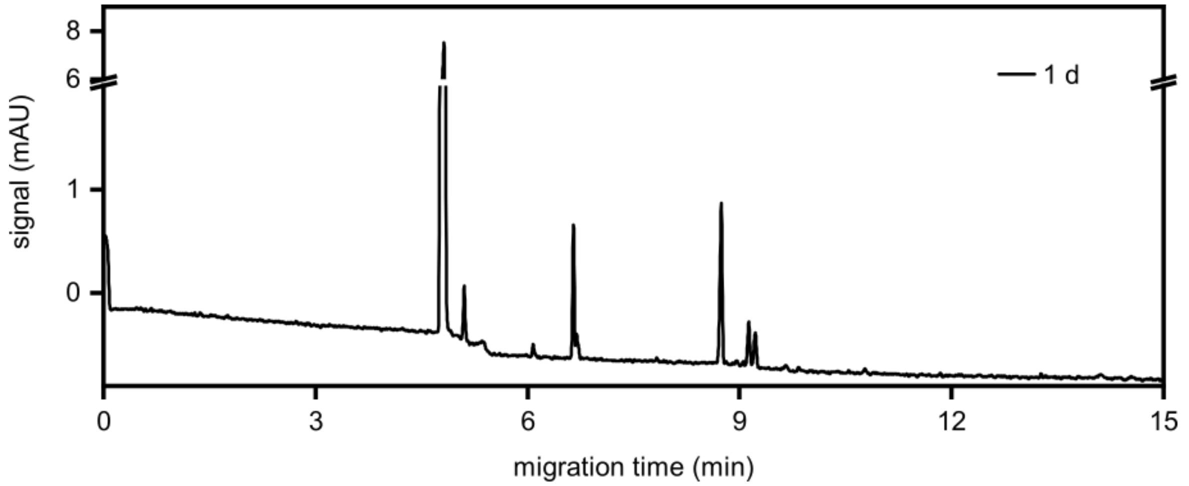

Supplementary Figure 45. Second analysis run of the reaction starting from A (100 mM, 1.0 eq.), H_3PO_3 (3.0 eq.) and urea (1.0 eq.) after 1 d. The sample was diluted to 500 µM referring to the initial A concentration for determination of the 5' AMP yield. Conditions of the electrophoretic separation: BFS capillary (l = 80 cm, length to detector: 71.5 cm); BGE: NH_4FA (30 mM, pH 9.5); CE inlet: 30 kV, pressure driven sample injection: 30 mbar for 10 s, detection at 254 nm.

Supplementary Table 17. First analysis run of the reaction starting from A (100 mM, 1.0 eq.), H_3PO_3 (3.0 eq.) and urea (1.0 eq.) after 1 d. The sample was diluted to 500 µM referring to the initial A concentration for determination of the 5' AMP yield.

**Product**	**Integral** (mAU x min)	$c_{sample\ vial}$ (µM)	$c_{reaction\ mixture}$ (mM)	**Yield** (%)
5' AMP	0.0659	37.86	7.57	7.57

Supplementary Table 18. Second analysis run of the reaction starting from A (100 mM, 1.0 eq.), H_3PO_3 (3.0 eq.) and urea (1.0 eq.) after 1 d. The sample was diluted to 500 µM referring to the initial A concentration for determination of the 5' AMP yield.

**Product**	**Integral** (mAU x min)	$c_{sample\ vial}$ (µM)	$c_{reaction\ mixture}$ (mM)	**Yield** (%)	**Ø Yield** (%)
5' AMP	0.0604	34.74	6.95	6.95	7.26 ± 0.44

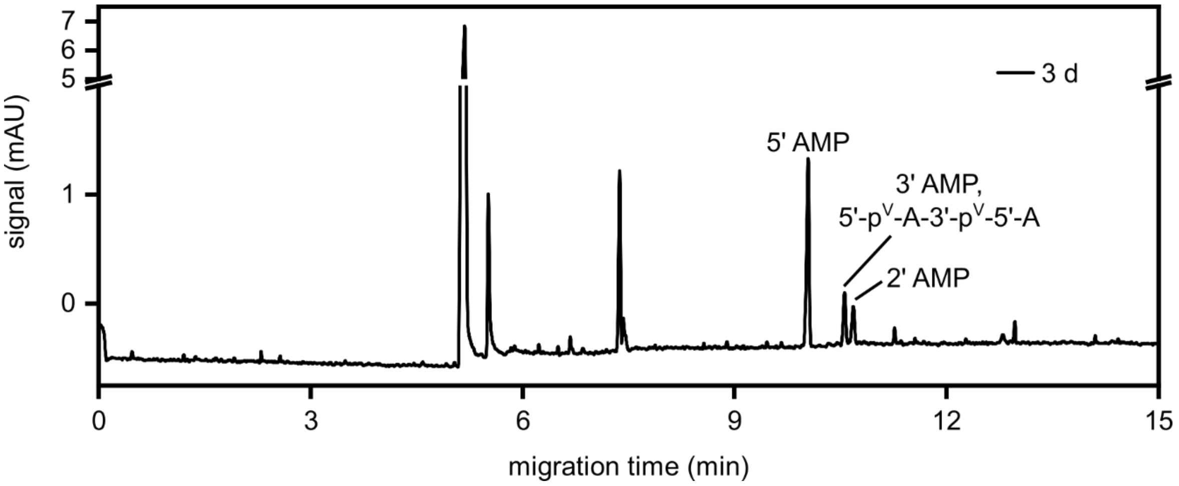

Supplementary Figure 46. First analysis run of the reaction starting from A (100 mM, 1.0 eq.), H_3PO_3 (3.0 eq.) and urea (1.0 eq.) after 3 d. The sample was diluted to 500 µM referring to the initial A concentration for determination of the 5’ AMP yield. Conditions of the electrophoretic separation: BFS capillary (l = 80 cm, length to detector: 71.5 cm); BGE: NH_4FA (30 mM, pH 9.5); CE inlet: 30 kV, pressure driven sample injection: 30 mbar for 10 s, detection at 254 nm.

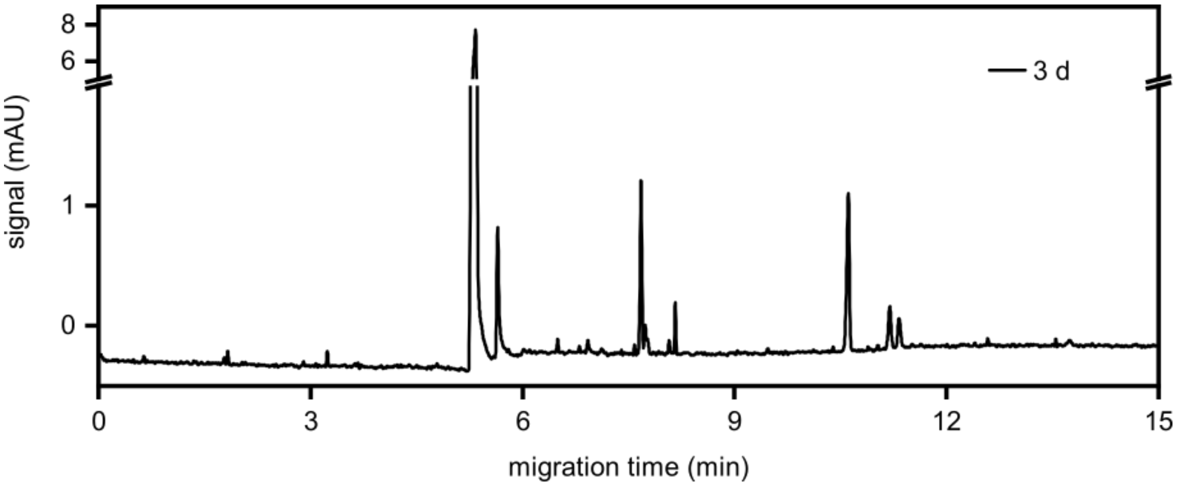

Supplementary Figure 47. Second analysis run of the reaction starting from A (100 mM, 1.0 eq.), H_3PO_3 (3.0 eq.) and urea (1.0 eq.) after 3 d. The sample was diluted to 500 µM referring to the initial A concentration for determination of the 5’ AMP yield. Conditions of the electrophoretic separation: BFS capillary (l = 80 cm, length to detector: 71.5 cm); BGE: NH_4FA (30 mM, pH 9.5); CE inlet: 30 kV, pressure driven sample injection: 30 mbar for 10 s, detection at 254 nm.

Supplementary Table 19. First analysis run of the reaction starting from A (100 mM, 1.0 eq.), H_3PO_3 (3.0 eq.) and urea (1.0 eq.) after 3 d. The sample was diluted to 500 µM referring to the initial A concentration for determination of the 5' AMP yield.

**Product**	**Integral** (mAU x min)	$c_{sample\ vial}$ (µM)	$c_{reaction\ mixture}$ (mM)	**Yield** (%)
5' AMP	0.0585	33.59	6.72	6.72

Supplementary Table 20. Second analysis run of the reaction starting from A (100 mM, 1.0 eq.), H_3PO_3 (3.0 eq.) and urea (1.0 eq.) after 3 d. The sample was diluted to 500 µM referring to the initial A concentration for determination of the 5' AMP yield.

**Product**	**Integral** (mAU x min)	$c_{sample\ vial}$ (µM)	$c_{reaction\ mixture}$ (mM)	**Yield** (%)	**Ø Yield** (%)
5' AMP	0.0736	42.30	8.46	8.46	7.59 ± 1.23

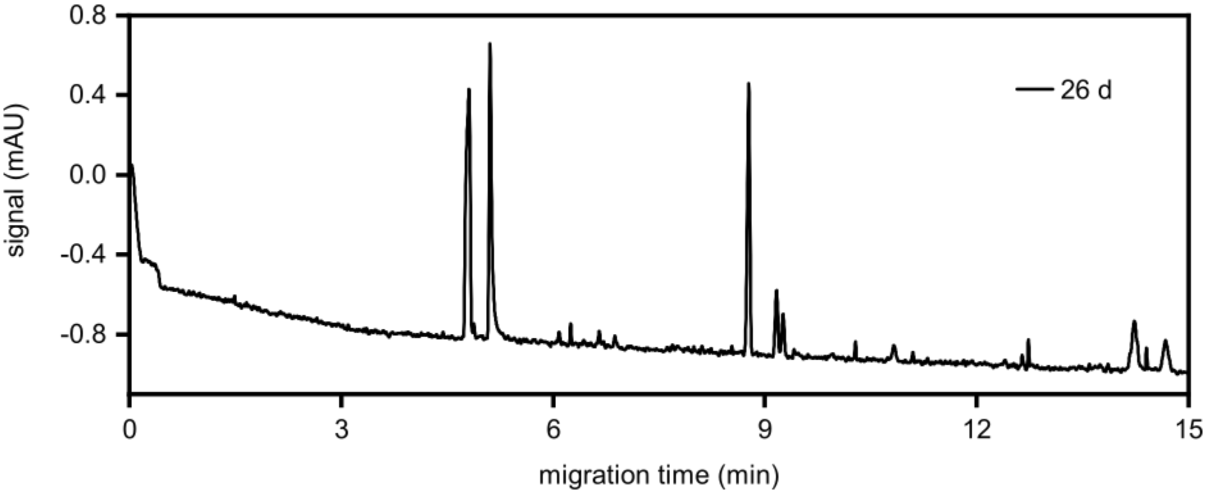

Supplementary Figure 48. First analysis run of the reaction starting from A (100 mM, 1.0 eq.), H_3PO_3 (3.0 eq.) and urea (1.0 eq.) after 26 d. The sample was diluted to 200 µM referring to the initial A concentration for determination of the 5’ AMP yield. Conditions of the electrophoretic separation: BFS capillary (l = 80 cm, length to detector: 71.5 cm); BGE: NH_4FA (30 mM, pH 9.5); CE inlet: 30 kV, pressure driven sample injection: 30 mbar for 10 s, detection at 254 nm.

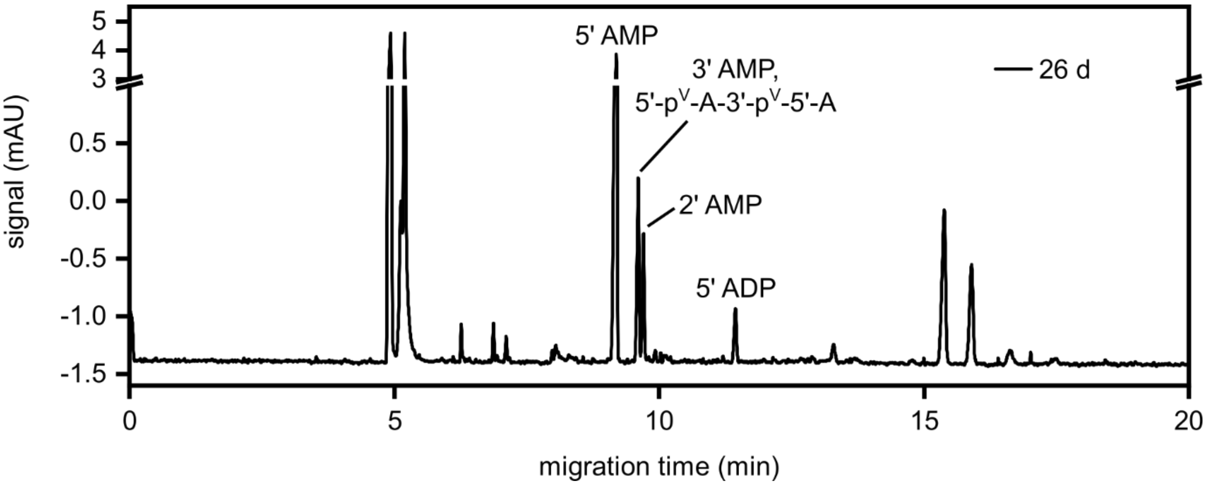

Supplementary Figure 49. First analysis run of the reaction starting from A (100 mM, 1.0 eq.), H_3PO_3 (3.0 eq.) and urea (1.0 eq.) after 26 d. The sample was diluted to 1 mM referring to the initial A concentration for determination of the 5’ ADP yield. Conditions of the electrophoretic separation: BFS capillary (l = 80 cm, length to detector: 71.5 cm); BGE: NH_4FA (30 mM, pH 9.5); CE inlet: 30 kV, pressure driven sample injection: 30 mbar for 10 s, detection at 254 nm.

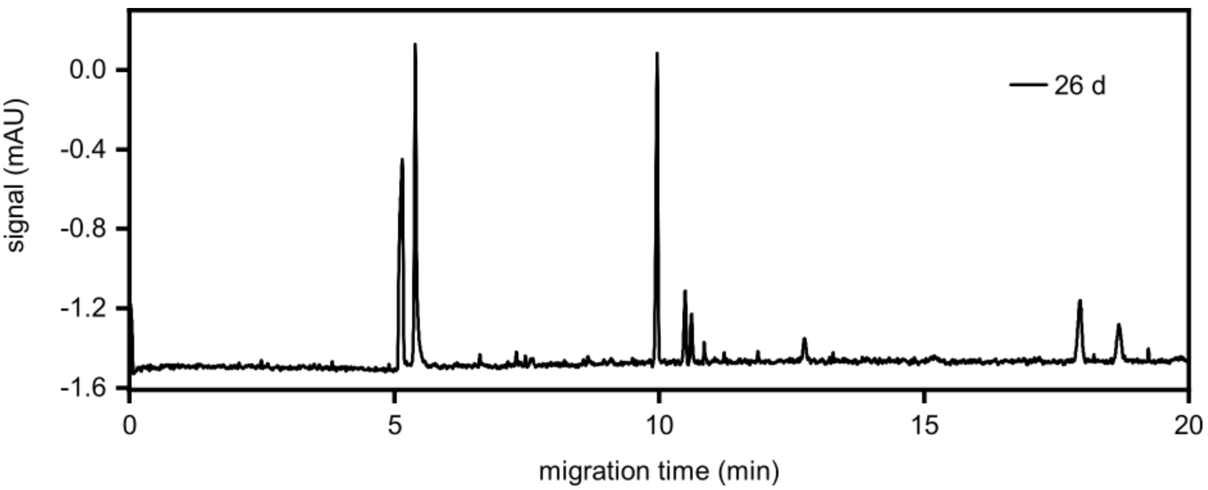

Supplementary Figure 50. Second analysis run of the reaction starting from A (100 mM, 1.0 eq.), H_3PO_3 (3.0 eq.) and urea (1.0 eq.) after 26 d. The sample was diluted to 200 µM referring to the initial A concentration for determination of the 5' AMP yield. Conditions of the electrophoretic separation: BFS capillary (l = 80 cm, length to detector: 71.5 cm); BGE: NH_4FA (30 mM, pH 9.5); CE inlet: 30 kV, pressure driven sample injection: 30 mbar for 10 s, detection at 254 nm.

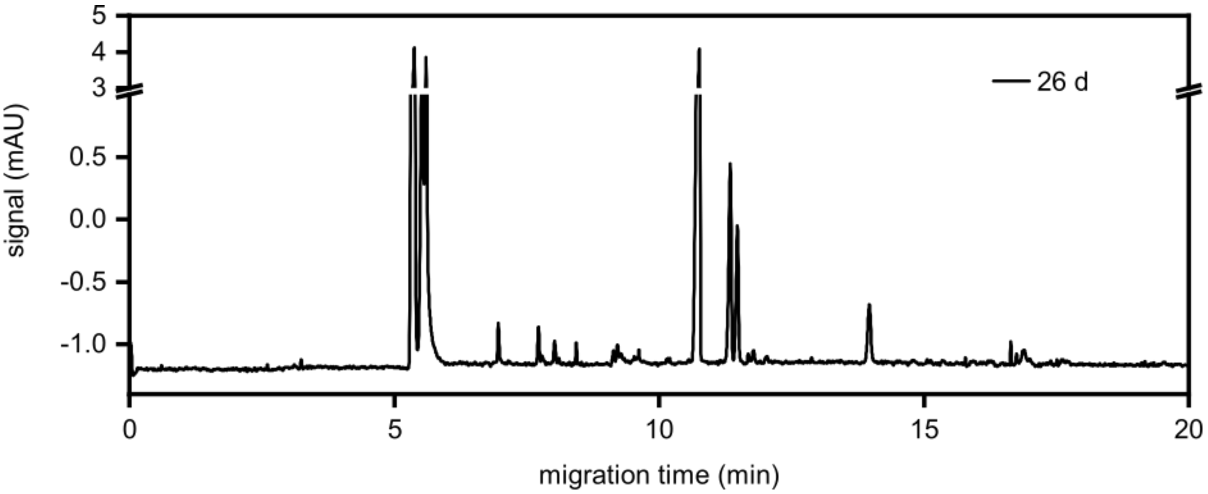

Supplementary Figure 51. Second analysis run of the reaction starting from A (100 mM, 1.0 eq.), H_3PO_3 (3.0 eq.) and urea (1.0 eq.) after 26 d. The sample was diluted to 1 mM referring to the initial A concentration for determination of the 5' ADP yield. Conditions of the electrophoretic separation: BFS capillary (l = 80 cm, length to detector: 71.5 cm); BGE: NH_4FA (30 mM, pH 9.5); CE inlet: 30 kV, pressure driven sample injection: 30 mbar for 10 s, detection at 254 nm.

Supplementary Table 21. First analysis runs of the reaction starting from A (100 mM, 1.0 eq.), H_3PO_3 (3.0 eq.) and urea (1.0 eq.) after 26 d. The sample was diluted to 200 µM referring to the initial A concentration for determination of the 5' AMP yield and to 1 mM for determination of the 5' ADP yield.

**Product**	**Integral** (mAU x min)	$c_{sample\ vial}$ (µM)	$c_{reaction\ mixture}$ (mM)	**Yield** (%)
5' AMP	0.0605	34.78	17.39	17.39
5' ADP	0.0257	9.23	0.92	0.92

Supplementary Table 22. Second analysis runs of the reaction starting from A (100 mM, 1.0 eq.), H_3PO_3 (3.0 eq.) and urea (1.0 eq.) after 26 d. The sample was diluted to 200 µM referring to the initial A concentration for determination of the 5' AMP yield and to 1 mM for determination of the 5' ADP yield.

**Product**	**Integral** (mAU x min)	$c_{sample\ vial}$ (µM)	$c_{reaction\ mixture}$ (mM)	**Yield** (%)	**Ø Yield** (%)
5' AMP	0.0698	40.12	20.06	20.06	18.72 ± 1.89
5' ADP	0.0333	11.98	1.20	1.20	1.06 ± 0.19

Reaction with 5’ AMP

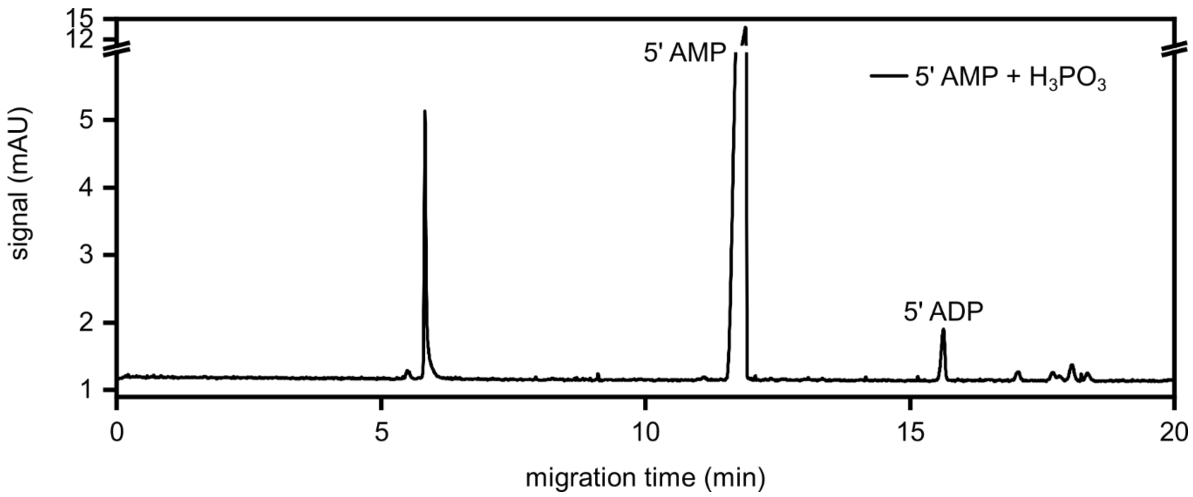

Supplementary Figure 52. First analysis run of the reaction starting from 5’ AMP (100 mM, 1.0 eq.), H_3PO_3 (3.0 eq.) and urea (1.0 eq.) after 7 d. The sample was diluted to 1 mM referring to the initial 5’ AMP concentration for determination of the 5’ ADP and 5’ ATP yield. Conditions of the electrophoretic separation: BFS capillary (l = 80 cm, length to detector: 71.5 cm); BGE: NH_4FA (30 mM, pH 9.5); CE inlet: 30 kV, pressure driven sample injection: 30 mbar for 10 s, detection at 254 nm.

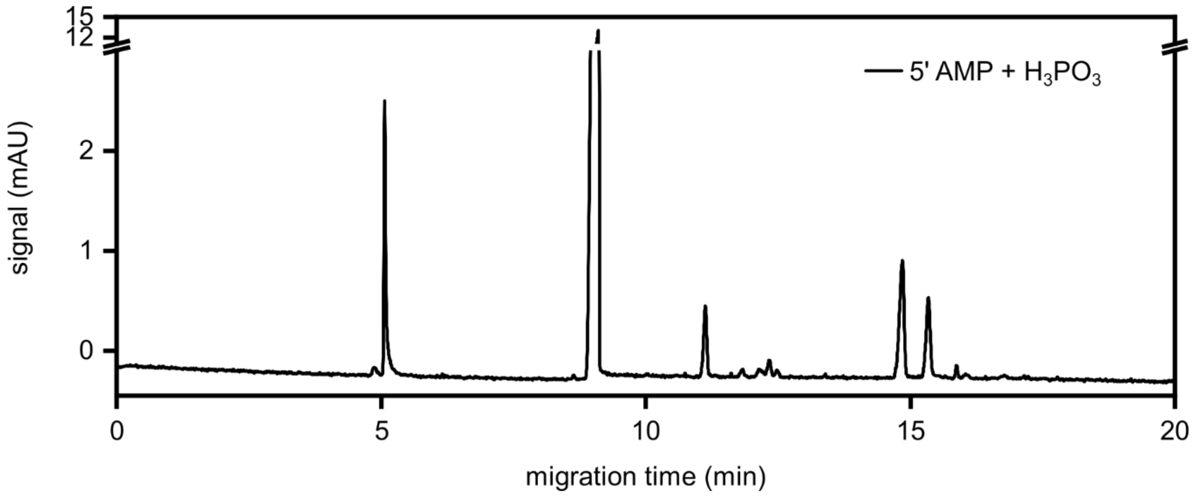

Supplementary Figure 53. Second analysis run of the reaction starting from 5’ AMP (100 mM, 1.0 eq.), H_3PO_3 (3.0 eq.) and urea (1.0 eq.) after 7 d. The sample was diluted to 1 mM referring to the initial 5’ AMP concentration for determination of the 5’ ADP and 5’ ATP yield. Conditions of the electrophoretic separation: BFS capillary (l = 80 cm, length to detector: 71.5 cm); BGE: NH_4FA (30 mM, pH 9.5); CE inlet: 30 kV, pressure driven sample injection: 30 mbar for 10 s, detection at 254 nm.

Supplementary Table 23. First analysis run of the reaction starting from 5’ AMP (100 mM, 1.0 eq.), H_3PO_3 (3.0 eq.) and urea (1.0 eq.) after 7 d. The sample was diluted to 1 mM referring to the initial 5’ AMP concentration for determination of the 5’ ADP and 5’ ATP yield.

**Product**	**Integral** (mAU x min)	$c_{sample\ vial}$ (µM)	$c_{reaction\ mixture}$ (mM)	**Yield** (%)
5’ ADP	0.0618	22.22	2.22	2.22
5’ ATP	0.0216	5.18	0.52	0.52

Supplementary Table 24. Second analysis run of the reaction starting from 5’ AMP (100 mM, 1.0 eq.), H_3PO_3 (3.0 eq.) and urea (1.0 eq.) after 7 d. The sample was diluted to 1 mM referring to the initial 5’ AMP concentration for determination of the 5’ ADP and 5’ ATP yield.

**Product**	**Integral** (mAU x min)	$c_{sample\ vial}$ (µM)	$c_{reaction\ mixture}$ (mM)	**Yield** (%)	**Ø Yield** (%)
5’ ADP	0.0489	17.59	1.76	1.76	1.99 ± 0.33
5’ ATP	0.0106	2.53	0.25	0.25	0.39 ± 0.19

Reactions with 5’ ADP

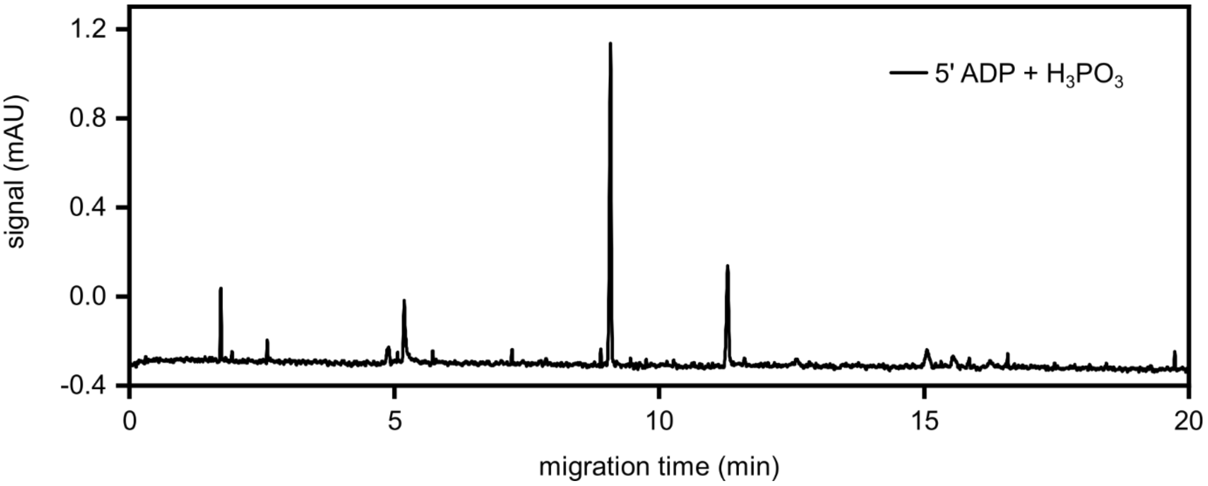

Supplementary Figure 54. First analysis run of the reaction starting from 5’ ADP (100 mM, 1.0 eq.), H_3PO_3 (3.0 eq.) and urea (1.0 eq.) after 7 d. The sample was diluted to 50 µM referring to the initial 5’ ADP concentration for determination of the 5’ AMP yield. Conditions of the electrophoretic separation: BFS capillary (l = 80 cm, length to detector: 71.5 cm); BGE: NH_4FA (30 mM, pH 9.5); CE inlet: 30 kV, pressure driven sample injection: 30 mbar for 10 s, detection at 254 nm.

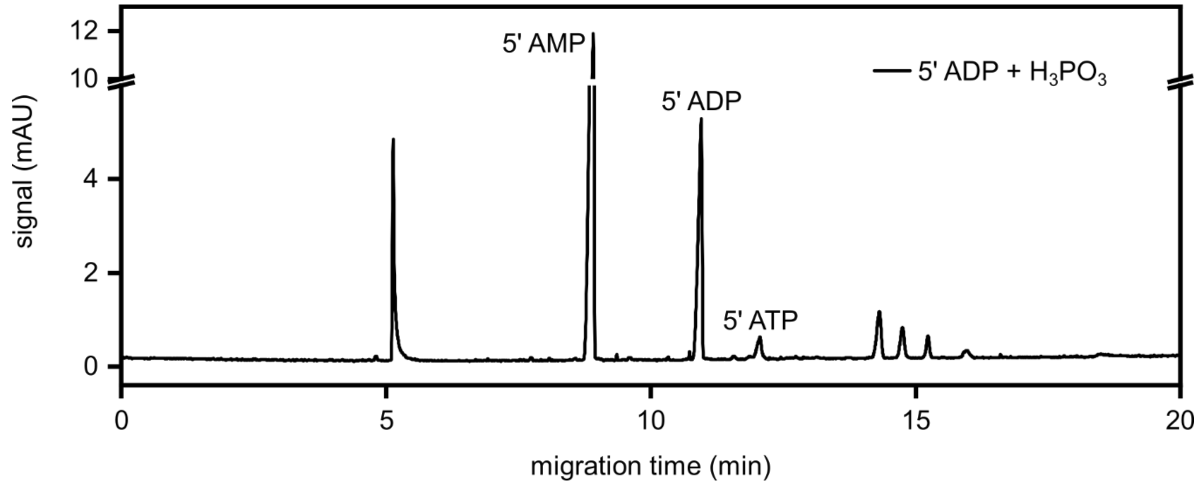

Supplementary Figure 55. First analysis run of the reaction starting from 5’ ADP (100 mM, 1.0 eq.), H_3PO_3 (3.0 eq.) and urea (1.0 eq.) after 7 d. The sample was diluted to 1 mM referring to the initial 5’ ADP concentration for determination of the 5’ ATP yield. Conditions of the electrophoretic separation: BFS capillary (l = 80 cm, length to detector: 71.5 cm); BGE: NH_4FA (30 mM, pH 9.5); CE inlet: 30 kV, pressure driven sample injection: 30 mbar for 10 s, detection at 254 nm.

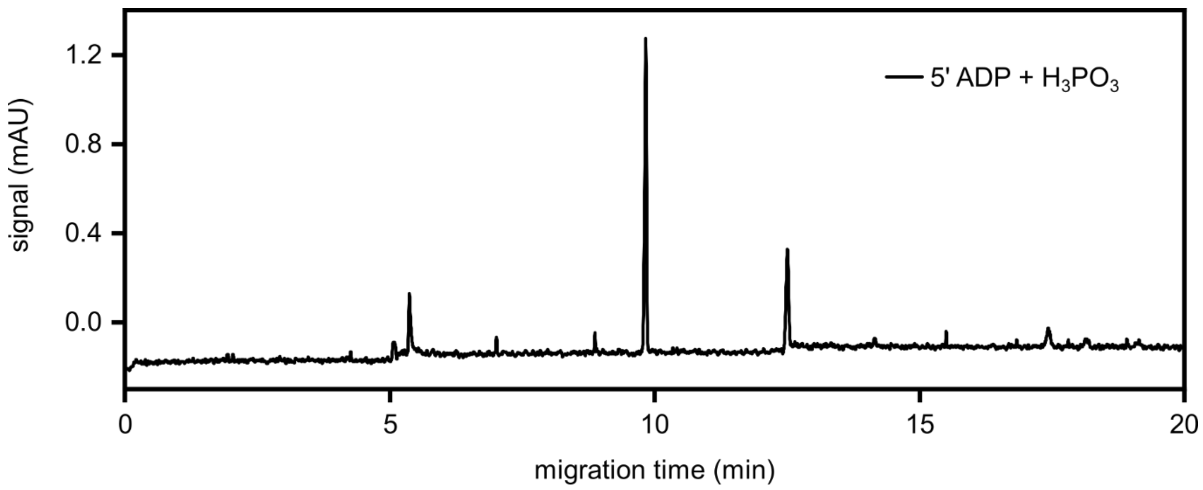

Supplementary Figure 56. Second analysis run of the reaction starting from 5′ ADP (100 mM, 1.0 eq.), H_3PO_3 (3.0 eq.) and urea (1.0 eq.) after 7 d. The sample was diluted to 50 µM referring to the initial 5′ ADP concentration for determination of the 5′ AMP yield. Conditions of the electrophoretic separation: BFS capillary (l = 80 cm, length to detector: 71.5 cm); BGE: NH_4FA (30 mM, pH 9.5); CE inlet: 30 kV, pressure driven sample injection: 30 mbar for 10 s, detection at 254 nm.

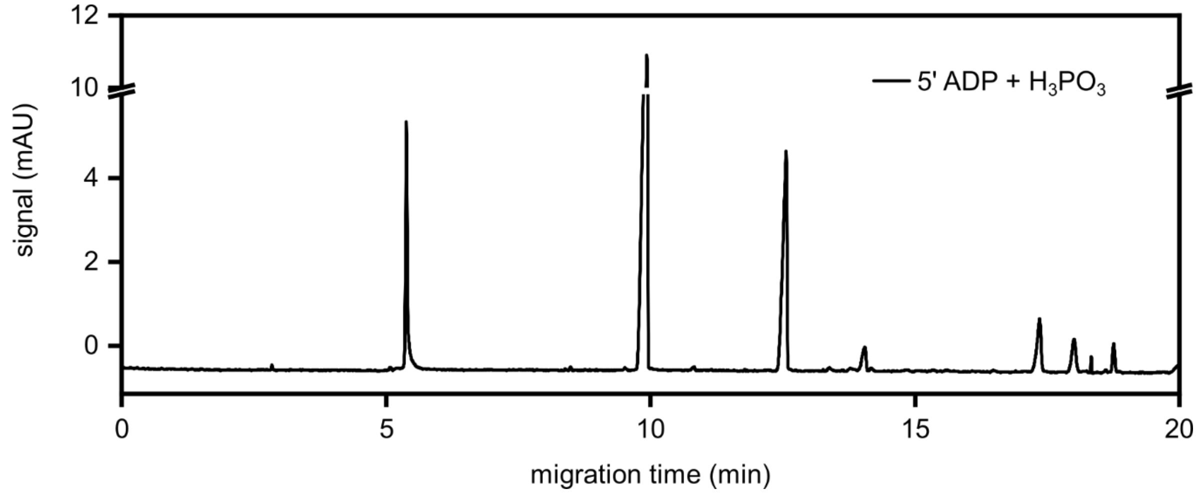

Supplementary Figure 57. Second analysis run of the reaction starting from 5′ ADP (100 mM, 1.0 eq.), H_3PO_3 (3.0 eq.) and urea (1.0 eq.) after 7 d. The sample was diluted to 1 mM referring to the initial 5′ ADP concentration for determination of the 5′ ATP yield. Conditions of the electrophoretic separation: BFS capillary (l = 80 cm, length to detector: 71.5 cm); BGE: NH_4FA (30 mM, pH 9.5); CE inlet: 30 kV, pressure driven sample injection: 30 mbar for 10 s, detection at 254 nm.

Supplementary Table 25. First analysis runs of the reaction starting from 5' ADP (100 mM, 1.0 eq.), H_3PO_3 (3.0 eq.) and urea (1.0 eq.) after 7 d. The sample was diluted to 50 µM referring to the initial 5' ADP concentration for determination of the 5' AMP yield and to 1 mM for determination of the 5' ATP yield.

**Product**	**Integral** (mAU x min)	$c_{sample\ vial}$ (µM)	$c_{reaction\ mixture}$ (mM)	**Yield** (%)
5' AMP	0.0576	33.11	66.21	66.21
5' ATP	0.0430	10.30	1.03	1.03

Supplementary Table 26. Second analysis runs of the reaction starting from 5' ADP (100 mM, 1.0 eq.), H_3PO_3 (3.0 eq.) and urea (1.0 eq.) after 7 d. The sample was diluted to 50 µM referring to the initial 5' ADP concentration for determination of the 5' AMP yield and to 1 mM for determination of the 5' ATP yield.

**Product**	**Integral** (mAU x min)	$c_{sample\ vial}$ (µM)	$c_{reaction\ mixture}$ (mM)	**Yield** (%)	**Ø Yield** (%)
5' AMP	0.0621	35.71	71.43	71.43	68.82 ± 3.69
5' ATP	0.0479	11.48	1.15	1.15	1.09 ± 0.08

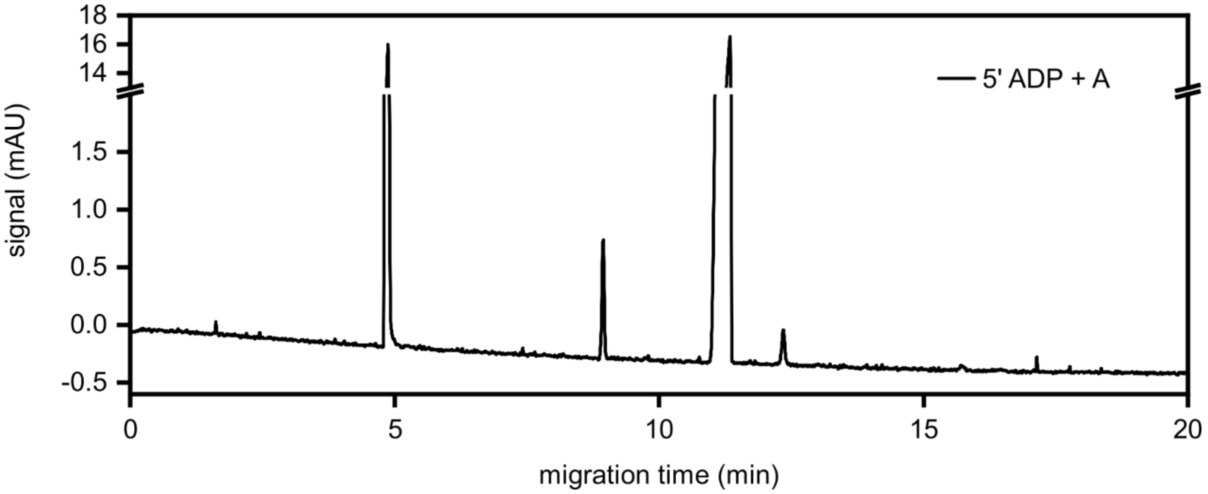

Supplementary Figure 58. First analysis run of the reaction starting from 5’ ADP (100 mM, 1.0 eq.), A (1.0 eq.) and urea (1.0 eq.) after 7 d. The sample was diluted to 1 mM referring to the initial 5’ ADP concentration for determination of the 5’ AMP and 5’ ATP yield. Conditions of the electrophoretic separation: BFS capillary (l = 80 cm, length to detector: 71.5 cm); BGE: NH_4FA (30 mM, pH 9.5); CE inlet: 30 kV, pressure driven sample injection: 30 mbar for 10 s, detection at 254 nm.

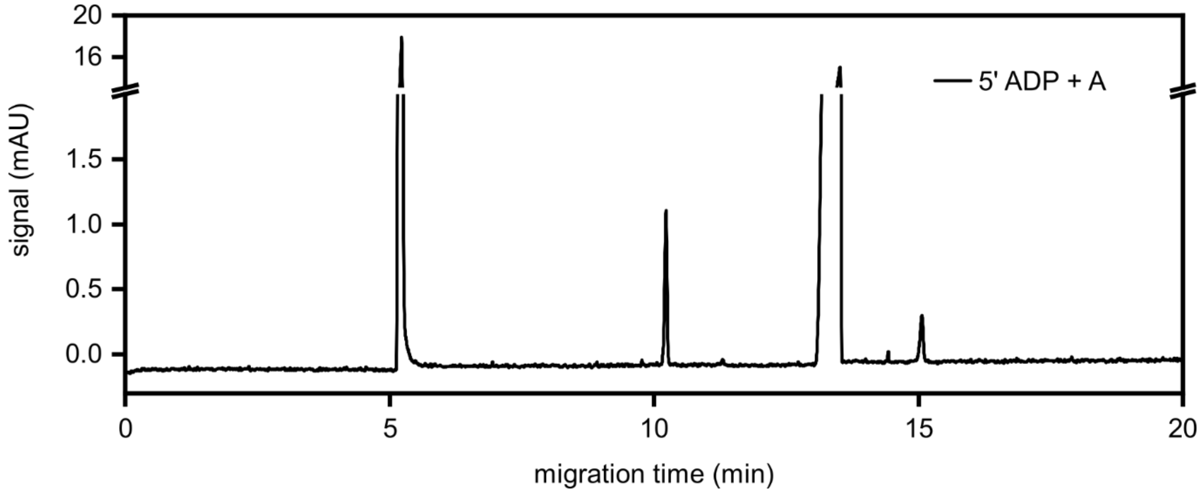

Supplementary Figure 59. Second analysis run of the reaction starting from 5’ ADP (100 mM, 1.0 eq.), A (1.0 eq.) and urea (1.0 eq.) after 7 d. The sample was diluted to 1 mM referring to the initial 5’ ADP concentration for determination of the 5’ AMP and 5’ ATP yield. Conditions of the electrophoretic separation: BFS capillary (l = 80 cm, length to detector: 71.5 cm); BGE: NH_4FA (30 mM, pH 9.5); CE inlet: 30 kV, pressure driven sample injection: 30 mbar for 10 s, detection at 254 nm.

Supplementary Table 27. First analysis run of the reaction starting from 5′ ADP (100 mM, 1.0 eq.), A (1.0 eq.) and urea (1.0 eq.) after 7 d. The sample was diluted to 1 mM referring to the initial 5′ ADP concentration for determination of the 5′ AMP and 5′ ATP yield.

**Product**	**Integral** (mAU x min)	$c_{\text{sample vial}}$ (µM)	$c_{\text{reaction mixture}}$ (mM)	**Yield** (%)
5′ AMP	0.0549	31.53	3.15	3.15
5′ ATP	0.0196	4.69	0.47	0.47

Supplementary Table 28. Second analysis run of the reaction starting from 5′ ADP (100 mM, 1.0 eq.), A (1.0 eq.) and urea (1.0 eq.) after 7 d. The sample was diluted to 1 mM referring to the initial 5′ ADP concentration for determination of the 5′ AMP and 5′ ATP yield.

**Product**	**Integral** (mAU x min)	$c_{\text{sample vial}}$ (µM)	$c_{\text{reaction mixture}}$ (mM)	**Yield** (%)	**Ø Yield** (%)
5′ AMP	0.0621	35.69	3.57	3.57	3.36 ± 0.29
5′ ATP	0.0247	5.92	0.59	0.59	0.53 ± 0.09

Reaction with cytidine

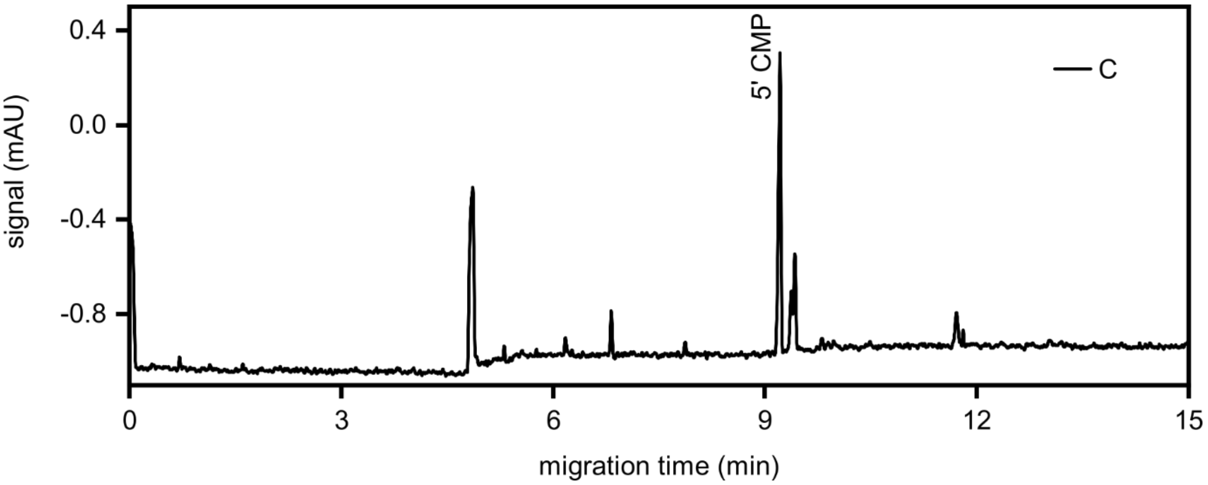

Supplementary Figure 60. First analysis run of the reaction starting from cytidine (C) (100 mM, 1.0 eq.), H_3PO_3 (3.0 eq.) and urea (1.0 eq.) after 7 d. The sample was diluted to 200 µM referring to the initial C concentration for determination of the 5' CMP yield. Conditions of the electrophoretic separation: BFS capillary (l = 80 cm, length to detector: 71.5 cm); BGE: NH_4FA (30 mM, pH 9.5); CE inlet: 30 kV, pressure driven sample injection: 30 mbar for 10 s, detection at 254 nm.

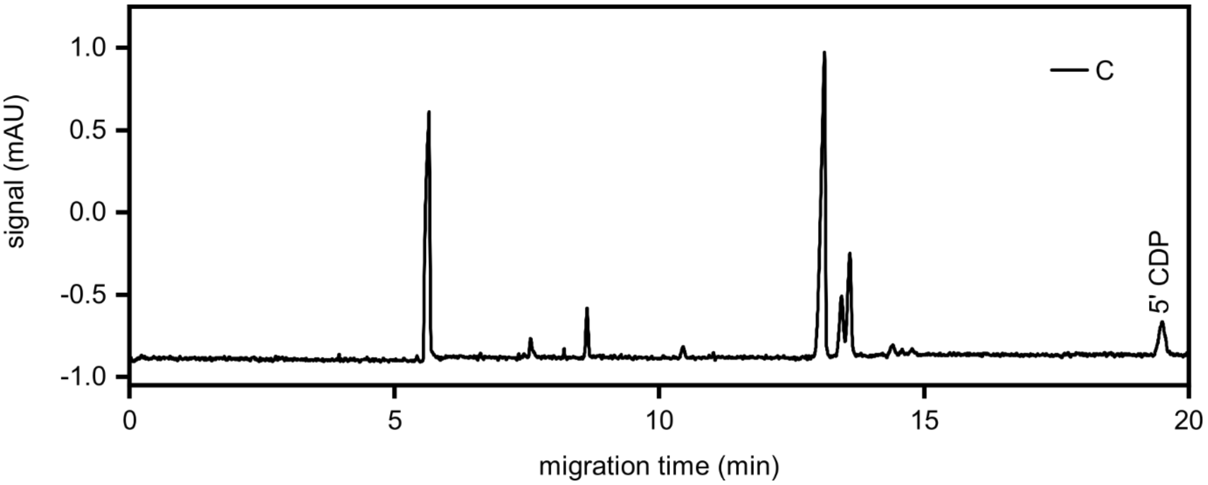

Supplementary Figure 61. First analysis run of the reaction starting from C (100 mM, 1.0 eq.), H_3PO_3 (3.0 eq.) and urea (1.0 eq.) after 7 d. The sample was diluted to 500 µM referring to the initial C concentration for determination of the 5' CDP yield. Conditions of the electrophoretic separation: BFS capillary (l = 80 cm, length to detector: 71.5 cm); BGE: NH_4FA (30 mM, pH 9.5); CE inlet: 30 kV, pressure driven sample injection: 30 mbar for 10 s, detection at 254 nm.

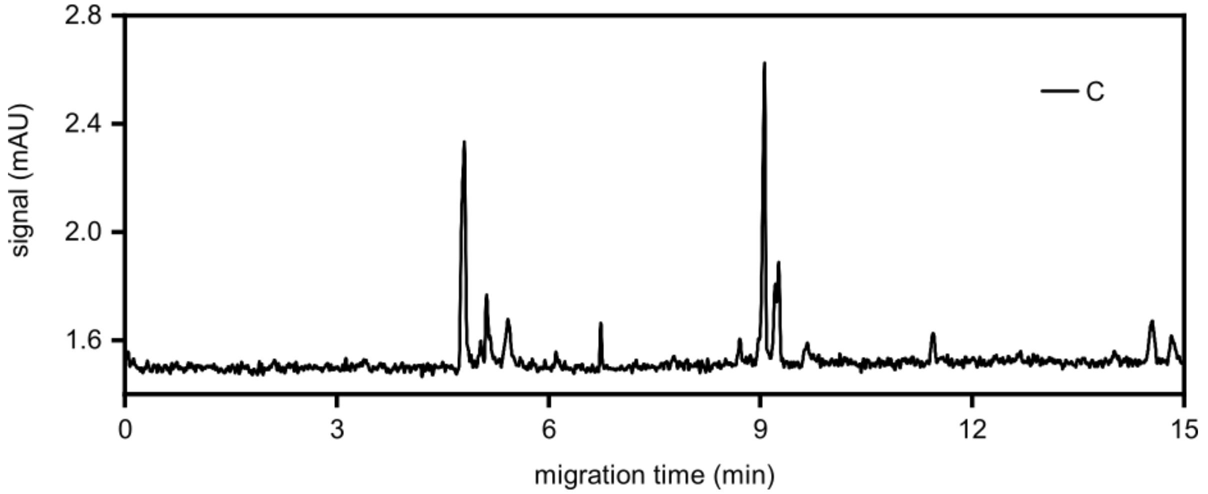

Supplementary Figure 62. Second analysis run of the reaction starting from C (100 mM, 1.0 eq.), H_3PO_3 (3.0 eq.) and urea (1.0 eq.) after 7 d. The sample was diluted to 200 µM referring to the initial C concentration for determination of the 5' CMP yield. Conditions of the electrophoretic separation: BFS capillary (l = 80 cm, length to detector: 71.5 cm); BGE: NH_4FA (30 mM, pH 9.5); CE inlet: 30 kV, pressure driven sample injection: 30 mbar for 10 s, detection at 254 nm.

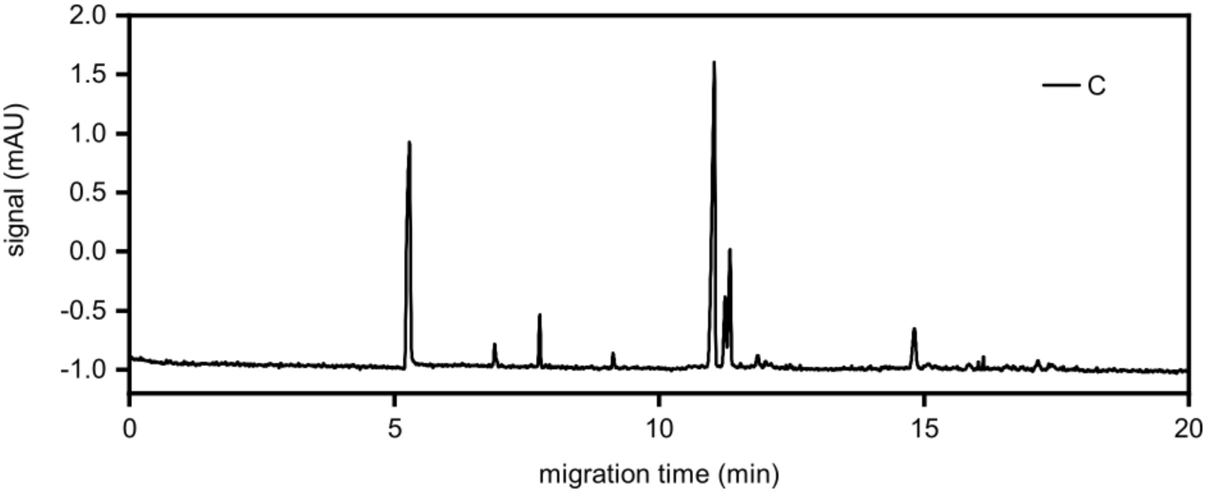

Supplementary Figure 63. Second analysis run of the reaction starting from C (100 mM, 1.0 eq.), H_3PO_3 (3.0 eq.) and urea (1.0 eq.) after 7 d. The sample was diluted to 500 µM referring to the initial C concentration for determination of the 5' CDP yield. Conditions of the electrophoretic separation: BFS capillary (l = 80 cm, length to detector: 71.5 cm); BGE: NH_4FA (30 mM, pH 9.5); CE inlet: 30 kV, pressure driven sample injection: 30 mbar for 10 s, detection at 254 nm.

Supplementary Table 29. First analysis runs of the reaction starting from C (100 mM, 1.0 eq.), H_3PO_3 (3.0 eq.) and urea (1.0 eq.) after 7 d. The sample was diluted to 200 µM referring to the initial C concentration for determination of the 5′ CMP yield and to 500 µM for determination of the 5′ CDP yield.

**Product**	**Integral** (mAU x min)	$c_{sample\ vial}$ (µM)	$c_{reaction\ mixture}$ (mM)	**Yield** (%)
5′ CMP	0.0537	49.70	24.85	24.85
5′ CDP	0.0287	16.39	3.28	3.28

Supplementary Table 30. Second analysis runs of the reaction starting from C (100 mM, 1.0 eq.), H_3PO_3 (3.0 eq.) and urea (1.0 eq.) after 7 d. The sample was diluted to 200 µM referring to the initial C concentration for determination of the 5′ CMP yield and to 500 µM for determination of the 5′ CDP yield.

**Product**	**Integral** (mAU x min)	$c_{sample\ vial}$ (µM)	$c_{reaction\ mixture}$ (mM)	**Yield** (%)	**Ø Yield** (%)
5′ CMP	0.0463	42.90	21.45	21.45	23.15 ± 2.40
5′ CDP	0.0244	13.96	2.79	2.79	3.04 ± 0.34

Reaction with uridine

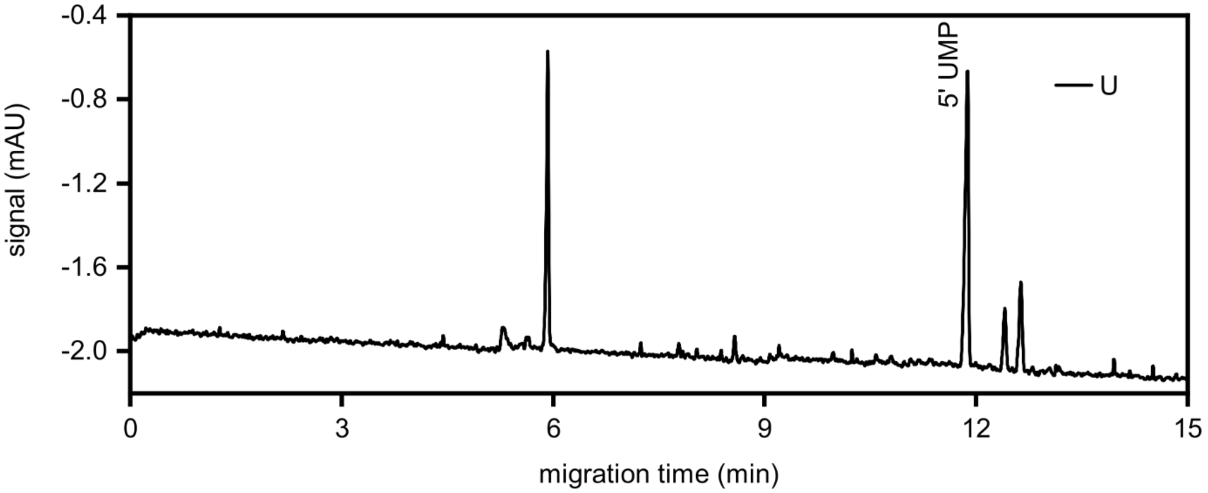

Supplementary Figure 64. First analysis run of the reaction starting from uridine (U) (100 mM, 1.0 eq.), H_3PO_3 (3.0 eq.) and urea (1.0 eq.) after 7 d. The sample was diluted to 200 µM referring to the initial U concentration for determination of the 5' UMP yield. Conditions of the electrophoretic separation: BFS capillary (l = 80 cm, length to detector: 71.5 cm); BGE: NH_4FA (30 mM, pH 9.5); CE inlet: 30 kV, pressure driven sample injection: 30 mbar for 10 s, detection at 254 nm.

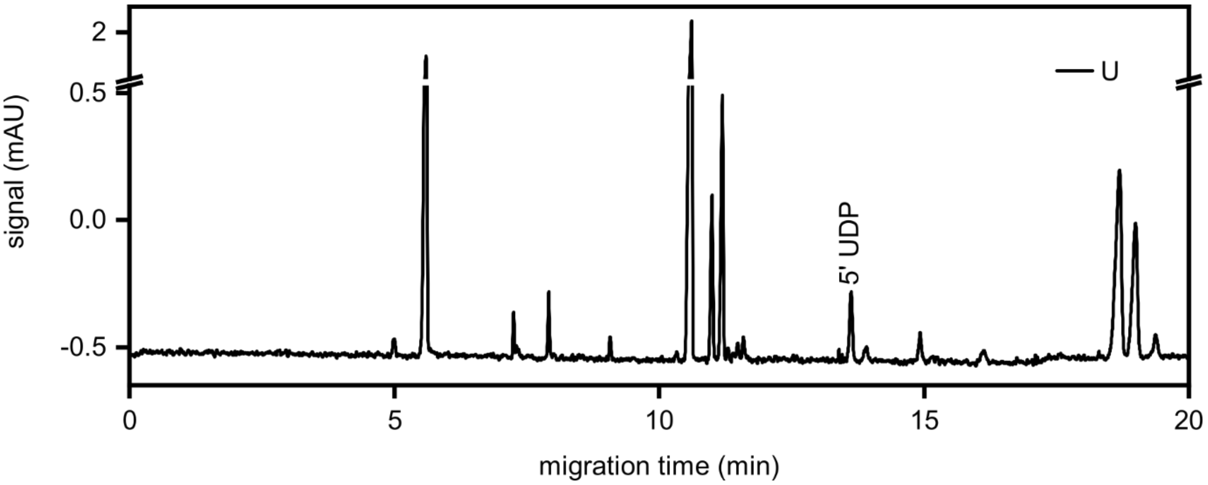

Supplementary Figure 65. First analysis run of the reaction starting from U (100 mM, 1.0 eq.), H_3PO_3 (3.0 eq.) and urea (1.0 eq.) after 7 d. The sample was diluted to 500 µM referring to the initial U concentration for determination of the 5' UDP yield. Conditions of the electrophoretic separation: BFS capillary (l = 80 cm, length to detector: 71.5 cm); BGE: NH_4FA (30 mM, pH 9.5); CE inlet: 30 kV, pressure driven sample injection: 30 mbar for 10 s, detection at 254 nm.

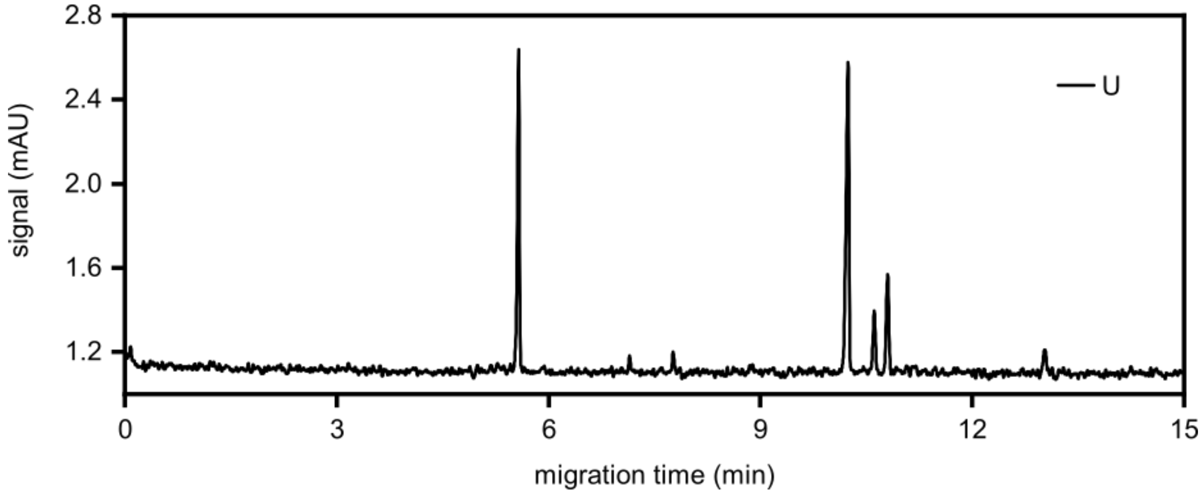

Supplementary Figure 66. Second analysis run of the reaction starting from U (100 mM, 1.0 eq.), H_3PO_3 (3.0 eq.) and urea (1.0 eq.) after 7 d. The sample was diluted to 200 µM referring to the initial U concentration for determination of the 5' UMP yield. Conditions of the electrophoretic separation: BFS capillary (l = 80 cm, length to detector: 71.5 cm); BGE: NH_4FA (30 mM, pH 9.5); CE inlet: 30 kV, pressure driven sample injection: 30 mbar for 10 s, detection at 254 nm.

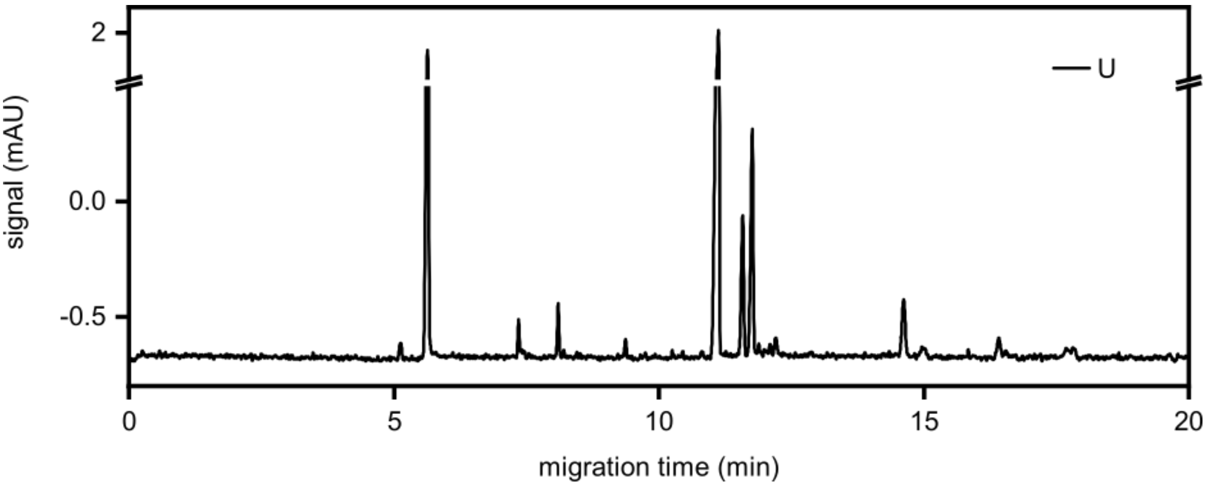

Supplementary Figure 67. Second analysis run of the reaction starting from U (100 mM, 1.0 eq.), H_3PO_3 (3.0 eq.) and urea (1.0 eq.) after 7 d. The sample was diluted to 500 µM referring to the initial U concentration for determination of the 5' UDP yield. Conditions of the electrophoretic separation: BFS capillary (l = 80 cm, length to detector: 71.5 cm); BGE: NH_4FA (30 mM, pH 9.5); CE inlet: 30 kV, pressure driven sample injection: 30 mbar for 10 s, detection at 254 nm.

Supplementary Table 31. First analysis runs of the reaction starting from U (100 mM, 1.0 eq.), H_3PO_3 (3.0 eq.) and urea (1.0 eq.) after 7 d. The sample was diluted to 200 µM referring to the initial U concentration for determination of the 5' UMP yield and to 500 µM for determination of the 5' UDP yield.

**Product**	**Integral** (mAU x min)	$c_{sample\ vial}$ (µM)	$c_{reaction\ mixture}$ (mM)	**Yield** (%)
5' UMP	0.0816	62.77	31.38	31.38
5' UDP	0.0176	7.65	1.53	1.53

Supplementary Table 32. Second analysis runs of the reaction starting from U (100 mM, 1.0 eq.), H_3PO_3 (3.0 eq.) and urea (1.0 eq.) after 7 d. The sample was diluted to 200 µM referring to the initial U concentration for determination of the 5' UMP yield and to 500 µM for determination of the 5' UDP yield.

**Product**	**Integral** (mAU x min)	$c_{sample\ vial}$ (µM)	$c_{reaction\ mixture}$ (mM)	**Yield** (%)	**Ø Yield** (%)
5' UMP	0.0725	55.76	27.88	27.88	29.63 ± 2.48
5' UDP	0.0178	7.76	1.55	1.55	1.54 ± 0.01

Reaction with guanosine

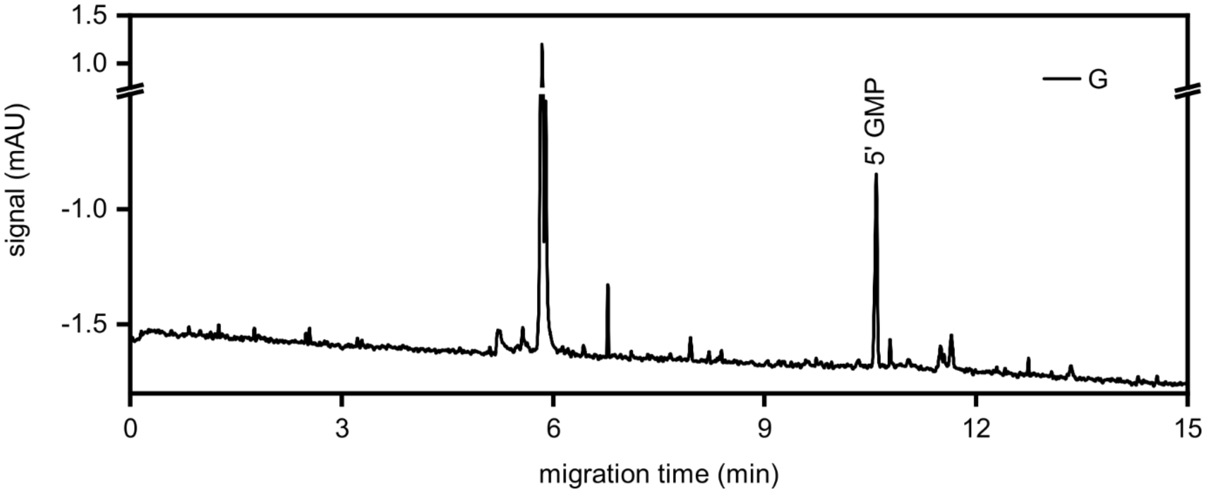

Supplementary Figure 68. First analysis run of the reaction starting from guanosine (G) (100 mM, 1.0 eq.), H_3PO_3 (3.0 eq.) and urea (1.0 eq.) after 7 d. The sample was diluted to 200 µM referring to the initial G concentration for determination of the 5' GMP yield. Conditions of the electrophoretic separation: BFS capillary (l = 80 cm, length to detector: 71.5 cm); BGE: NH_4FA (30 mM, pH 9.5); CE inlet: 30 kV, pressure driven sample injection: 30 mbar for 10 s, detection at 254 nm.

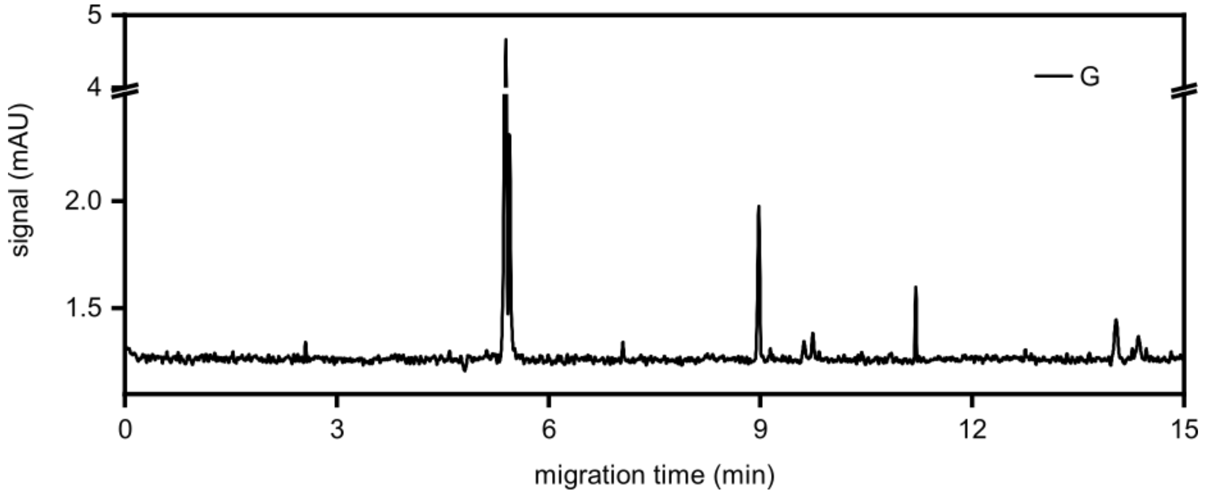

Supplementary Figure 69. Second analysis run of the reaction starting from G (100 mM, 1.0 eq.), H_3PO_3 (3.0 eq.) and urea (1.0 eq.) after 7 d. The sample was diluted to 200 µM referring to the initial G concentration for determination of the 5' GMP yield**.** Conditions of the electrophoretic separation: BFS capillary (l = 80 cm, length to detector: 71.5 cm); BGE: NH_4FA (30 mM, pH 9.5); CE inlet: 30 kV, pressure driven sample injection: 30 mbar for 10 s, detection at 254 nm.

Supplementary Table 33. First analysis run of the reaction starting from G (100 mM, 1.0 eq.), H_3PO_3 (3.0 eq.) and urea (1.0 eq.) after 7 d. The sample was diluted to 200 µM referring to the initial G concentration for determination of the 5′ GMP yield.

**Product**	**Integral** (mAU x min)	**$c_{sample\ vial}$** (µM)	**$c_{reaction\ mixture}$** (mM)	**Yield** (%)
5′ GMP	0.0351	16.41	8.20	8.20

Supplementary Table 34. Second analysis run of the reaction starting from G (100 mM, 1.0 eq.), H_3PO_3 (3.0 eq.) and urea (1.0 eq.) after 7 d. The sample was diluted to 200 µM referring to the initial G concentration for determination of the 5′ GMP yield.

**Product**	**Integral** (mAU x min)	**$c_{sample\ vial}$** (µM)	**$c_{reaction\ mixture}$** (mM)	**Yield** (%)	**Ø Yield** (%)
5′ GMP	0.0255	11.91	5.96	5.96	7.08 ± 1.59

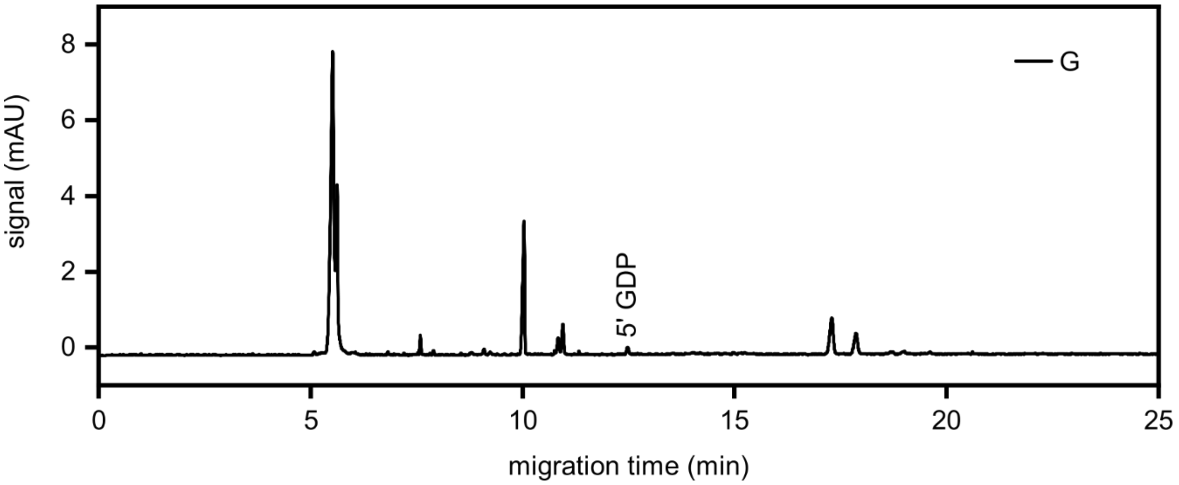

Supplementary Figure 70. Assignment of 5′ guanosine diphosphate obtained from the reaction with G (100 mM, 1.0 eq.), H_3PO_3 (3.0 eq.) and urea (1.0 eq.) after 7 d. The sample was diluted to 1 mM referring to the initial G concentration. Conditions of the electrophoretic separation: BFS capillary (l = 80 cm, length to detector: 71.5 cm); BGE: NH_4FA (30 mM, pH 9.5); CE inlet: 30 kV, pressure driven sample injection: 30 mbar for 10 s, detection at 254 nm.

Reaction with deoxycytidine

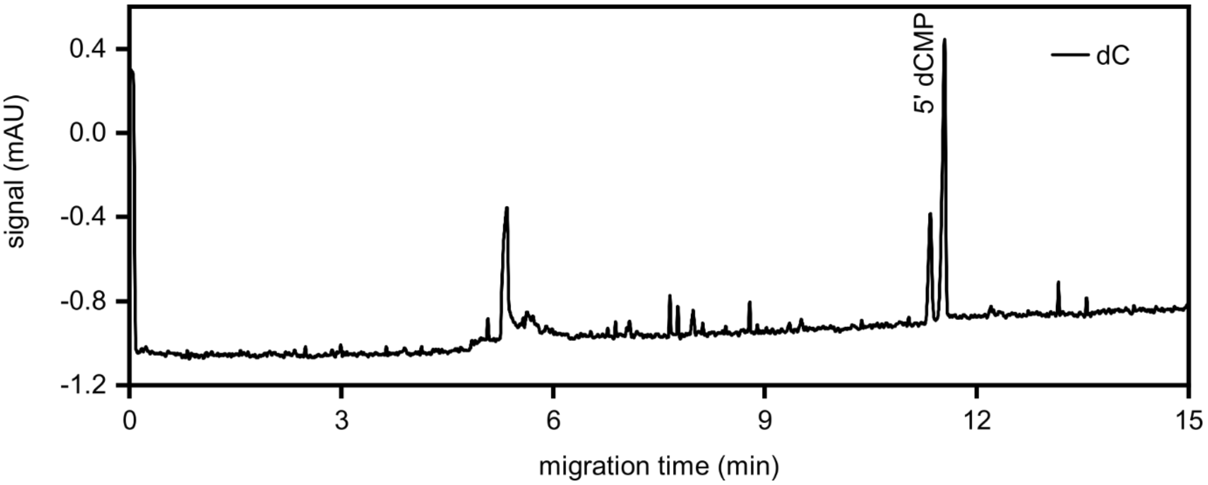

Supplementary Figure 71. First analysis run of the reaction starting from deoxycytidine (dC) (100 mM, 1.0 eq.), H_3PO_3 (3.0 eq.) and urea (1.0 eq.) after 7 d. The sample was diluted to 200 µM referring to the initial dC concentration for determination of the 5' dCMP yield. Conditions of the electrophoretic separation: BFS capillary (l = 80 cm, length to detector: 71.5 cm); BGE: NH_4FA (30 mM, pH 9.5); CE inlet: 30 kV, pressure driven sample injection: 30 mbar for 10 s, detection at 254 nm.

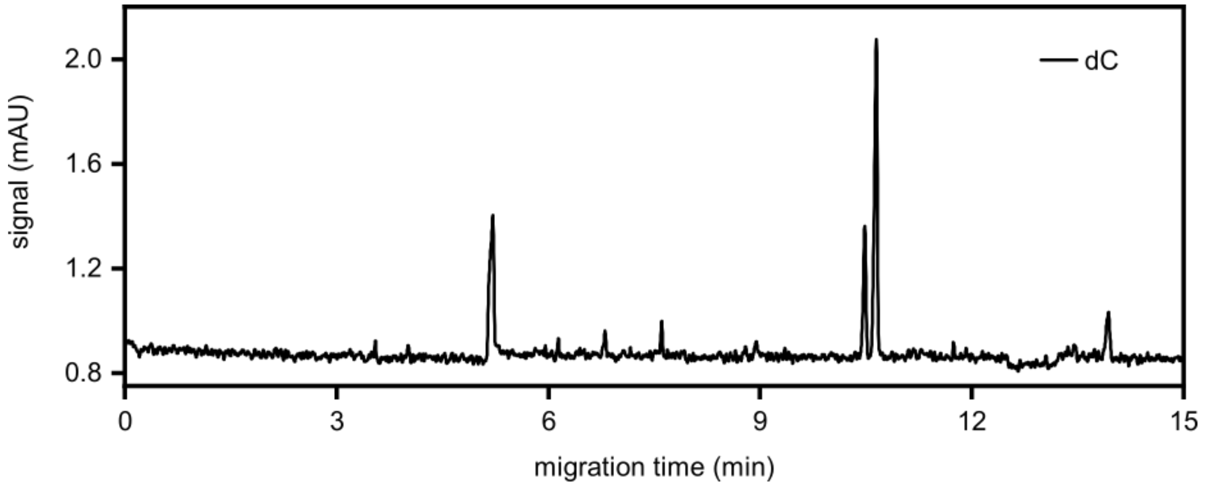

Supplementary Figure 72. Second analysis run of the reaction starting from dC (100 mM, 1.0 eq.), H_3PO_3 (3.0 eq.) and urea (1.0 eq.) after 7 d. The sample was diluted to 200 µM referring to the initial dC concentration for determination of the 5' dCMP yield. Conditions of the electrophoretic separation: BFS capillary (l = 80 cm, length to detector: 71.5 cm); BGE: NH_4FA (30 mM, pH 9.5); CE inlet: 30 kV, pressure driven sample injection: 30 mbar for 10 s, detection at 254 nm.

Supplementary Table 35. First analysis run of the reaction starting from dC (100 mM, 1.0 eq.), H_3PO_3 (3.0 eq.) and urea (1.0 eq.) after 7 d. The sample was diluted to 200 µM referring to the initial dC concentration for determination of the 5' dCMP yield.

**Product**	**Integral** (mAU x min)	$c_{sample\ vial}$ (µM)	$c_{reaction\ mixture}$ (mM)	**Yield** (%)
5' dCMP	0.0738	73.08	36.54	36.54

Supplementary Table 36. Second analysis run of the reaction starting from dC (100 mM, 1.0 eq.), H_3PO_3 (3.0 eq.) and urea (1.0 eq.) after 7 d. The sample was diluted to 200 µM referring to the initial dC concentration for determination of the 5' dCMP yield.

**Product**	**Integral** (mAU x min)	$c_{sample\ vial}$ (µM)	$c_{reaction\ mixture}$ (mM)	**Yield** (%)	**Ø Yield** (%)
5' dCMP	0.0580	57.42	28.71	28.71	32.62 ± 5.54

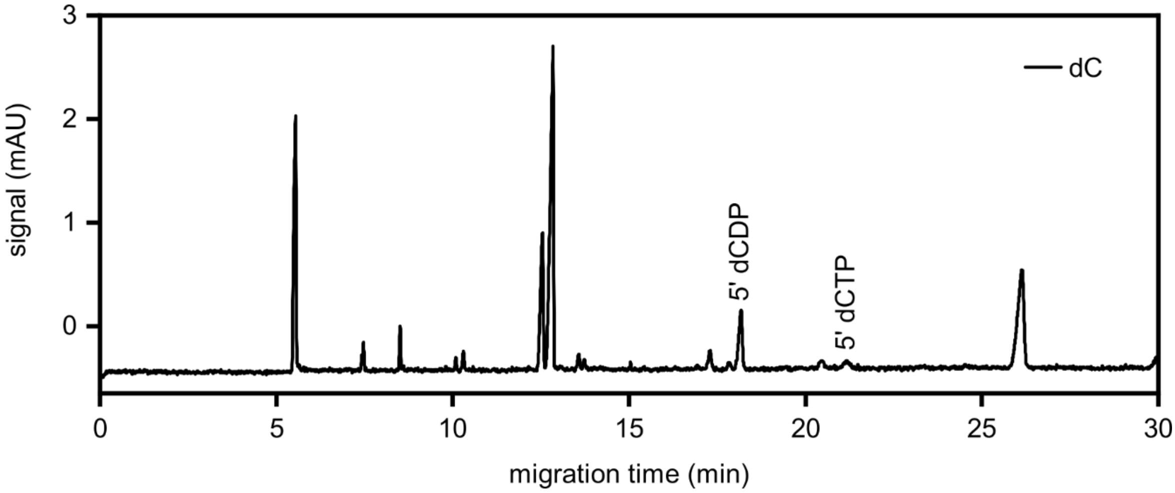

Supplementary Figure 73. Assignment of the 5' substituted polyphosphate products obtained from the reaction with dC (100 mM, 1.0 eq.), H_3PO_3 (3.0 eq.) and urea (1.0 eq.) after 7 d. The sample was diluted to 1 mM referring to the initial dC concentration. Conditions of the electrophoretic separation: BFS capillary (l = 80 cm, length to detector: 71.5 cm); BGE: NH_4FA (30 mM, pH 9.5); CE inlet: 30 kV, pressure driven sample injection: 30 mbar for 10 s, detection at 254 nm.

Reaction with deoxythymidine

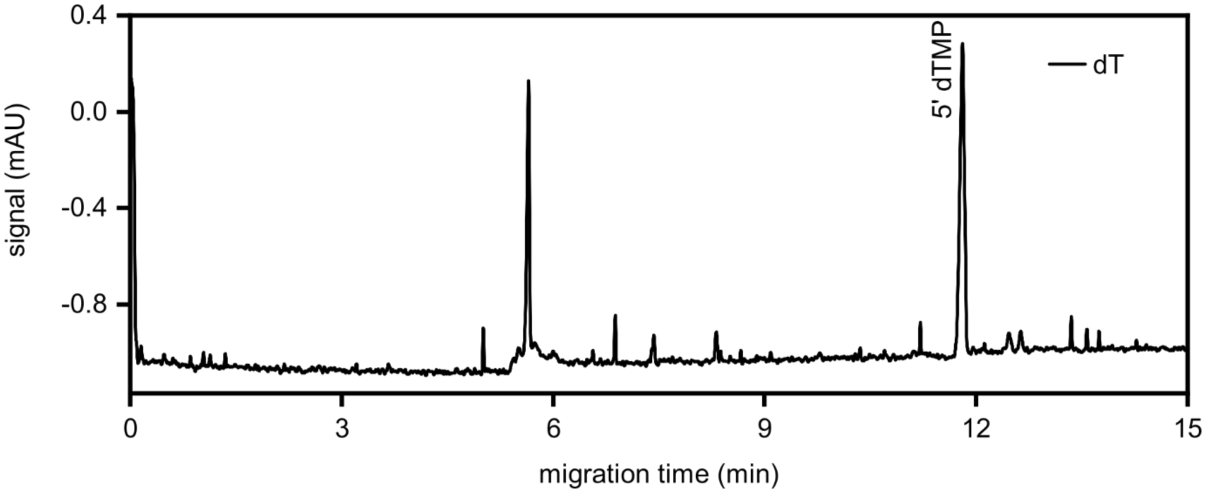

Supplementary Figure 74. First analysis run of the reaction starting from deoxythymidine (dT) (100 mM, 1.0 eq.), H_3PO_3 (3.0 eq.) and urea (1.0 eq.) after 7 d. The sample was diluted to 200 µM referring to the initial dT concentration for determination of the 5' dTMP yield. Conditions of the electrophoretic separation: BFS capillary (l = 80 cm, length to detector: 71.5 cm); BGE: NH_4FA (30 mM, pH 9.5); CE inlet: 30 kV, pressure driven sample injection: 30 mbar for 10 s, detection at 254 nm.

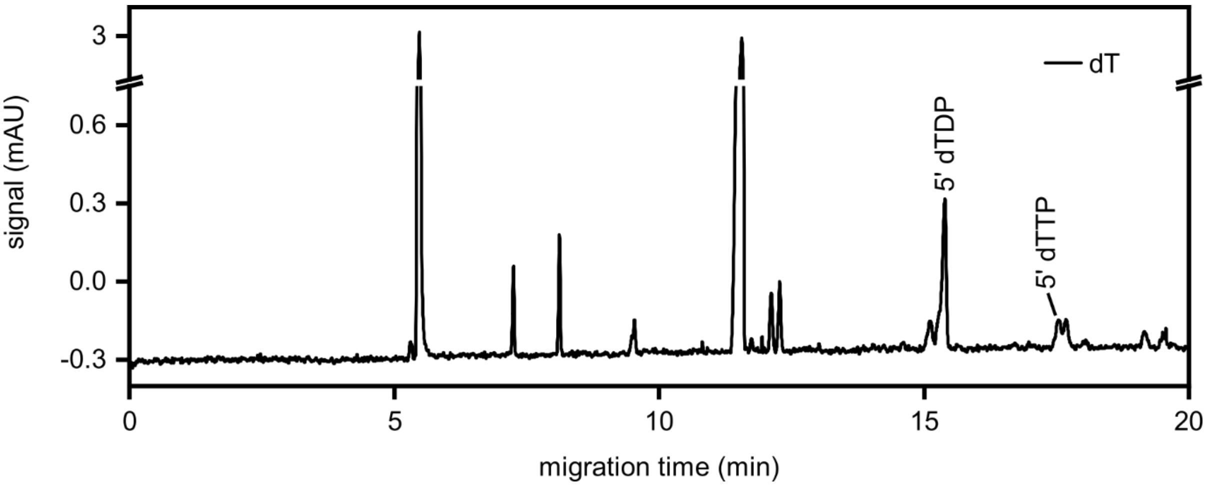

Supplementary Figure 75. First analysis run of the reaction starting from dT (100 mM, 1.0 eq.), H_3PO_3 (3.0 eq.) and urea (1.0 eq.) after 7 d. The sample was diluted to 1 mM referring to the initial dT concentration for determination of the 5' dTDP yield and assignment of the 5' deoxythymidine triphosphate (5' dTTP) signal. Conditions of the electrophoretic separation: BFS capillary (l = 80 cm, length to detector: 71.5 cm); BGE: NH_4FA (30 mM, pH 9.5); CE inlet: 30 kV, pressure driven sample injection: 30 mbar for 10 s, detection at 254 nm.

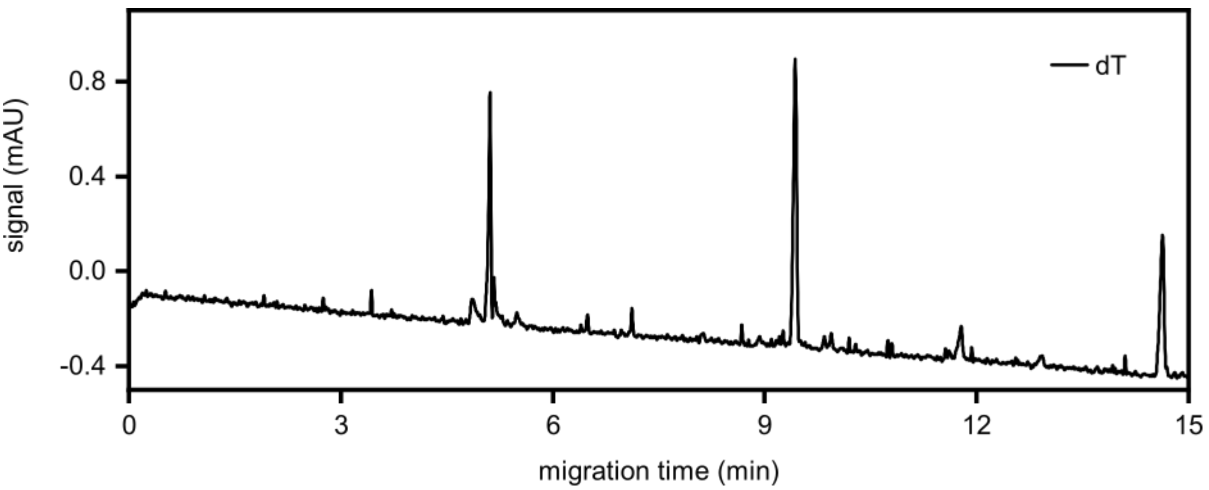

Supplementary Figure 76. Second analysis run of the reaction starting from dT (100 mM, 1.0 eq.), H_3PO_3 (3.0 eq.) and urea (1.0 eq.) after 7 d. The sample was diluted to 200 µM referring to the initial dT concentration for determination of the 5' dTMP yield. Conditions of the electrophoretic separation: BFS capillary (l = 80 cm, length to detector: 71.5 cm); BGE: NH_4FA (30 mM, pH 9.5); CE inlet: 30 kV, pressure driven sample injection: 30 mbar for 10 s, detection at 254 nm.

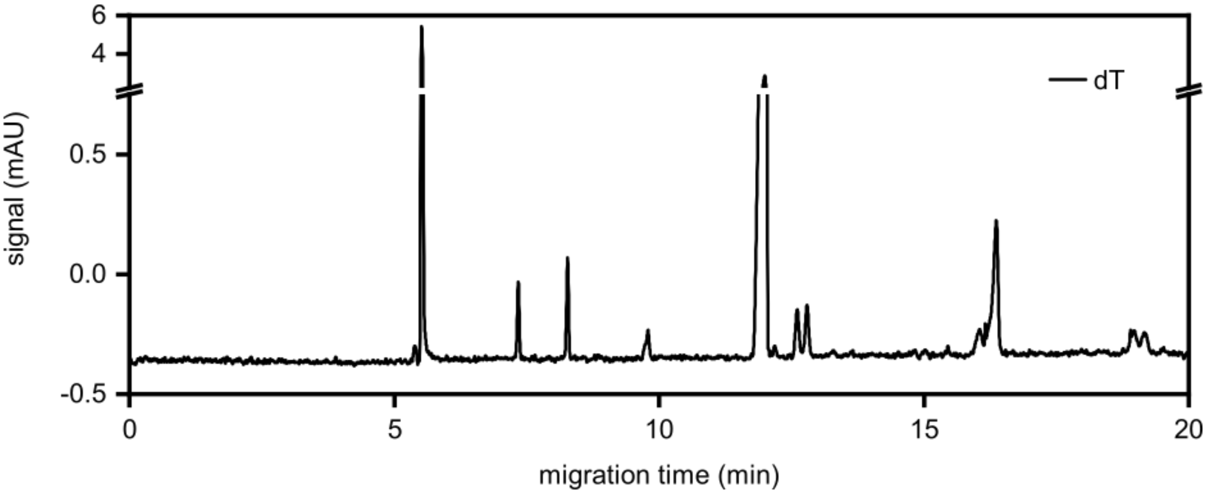

Supplementary Figure 77. Second analysis run of the reaction starting from dT (100 mM, 1.0 eq.), H_3PO_3 (3.0 eq.) and urea (1.0 eq.) after 7 d. The sample was diluted to 1 mM referring to the initial dT concentration for determination of the 5' dTDP yield. Conditions of the electrophoretic separation: BFS capillary (l = 80 cm, length to detector: 71.5 cm); BGE: NH_4FA (30 mM, pH 9.5); CE inlet: 30 kV, pressure driven sample injection: 30 mbar for 10 s, detection at 254 nm.

Supplementary Table 37. First analysis runs of the reaction starting from dT (100 mM, 1.0 eq.), H_3PO_3 (3.0 eq.) and urea (1.0 eq.) after 7 d. The sample was diluted to 200 µM referring to the initial dT concentration for determination of the 5' dTMP yield and to 1 mM for determination of the 5' dTDP yield.

**Product**	**Integral** (mAU x min)	$c_{sample\ vial}$ (µM)	$c_{reaction\ mixture}$ (mM)	**Yield** (%)
5' dTMP	0.1004	76.66	38.33	38.33
5' dTDP	0.0555	34.24	3.42	3.42

Supplementary Table 38. Second analysis runs of the reaction starting from dT (100 mM, 1.0 eq.), H_3PO_3 (3.0 eq.) and urea (1.0 eq.) after 7 d. The sample was diluted to 200 µM referring to the initial dT concentration for determination of the 5' dTMP yield and to 1 mM for determination of the 5' dTDP yield.

**Product**	**Integral** (mAU x min)	$c_{sample\ vial}$ (µM)	$c_{reaction\ mixture}$ (mM)	**Yield** (%)	**Ø Yield** (%)
5' dTMP	0.0657	50.13	25.06	25.06	31.70 ± 9.38
5' dTDP	0.0608	37.56	3.76	3.76	3.59 ± 0.23

Supplementary Note 2

Sulphur redox chemistry

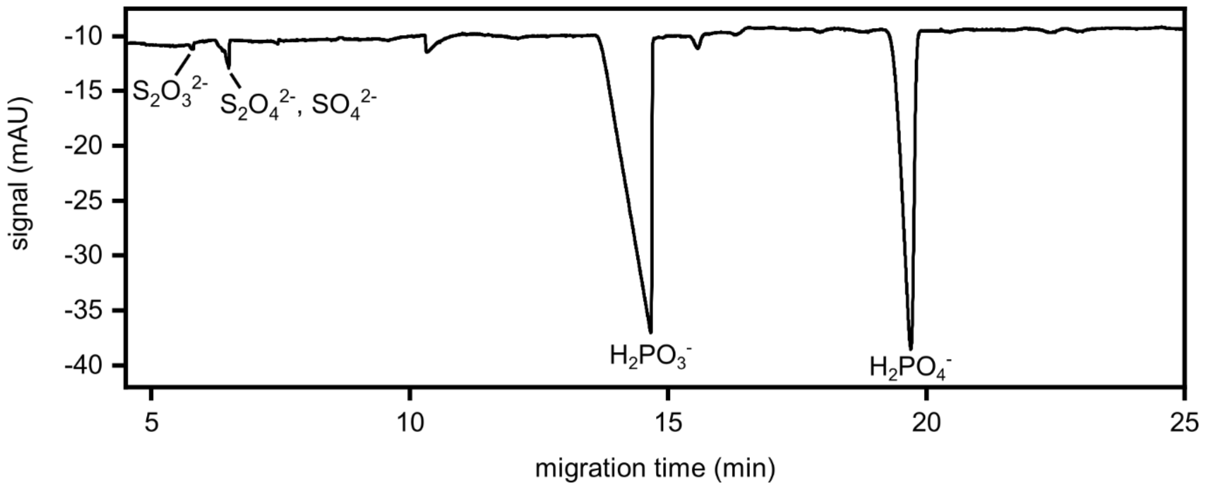

Supplementary Figure 78. Analysis of the reaction starting from A (100 mM, 1.0 eq.), H_3PO_3 (3.0 eq.) and urea (3.0 eq.) after 7 d. The sample was diluted to 10 mM referring to the initial A concentration. Conditions of the electrophoretic separation: BFS capillary (l = 80 cm, length to detector: 71.5 cm); BGE: 20 mM salicylic acid + 30 mM BIS-BIS (pH 6.0); CE inlet: −30 kV, assisting pressure 40 mbar, pressure driven sample injection: 20 mbar for 10 s, detection at 214 nm.

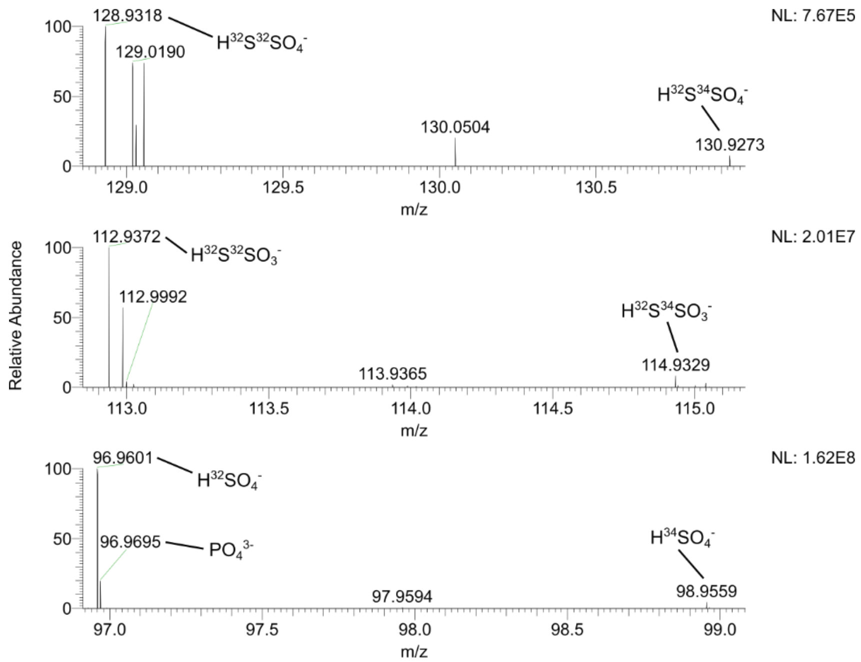

Supplementary Figure 79. Sections of the direct injection mass spectrum of the reaction starting from A (100 mM, 1.0 eq.), H_3PO_3 (3.0 eq.) and urea (3.0 eq.) after 7 d. The sample was diluted to 1 mM referring to the initial A concentration. Mass spectrometric settings: m/z 95-1425, resolution 140000, spray voltage −4.0 kV, capillary temperature = 320 °C, S-lens RF value was set to 50.

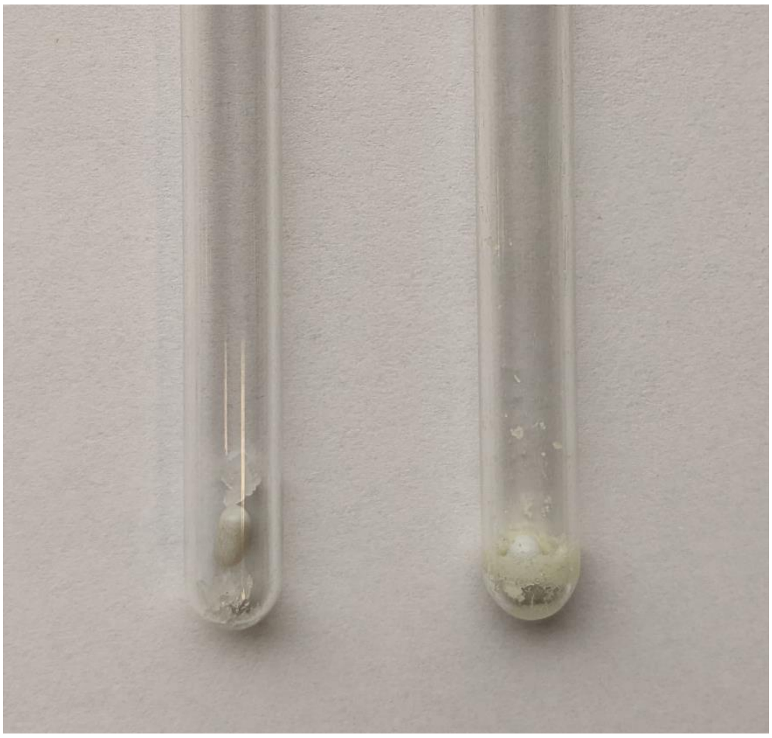

Supplementary Figure 80. Photograph of the reaction mixture starting from H_3PO_3 (300 mM) prior to the addition of liquid SO_2 (left) and after the removal of liquid SO_2 after 7 d (right).
