# Supplementary material for: Phosphorylation in liquid sulfur dioxide under prebiotically plausible conditions
Source: Commun Chem. 2022 Nov 3;5:143. doi: 10.1038/s42004-022-00761-w (PMC9814524; doi:10.1038/s42004-022-00761-w)
Supplement: Supplementary file 3 — Description of Additional Supplementary Files [file 42004_2022_761_MOESM3_ESM.pdf]

Description of Additional Supplementary Files

File Name: Supplementary Data 1

Description: extracted ion electropherograms (EIEs) of CE-MS analysis

File Name: Supplementary Data 2

Description: MS/MS spectra of dinucleotides
