# Supplementary material for: Phosphorylation in liquid sulfur dioxide under prebiotically plausible conditions
Source: Commun Chem. 2022 Nov 3;5:143. doi: 10.1038/s42004-022-00761-w (PMC9814524; doi:10.1038/s42004-022-00761-w)
Supplement: Supplementary file 4 — Supplementary Data 1 [file 42004_2022_761_MOESM4_ESM.docx]

**Supplementary Data 1**

**Extracted ion electropherograms (CE-MS)**

[Reactions with adenosine 2](#_Toc115860842)

[Reaction with 5’ AMP 21](#_Toc115860843)

[Reactions with 5’ ADP 22](#_Toc115860844)

[Reaction with cytidine 25](#_Toc115860845)

[Reaction with uridine 28](#_Toc115860846)

[Reaction with guanosine 30](#_Toc115860847)

[Reaction with deoxyadenosine 32](#_Toc115860848)

[Reaction with deoxyguanosine 33](#_Toc115860849)

[Reaction with deoxycytidine 34](#_Toc115860850)

[Reaction with deoxythymidine 36](#_Toc115860851)

[Reaction with glycerol 38](#_Toc115860852)

[Reaction with glyceraldehyde 39](#_Toc115860853)

[Reaction with D-ribose 40](#_Toc115860854)

[Reaction with sodium L-lactate 41](#_Toc115860855)

[Reaction with L-serine 42](#_Toc115860856)

[Reaction of the complete nucleoside mixture 43](#_Toc115860857)

### Reactions with adenosine

#### Variation of the H_3_PO_3_ concentration


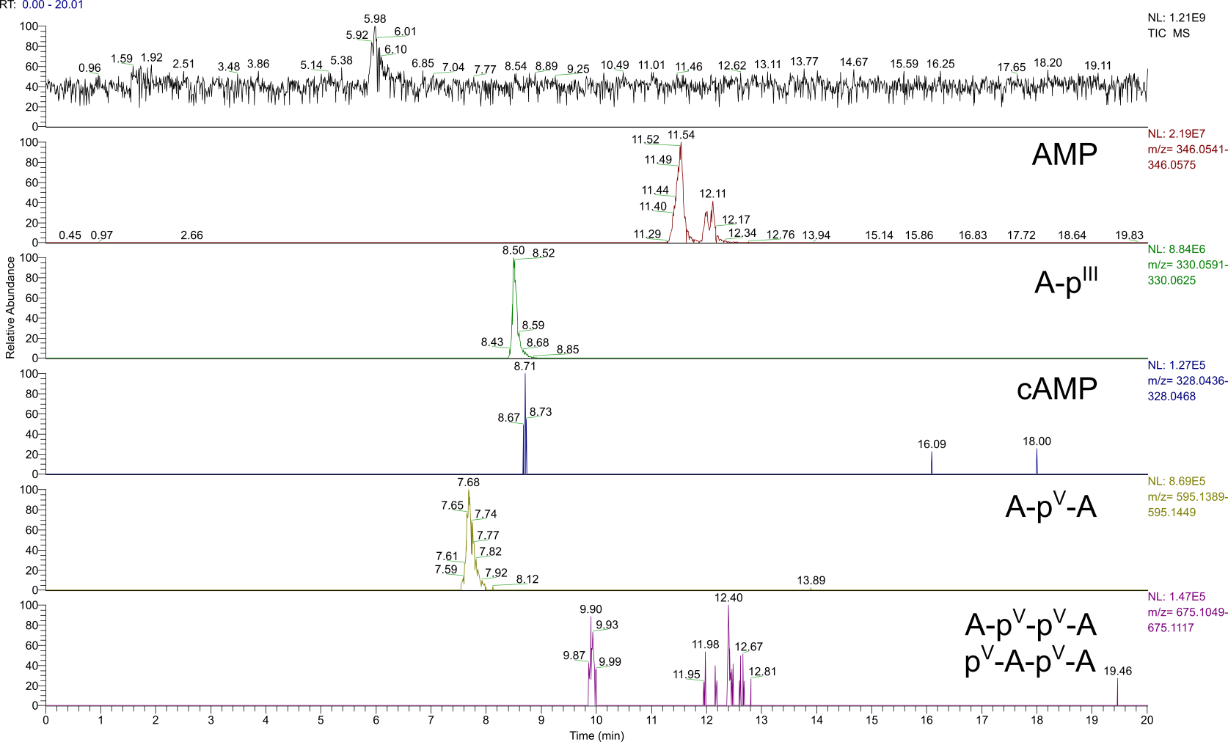


Supplementary Figure 1. Total ion electropherogram (TIC) (m/z 50-750) and extracted ion electropherograms (EIEs) (Δ = 5.0 ppm) of the products of the reaction starting from A (100 mM) and H_3_PO_3_ (1.0 eq.) after 7 d. Constitution of potential isomers has not been determined. Labels illustrate all possible phosphate/phosphonate binding modes and refer to the entirety of all formed isomers.


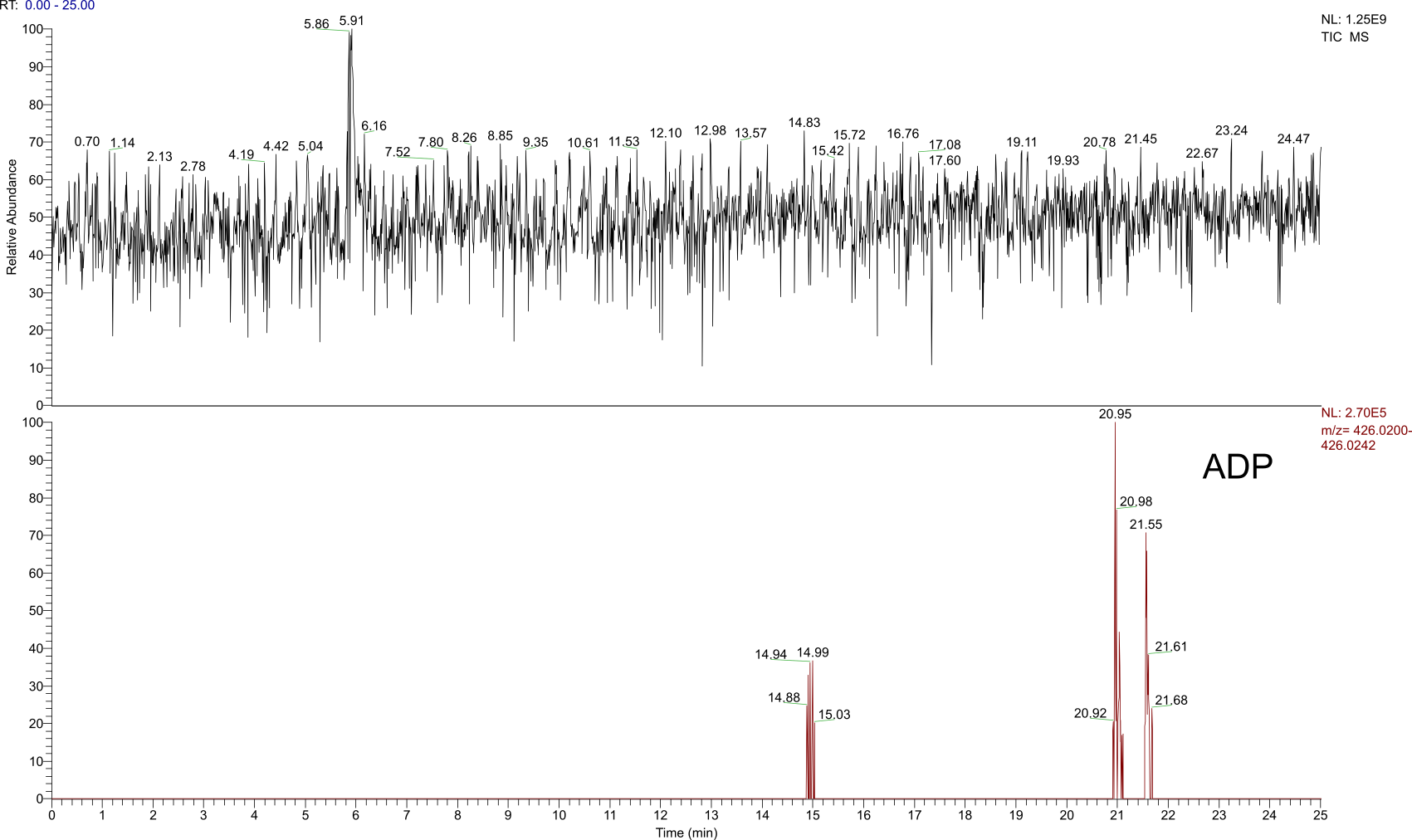


Supplementary Figure 2. TIC (m/z 50-750) and EIE (Δ = 5.0 ppm) of the products of the reaction starting from A (100 mM) and H_3_PO_3_ (1.0 eq.) after 7 d. Constitution of potential isomers has not been determined. The label illustrates all possible phosphate binding modes and refers to the entirety of all formed isomers.


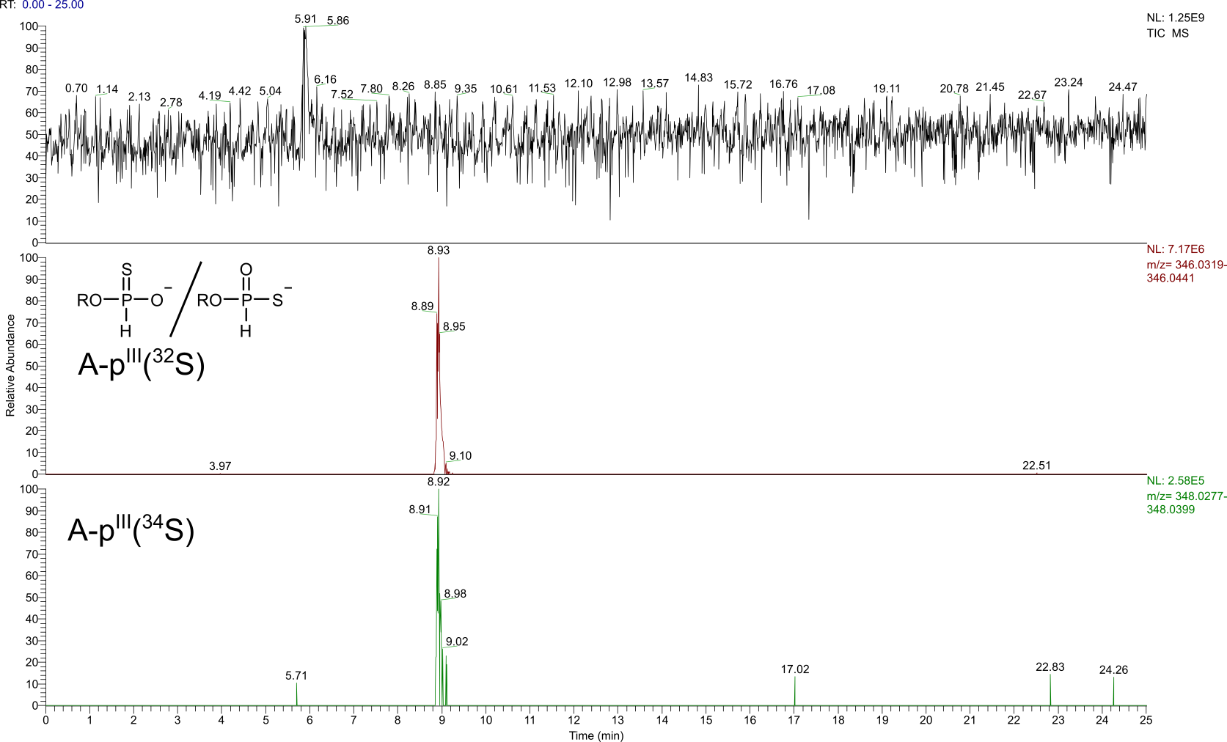


Supplementary Figure 3. TIC (m/z 50-750) and EIEs (Δ = 17.5 ppm) of the thiophosphonate product of the reaction starting from A (100 mM) and H_3_PO_3_ (1.0 eq.) after 7 d. Constitution of potential isomers has not been determined. The label illustrates all possible phosphate binding modes and refers to the entirety of all formed isomers.


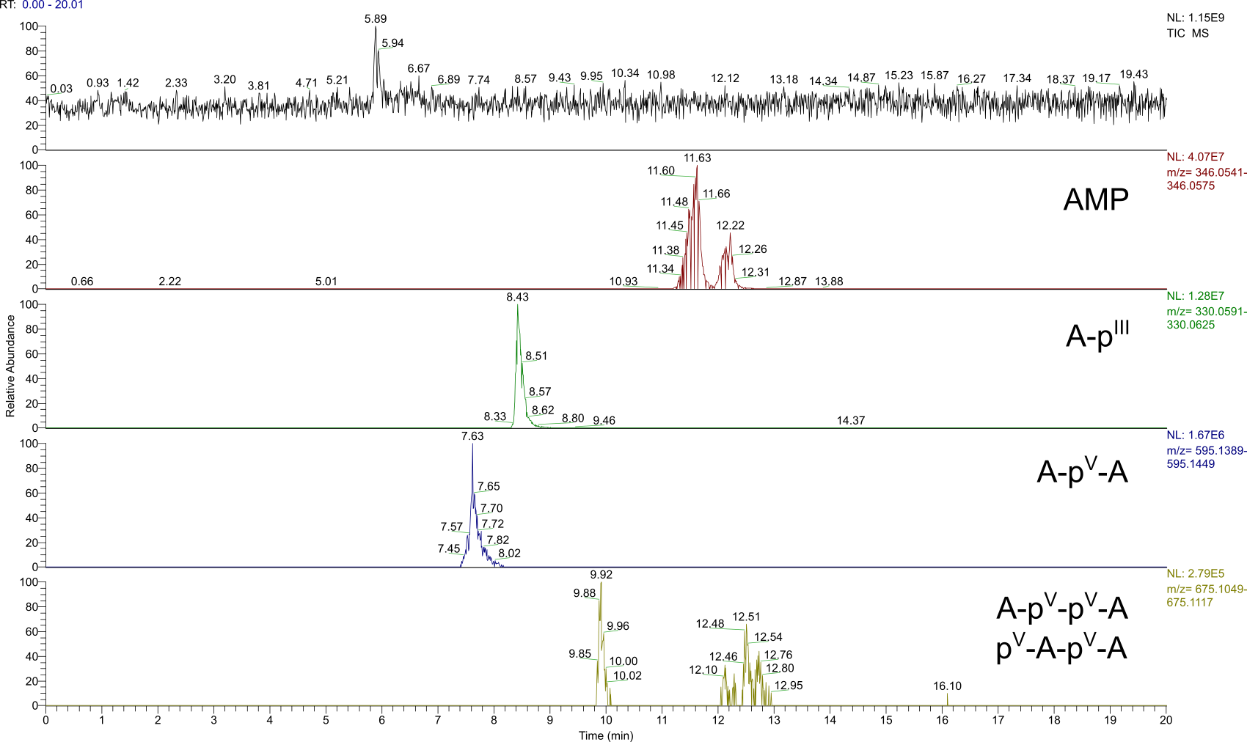


Supplementary Figure 4. TIC (m/z 50-750) and EIEs (Δ = 5.0 ppm) of the products of the reaction starting from A (100 mM) and H_3_PO_3_ (3.0 eq.) after 7 d. Constitution of potential isomers has not been determined. Labels illustrate all possible phosphate/phosphonate binding modes and refer to the entirety of all formed isomers.


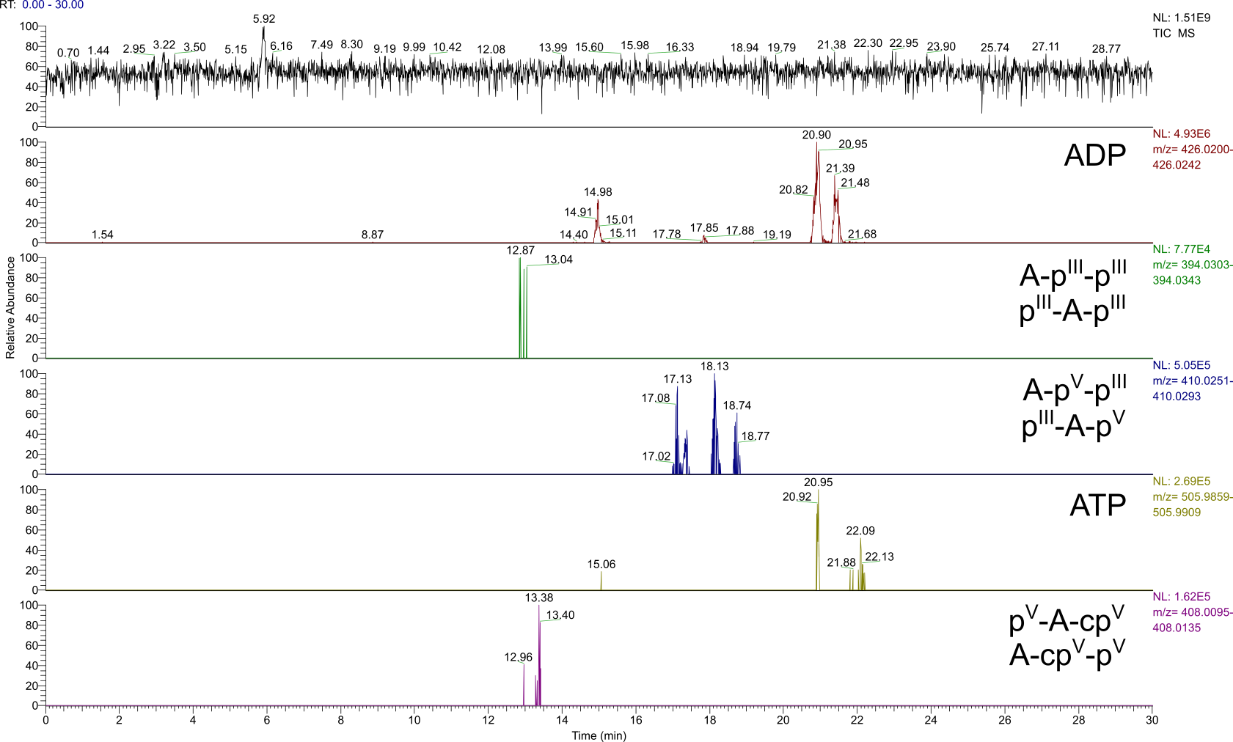


Supplementary Figure 5. TIC (m/z 50-750) and EIEs (Δ = 5.0 ppm) of the products of the reaction starting from A (100 mM) and H_3_PO_3_ (3.0 eq.) after 7 d. Constitution of potential isomers has not been determined. Labels illustrate all possible phosphate/phosphonate binding modes and refer to the entirety of all formed isomers.


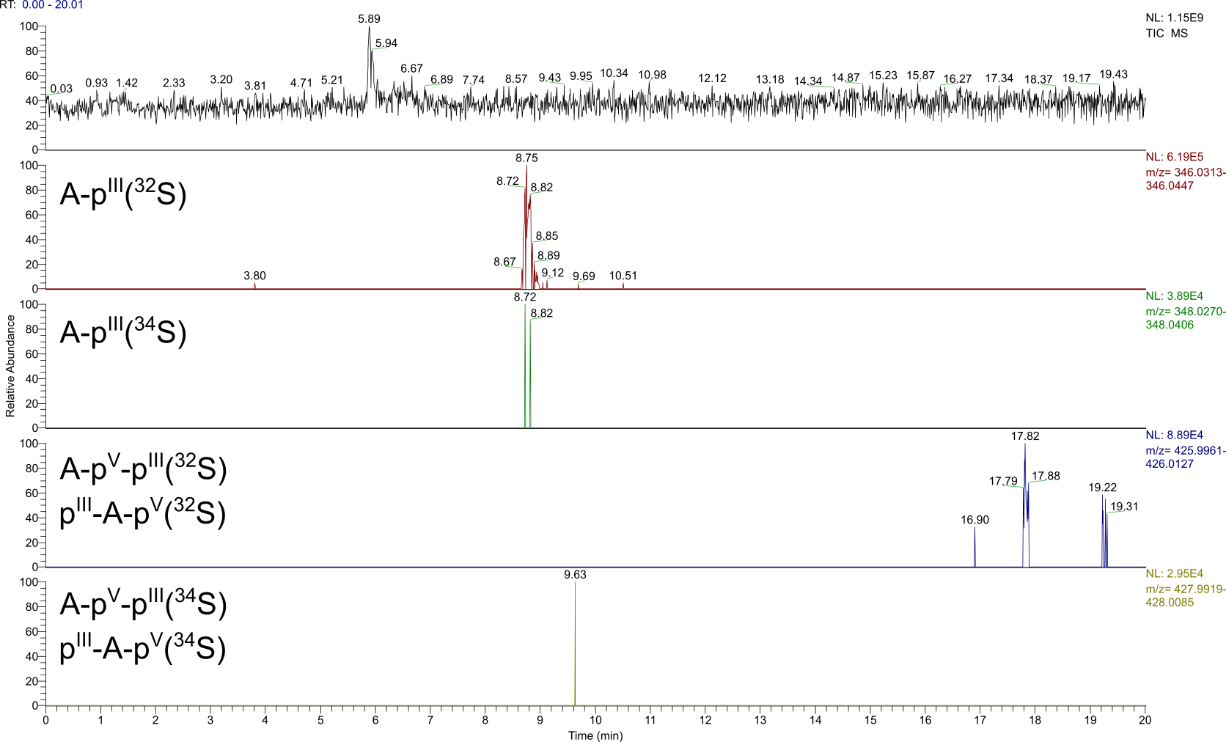


Supplementary Figure 6. TIC (m/z 50-750) and EIEs (Δ = 19.5 ppm) of the thiophosphonate/-phosphate products of the reaction starting from A (100 mM) and H_3_PO_3_ (3.0 eq.) after 7 d. Constitution of potential isomers has not been determined. Labels illustrate all possible phosphate/phosphonate binding modes and refer to the entirety of all formed isomers.


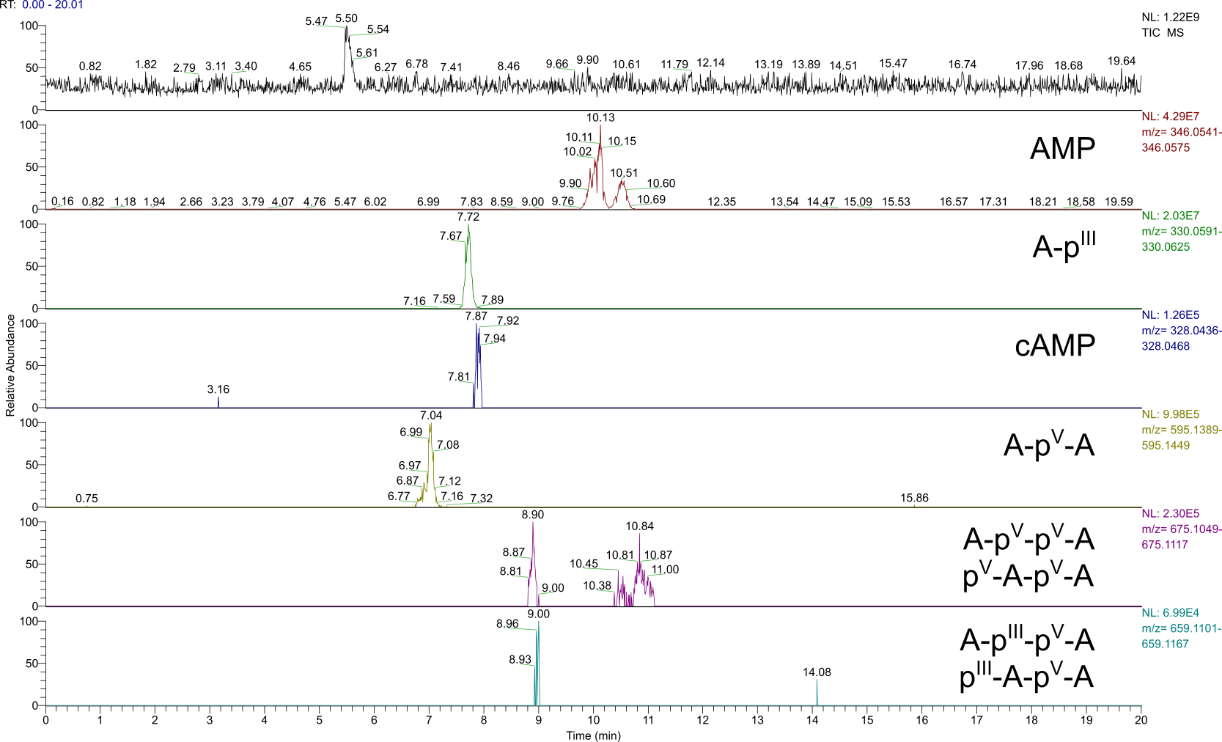


Supplementary Figure 7. TIC (m/z 50-750) and EIEs (Δ = 5.0 ppm) of the products of the reaction starting from A (100 mM) and H_3_PO_3_ (5.0 eq.) after 7d. Constitution of potential isomers has not been determined. Labels illustrate all possible phosphate/phosphonate binding modes and refer to the entirety of all formed isomers.


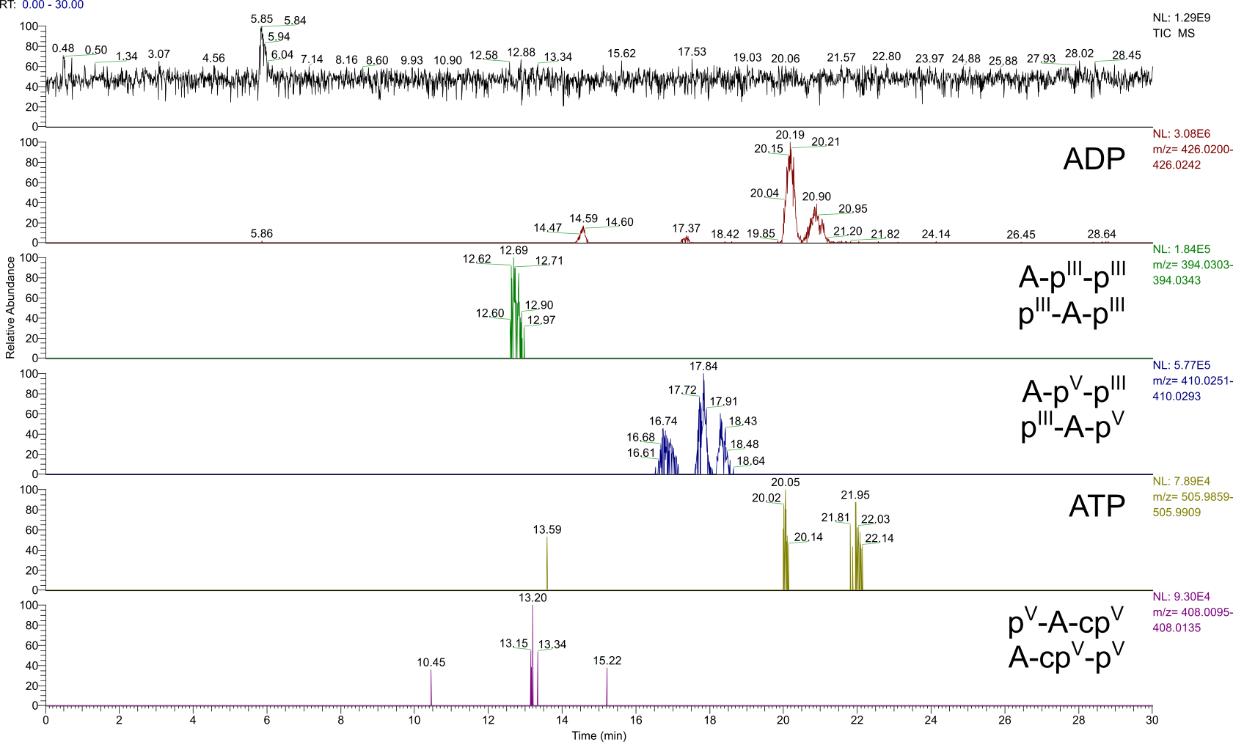


Supplementary Figure 8. TIC (m/z 50-750) and EIEs (Δ = 5.0 ppm) of the products of the reaction starting from A (100 mM) and H_3_PO_3_ (5.0 eq.) after 7 d. Constitution of potential isomers has not been determined. Labels illustrate all possible phosphate/phosphonate binding modes and refer to the entirety of all formed isomers.


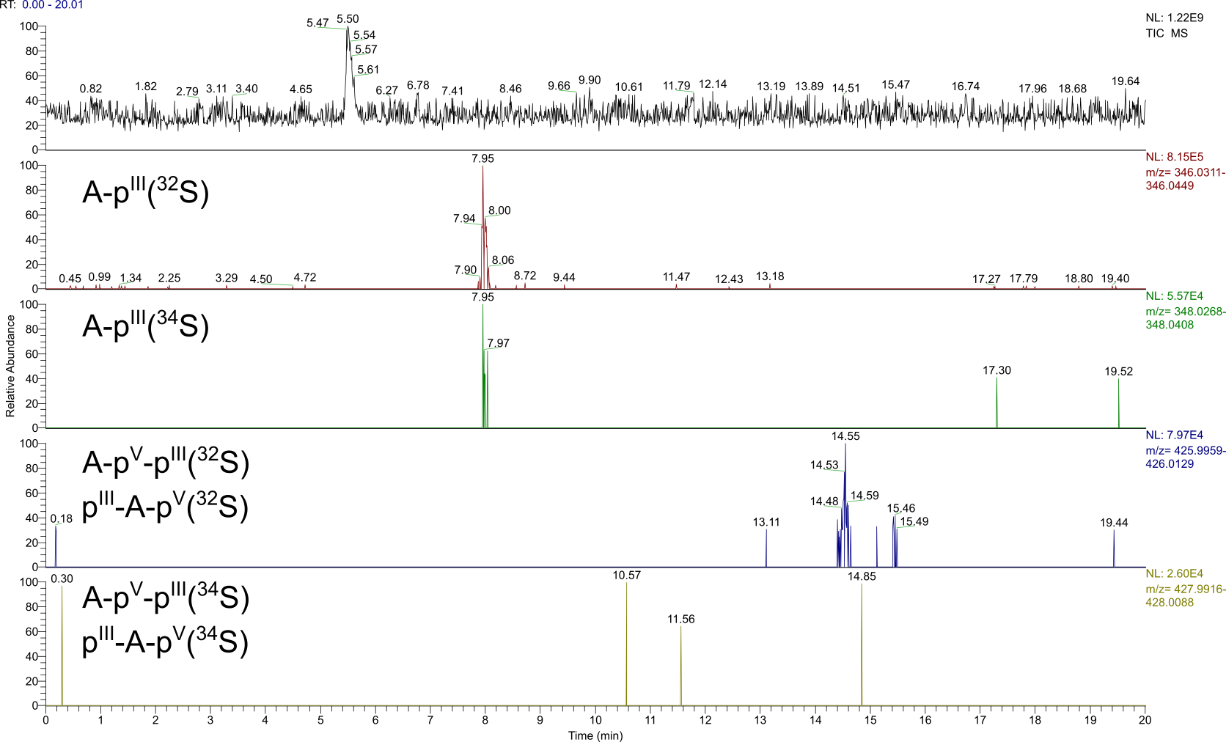


Supplementary Figure 9. TIC (m/z 50-750) and EIEs (Δ = 20.0 ppm) of the thiophosphonate/-phosphate products of the reaction starting from A (100 mM) and H_3_PO_3_ (5.0 eq.) after 7 d. Constitution of potential isomers has not been determined. Labels illustrate all possible phosphate/phosphonate binding modes and refer to the entirety of all formed isomers.

#### Variation of the urea concentration


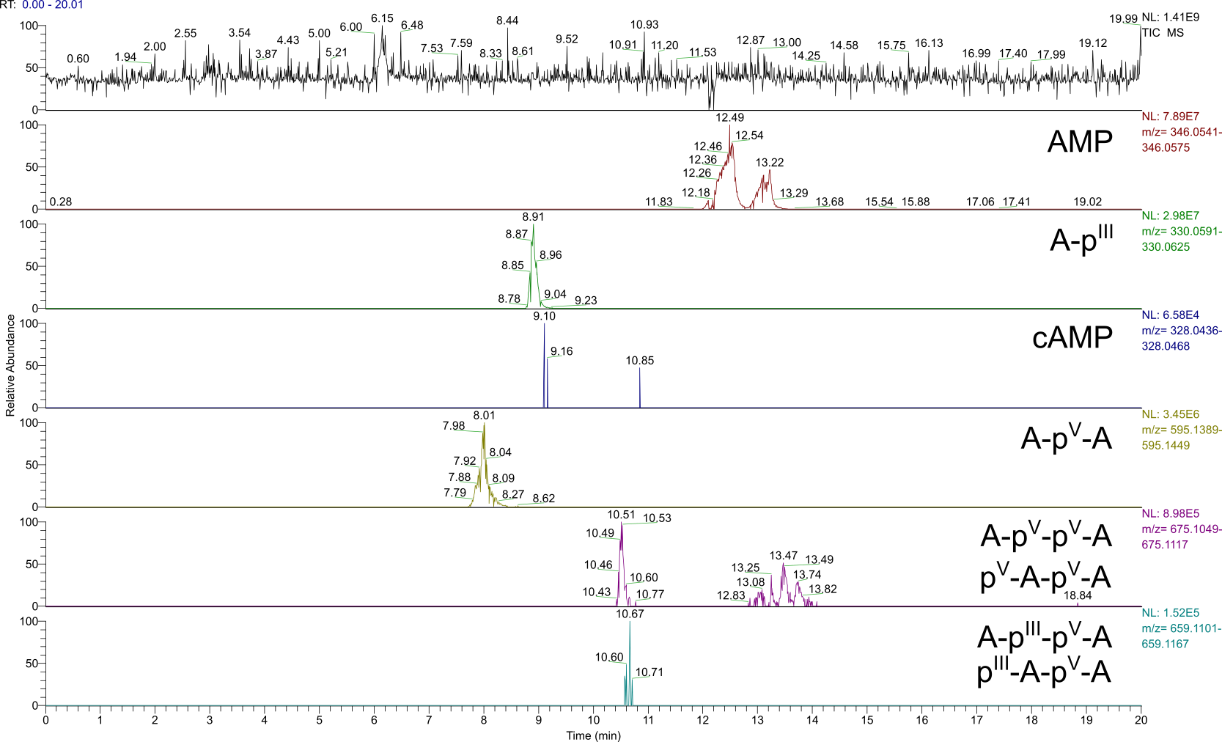


Supplementary Figure 10. TIC (m/z 50-750) and EIEs (Δ = 5.0 ppm) of the products of the reaction starting from A (100 mM), H_3_PO_3_ (3.0 eq.) and urea (1.0 eq.) after 7 d. Constitution of potential isomers has not been determined. Labels illustrate all possible phosphate/phosphonate binding modes and refer to the entirety of all formed isomers.


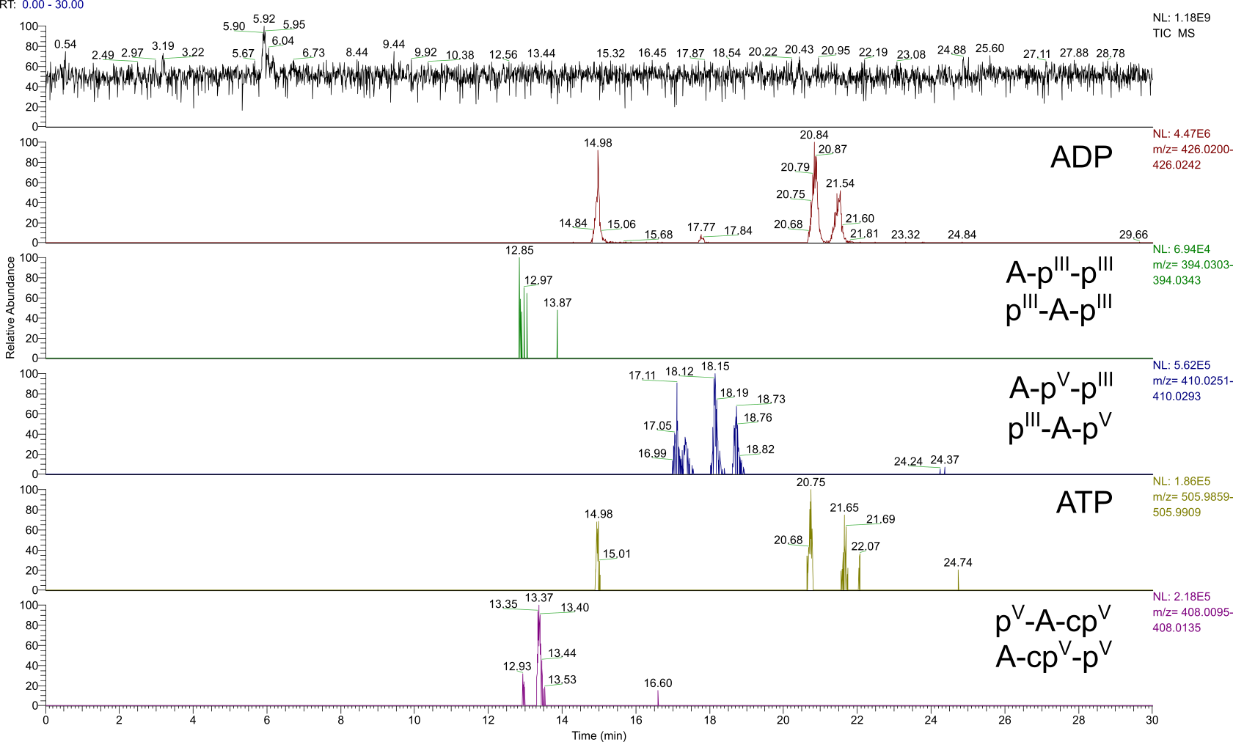


Supplementary Figure 11. TIC (m/z 50-750) and EIEs (Δ = 5.0 ppm) of the products of the reaction starting from A (100 mM), H_3_PO_3_ (3.0 eq.) and urea (1.0 eq.) after 7 d. Constitution of potential isomers has not been determined. Labels illustrate all possible phosphate/phosphonate binding modes and refer to the entirety of all formed isomers.


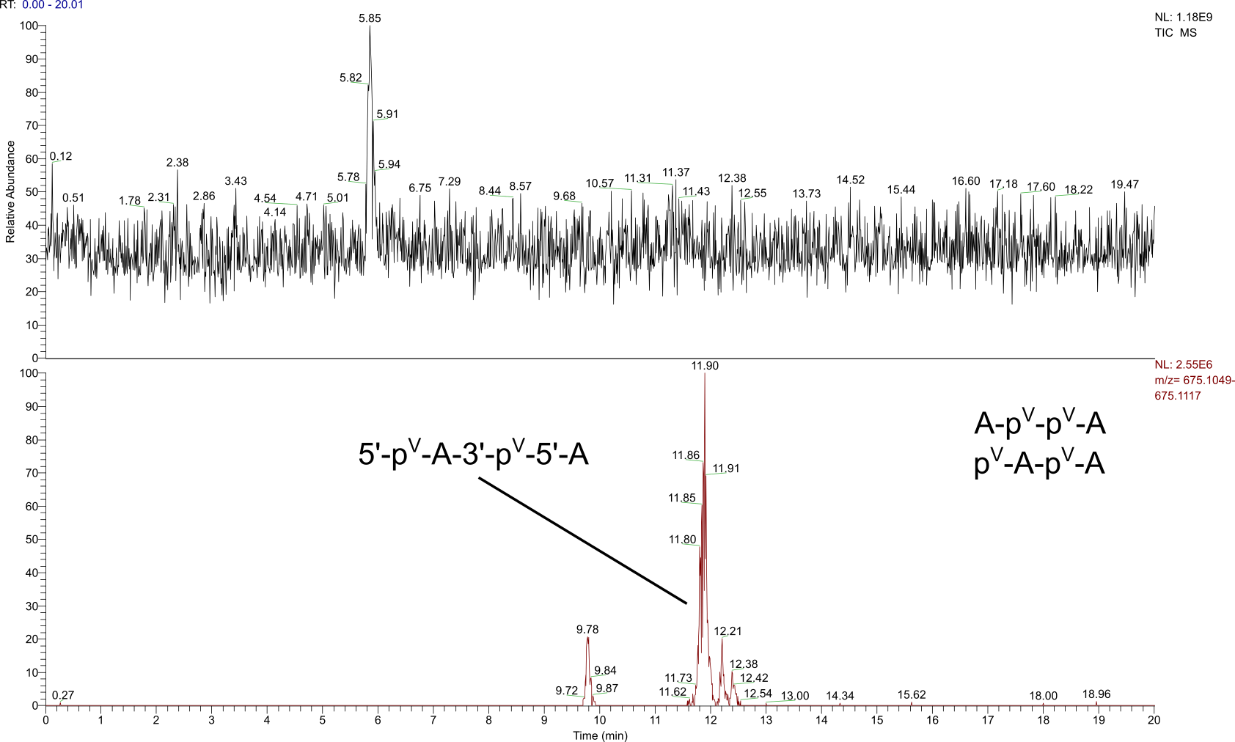


Supplementary Figure 12. TIC (m/z 50-750) and EIE (Δ = 5.0 ppm) of the product mixture from the reaction of A (100 mM), H_3_PO_3_ (3.0 eq.) and urea (1.0 eq.) after 7 d with co-injected 5’-p^V^-A-3’-p^V^-5’-A (50 µM). The constitution of the other potential isomers has not been determined. Labels illustrate all possible phosphate/phosphonate binding modes and refer to the entirety of all formed isomers.


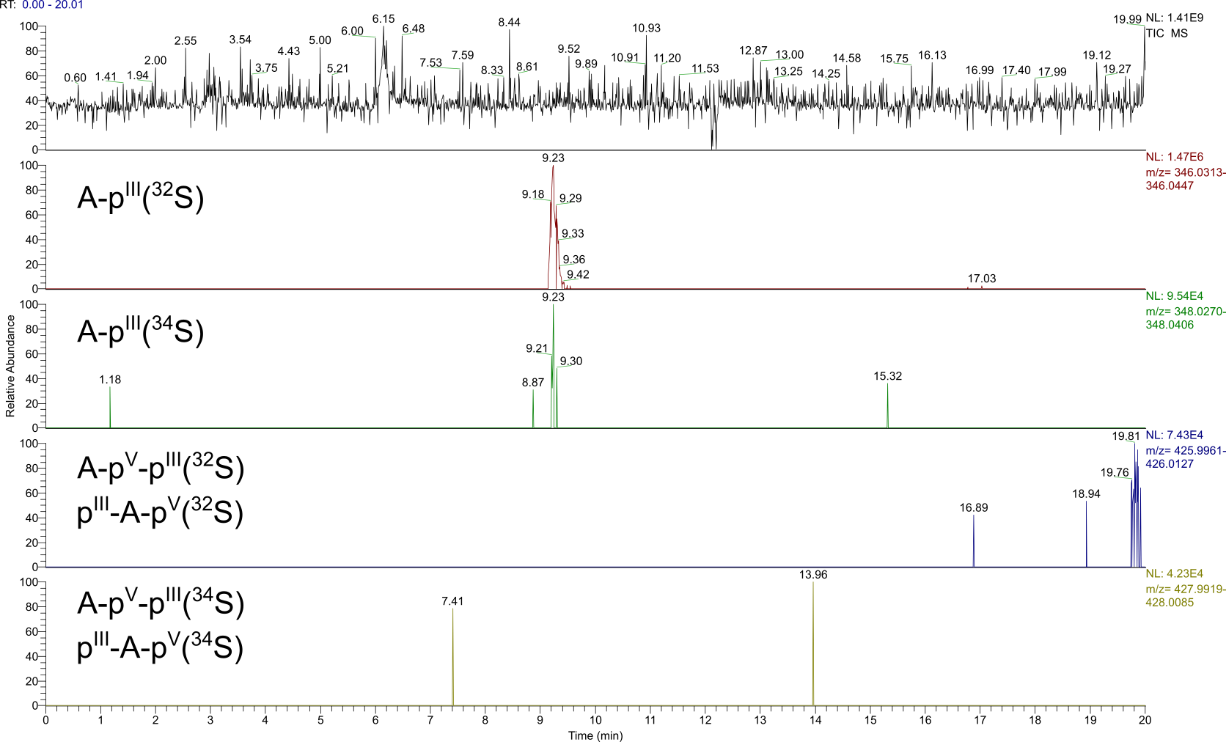


Supplementary Figure 13. TIC (m/z 50-750) and EIEs (Δ = 19.5 ppm) of the thiophosphonate/-phosphate products of the reaction starting from A (100 mM), H_3_PO_3_ (3.0 eq.) and urea (1.0 eq.) after 7 d. Constitution of potential isomers has not been determined. Labels illustrate all possible phosphate/phosphonate binding modes and refer to the entirety of all formed isomers.


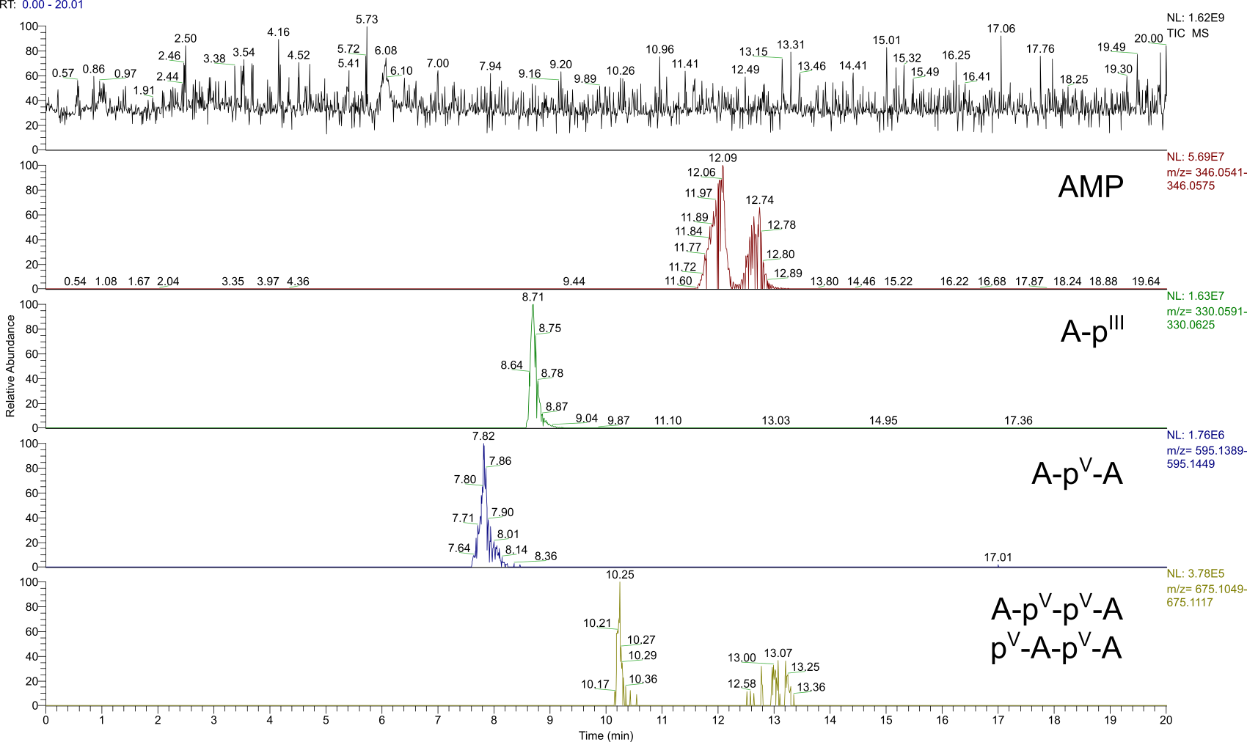


Supplementary Figure 14. TIC (m/z 50-750) and EIEs (Δ = 5.0 ppm) of the products of the reaction starting from A (100 mM), H_3_PO_3_ (3.0 eq.) and urea (3.0 eq.) after 7 d. Constitution of potential isomers has not been determined. Labels illustrate all possible phosphate/phosphonate binding modes and refer to the entirety of all formed isomers.


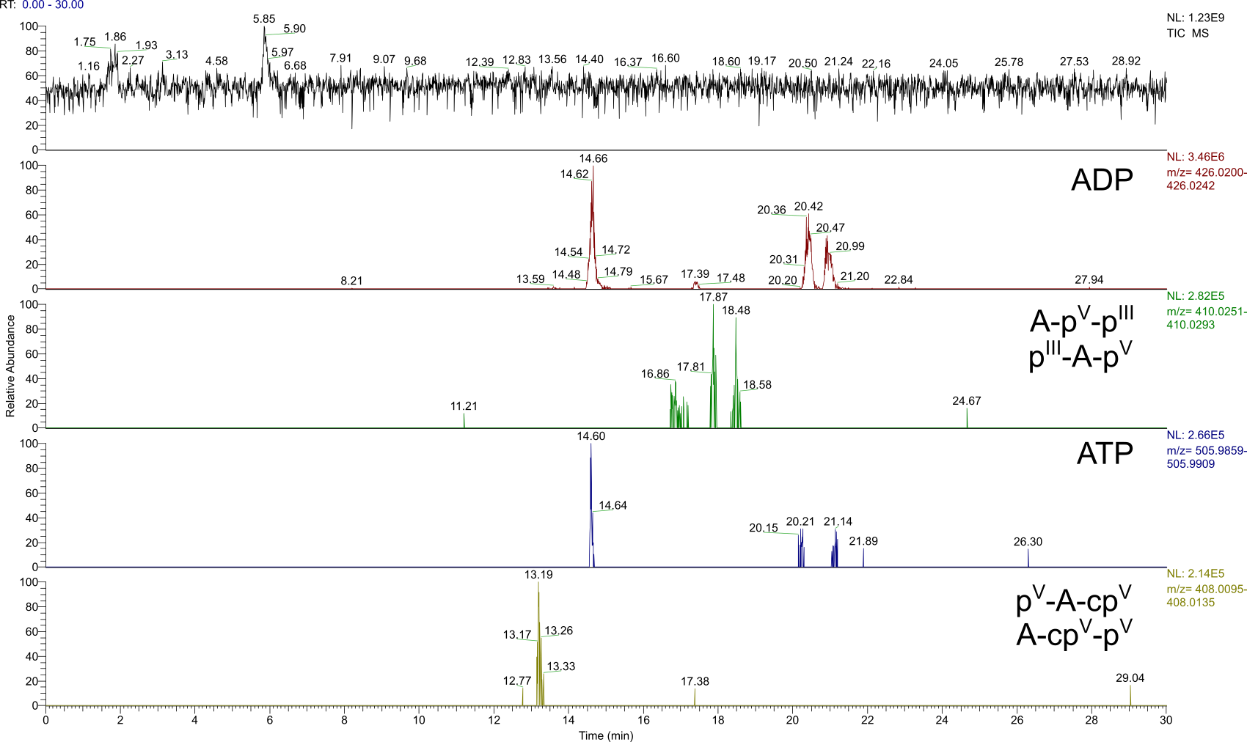


Supplementary Figure 15. TIC (m/z 50-750) and EIEs (Δ = 5.0 ppm) of the products of the reaction starting from A (100 mM), H_3_PO_3_ (3.0 eq.) and urea (3.0 eq.) after 7 d. Constitution of potential isomers has not been determined. Labels illustrate all possible phosphate/phosphonate binding modes and refer to the entirety of all formed isomers.


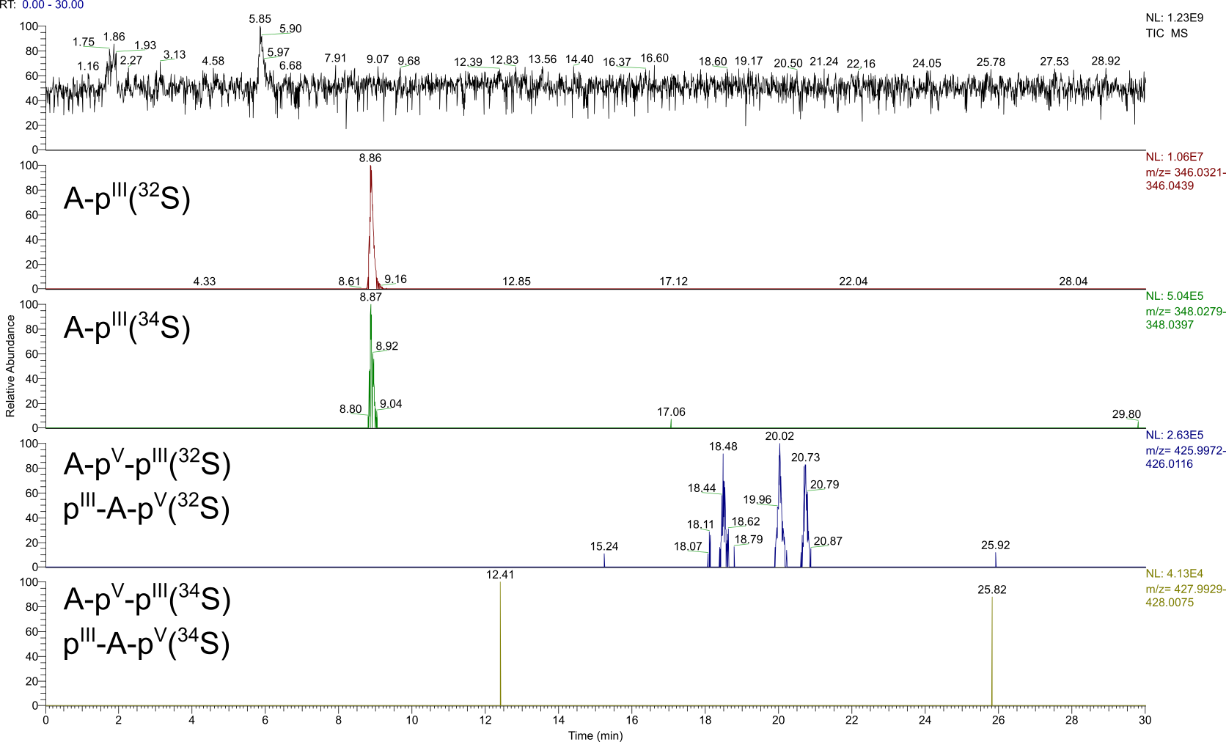


Supplementary Figure 16. TIC (m/z 50-750) and EIEs (Δ = 17.0 ppm) of the thiophosphonate/-phosphate products of the reaction starting from A (100 mM), H_3_PO_3_ (3.0 eq.) and urea (3.0 eq.) after 7 d. Constitution of potential isomers has not been determined. Labels illustrate all possible phosphate/phosphonate binding modes and refer to the entirety of all formed isomers.

#### Reaction in water


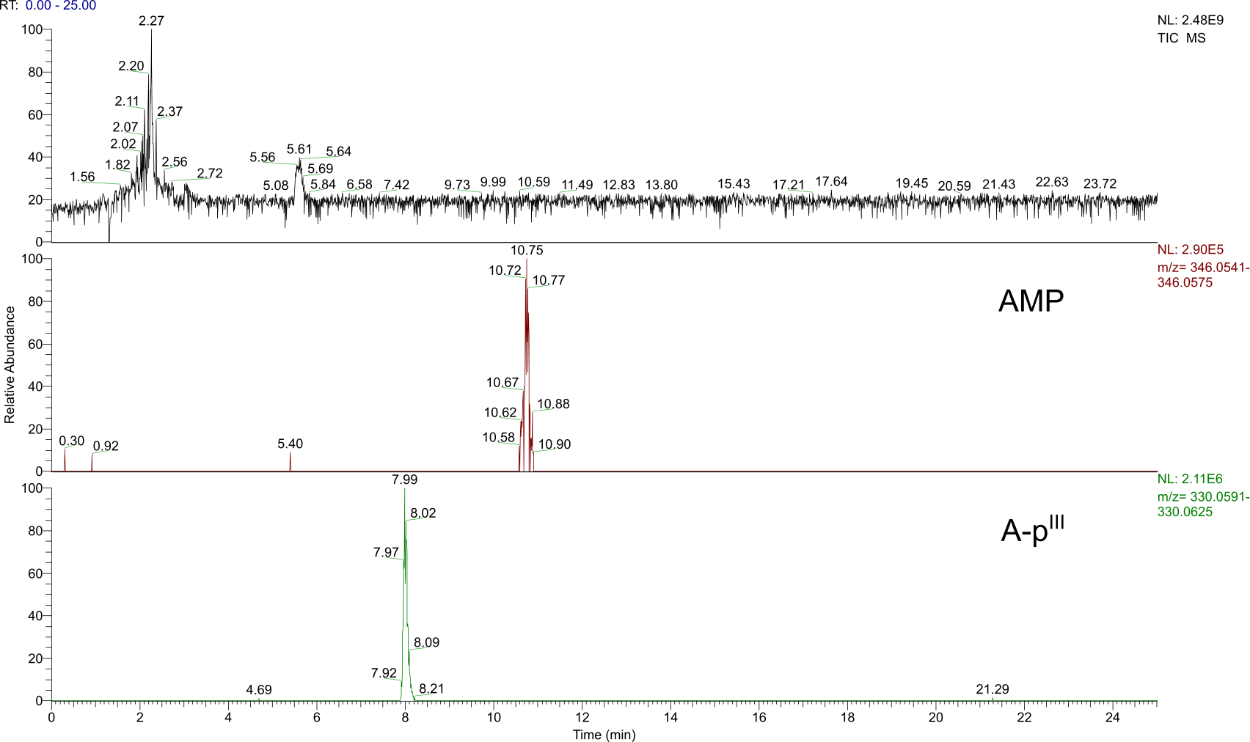


Supplementary Figure 17. TIC (m/z 50-750) and EIEs (Δ = 5.0 ppm) of the products of the reaction starting from A (100 mM), H_3_PO_3_ (3.0 eq.) and urea (1.0 eq.) after 7 d in water under anoxic conditions. Constitution of potential isomers has not been determined. Labels illustrate all possible phosphate/phosphonate binding modes and refer to the entirety of all formed isomers.

#### Variation of the adenosine concentration


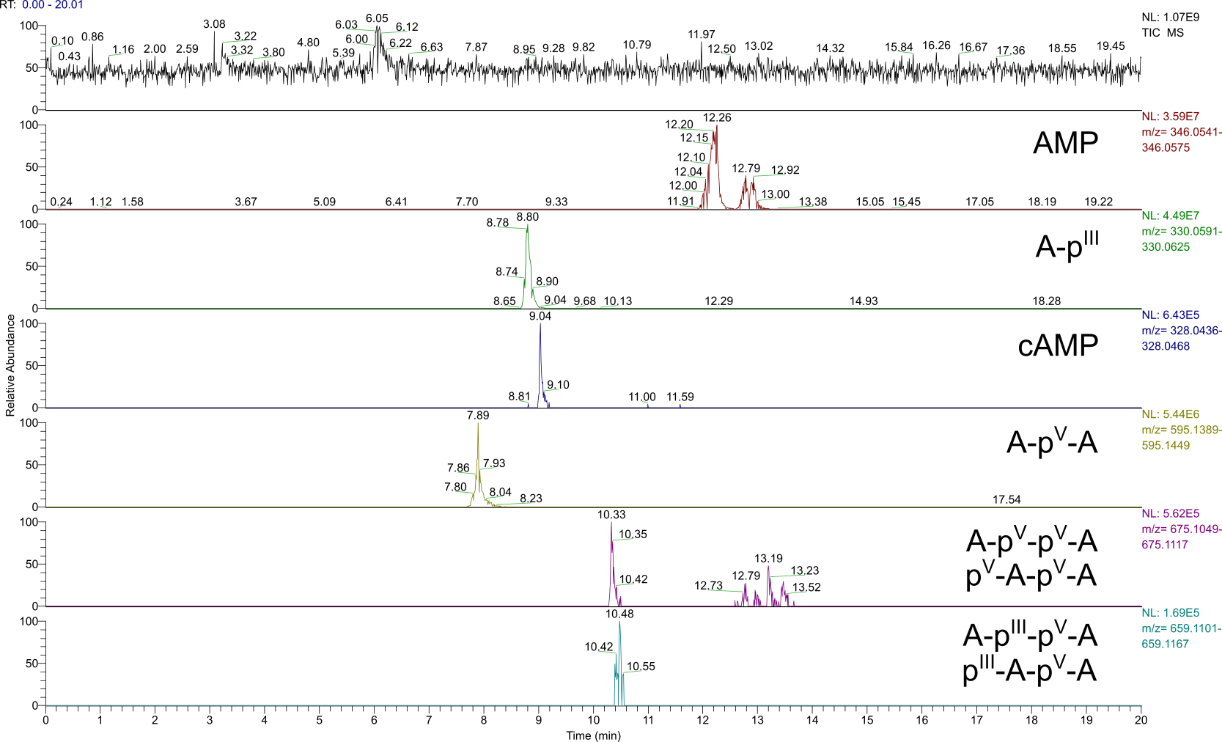


Supplementary Figure 18. TIC (m/z 50-750) and EIEs (Δ = 5.0 ppm) of the products of the reaction starting from A (25 mM) and H_3_PO_3_ (1.0 eq.) after 7 d. Constitution of potential isomers has not been determined. Labels illustrate all possible phosphate/phosphonate binding modes and refer to the entirety of all formed isomers.


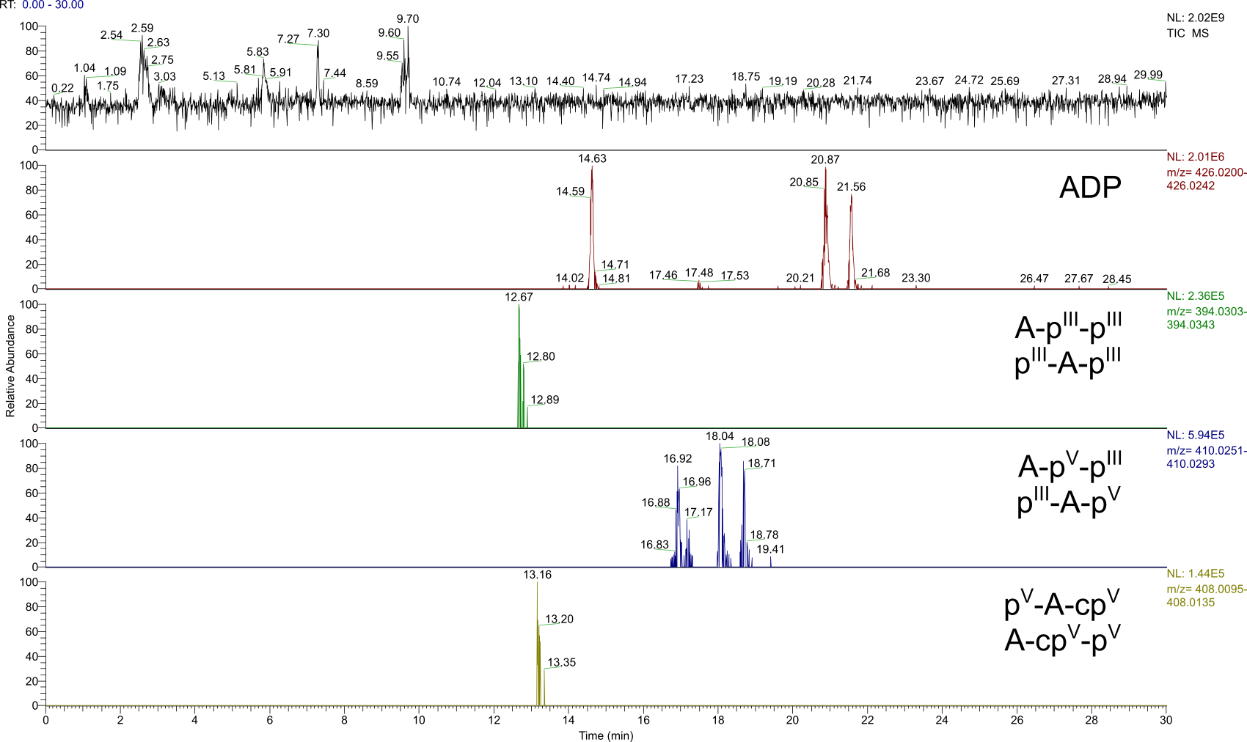


Supplementary Figure 19. TIC (m/z 50-750) and EIEs (Δ = 5.0 ppm) of the products of the reaction starting from A (25 mM) and H_3_PO_3_ (1.0 eq.) after 7 d. Constitution of potential isomers has not been determined. Labels illustrate all possible phosphate/phosphonate binding modes and refer to the entirety of all formed isomers.


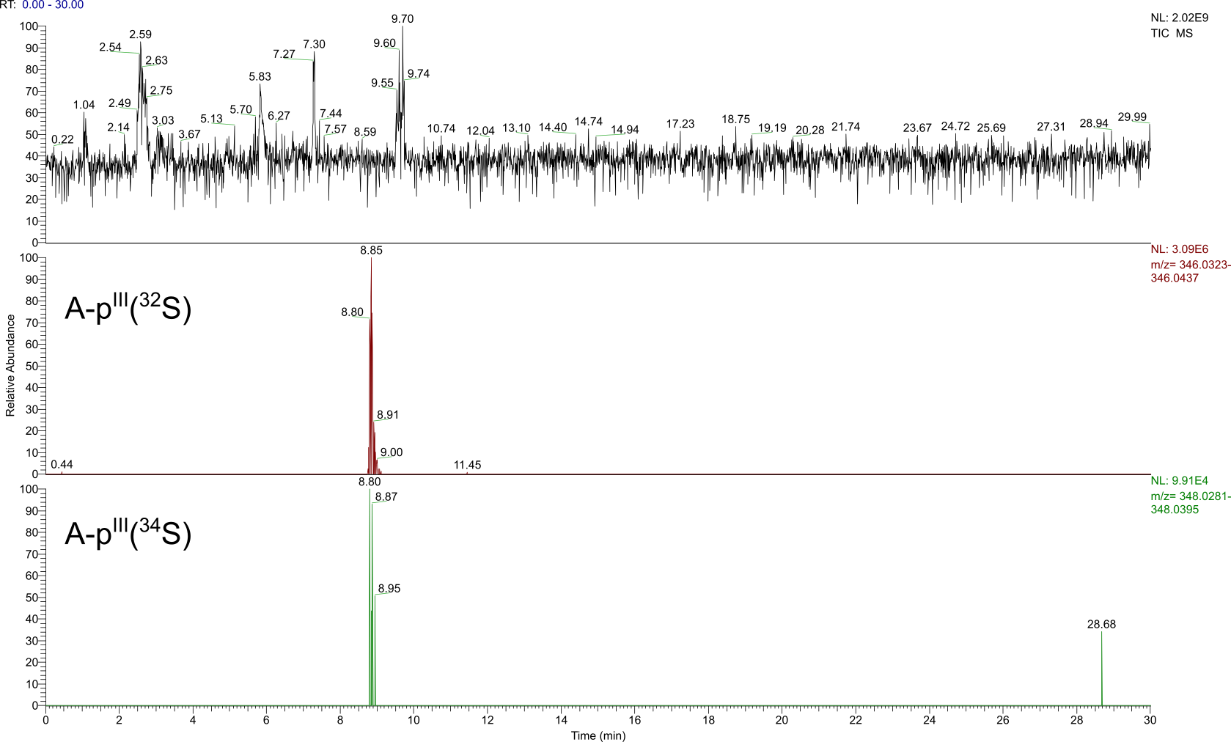


Supplementary Figure 20. TIC (m/z 50-750) and EIEs (Δ = 16.5 ppm) of the thiophosphonate product of the reaction starting from A (25 mM) and H_3_PO_3_ (1.0 eq.) after 7 d. Constitution of potential isomers has not been determined. Labels illustrate all possible phosphate/phosphonate binding modes and refer to the entirety of all formed isomers.


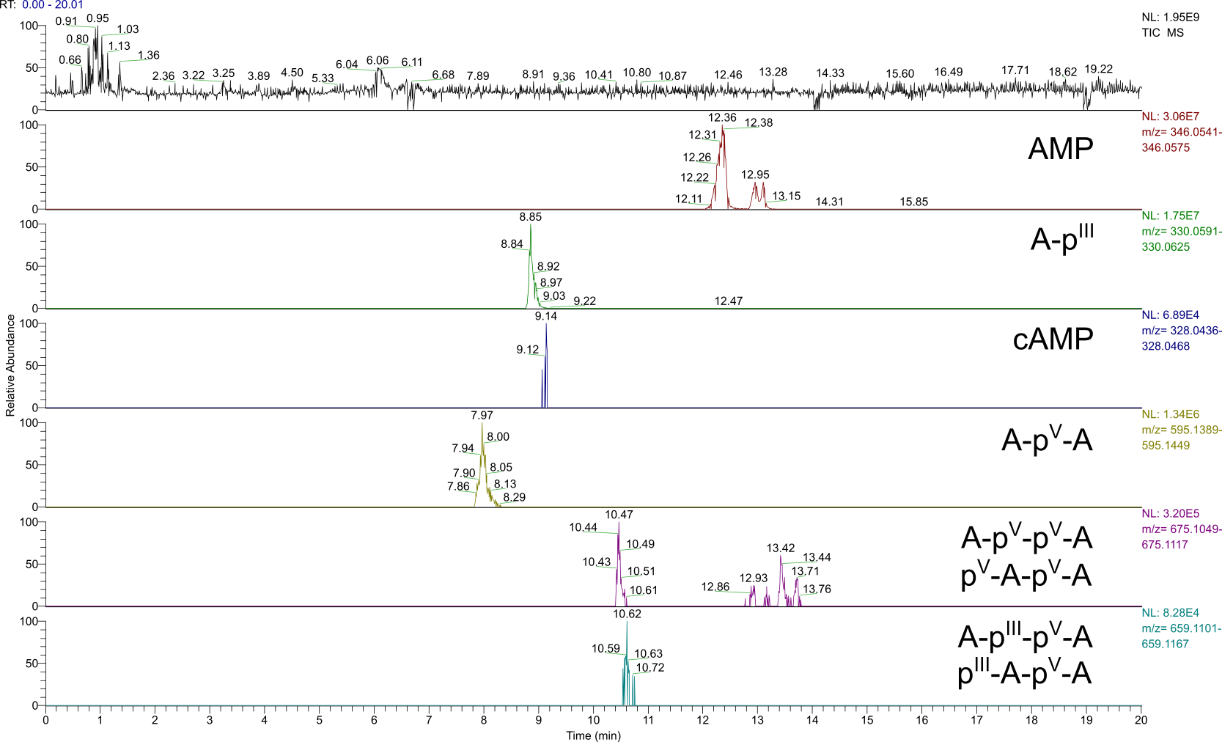


Supplementary Figure 21. TIC (m/z 50-750) and EIEs (Δ = 5.0 ppm) of the products of the reaction starting from A (50 mM) and H_3_PO_3_ (1.0 eq.) after 7 d. Constitution of potential isomers has not been determined. Labels illustrate all possible phosphate/phosphonate binding modes and refer to the entirety of all formed isomers.


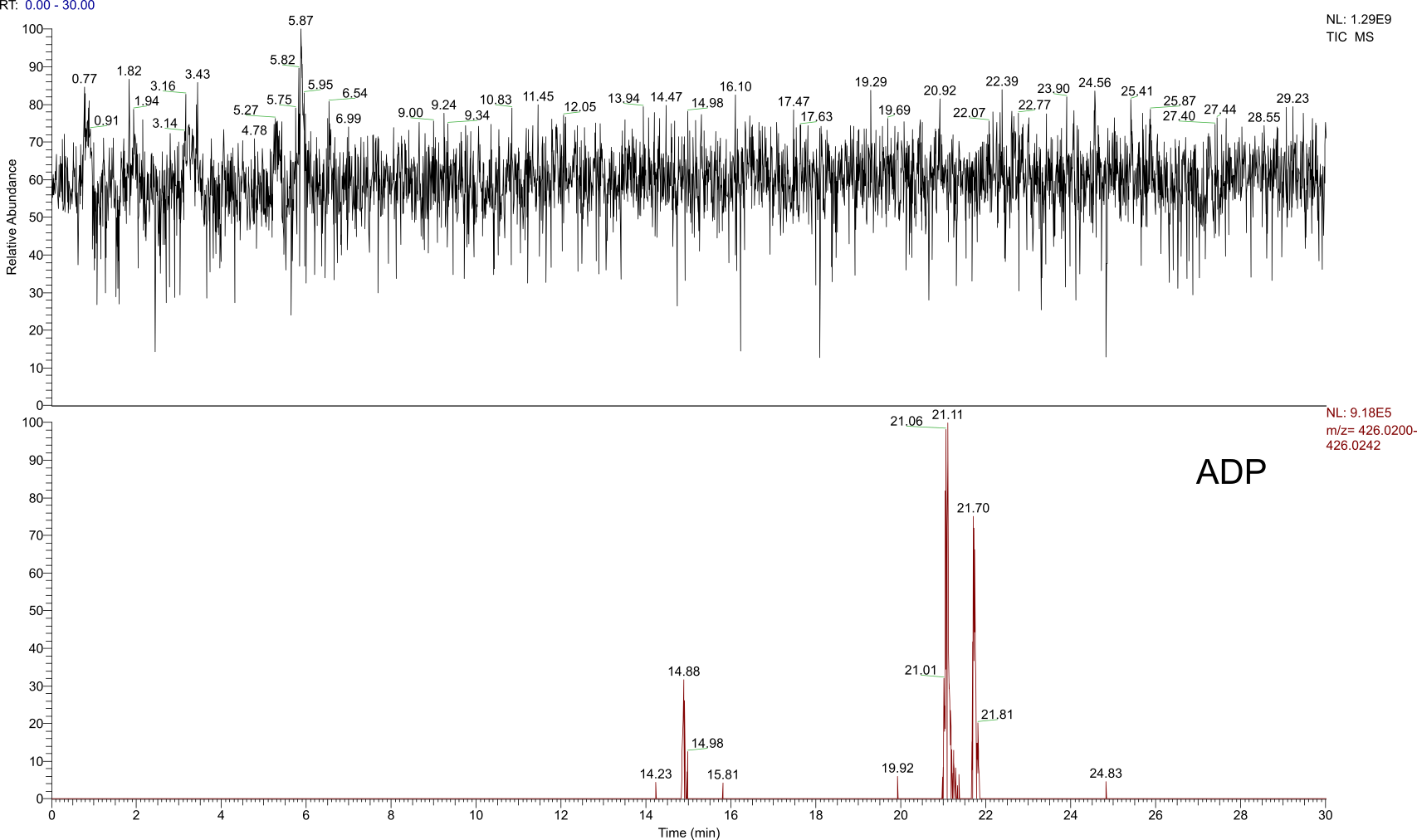


Supplementary Figure 22. TIC (m/z 50-750) and EIE (Δ = 5.0 ppm) of the products of the reaction starting from A (50 mM) and H_3_PO_3_ (1.0 eq.) after 7 d. Constitution of potential isomers has not been determined. The label illustrates all possible phosphate binding modes and refers to the entirety of all formed isomers.


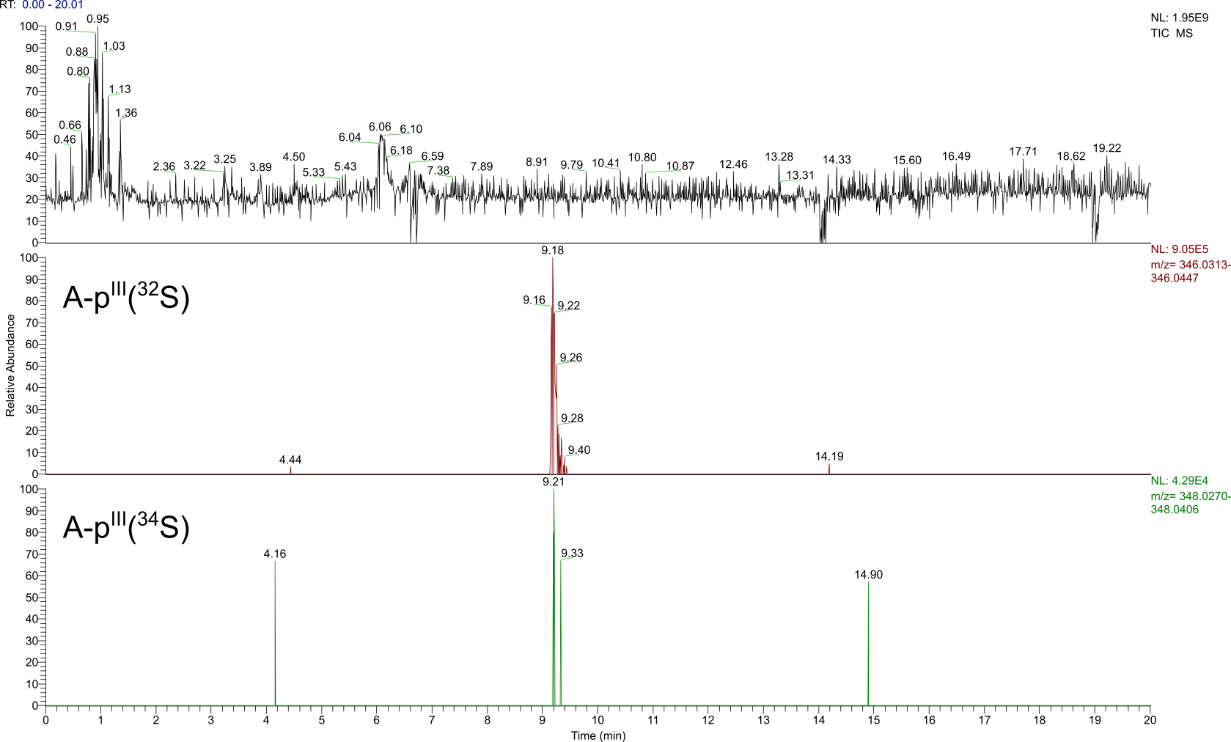


Supplementary Figure 23. TIC (m/z 50-750) and EIEs (Δ = 19.5 ppm) of the thiophosphonate product of the reaction starting from A (50 mM) and H_3_PO_3_ (1.0 eq.) after 7 d. Constitution of potential isomers has not been determined. Labels illustrate all possible phosphate/phosphonate binding modes and refer to the entirety of all formed isomers.


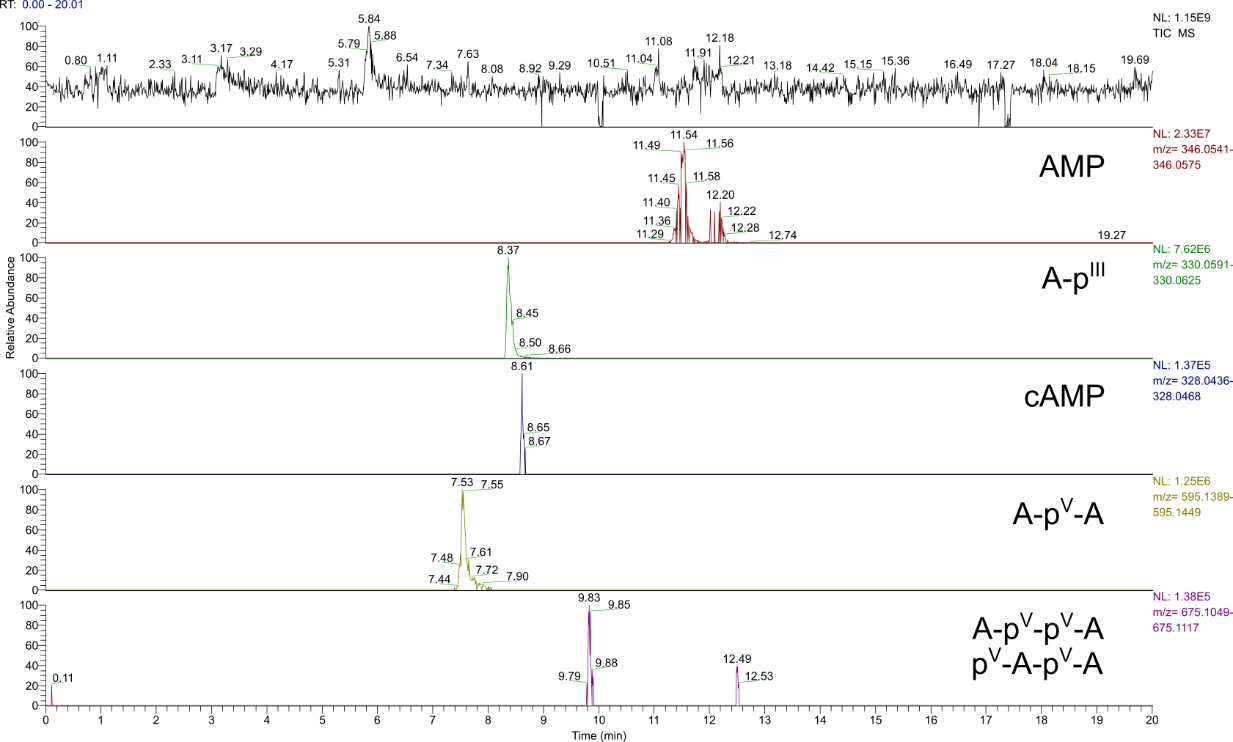


Supplementary Figure 24. TIC (m/z 50-750) and EIEs (Δ = 5.0 ppm) of the products of the reaction starting from A (400 mM) and H_3_PO_3_ (1.0 eq.) after 7 d. Constitution of potential isomers has not been determined. Labels illustrate all possible phosphate/phosphonate binding modes and refer to the entirety of all formed isomers.


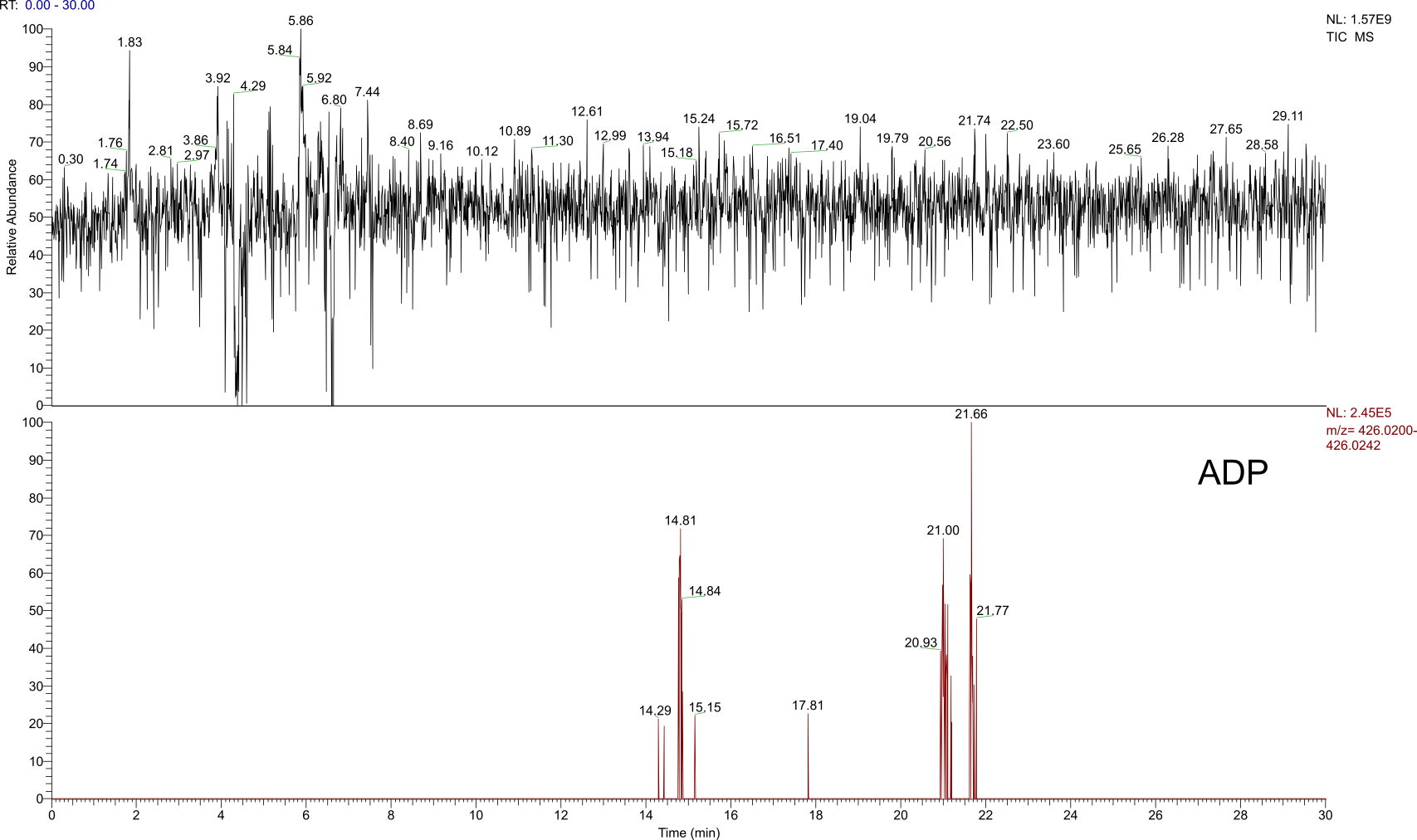


Supplementary Figure 25. TIC (m/z 50-750) and EIE (Δ = 5.0 ppm) of the products of the reaction starting from A (400 mM) and H_3_PO_3_ (1.0 eq.) after 7 d. Constitution of potential isomers has not been determined. The label illustrates all possible phosphate binding modes and refers to the entirety of all formed isomers.


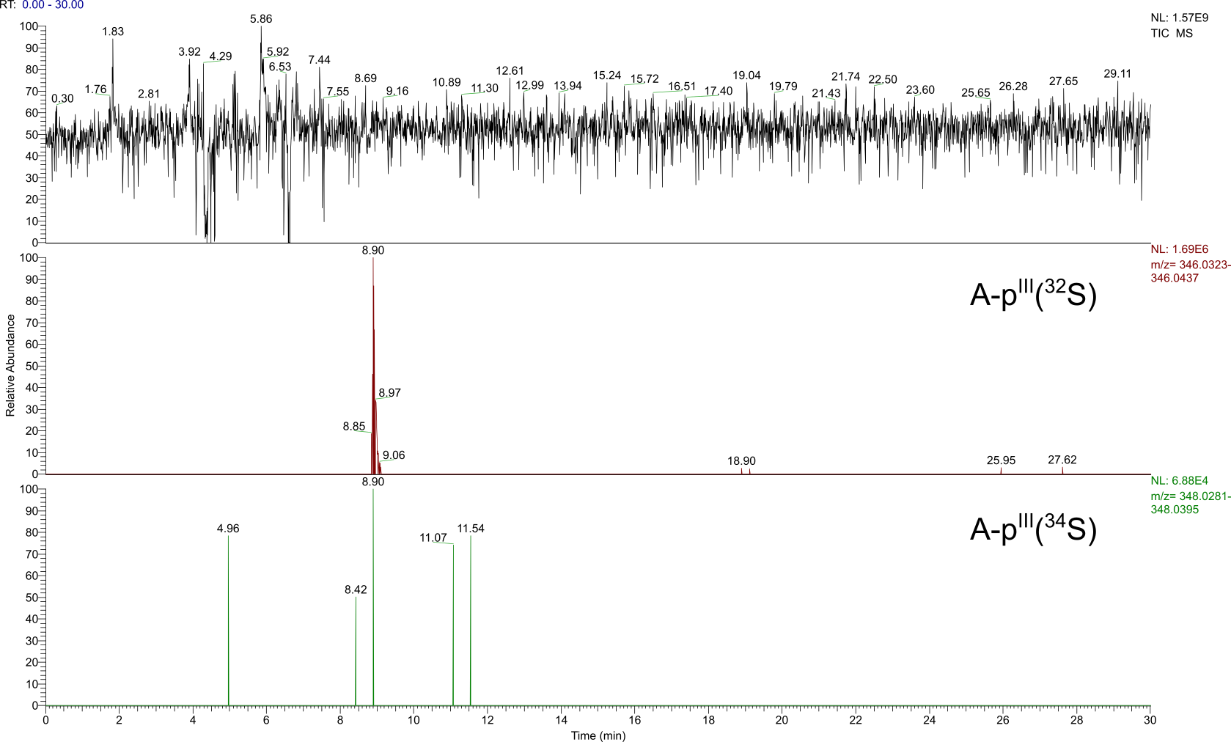


Supplementary Figure 26. TIC (m/z 50-750) and EIE (Δ = 16.5 ppm) of the thiophosphonate product of the reaction starting from A (400 mM) and H_3_PO_3_ (1.0 eq.) after 7 d. Constitution of potential isomers has not been determined. The label illustrates all possible phosphate binding modes and refers to the entirety of all formed isomers.

#### Time dependence of the reaction


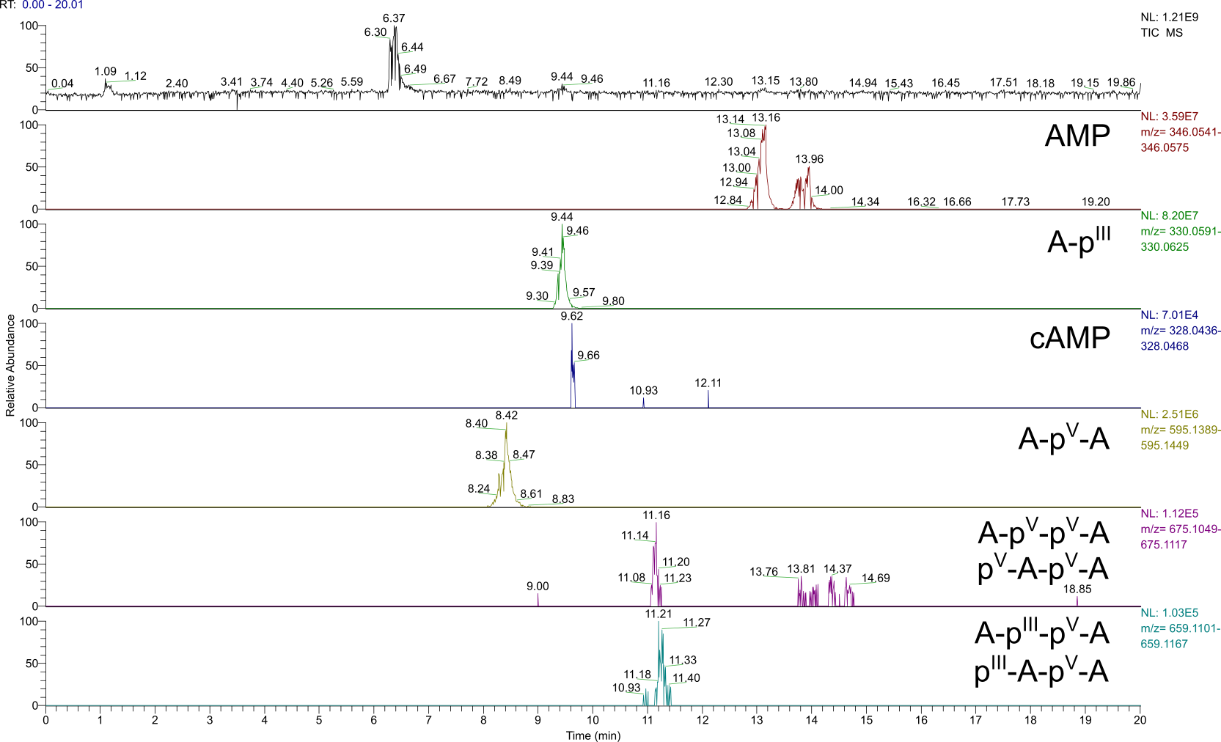


Supplementary Figure 27. TIC (m/z 80-1200) and EIEs (Δ = 5.0 ppm) of the products of the reaction starting from A (100 mM), H_3_PO_3_ (3.0 eq.) and urea (1.0 eq.) after 1 d. Constitution of potential isomers has not been determined. Labels illustrate all possible phosphate/phosphonate binding modes and refer to the entirety of all formed isomers.


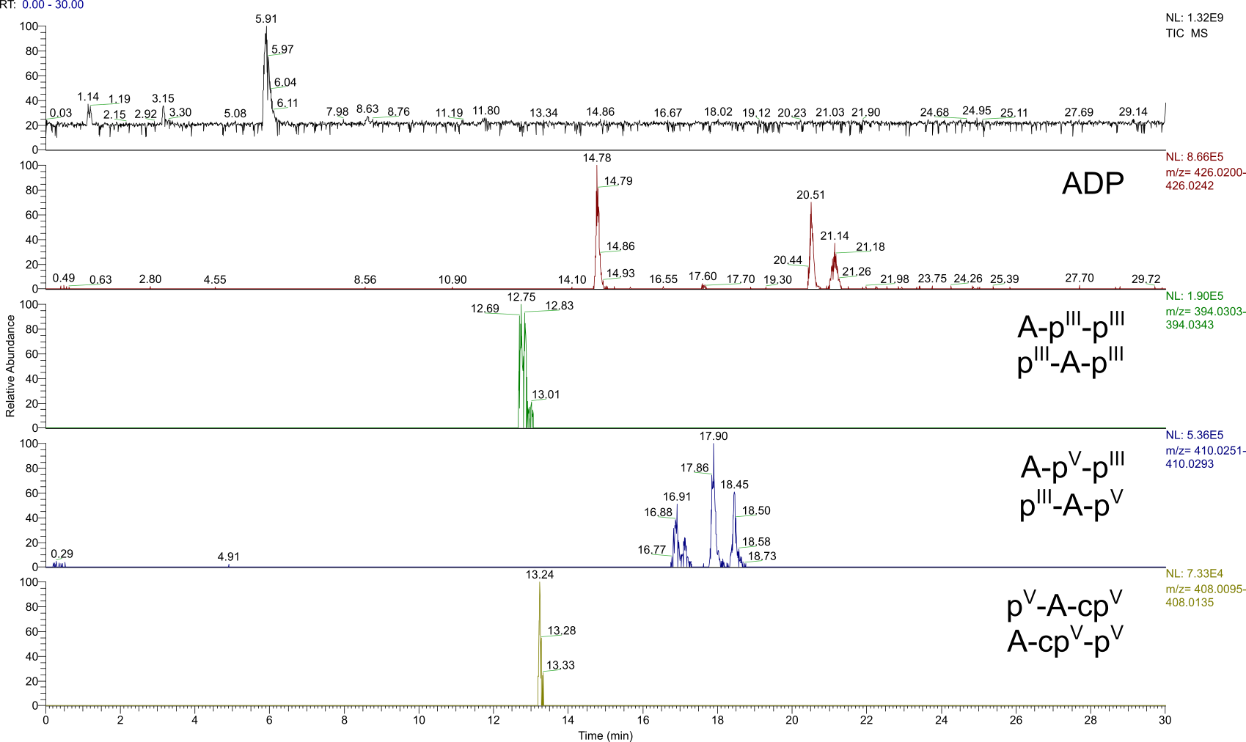


Supplementary Figure 28. TIC (m/z 80-1200) and EIEs (Δ = 5.0 ppm) of the products of the reactions starting from A (100 mM), H_3_PO_3_ (3.0 eq.) and urea (1.0 eq.) after 1 d. Constitution of potential isomers has not been determined. Labels illustrate all possible phosphate/phosphonate binding modes and refer to the entirety of all formed isomers.


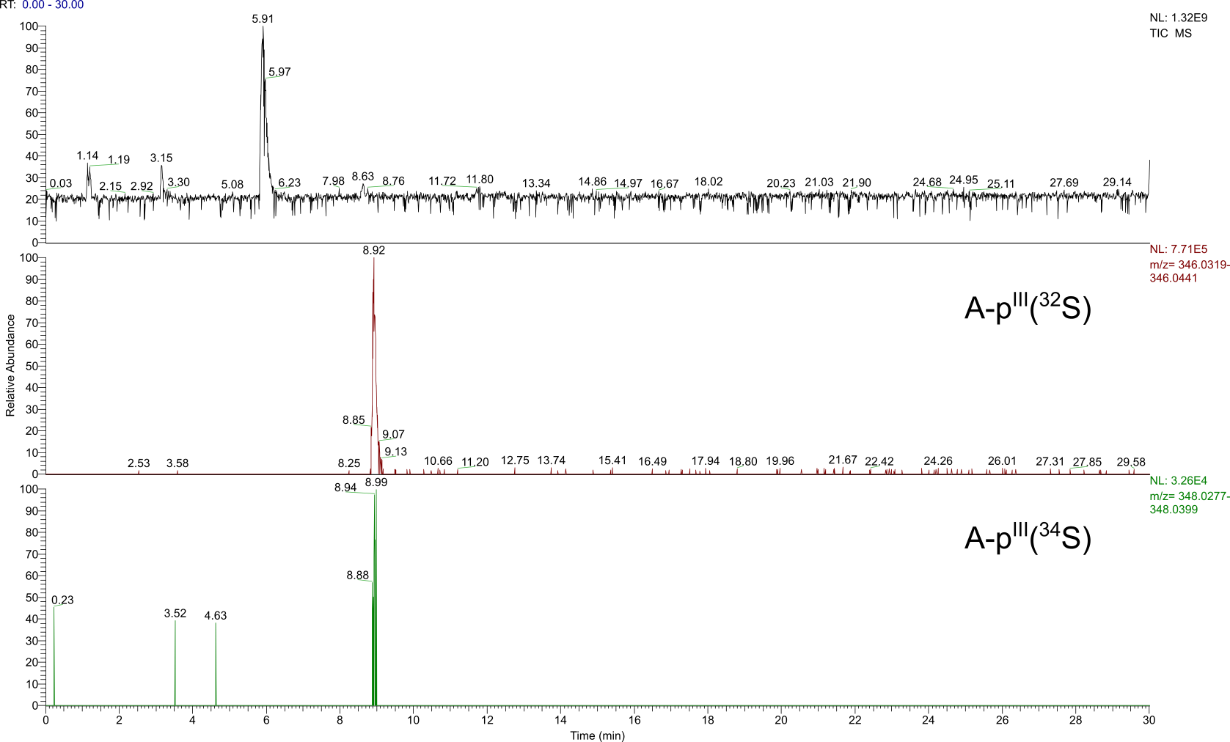


Supplementary Figure 29. TIC (m/z 80-1200) and EIEs (Δ = 17.5 ppm) of the thiophosphonate product of the reactions starting from A (100 mM), H_3_PO_3_ (3.0 eq.) and urea (1.0 eq.) after 1 d. Constitution of potential isomers has not been determined. Labels illustrate all possible phosphate/phosphonate binding modes and refer to the entirety of all formed isomers.


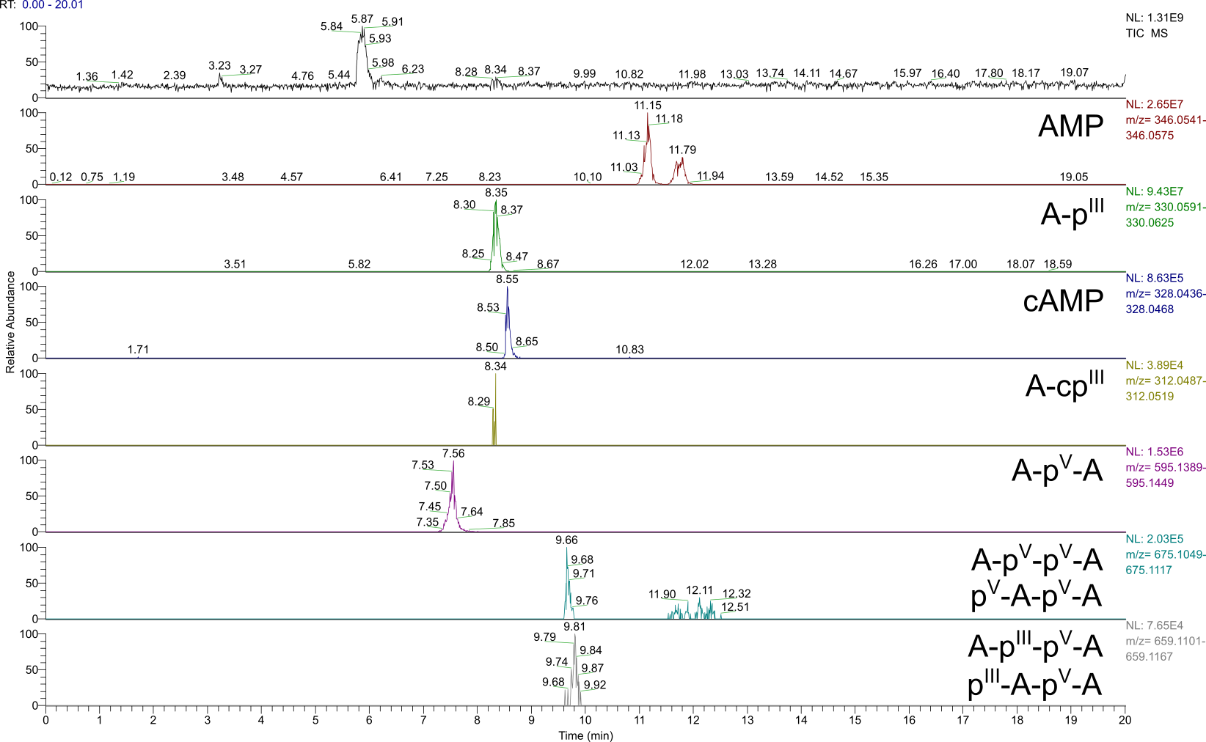


Supplementary Figure 30. TIC (m/z 80-1200) and EIEs (Δ = 5.0 ppm) of the products of the reaction starting from A (100 mM), H_3_PO_3_ (3.0 eq.) and urea (1.0 eq.) after 3 d. Constitution of potential isomers has not been determined. Labels illustrate all possible phosphate/phosphonate binding modes and refer to the entirety of all formed isomers.


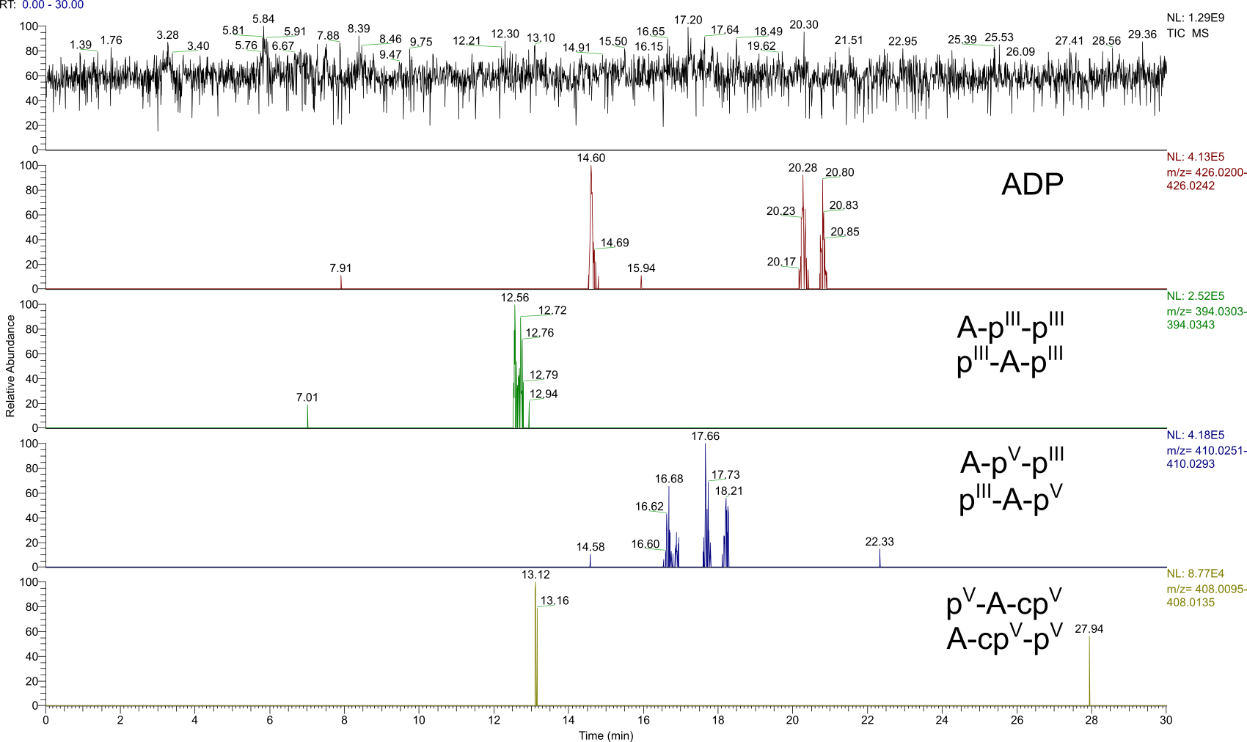


Supplementary Figure 31. TIC (m/z 50-750) and EIEs (Δ = 5.0 ppm) of the products of the reaction starting from A (100 mM), H_3_PO_3_ (3.0 eq.) and urea (1.0 eq.) after 3 d. Constitution of potential isomers has not been determined. Labels illustrate all possible phosphate/phosphonate binding modes and refer to the entirety of all formed isomers.


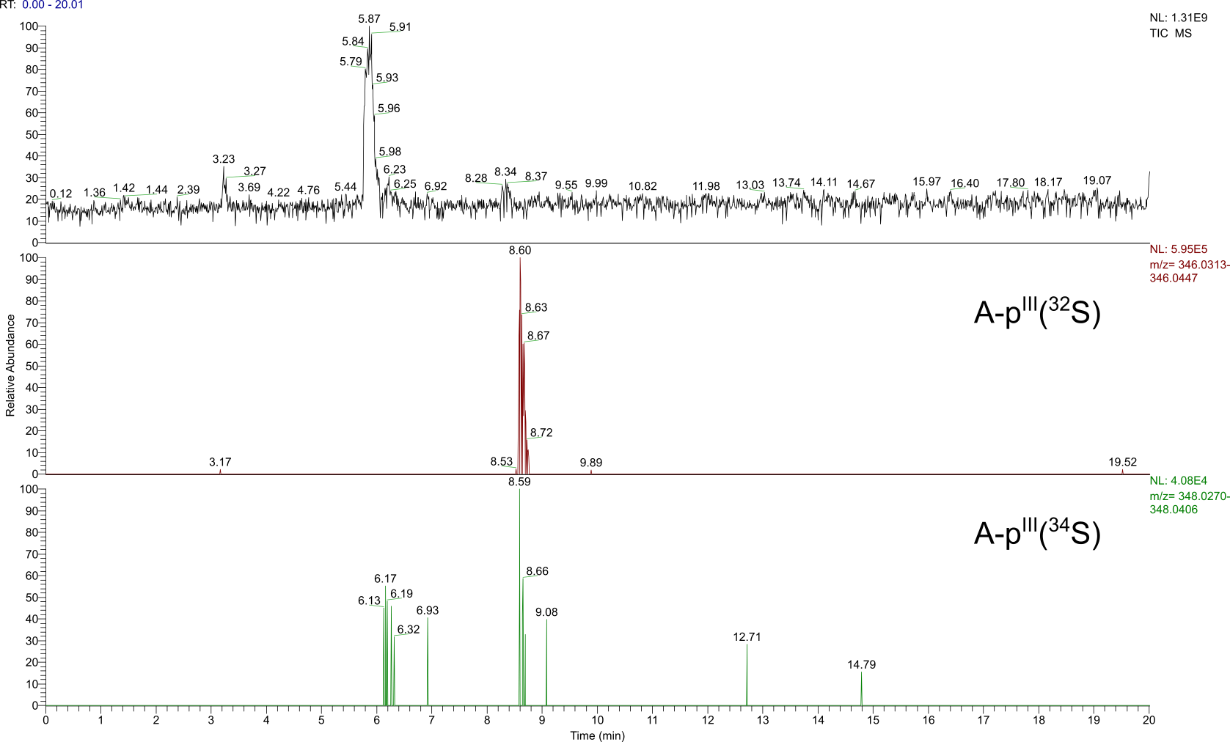


Supplementary Figure 32. TIC (m/z 80-1200) and EIEs (Δ = 19.5 ppm) of the thiophosphonate product of the reaction starting from A (100 mM), H_3_PO_3_ (3.0 eq.) and urea (1.0 eq.) after 3 d. Constitution of potential isomers has not been determined. Labels illustrate all possible phosphate/phosphonate binding modes and refer to the entirety of all formed isomers.


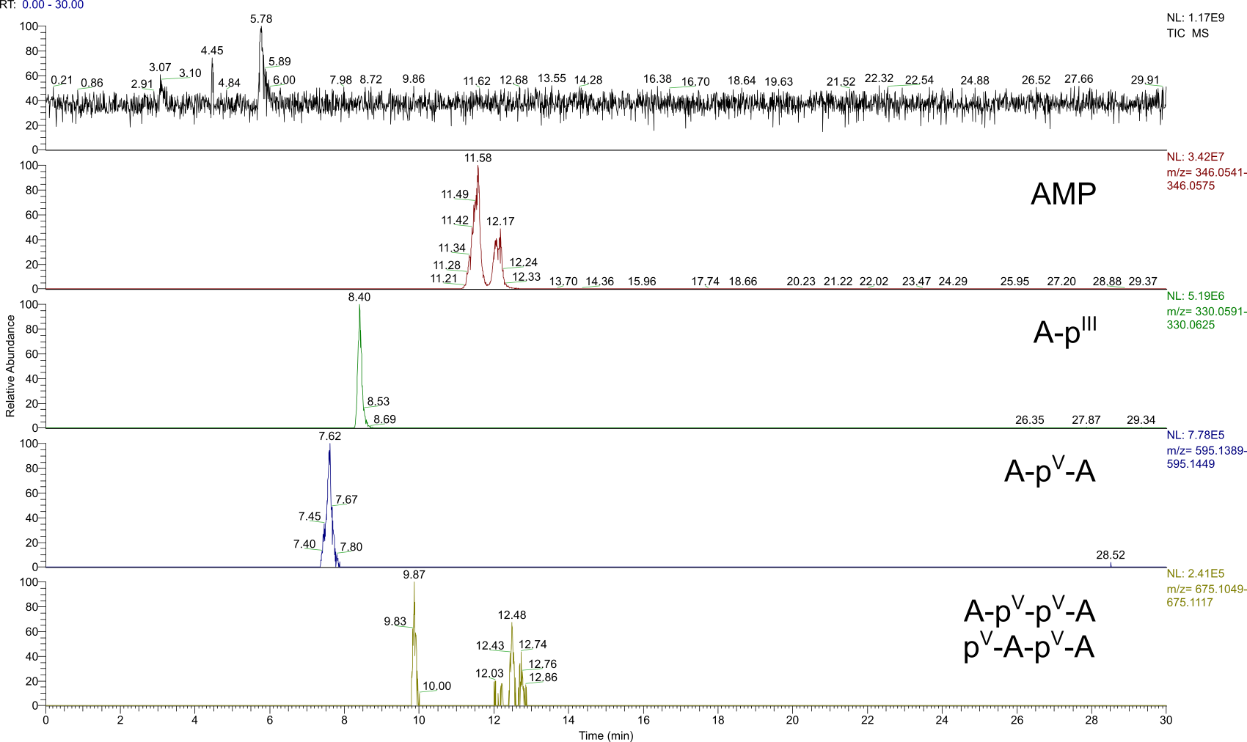


Supplementary Figure 33. TIC (m/z 50-750) and EIEs (Δ = 5.0 ppm) of the products of the reaction starting from A (100 mM), H_3_PO_3_ (3.0 eq.) and urea (1.0 eq.) after 26 d. Constitution of potential isomers has not been determined. Labels illustrate all possible phosphate/phosphonate binding modes and refer to the entirety of all formed isomers.


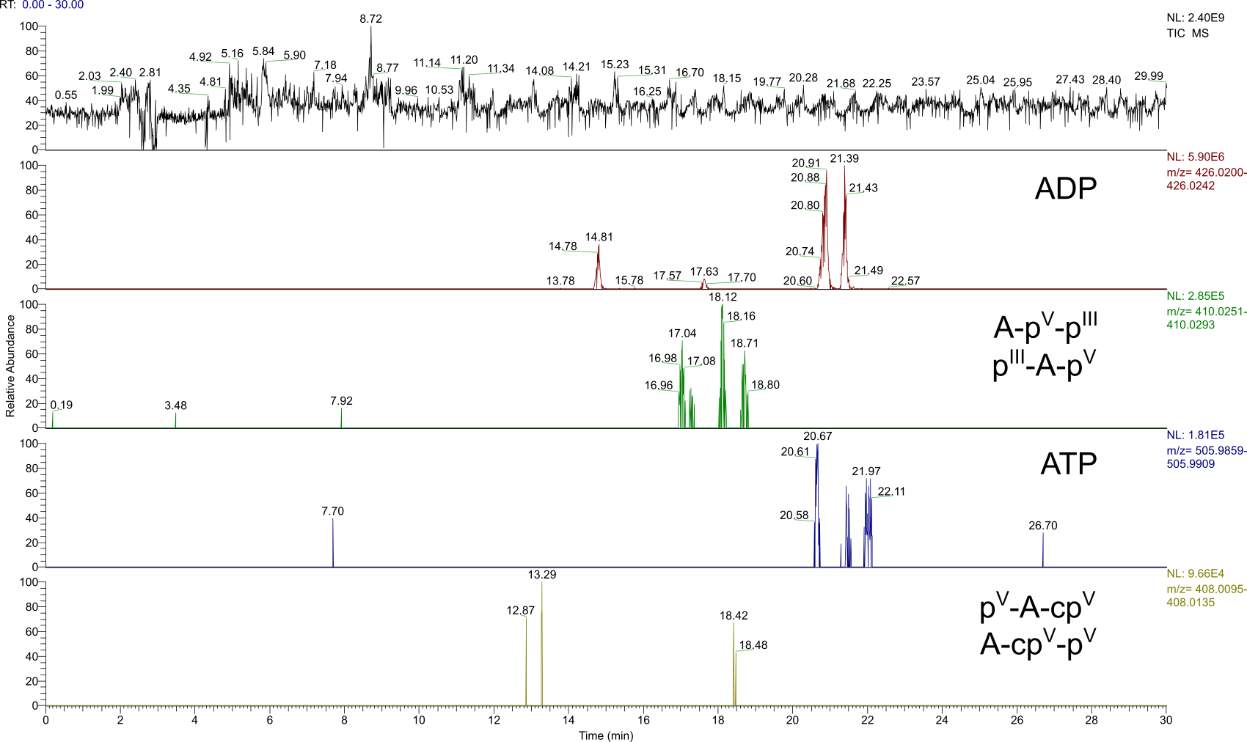


Supplementary Figure 34. TIC (m/z 50-750) and EIEs (Δ = 5.0 ppm) of the products of the reaction starting from A (100 mM), H_3_PO_3_ (3.0 eq.) and urea (1.0 eq.) after 26 d. Constitution of potential isomers has not been determined. Labels illustrate all possible phosphate/phosphonate binding modes and refer to the entirety of all formed isomers.


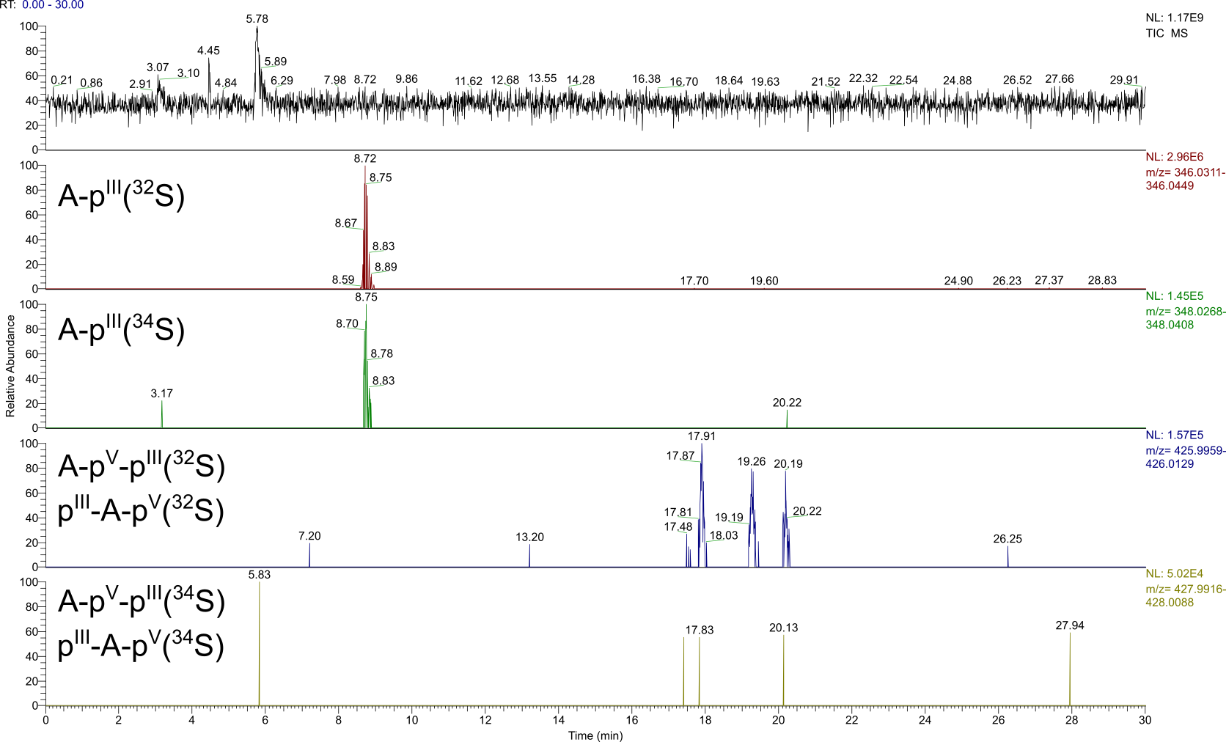


Supplementary Figure 35. TIC (m/z 50-750) and EIEs (Δ = 20.0 ppm) of the thiophosphonate/-phosphate products of the reaction starting from A (100 mM), H_3_PO_3_ (3.0 eq.) and urea (1.0 eq.) after 26 d. Constitution of potential isomers has not been determined. Labels illustrate all possible phosphate/phosphonate binding modes and refer to the entirety of all formed isomers.

#### Reactions with H_3_PO_4_


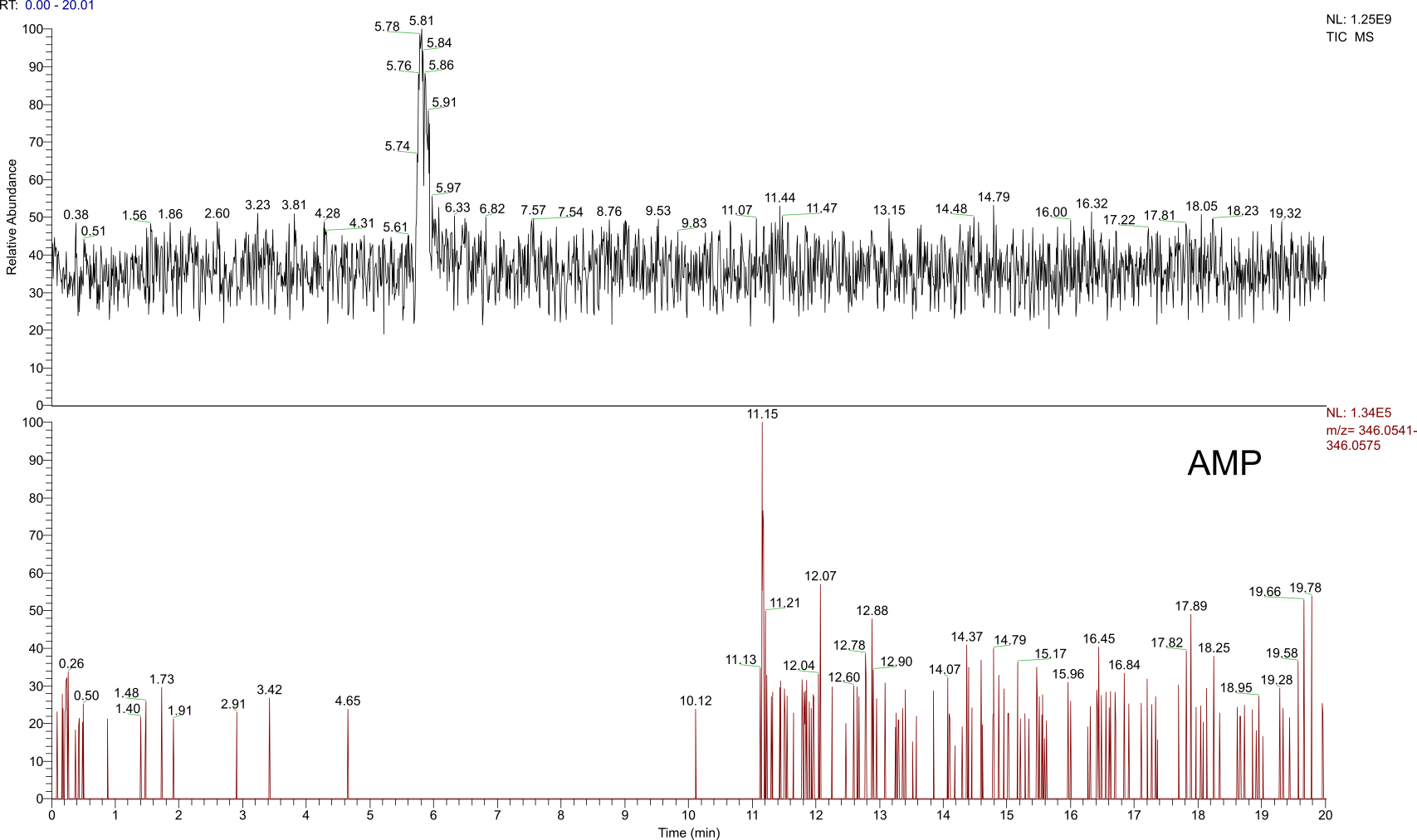


Supplementary Figure 36. TIC (m/z 50-750) and EIE (Δ = 5.0 ppm) of the product of the reaction starting from A (100 mM) and H_3_PO_4_ (1.0 eq.) after 7 d. Constitution of potential isomers has not been determined. The label illustrates all possible phosphate binding modes and refers to the entirety of all formed isomers.


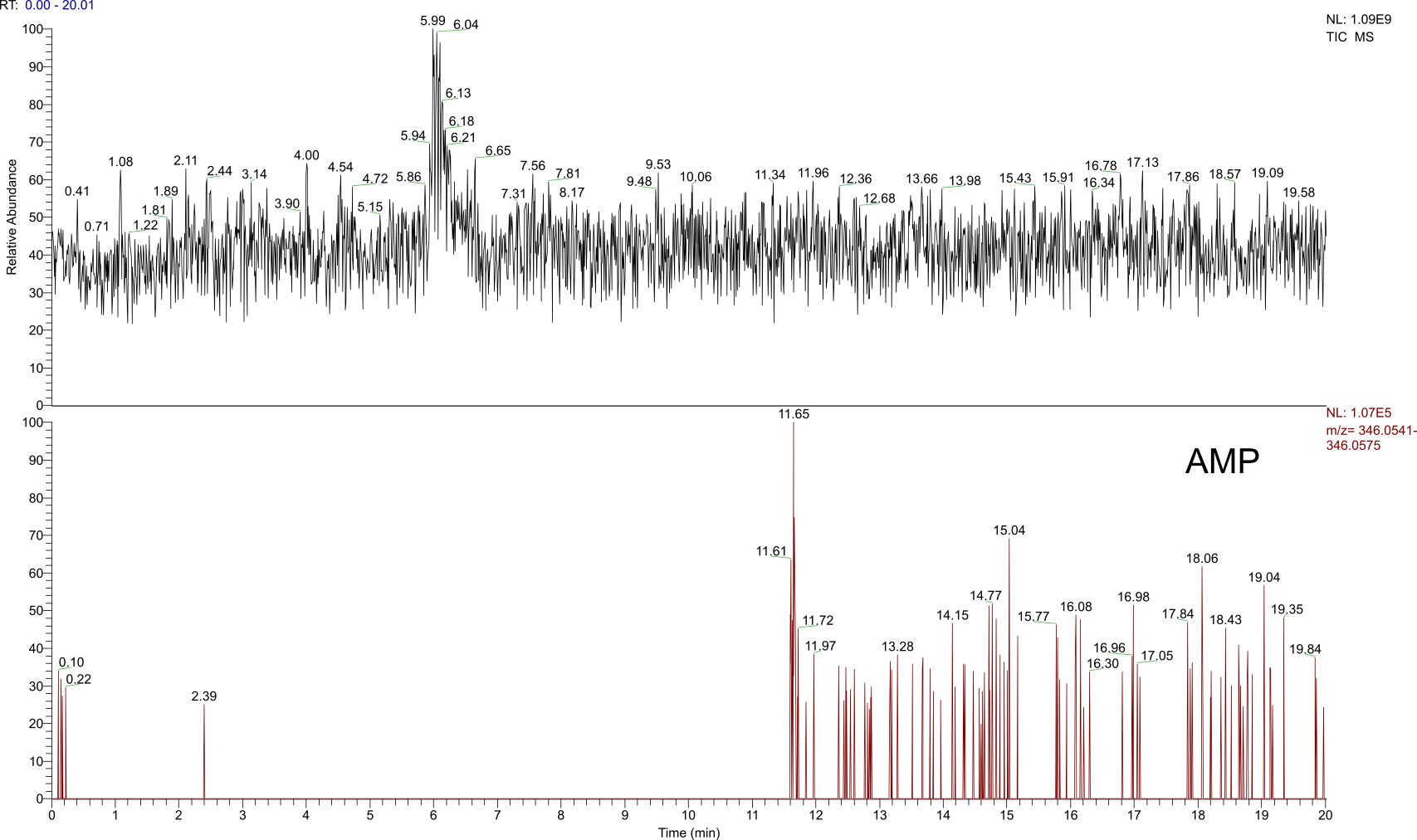


Supplementary Figure 37. TIC (m/z 50-750) and EIE (Δ = 5.0 ppm) of the product of the reaction starting from A (100 mM), H_3_PO_4_ and urea (each 1.0 eq.) after 14 d. Constitution of potential isomers has not been determined. The label illustrates all possible phosphate binding modes and refers to the entirety of all formed isomers.

### Reaction with 5’ AMP


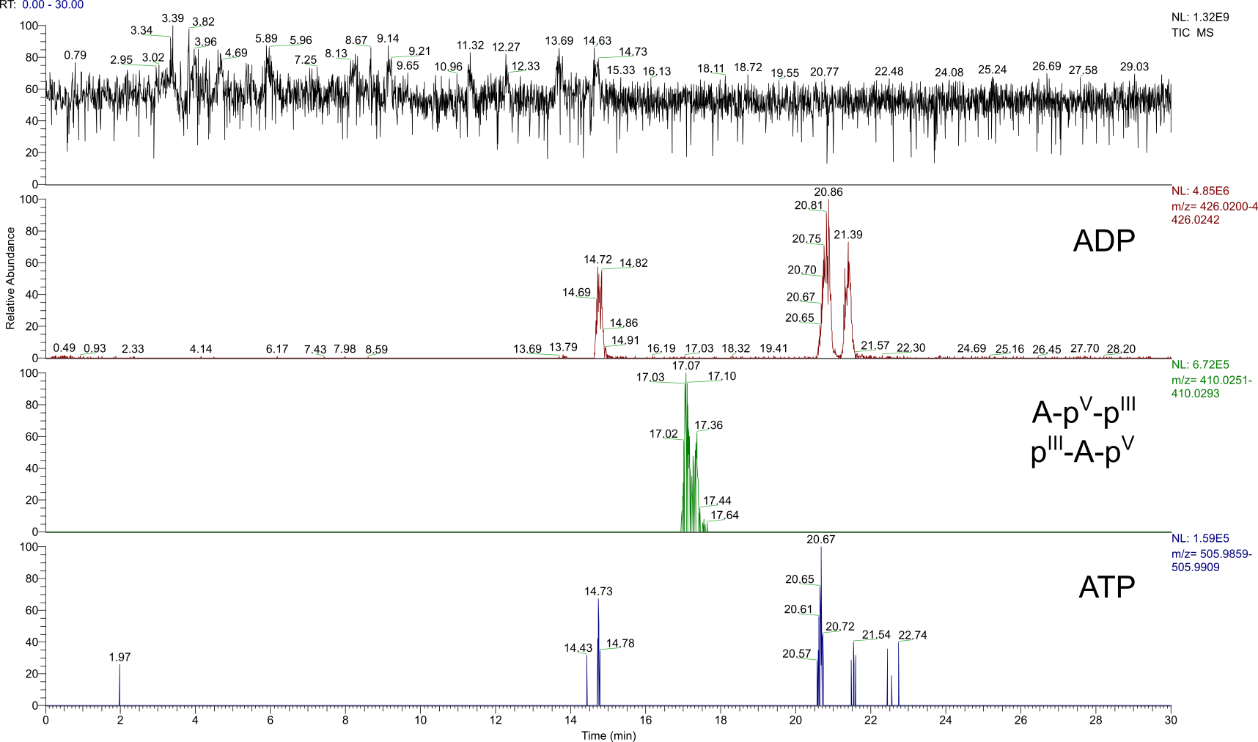


Supplementary Figure 38. TIC (m/z 50-750) and EIEs (Δ = 5.0 ppm) of the products of the reaction starting from 5’ AMP (100 mM), H_3_PO_3_ (3.0 eq.) and urea (1.0 eq.) after 7 d. Constitution of potential isomers has not been determined. Labels illustrate all possible phosphate/phosphonate binding modes and refer to the entirety of all formed isomers.


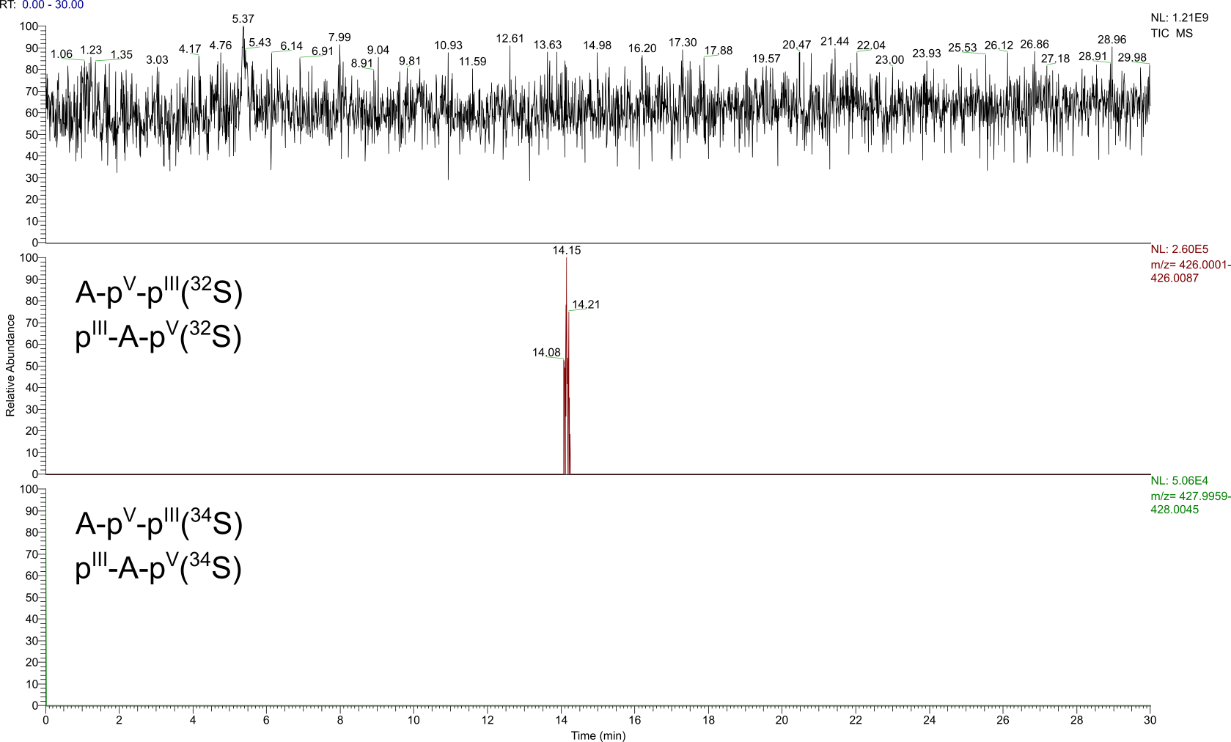


Supplementary Figure 39. TIC (m/z 50-750) and EIEs (Δ = 10.0 ppm) of the thiophosphonate/-phosphate product of the reaction starting from 5’ AMP (100 mM), H_3_PO_3_ (3.0 eq.) and urea (1.0 eq.) after 7 d. Constitution of potential isomers has not been determined. Labels illustrate all possible phosphate/phosphonate binding modes and refer to the entirety of all formed isomers.

### Reactions with 5’ ADP


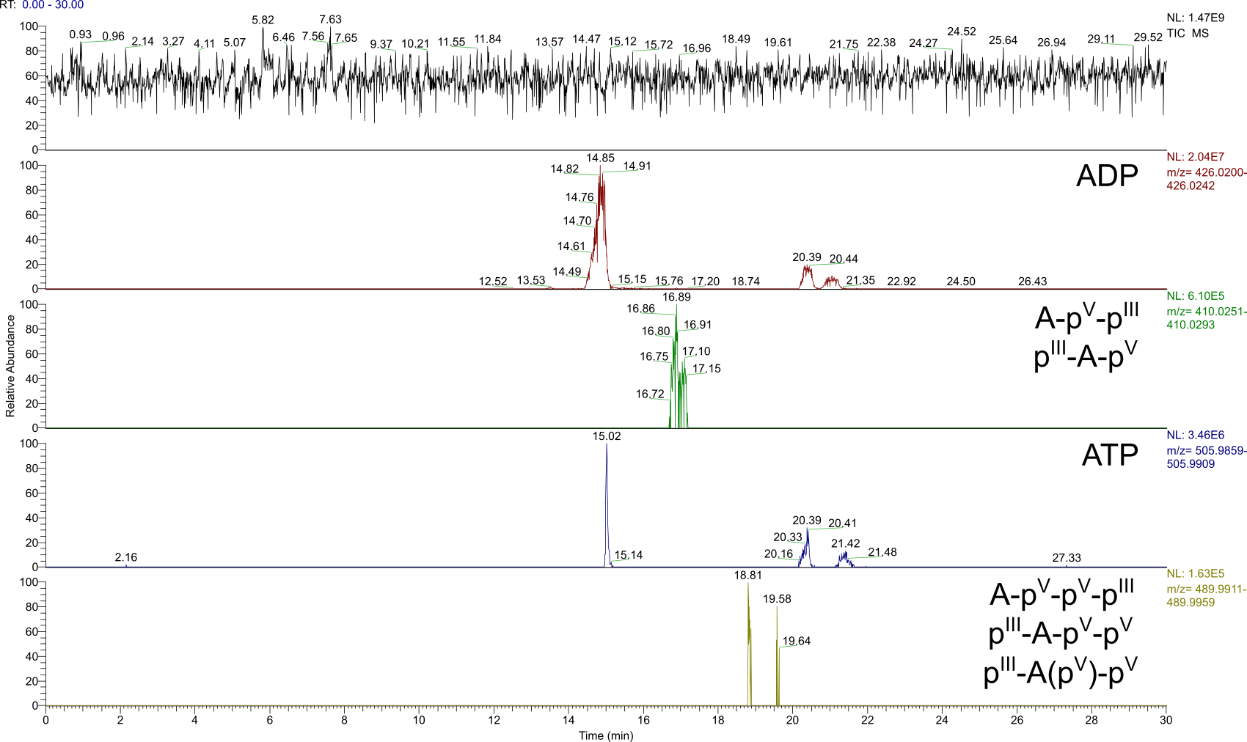


Supplementary Figure 40. TIC (m/z 50-750) and EIEs (Δ = 5.0 ppm) of the products of the reaction starting from 5’ ADP (100 mM), H_3_PO_3_ (3.0 eq.) and urea (1.0 eq.) after 7 d. Constitution of potential isomers has not been determined. Labels illustrate all possible phosphate/phosphonate binding modes and refer to the entirety of all formed isomers.


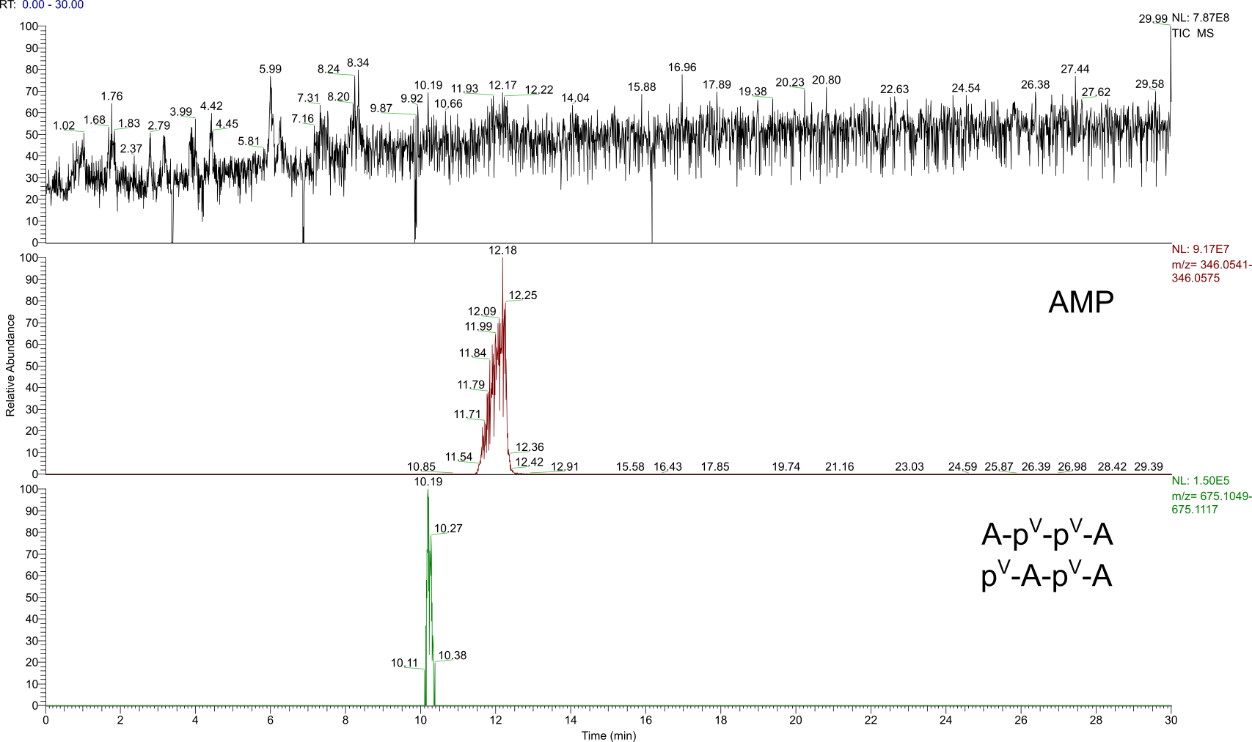


Supplementary Figure 41. TIC (m/z 50-750) and EIEs (Δ = 5.0 ppm) of the products of the reaction starting from 5’ ADP (100 mM), H_3_PO_3_ (3.0 eq.) and urea (1.0 eq.) after 7 d. Constitution of potential isomers has not been determined. Labels illustrate all possible phosphate/phosphonate binding modes and refer to the entirety of all formed isomers.


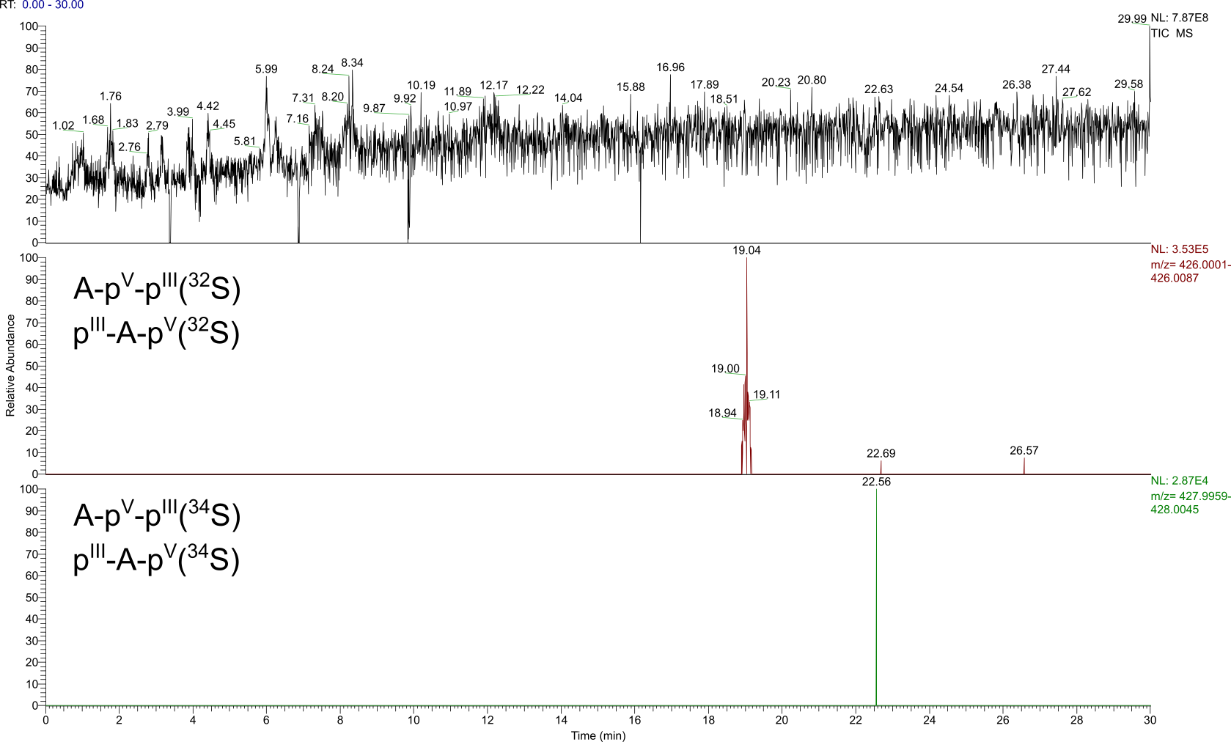


Supplementary Figure 42. TIC (m/z 50-750) and EIEs (Δ = 10.0 ppm) of the thiophosphonate/-phosphate product of the reaction starting from 5’ ADP (100 mM), H_3_PO_3_ (3.0 eq.) and urea (1.0 eq.) after 7 d. Constitution of potential isomers has not been determined. Labels illustrate all possible phosphate/phosphonate binding modes and refer to the entirety of all formed isomers.


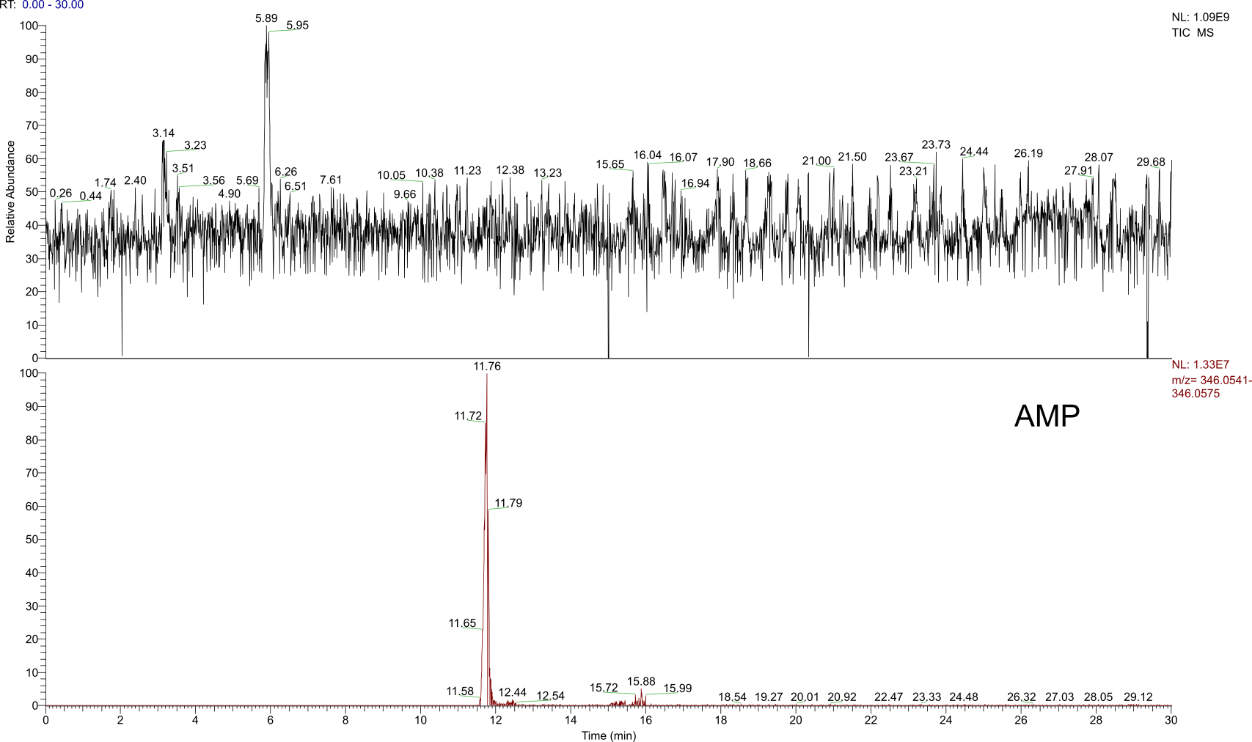


Supplementary Figure 43. TIC (m/z 50-750) and EIEs (Δ = 5.0 ppm) of the products of the reaction starting from 5’ ADP, A and urea (each 100 mM) after 7 d. Constitution of potential isomers has not been determined. The label illustrates all possible phosphate binding modes and refers to the entirety of all formed isomers.


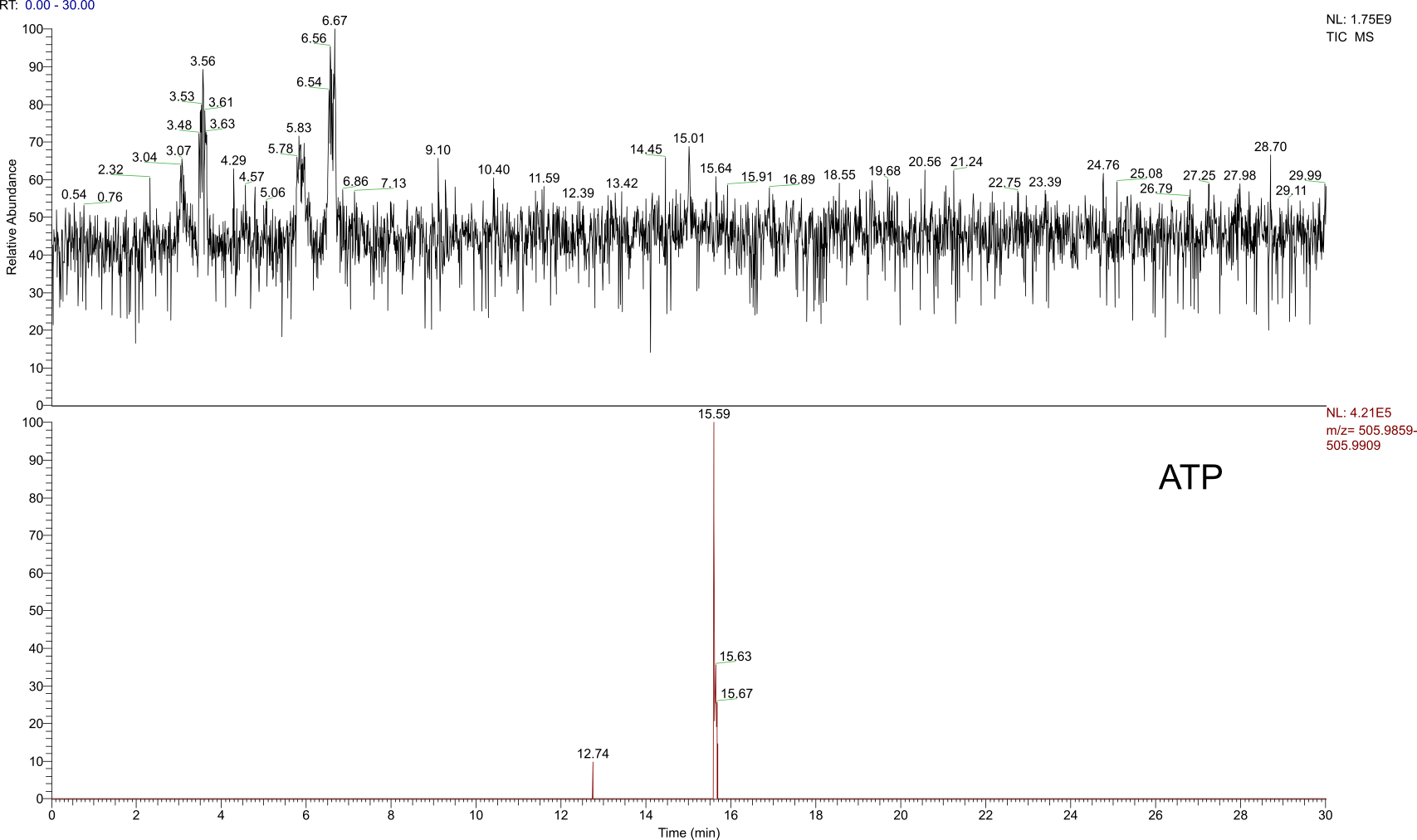


Supplementary Figure 44. TIC (m/z 50-750) and EIEs (Δ = 5.0 ppm) of the products of the reaction starting from 5’ ADP, A and urea (each 100 mM) after 7 d. Constitution of potential isomers has not been determined. The label illustrates all possible phosphate binding modes and refers to the entirety of all formed isomers.

### Reaction with cytidine


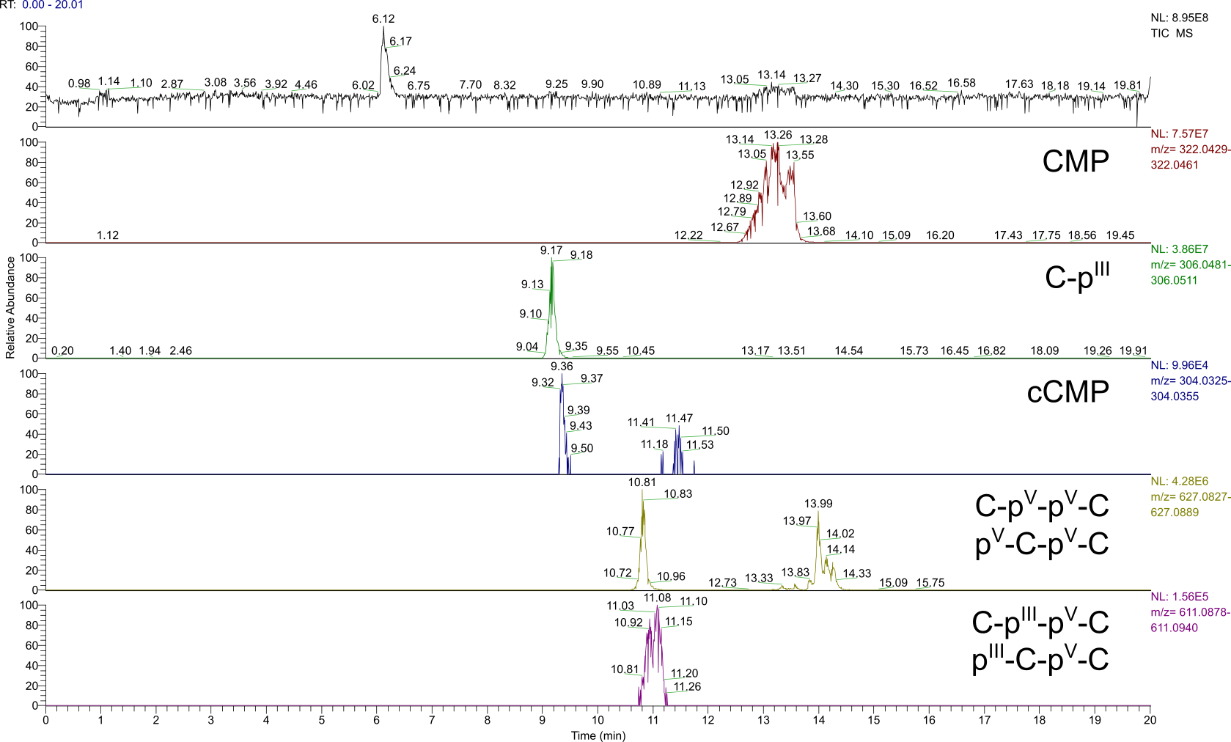


Supplementary Figure 45. TIC (m/z 80-1200) and EIEs (Δ = 5.0 ppm) of the products of the reaction starting from C (100 mM), H_3_PO_3_ (3.0 eq.) and urea (1.0 eq.) after 7 d. Constitution of potential isomers has not been determined. Labels illustrate all possible phosphate/phosphonate binding modes and refer to the entirety of all formed isomers.


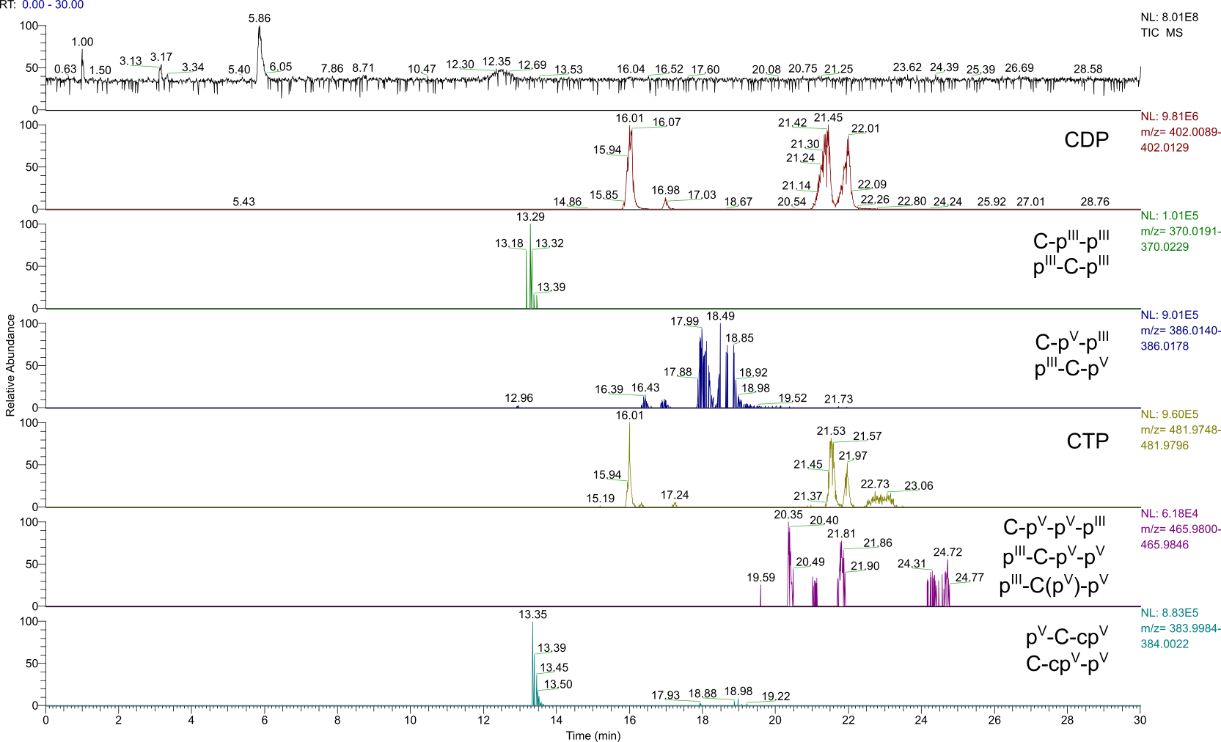


Supplementary Figure 46. TIC (m/z 80-1200) and EIEs (Δ = 5.0 ppm) of the products of the reaction starting from C (100 mM), H_3_PO_3_ (3.0 eq.) and urea (1.0 eq.) after 7 d. Constitution of potential isomers has not been determined. Labels illustrate all possible phosphate/phosphonate binding modes and refer to the entirety of all formed isomers.


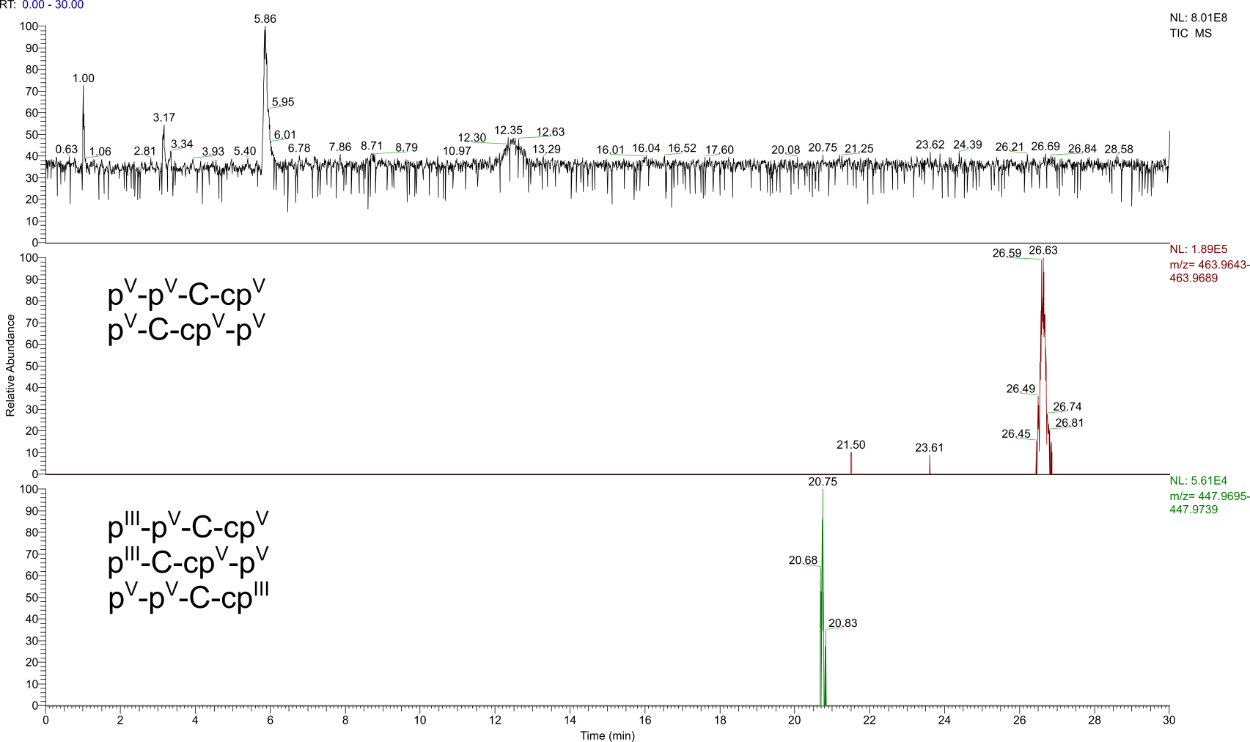


Supplementary Figure 47. TIC (m/z 80-1200) and EIEs (Δ = 5.0 ppm) of the products of the reaction starting from C (100 mM), H_3_PO_3_ (3.0 eq.) and urea (1.0 eq.) after 7 d. Constitution of potential isomers has not been determined. Labels illustrate all possible phosphate/phosphonate binding modes and refer to the entirety of all formed isomers.


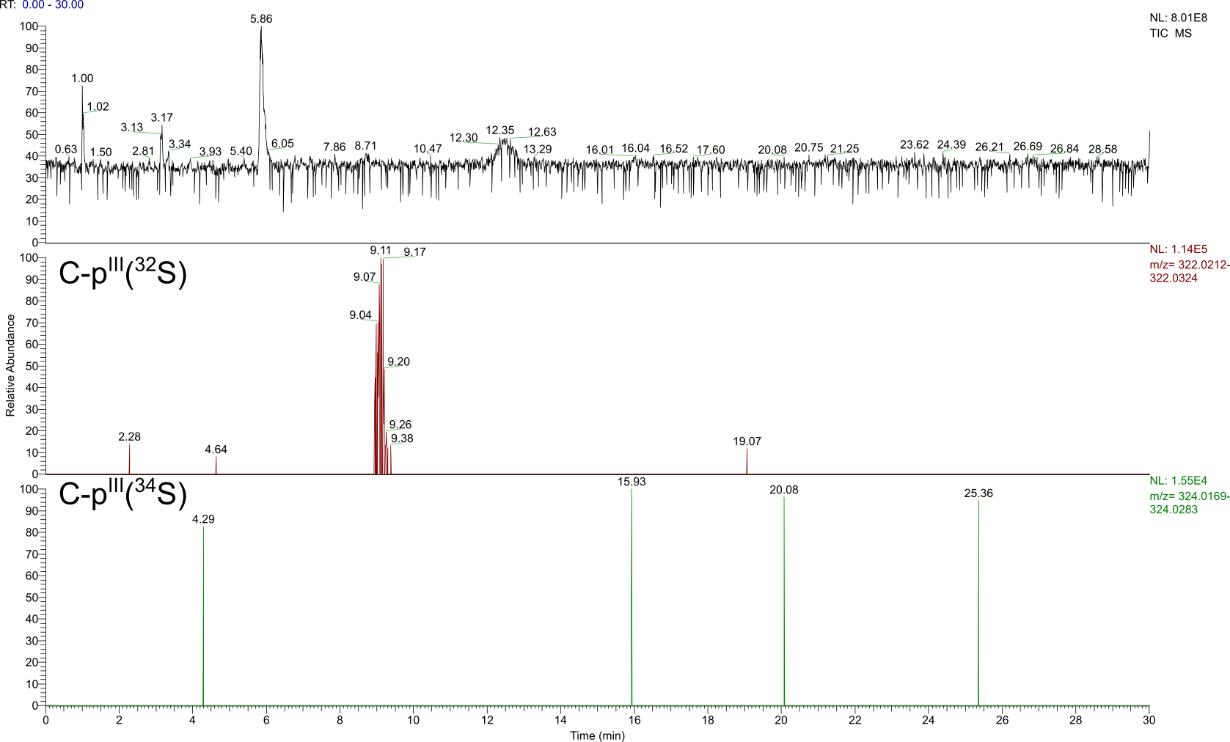


Supplementary Figure 48. TIC (m/z 80-1200) and EIEs (Δ = 17.5 ppm) of the thiophosphonate product of the reaction starting from C (100 mM), H_3_PO_3_ (3.0 eq.) and urea (1.0 eq.) after 7 d. Constitution of potential isomers has not been determined. Labels illustrate all possible phosphate/phosphonate binding modes and refer to the entirety of all formed isomers.


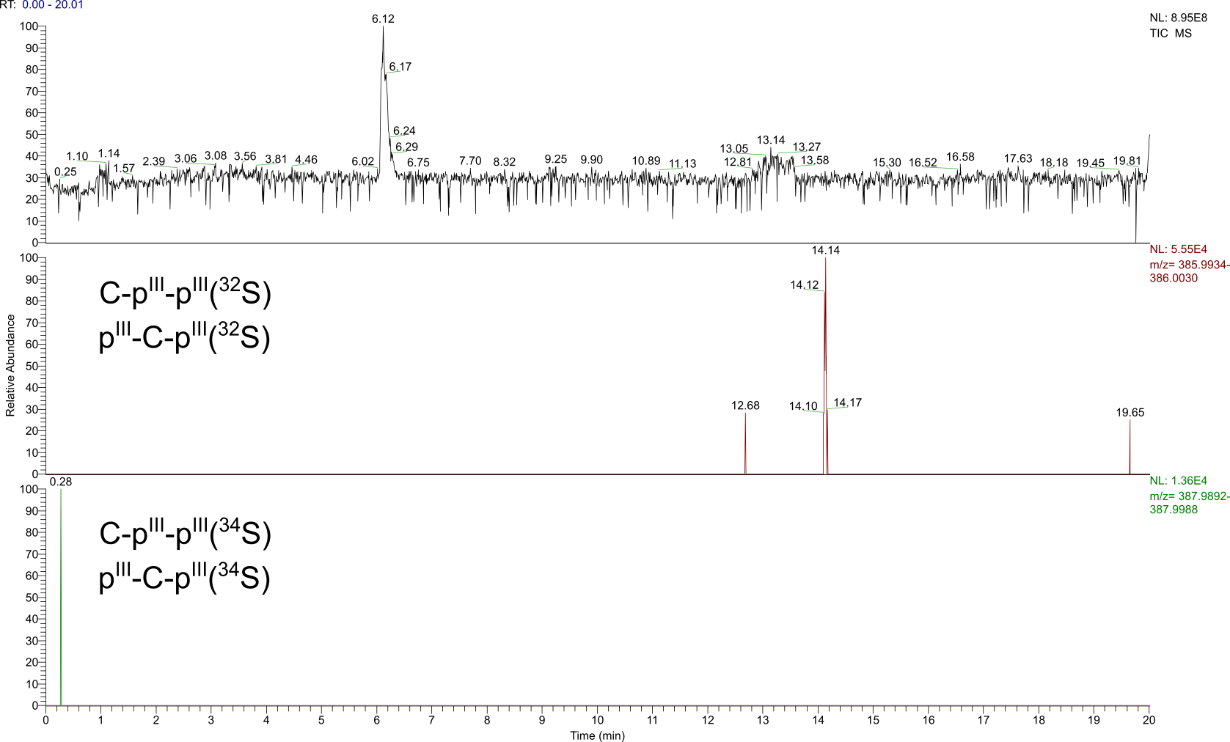


Supplementary Figure 49. TIC (m/z 80-1200) and EIEs (Δ = 12.5 ppm) of the thiophosphonate/-phosphate product of the reaction starting from C (100 mM), H_3_PO_3_ (3.0 eq.) and urea (1.0 eq.) after 7 d. Constitution of potential isomers has not been determined. Labels illustrate all possible phosphate/phosphonate binding modes and refer to the entirety of all formed isomers.

### Reaction with uridine


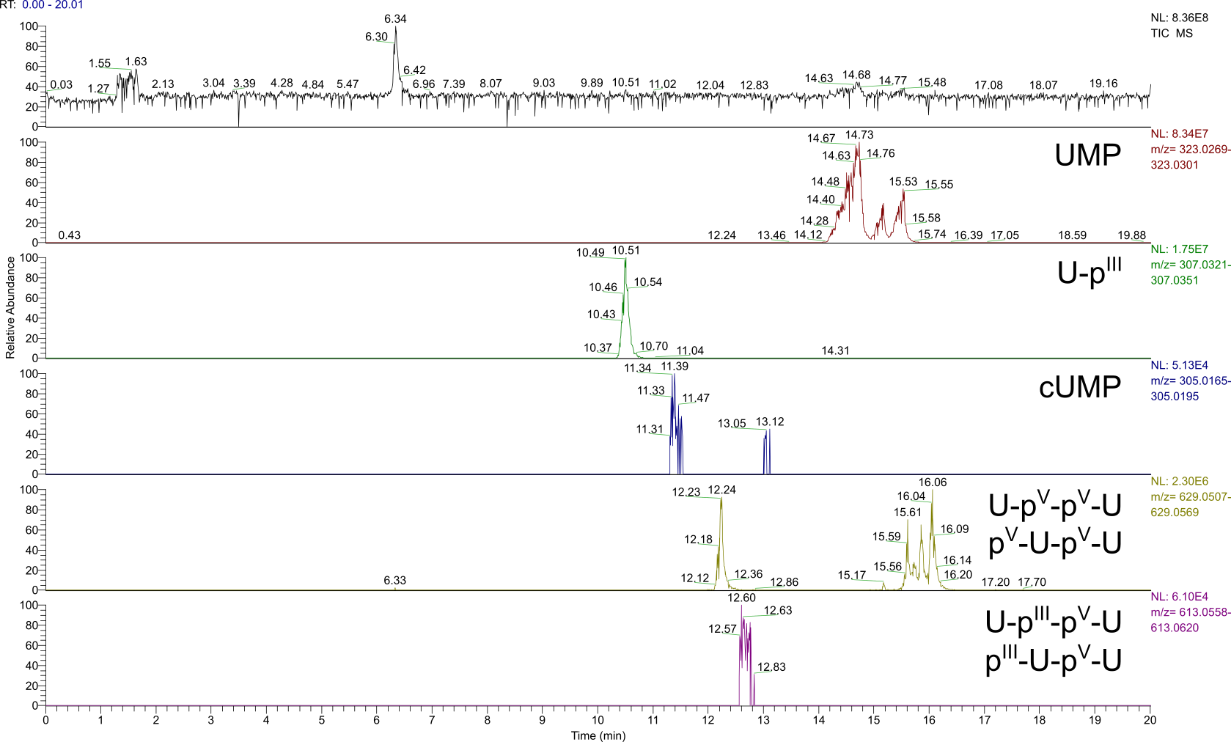


Supplementary Figure 50. TIC (m/z 80-1200) and EIEs (Δ = 5.0 ppm) of the products of the reaction starting from U (100 mM), H_3_PO_3_ (3.0 eq.) and urea (1.0 eq.) after 7 d. Constitution of potential isomers has not been determined. Labels illustrate all possible phosphate/phosphonate binding modes and refer to the entirety of all formed isomers.


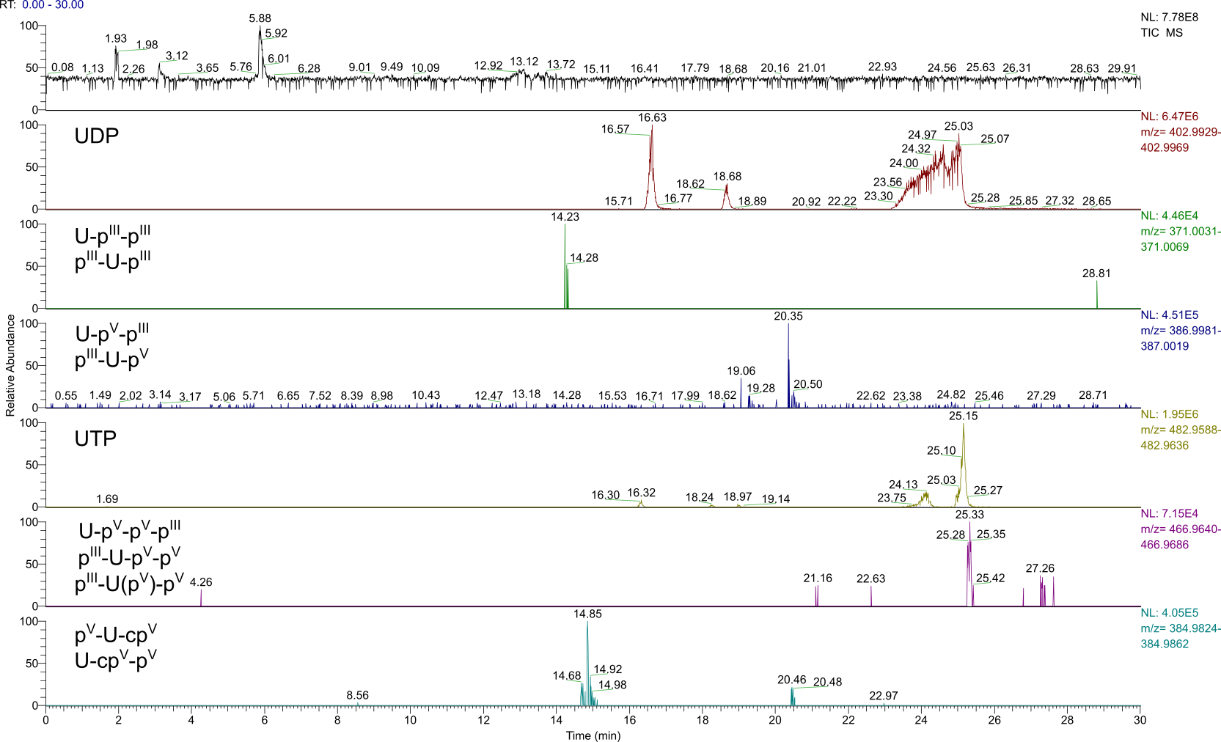


Supplementary Figure 51. TIC (m/z 80-1200) and EIEs (Δ = 5.0 ppm) of the products of the reaction starting from U (100 mM), H_3_PO_3_ (3.0 eq.) and urea (1.0 eq.) after 7 d. Constitution of potential isomers has not been determined. Labels illustrate all possible phosphate/phosphonate binding modes and refer to the entirety of all formed isomers.


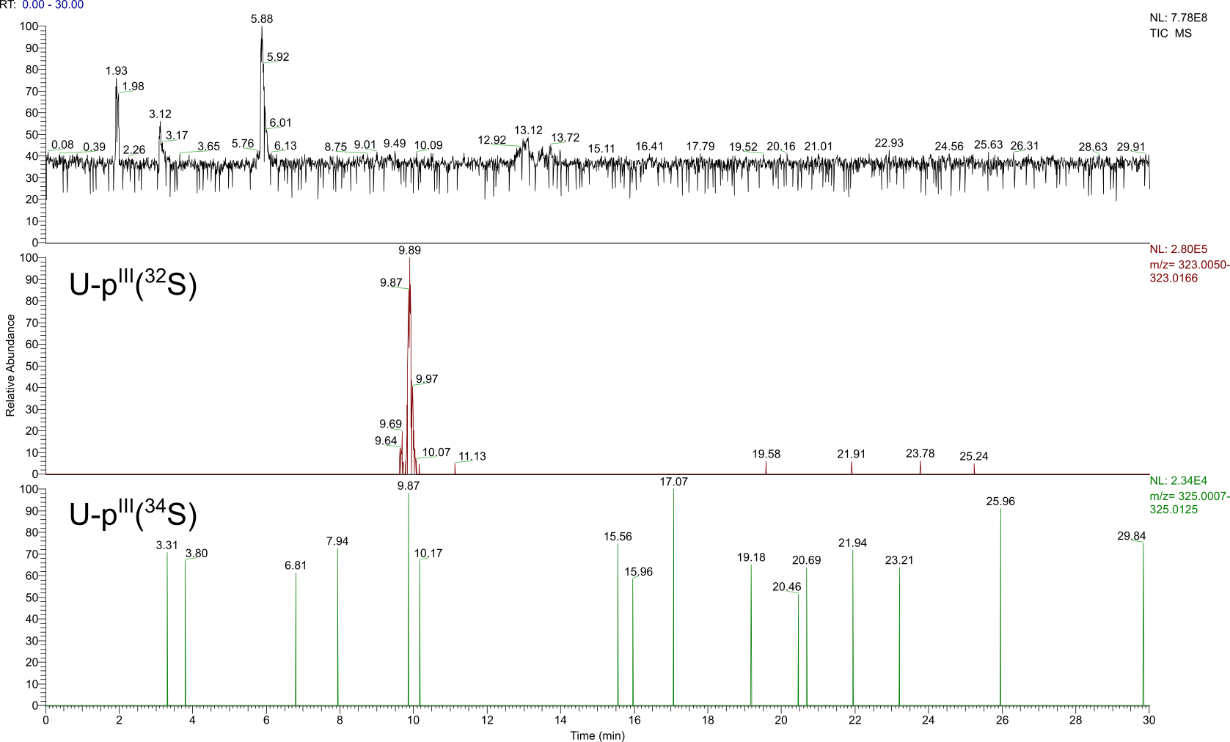


Supplementary Figure 52. TIC (m/z 80-1200) and EIEs (Δ = 18.0 ppm) of the thiophosphonate product of the reaction starting from U (100 mM), H_3_PO_3_ (3.0 eq.) and urea (1.0 eq.) after 7 d. Constitution of potential isomers has not been determined. Labels illustrate all possible phosphate/phosphonate binding modes and refer to the entirety of all formed isomers.

### Reaction with guanosine


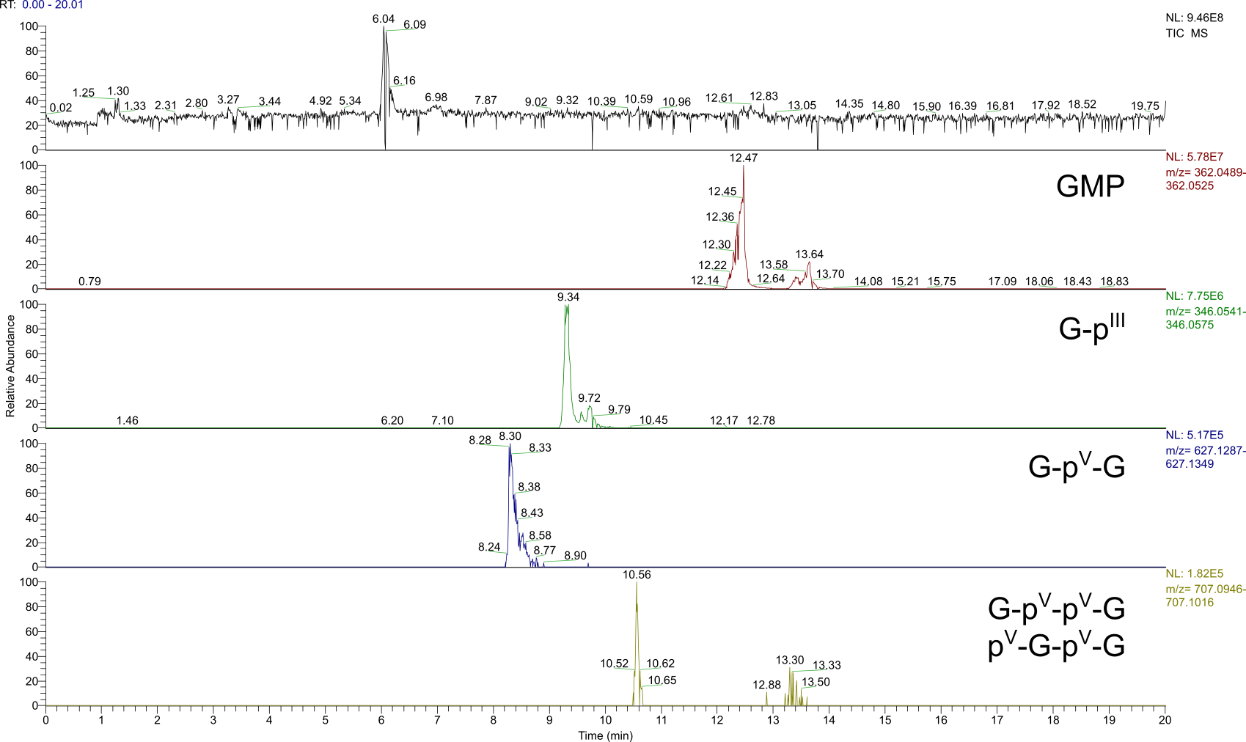


Supplementary Figure 53. TIC (m/z 80-1200) and EIEs (Δ = 5.0 ppm) of the products of the reaction starting from G (100 mM), H_3_PO_3_ (3.0 eq.) and urea (1.0 eq.) after 7 d. Constitution of potential isomers has not been determined. Labels illustrate all possible phosphate/phosphonate binding modes and refer to the entirety of all formed isomers.


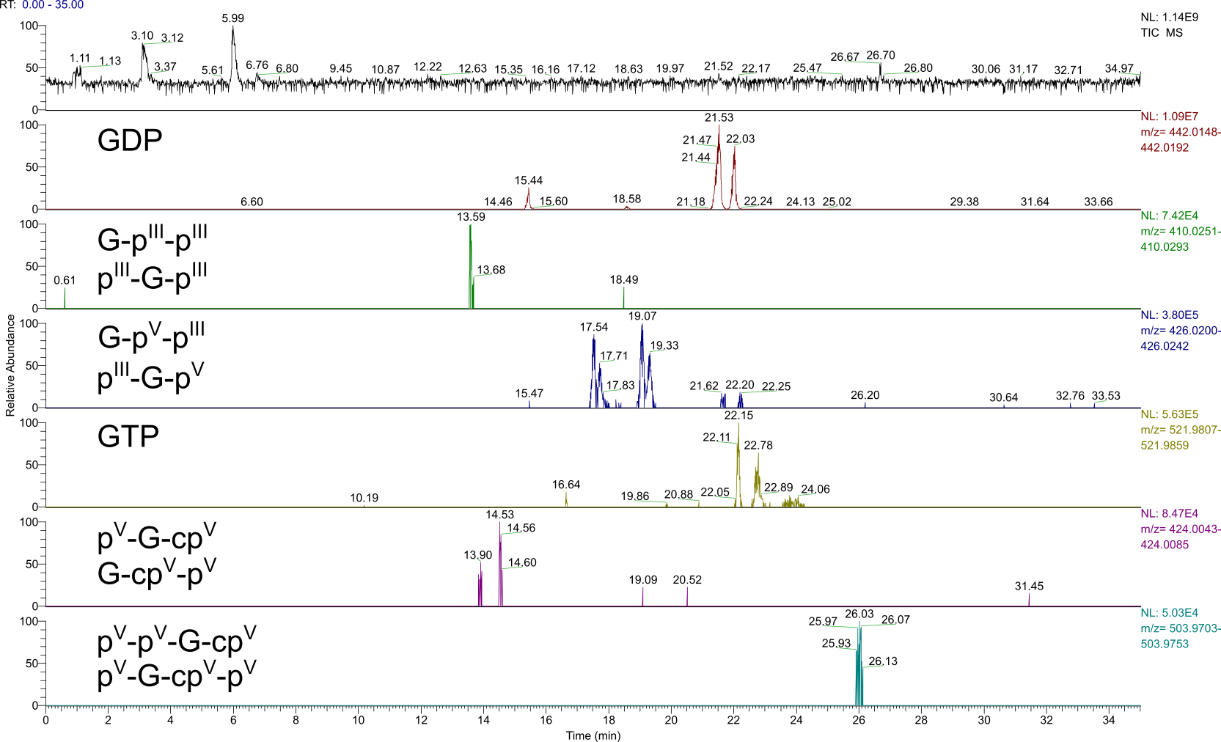


Supplementary Figure 54. TIC (m/z 80-1200) and EIEs (Δ = 5.0 ppm) of the products of the reaction starting from G (100 mM), H_3_PO_3_ (3.0 eq.) and urea (1.0 eq.) after 7 d. Constitution of potential isomers has not been determined. Labels illustrate all possible phosphate/phosphonate binding modes and refer to the entirety of all formed isomers.


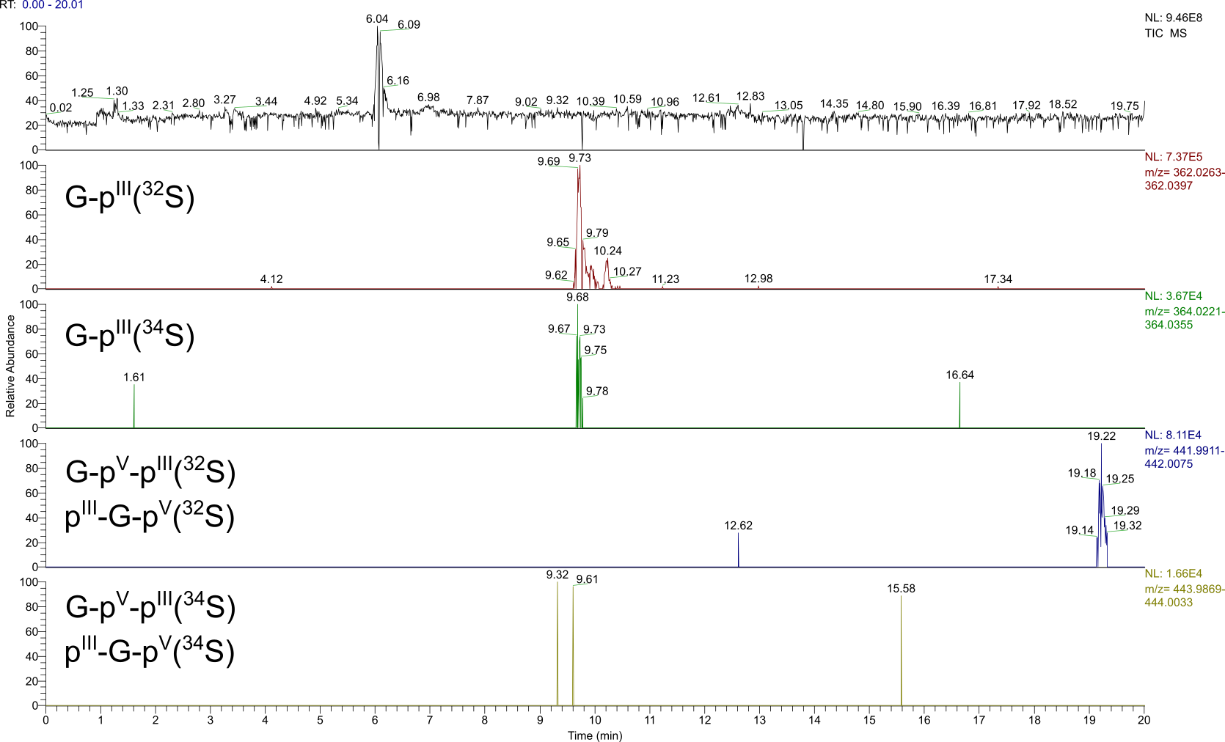


Supplementary Figure 55. TIC (m/z 80-1200) and EIEs (Δ = 18.5 ppm) of the thiophosphonate/-phosphate products of the reaction starting from G (100 mM), H_3_PO_3_ (3.0 eq.) and urea (1.0 eq.) after 7 d. Constitution of potential isomers has not been determined. Labels illustrate all possible phosphate/phosphonate binding modes and refer to the entirety of all formed isomers.

### Reaction with deoxyadenosine


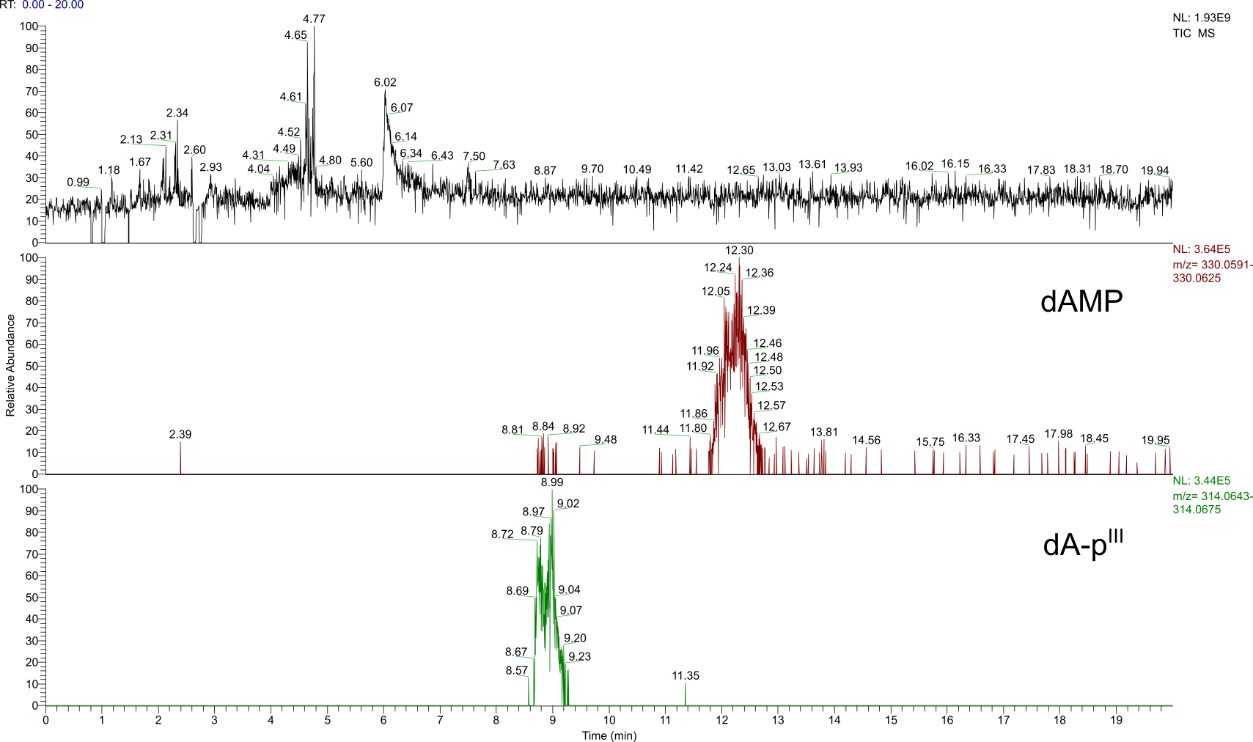


Supplementary Figure 56. TIC (m/z 50-750) and EIEs (Δ = 5.0 ppm) of the products of the reaction starting from deoxyadenosine (dA) (100 mM), H_3_PO_3_ (3.0 eq.) and urea (1.0 eq.) after7 d. Constitution of potential isomers has not been determined. Labels illustrate all possible phosphate/phosphonate binding modes and refer to the entirety of all formed isomers.

### Reaction with deoxyguanosine


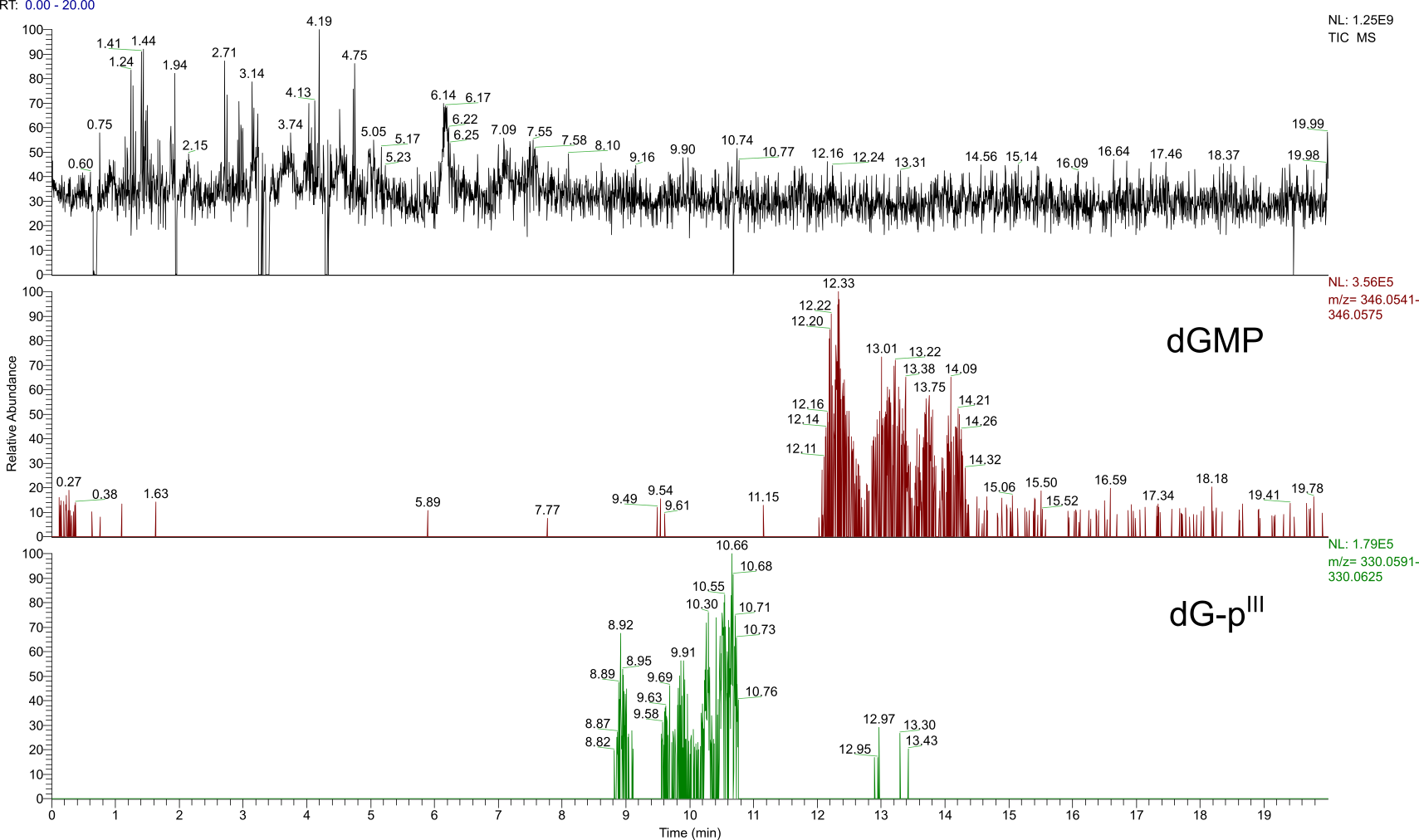


Supplementary Figure 57. TIC (m/z 50-750) and EIEs (Δ = 5.0 ppm) of the products of the reaction starting from deoxyguanosine (dG) (100 mM), H_3_PO_3_ (3.0 eq.) and urea (1.0 eq.) after 7 d. Constitution of potential isomers has not been determined. Labels illustrate all possible phosphate/phosphonate binding modes and refer to the entirety of all formed isomers.

### Reaction with deoxycytidine


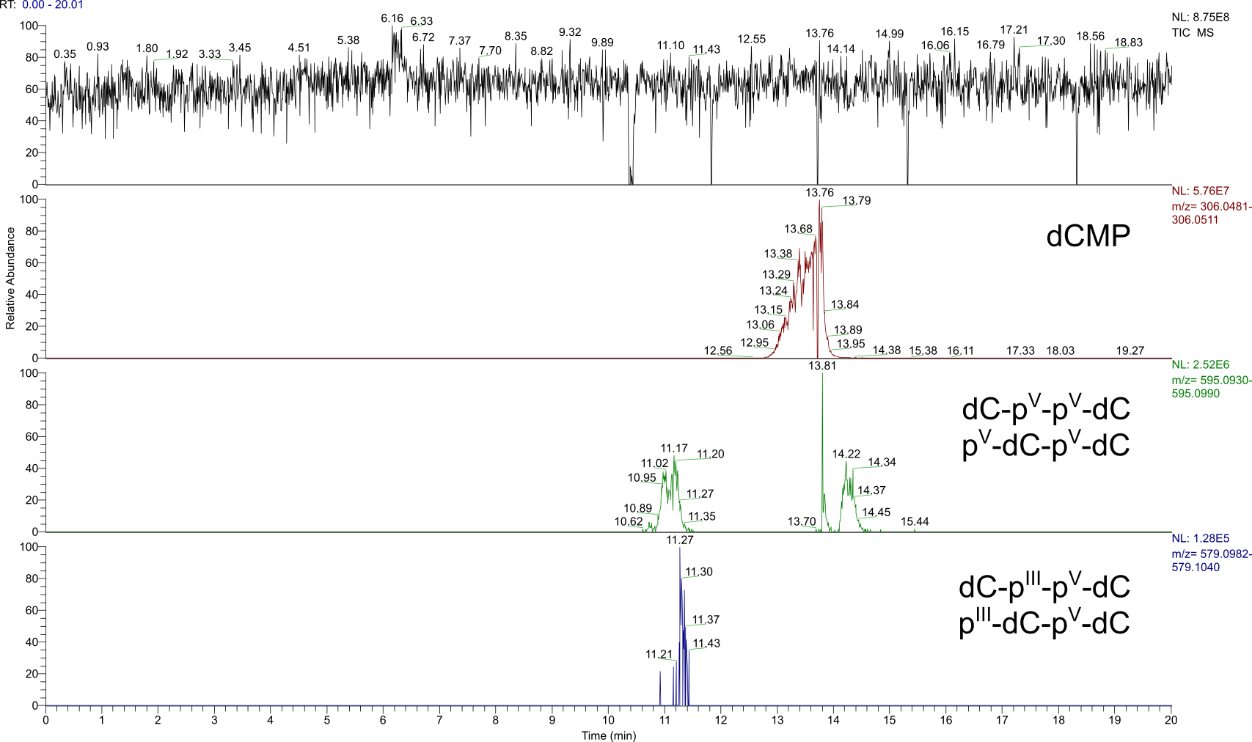


Supplementary Figure 58. TIC (m/z 50-750) and EIEs (Δ = 5.0 ppm) of the products of the reaction starting from dC (100 mM), H_3_PO_3_ (3.0 eq.) and urea (1.0 eq.) after 7 d. Constitution of potential isomers has not been determined. Labels illustrate all possible phosphate/phosphonate binding modes and refer to the entirety of all formed isomers.


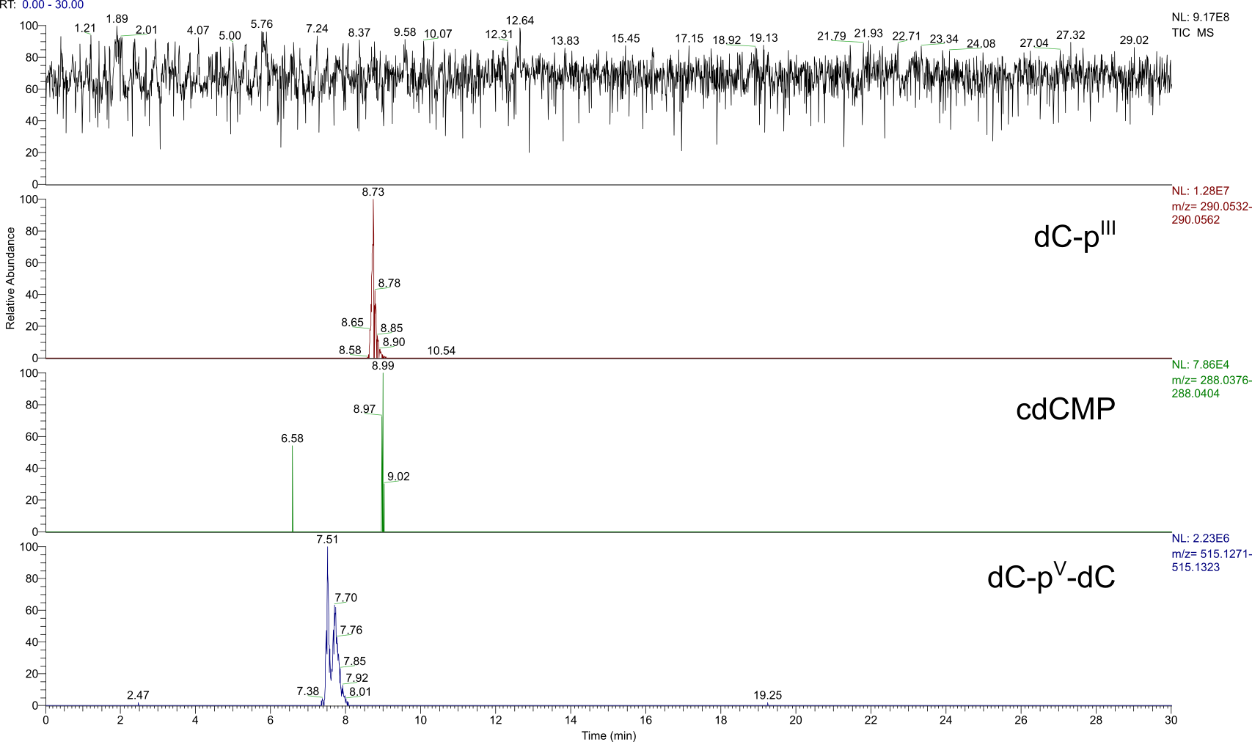


Supplementary Figure 59. TIC (m/z 50-750) and EIEs (Δ = 5.0 ppm) of the products of the reaction starting from dC (100 mM), H_3_PO_3_ (3.0 eq.) and urea (1.0 eq.) after 7 d. Constitution of potential isomers has not been determined. Labels illustrate all possible phosphate/phosphonate binding modes and refer to the entirety of all formed isomers.


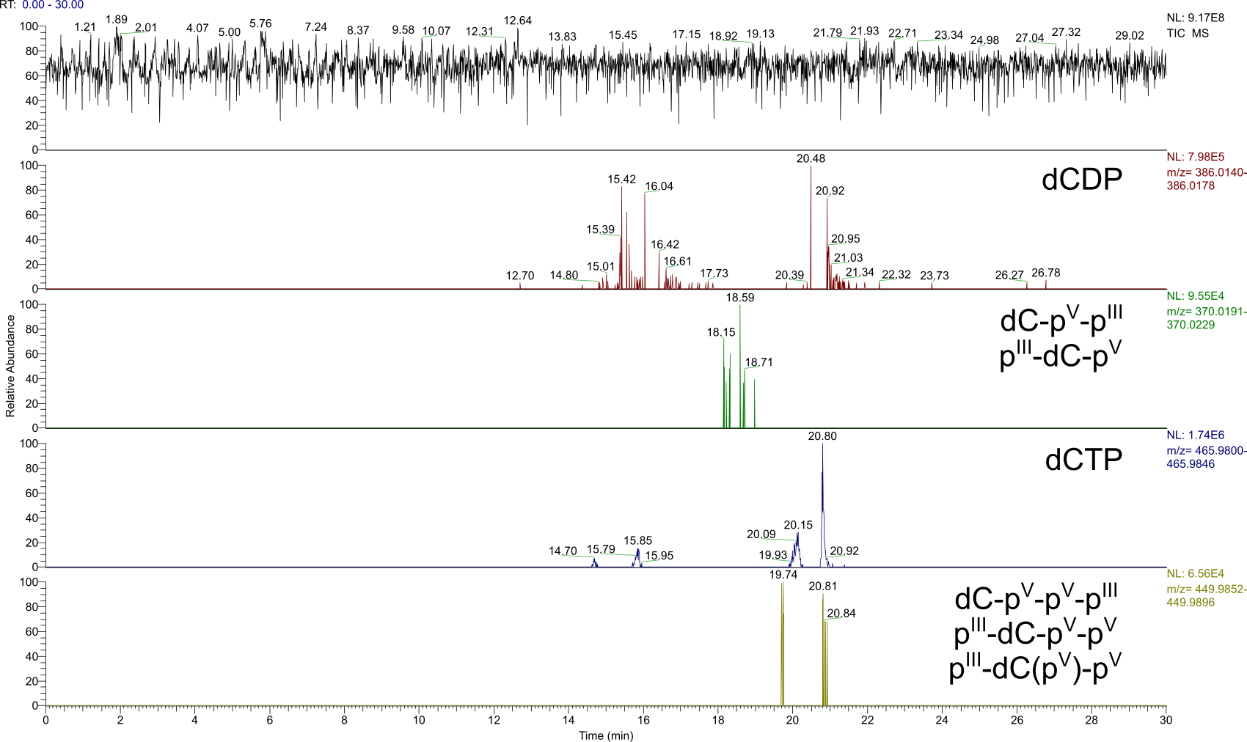


Supplementary Figure 60. TIC (m/z 50-750) and EIEs (Δ = 5.0 ppm) of the products of the reaction starting from dC (100 mM), H_3_PO_3_ (3.0 eq.) and urea (1.0 eq.) after 7 d. Constitution of potential isomers has not been determined. Labels illustrate all possible phosphate/phosphonate binding modes and refer to the entirety of all formed isomers.

### Reaction with deoxythymidine


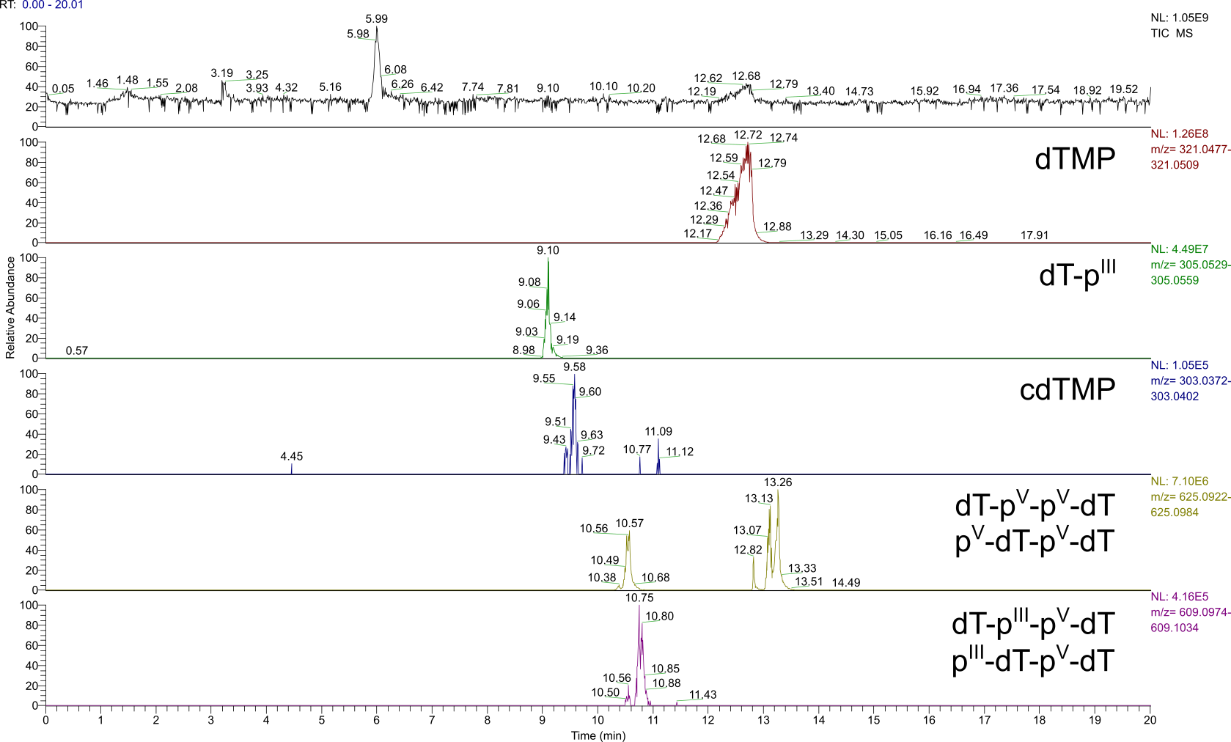


Supplementary Figure 61. TIC (m/z 80-1200) and EIEs (Δ = 5.0 ppm) of the products of the reaction starting from dT (100 mM), H_3_PO_3_ (3.0 eq.) and urea (1.0 eq.) after 7 d. Constitution of potential isomers has not been determined. Labels illustrate all possible phosphate/phosphonate binding modes and refer to the entirety of all formed isomers.


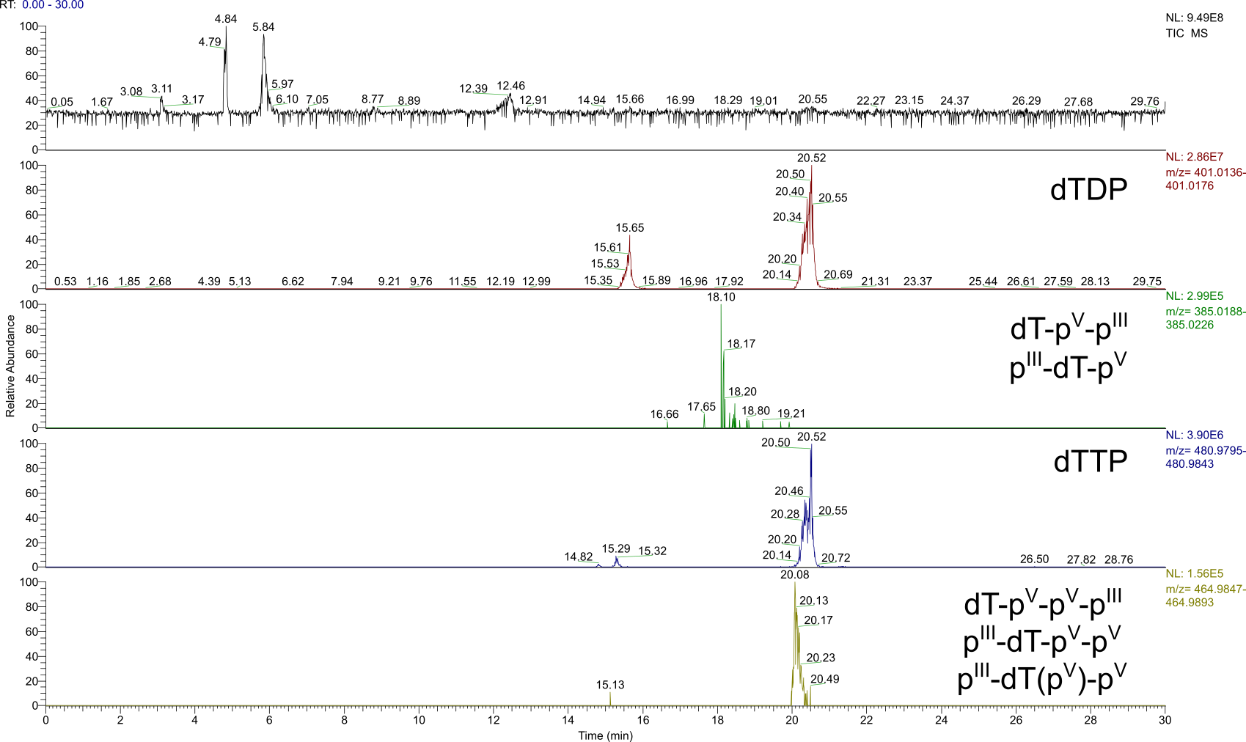


Supplementary Figure 62. TIC (m/z 80-1200) and EIEs (Δ = 5.0 ppm) of the products of the reaction starting from dT (100 mM), H_3_PO_3_ (3.0 eq.) and urea (1.0 eq.) after 7 d. Constitution of potential isomers has not been determined. Labels illustrate all possible phosphate/phosphonate binding modes and refer to the entirety of all formed isomers.


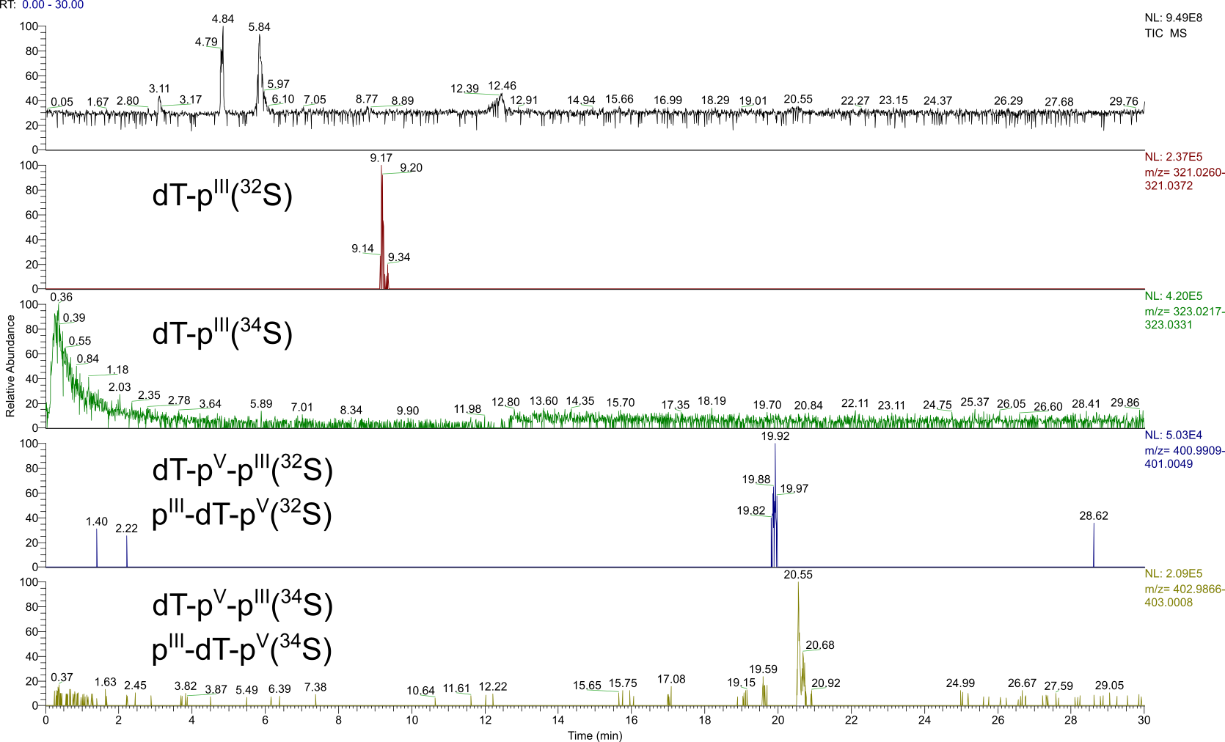


Supplementary Figure 63. TIC (m/z 80-1200) and EIEs (Δ = 17.5 ppm) of the thiophosphonate/-phosphate products of the reaction starting from dT (100 mM), H_3_PO_3_ (3.0 eq.) and urea (1.0 eq.) after 7 d. Constitution of potential isomers has not been determined. Labels illustrate all possible phosphate/phosphonate binding modes and refer to the entirety of all formed isomers.

### Reaction with glycerol


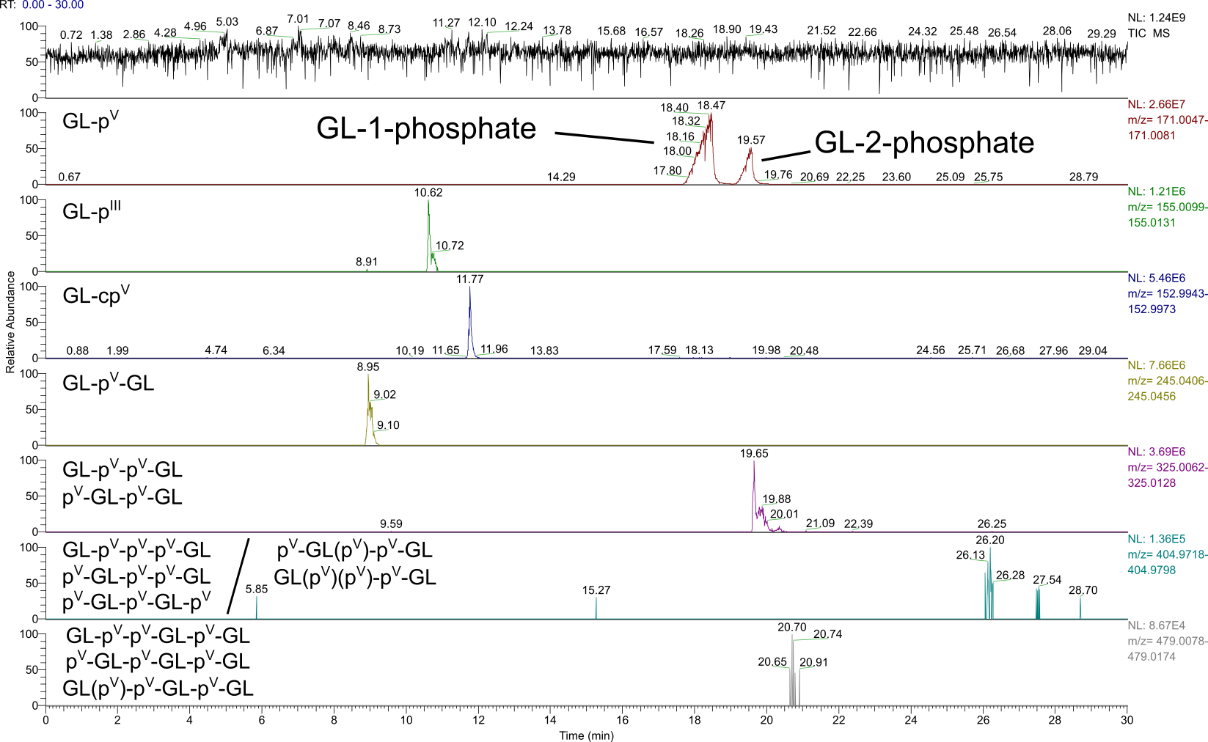


Supplementary Figure 64. TIC (m/z 50-750) and EIEs (Δ = 10.0 ppm) of the products of the reaction starting from glycerol (GL) (100 mM), H_3_PO_3_ (3.0 eq.) and urea (1.0 eq.) after 7 d. Signals of GL-1-phosphate and GL-2-phosphate were assigned by co-injection with reference compounds. The constitution of the other potential isomers has not been determined. Labels illustrate all possible phosphate/phosphonate binding modes and refer to the entirety of all formed isomers.


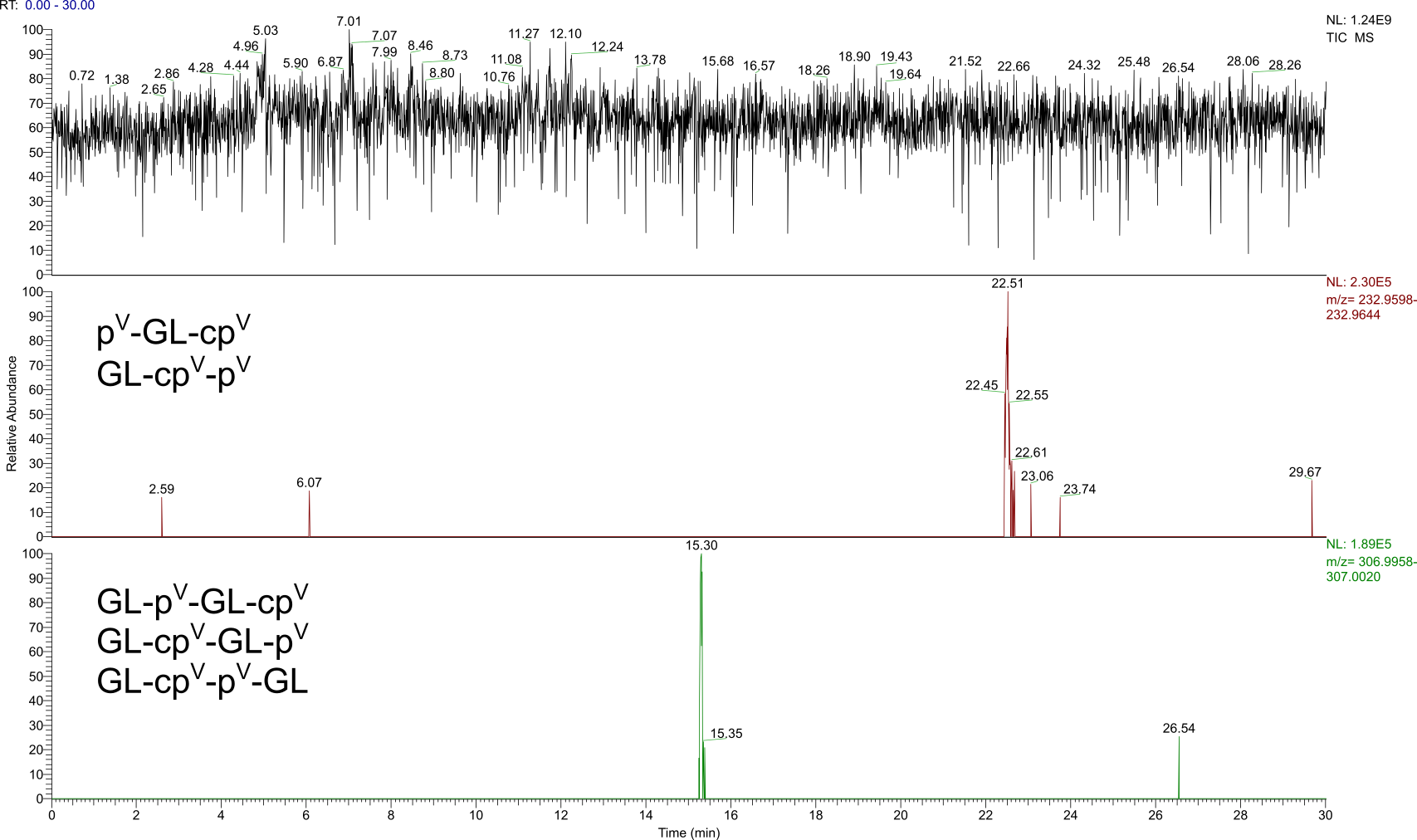


Supplementary Figure 65. TIC (m/z 50-750) and EIEs (Δ = 10.0 ppm) of the products of the reaction starting from GL (100 mM), H_3_PO_3_ (3.0 eq.) and urea (1.0 eq.) after 7 d. Constitution of potential isomers has not been determined. Labels illustrate all possible phosphate binding modes and refer to the entirety of all formed isomers.

### Reaction with glyceraldehyde


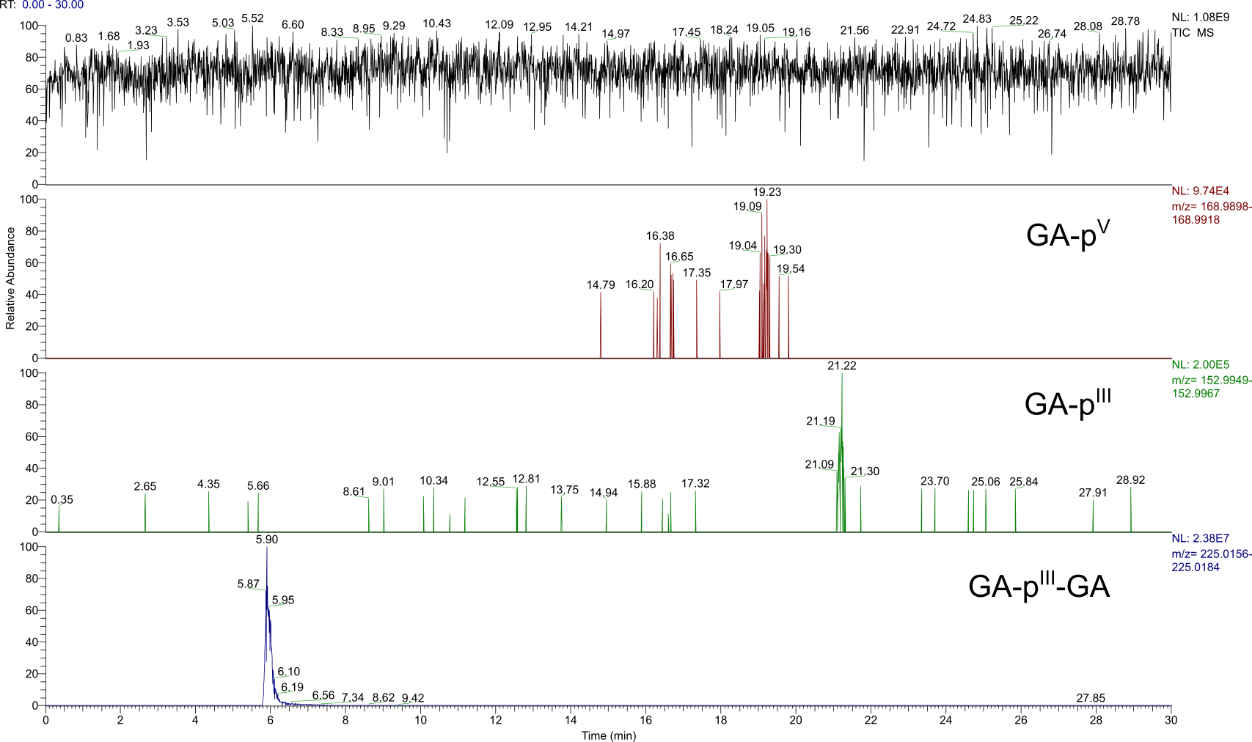


Supplementary Figure 66. TIC (m/z 50-750) and EIEs (Δ = 6.0 ppm) of the products of the reaction starting from glyceraldehyde (GA) (100 mM), H_3_PO_3_ (3.0 eq.) and urea (1.0 eq.) after 7 d. Constitution of potential isomers has not been determined. Labels illustrate all possible phosphate/phosphonate binding modes and refer to the entirety of all formed isomers.


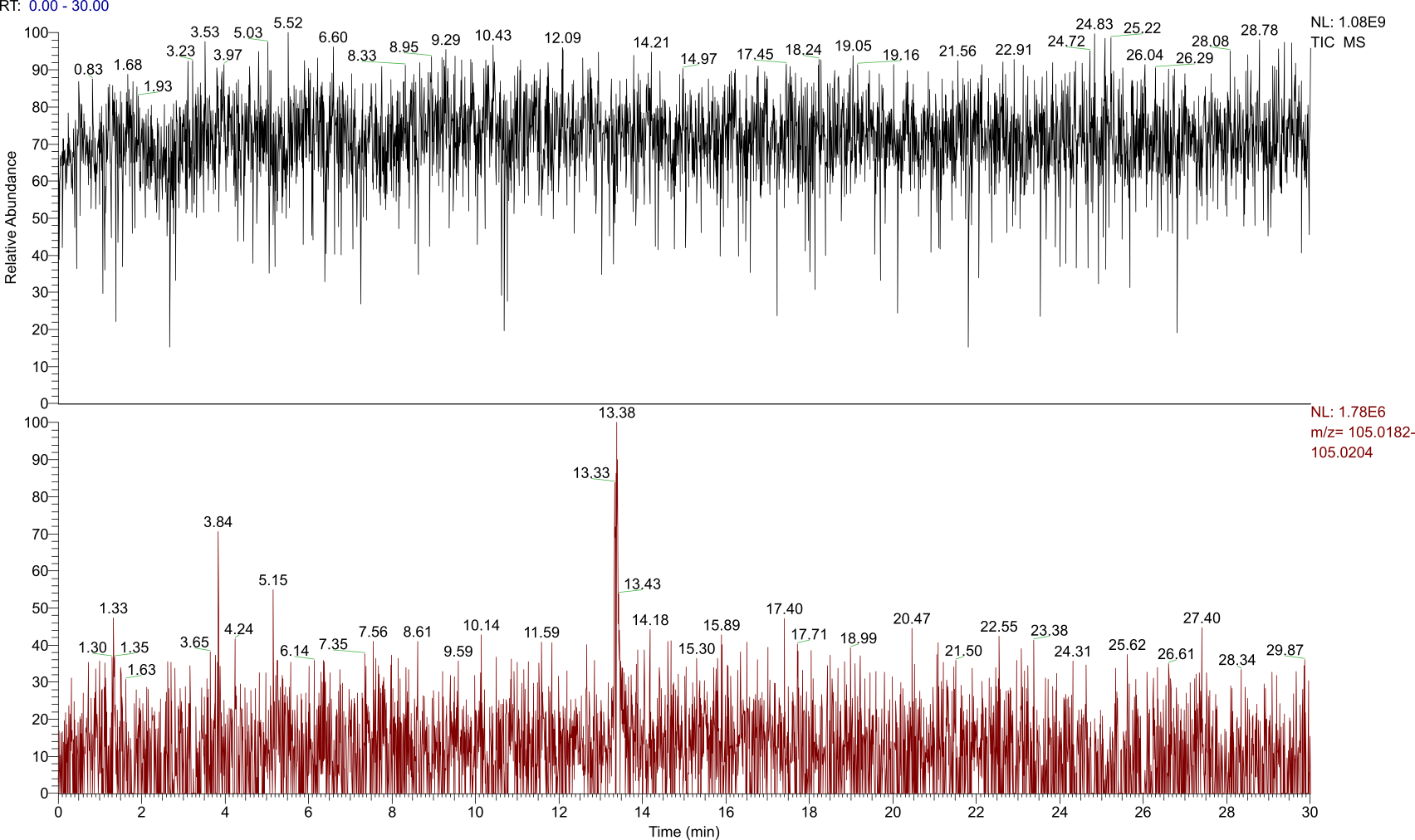


Supplementary Figure 67. TIC (m/z 50-750) and EIE (Δ = 10.0 ppm) of the GA oxidation product glyceric acid in the reaction starting from GA (100 mM), H_3_PO_3_ (3.0 eq.) and urea (1.0 eq.) after 7 d.

### Reaction with D-ribose


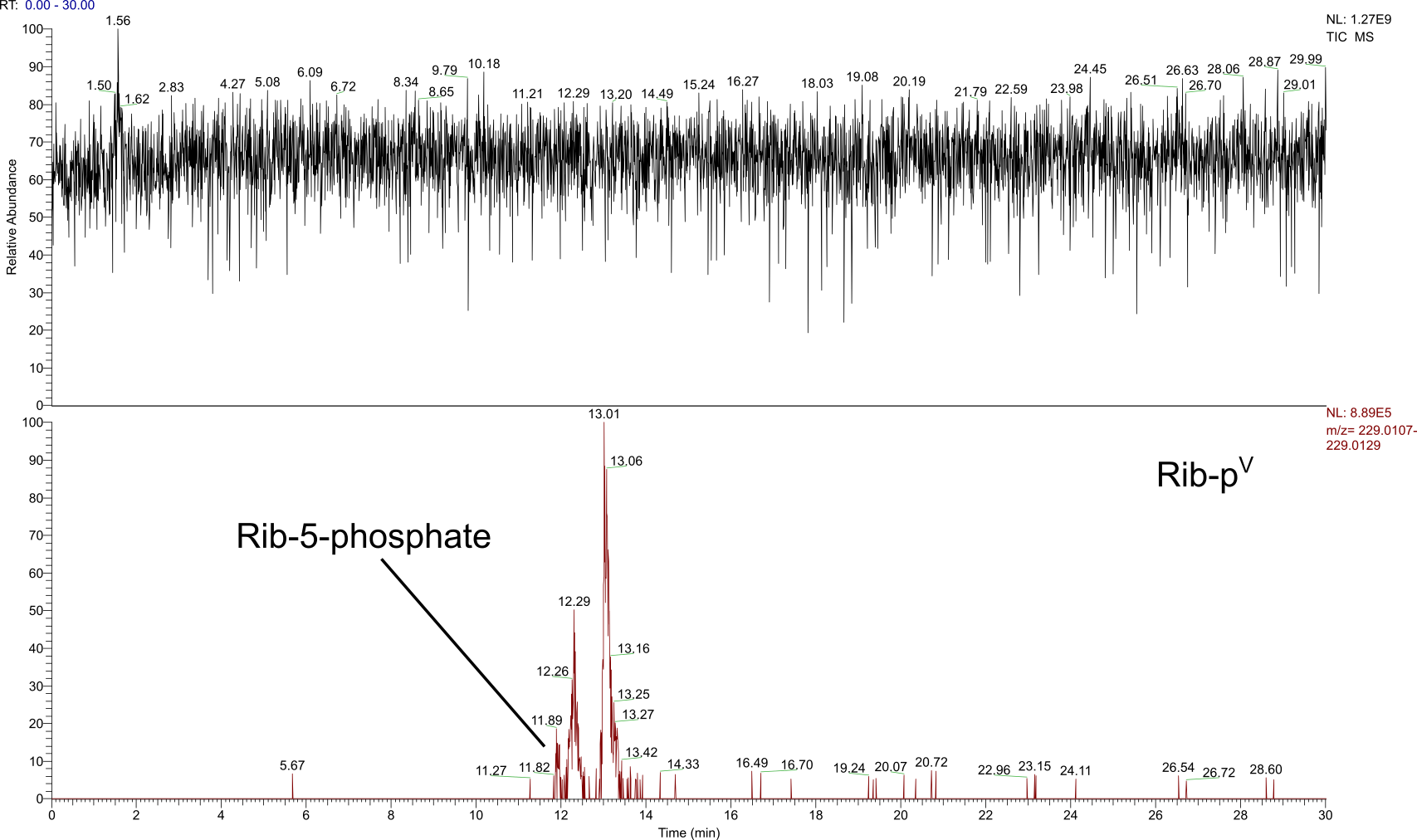


Supplementary Figure 68. TIC (m/z 50-750) and EIE (Δ = 5.0 ppm) of the products of the reaction starting from D-ribose (Rib) (100 mM), H_3_PO_3_ (3.0 eq.) and urea (1.0 eq.) after 7 d. The signal of Rib-5-phosphate was assigned by co-injection with a reference compound. The constitution of the other potential isomers has not been determined. The label illustrates all possible phosphate binding modes and refers to the entirety of all formed isomers.

### Reaction with sodium L-lactate


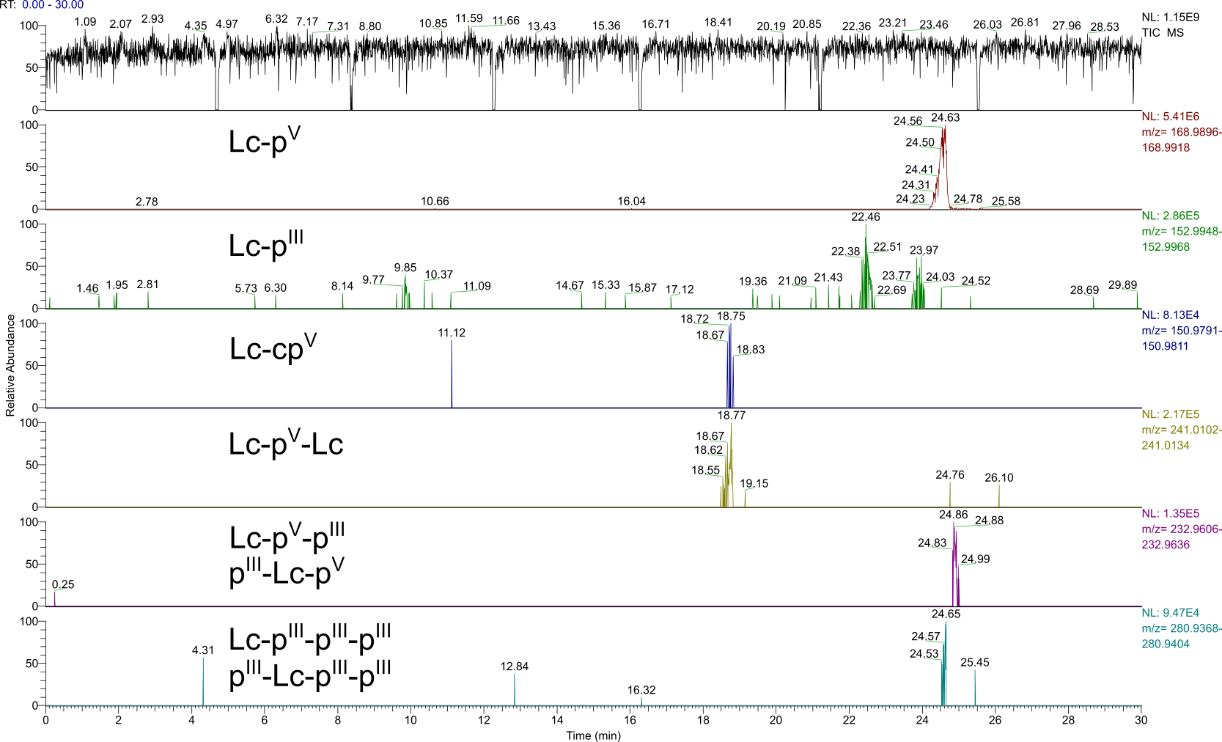


Supplementary Figure 69. TIC (m/z 50-750) and EIEs (Δ = 6.5 ppm) of the products of the reaction starting from sodium L-lactate (Lc) (100 mM), H_3_PO_3_ (30 eq.) and urea (1.0 eq.) after 7 d. Constitution of potential isomers has not been determined. Labels illustrate all possible phosphate/phosphonate binding modes and refer to the entirety of all formed isomers.

### Reaction with L-serine


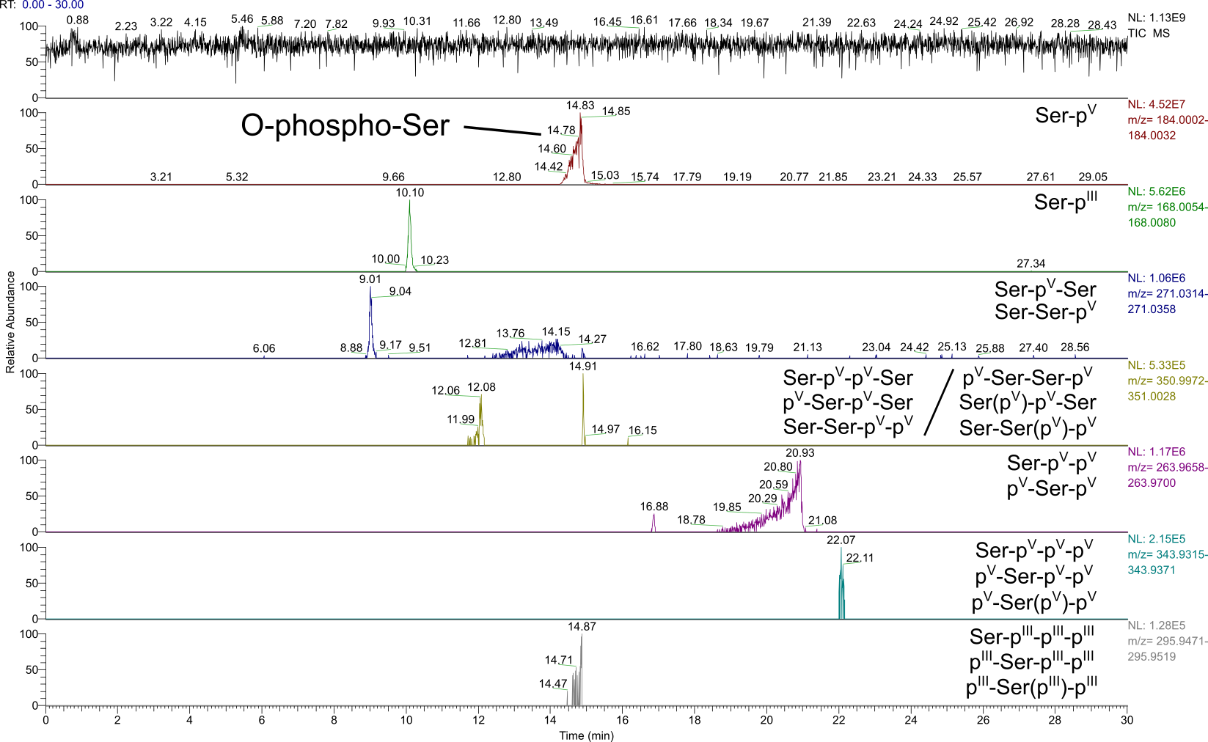


Supplementary Figure 70. TIC (m/z 50-750) and EIEs (Δ = 8.0 ppm) of the products of the reaction starting from L-serine (Ser) (100 mM), H_3_PO_3_ (3.0 eq.) and urea (1.0 eq.) after 7 d. The signal of O-phospho-Ser was assigned by co-injection with a reference compound. The constitution of the other potential isomers has not been determined. Labels illustrate all possible phosphate/phosphonate binding modes and refer to the entirety of all formed isomers.


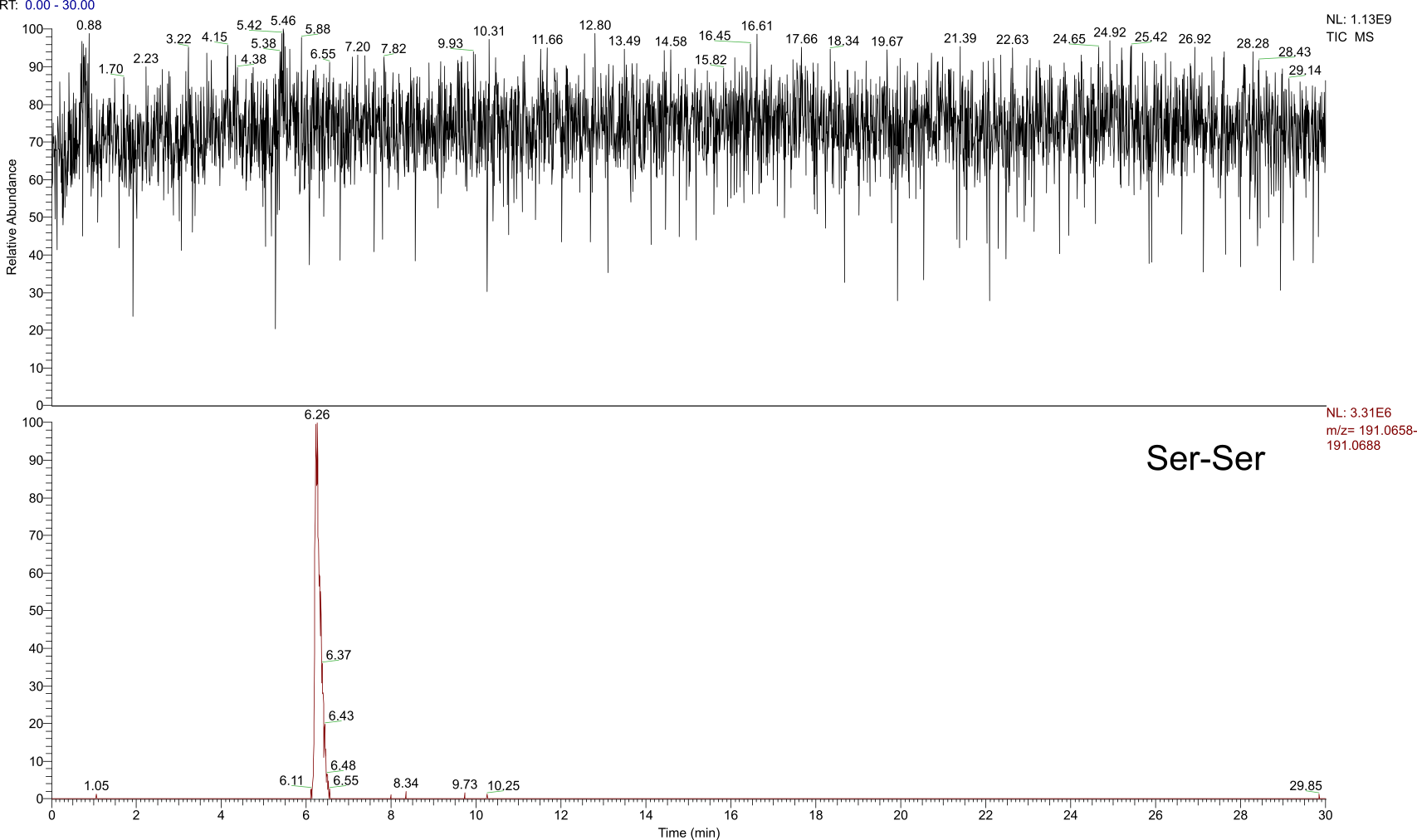


Supplementary Figure 71. TIC (m/z 50-750) and EIE (Δ = 8.0 ppm) of the dipeptide product of the reaction starting from Ser (100 mM), H_3_PO_3_ (3.0 eq.) and urea (1.0 eq.) after 7 d.

### Reaction of the complete nucleoside mixture


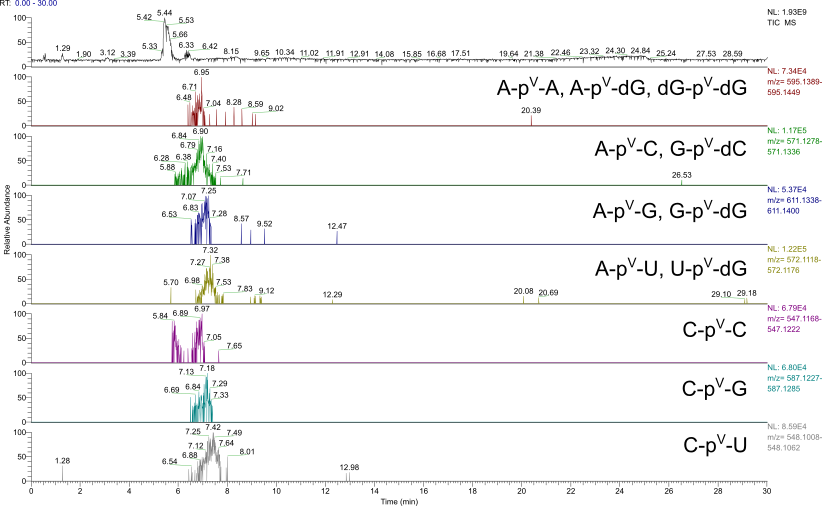


Supplementary Figure 72: TIC (m/z 80-1200) and EIEs (Δ = 5.0 ppm) of the products of the reaction starting from A, C, G, U, dA, dC, dG and dT (each 25 mM, in total 1.0 eq.) with H_3_PO_3_ (3.0 eq.) and urea (1.0 eq.) after 7 d. Constitution of potential isomers has not been determined. Labels illustrate all possible phosphate binding modes and refer to the entirety of all formed isomers (including inverse deoxyribo- and ribonucleoside combinations, e. g. G-dC and dG-C).


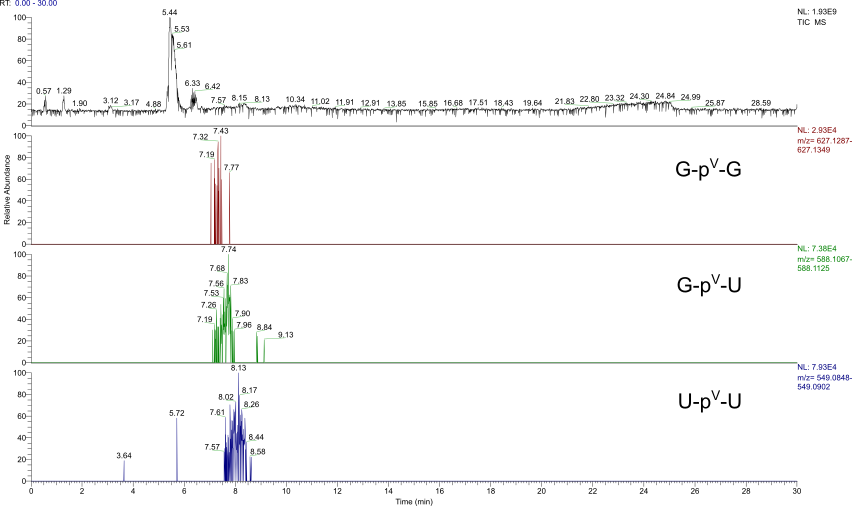


Supplementary Figure 73. TIC (m/z 80-1200) and EIEs (Δ = 5.0 ppm) of the products of the reaction starting from A, C, G, U, dA, dC, dG and dT (each 25 mM, in total 1.0 eq.) with H_3_PO_3_ (3.0 eq.) and urea (1.0 eq.) after 7 d. Constitution of potential isomers has not been determined. Labels illustrate all possible phosphate binding modes and refer to the entirety of all formed isomers.


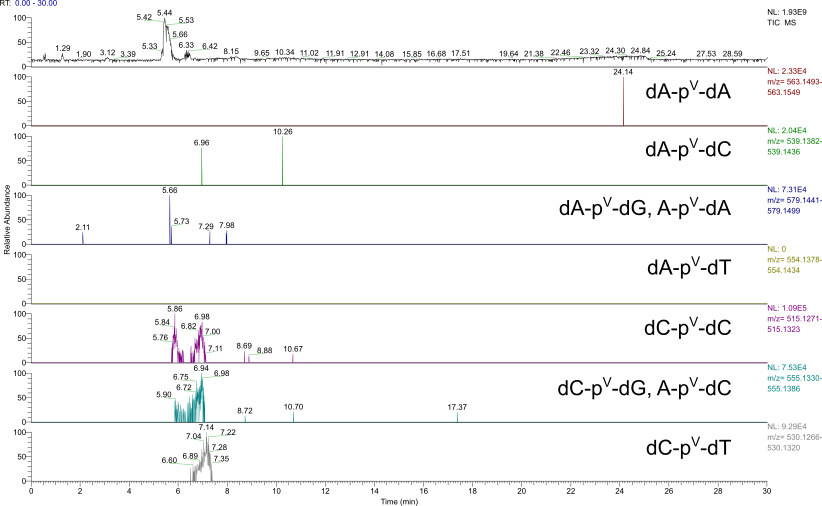


Supplementary Figure 74. TIC (m/z 80-1200) and EIEs (Δ = 5.0 ppm) of the products of the reaction starting from A, C, G, U, dA, dC, dG and dT (each 25 mM, in total 1.0 eq.) with H_3_PO_3_ (3.0 eq.) and urea (1.0 eq.) after 7 d. Constitution of potential isomers has not been determined. Labels illustrate all possible phosphate binding modes and refer to the entirety of all formed isomers (including inverse deoxyribo- and ribonucleoside combinations, e. g. A-dC and dA-C).


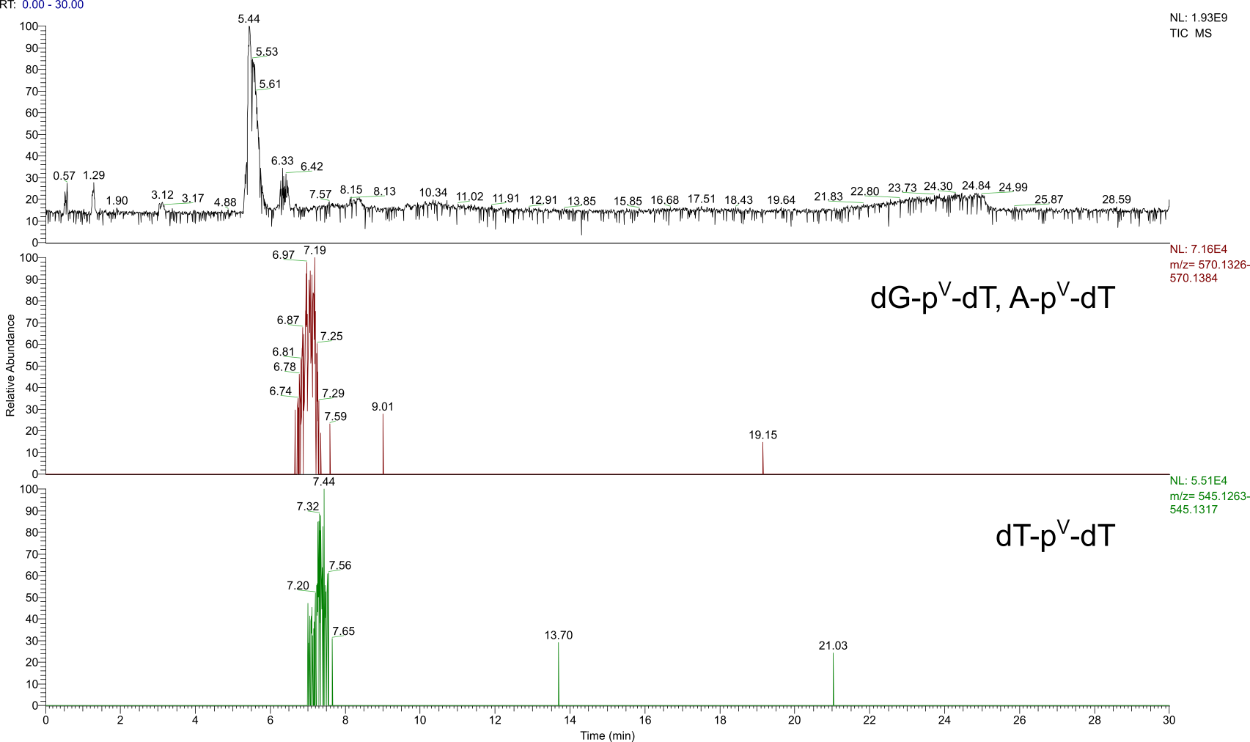


Supplementary Figure 75. TIC (m/z 80-1200) and EIEs (Δ = 5.0 ppm) of the products of the reaction starting from A, C, G, U, dA, dC, dG and dT (each 25 mM, in total 1.0 eq.) with H_3_PO_3_ (3.0 eq.) and urea (1.0 eq.) after 7 d. Constitution of potential isomers has not been determined. Labels illustrate all possible phosphate binding modes and refer to the entirety of all formed isomers.


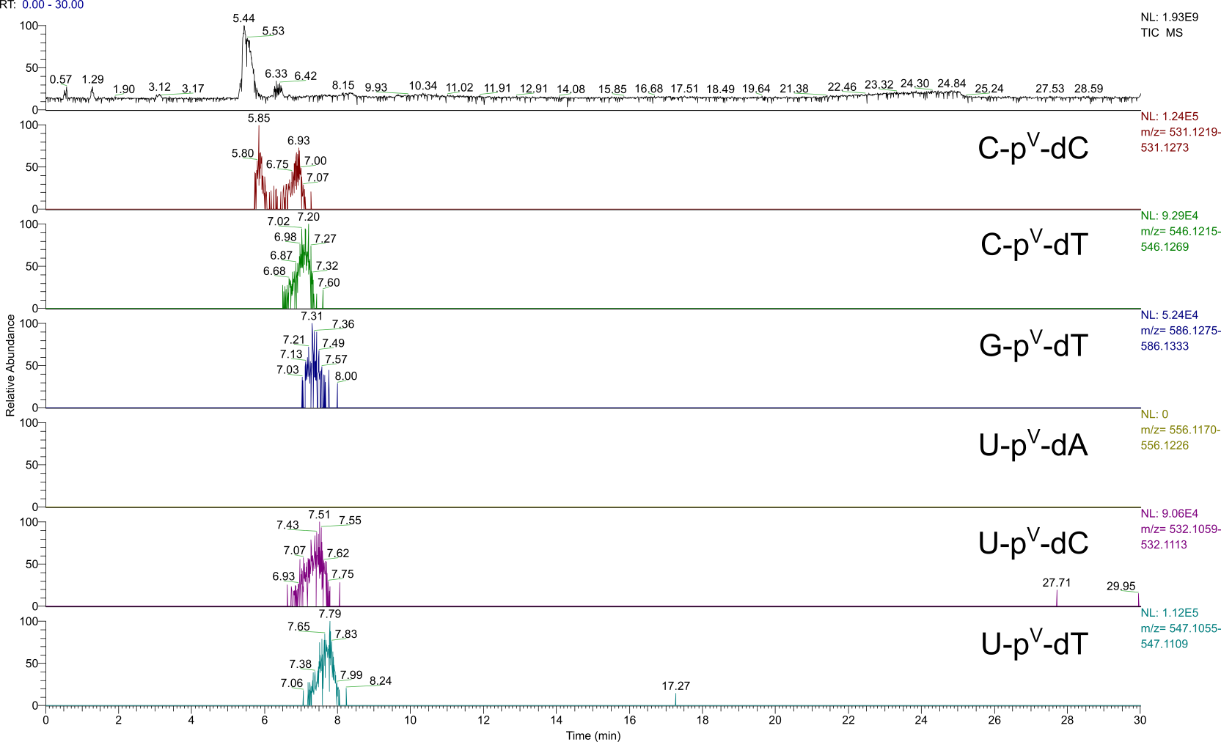


Supplementary Figure 76. TIC (m/z 80-1200) and EIEs (Δ = 5.0 ppm) of the products of the reaction starting from A, C, G, U, dA, dC, dG and dT (each 25 mM, in total 1.0 eq.) with H_3_PO_3_ (3.0 eq.) and urea (1.0 eq.) after 7 d. Constitution of potential isomers has not been determined. Labels illustrate all possible phosphate binding modes and refer to the entirety of all formed isomers.


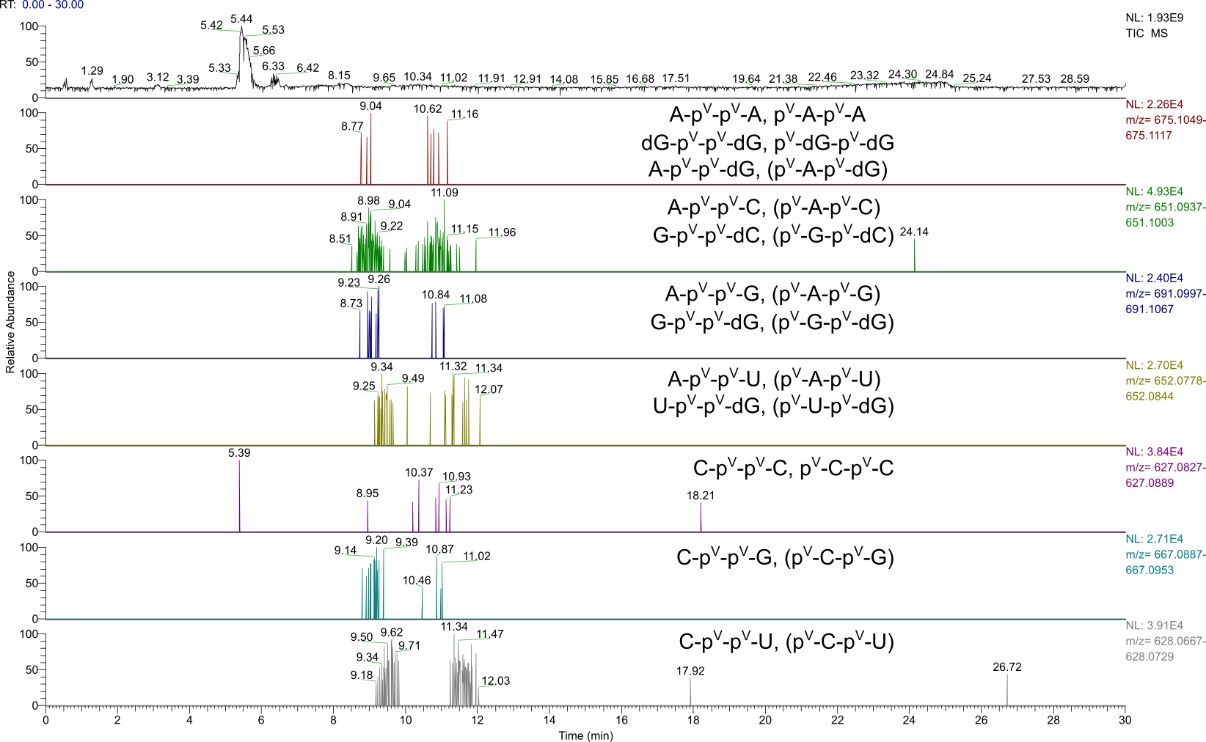


Supplementary Figure 77. TIC (m/z 80-1200) and EIEs (Δ = 5.0 ppm) of the products of the reaction starting from A, C, G, U, dA, dC, dG and dT (each 25 mM, in total 1.0 eq.) with H_3_PO_3_ (3.0 eq.) and urea (1.0 eq.) after 7 d. Constitution of potential isomers has not been determined. Labels in brackets indicate that an inverse nucleoside sequence is possible (e. g. A-C and C-A). Labels illustrate all possible phosphate binding modes and refer to the entirety of all formed isomers (including inverse deoxyribo- and ribonucleoside combinations, e. g. G-dC and dG-C).


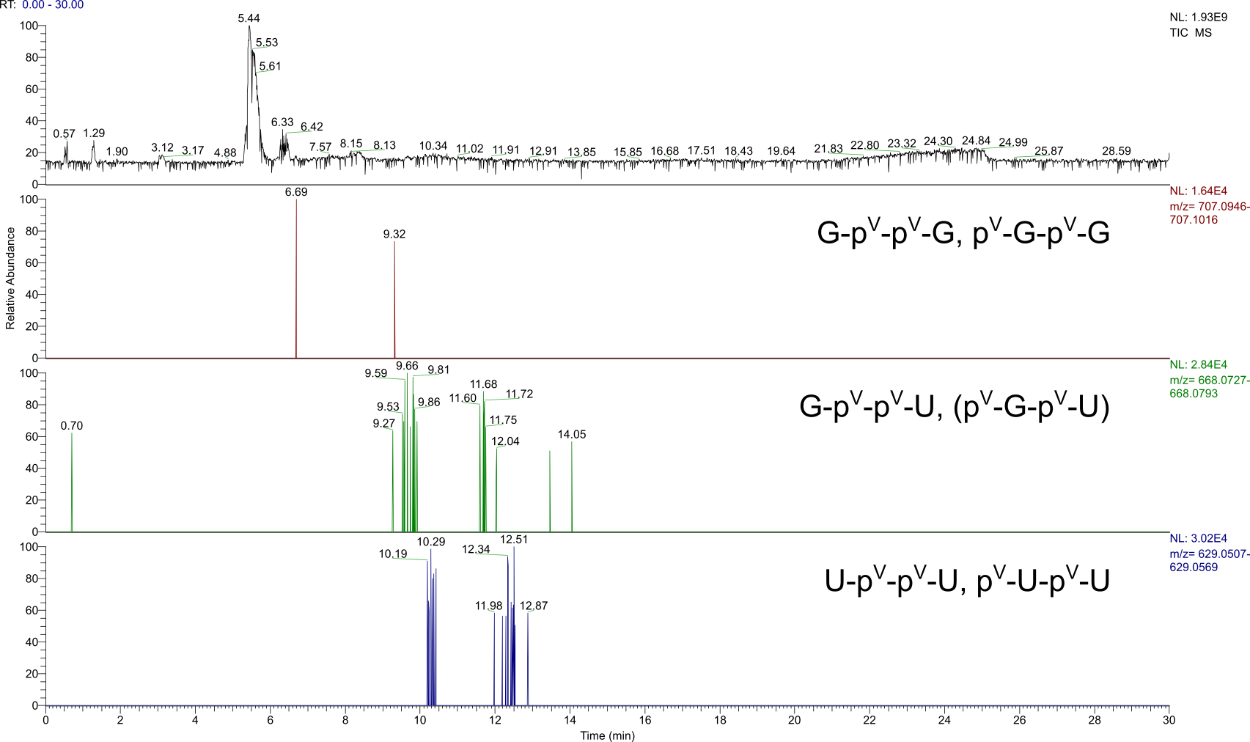


Supplementary Figure 78. TIC (m/z 80-1200) and EIEs (Δ = 5.0 ppm) of the products of the reaction starting from A, C, G, U, dA, dC, dG and dT (each 25 mM, in total 1.0 eq.) with H_3_PO_3_ (3.0 eq.) and urea (1.0 eq.) after 7 d. Constitution of potential isomers has not been determined. Labels in brackets indicate that an inverse nucleoside sequence is possible (e.g. G-U and U-G). Labels illustrate all possible phosphate binding modes and refer to the entirety of all formed isomers.


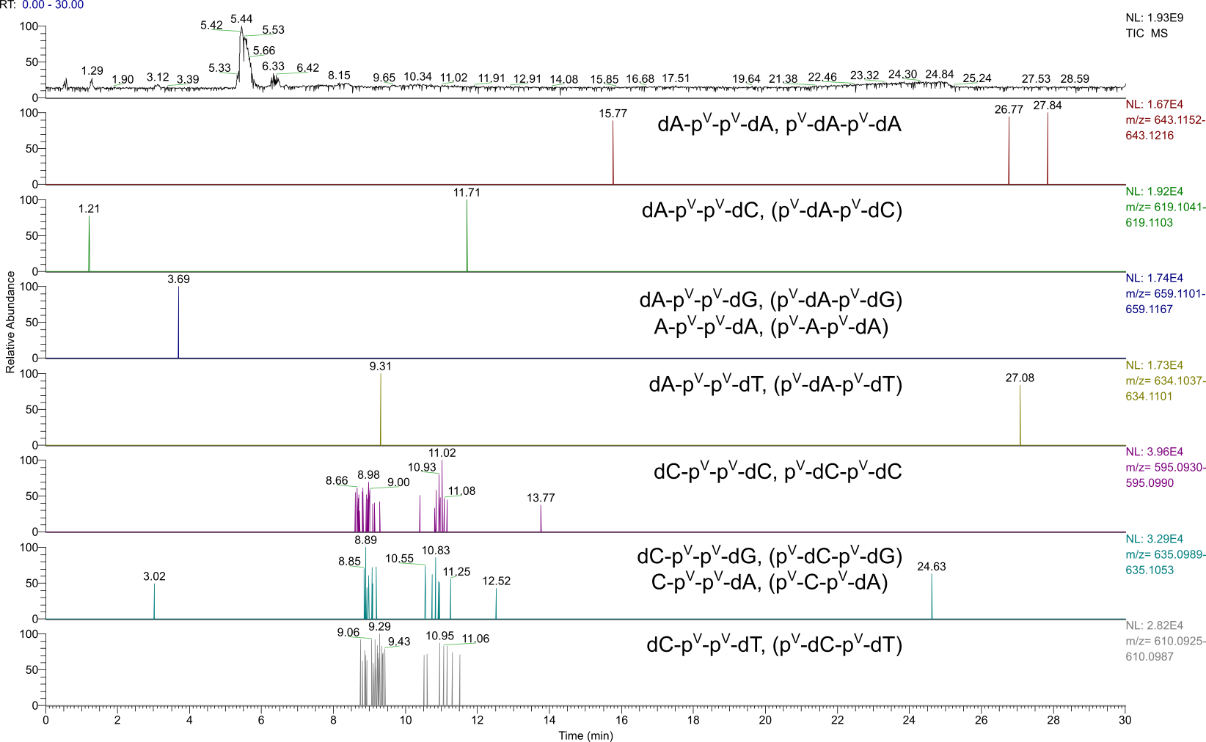


Supplementary Figure 79. TIC (m/z 80-1200) and EIEs (Δ = 5.0 ppm) of the products of the reaction starting from A, C, G, U, dA, dC, dG and dT (each 25 mM, in total 1.0 eq.) with H_3_PO_3_ (3.0 eq.) and urea (1.0 eq.) after 7 d. Constitution of potential isomers has not been determined. Labels in brackets indicate that an inverse nucleoside sequence is possible (e. g. dA-dC and dC-dA). Labels illustrate all possible phosphate binding modes and refer to the entirety of all formed isomers (including inverse deoxyribo- and ribonucleoside combinations, e. g. C-dA and dC-A).


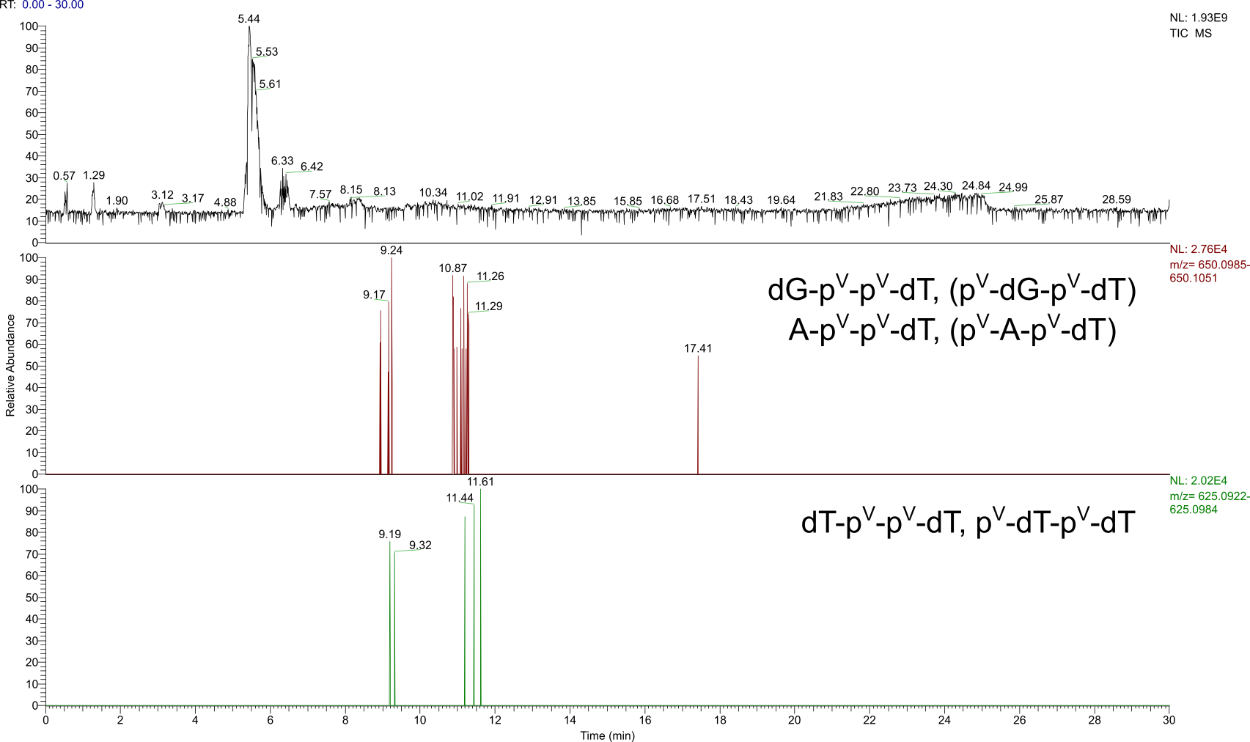


Supplementary Figure 80. TIC (m/z 80-1200) and EIEs (Δ = 5.0 ppm) of the products of the reaction starting from A, C, G, U, dA, dC, dG and dT (each 25 mM, in total 1.0 eq.) with H_3_PO_3_ (3.0 eq.) and urea (1.0 eq.) after 7 d. Constitution of potential isomers has not been determined. Labels in brackets indicate that an inverse nucleoside sequence is possible (e. g. dG-dT and dT-dG). Labels illustrate all possible phosphate binding modes and refer to the entirety of all formed isomers.


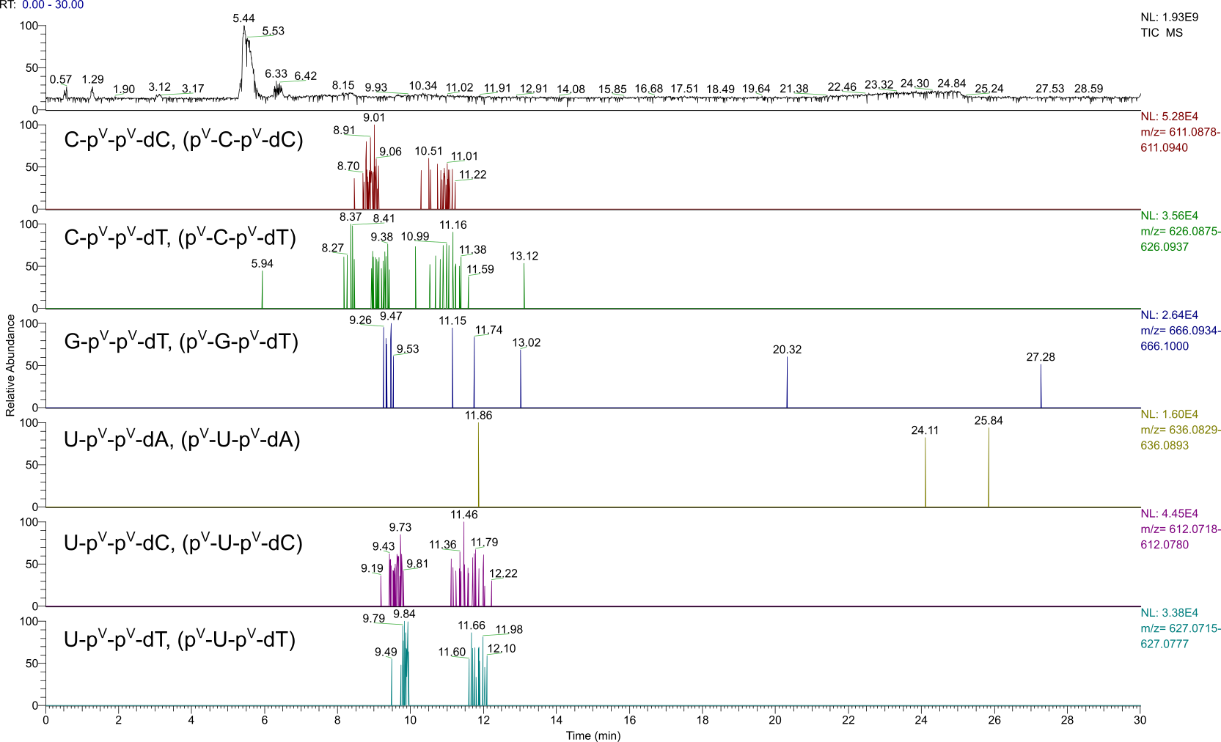


Supplementary Figure 81. TIC (m/z 80-1200) and EIEs (Δ = 5.0 ppm) of the products of the reaction starting from A, C, G, U, dA, dC, dG and dT (each 25 mM, in total 1.0 eq.) with H_3_PO_3_ (3.0 eq.) and urea (1.0 eq.) after 7 d. Constitution of potential isomers has not been determined. Labels in brackets indicate that an inverse nucleoside sequence is possible (e. g. C-dT and dT-C). Labels illustrate all possible phosphate binding modes and refer to the entirety of all formed isomers
