# Supplementary material for: Phosphorylation in liquid sulfur dioxide under prebiotically plausible conditions
Source: Commun Chem. 2022 Nov 3;5:143. doi: 10.1038/s42004-022-00761-w (PMC9814524; doi:10.1038/s42004-022-00761-w)
Supplement: Supplementary file 5 — Supplementary Data 2 [file 42004_2022_761_MOESM5_ESM.docx]

# Supplementary Data 2

## MS/MS Spectra of p-N-p-N and N-p-p-N species


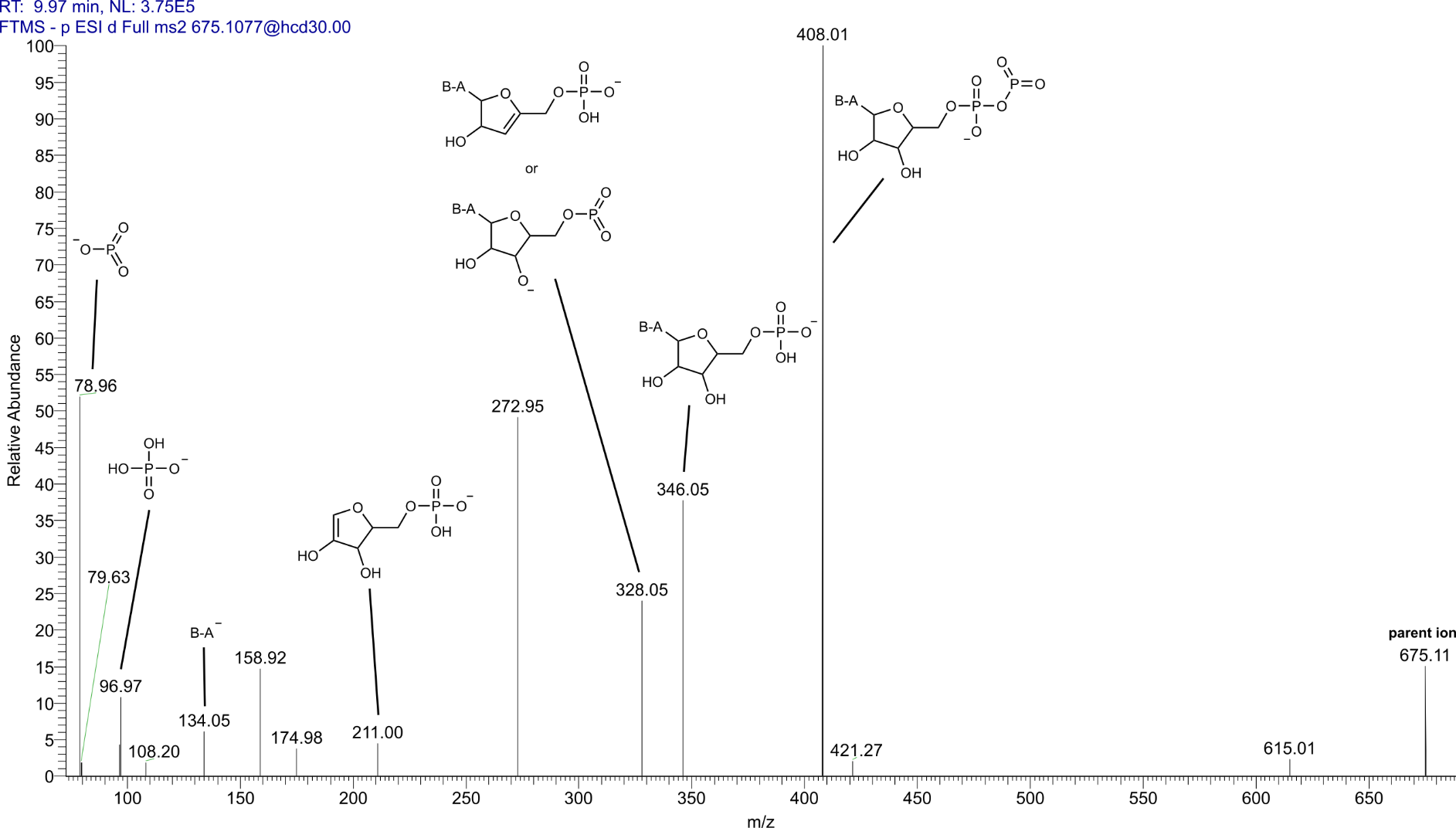


Supplementary Figure 1. MS/MS spectrum of the A based dinucleotide (m/z 675.1083) obtained from the reaction of A (100 mM) with H_3_PO_3_ (3.0 eq.) for 7 d. Collision induced dissociation (NCE 30%) of the parent ion at t_mig_ = 9.97 min after CE separation led to the assigned fragments. Displayed fragment structures have model character since there are multiple possible isomers (B-A: adenine).


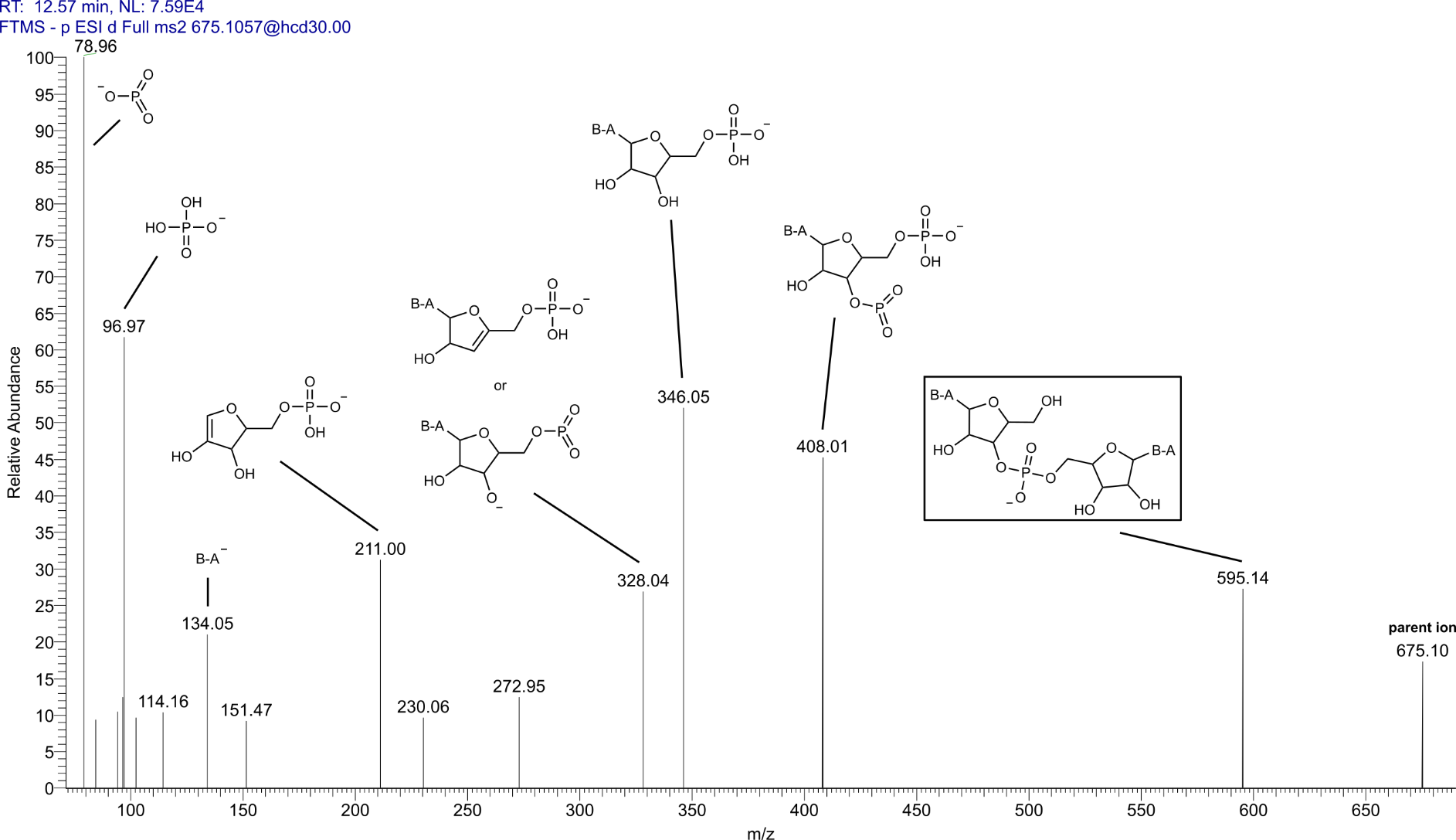


Supplementary Figure 2. MS/MS spectrum of the dinucleotide (m/z 675.1083) obtained from the reaction of A (100 mM) with H_3_PO_3_ (3.0 eq.) for 7 d. Collision induced dissociation (NCE 30%) at t_mig_ = 12.57 min led to the assigned fragments. Fragments in boxes are characteristic for alternating A and phosphate units. Displayed structures have model character since there are multiple possible isomers.


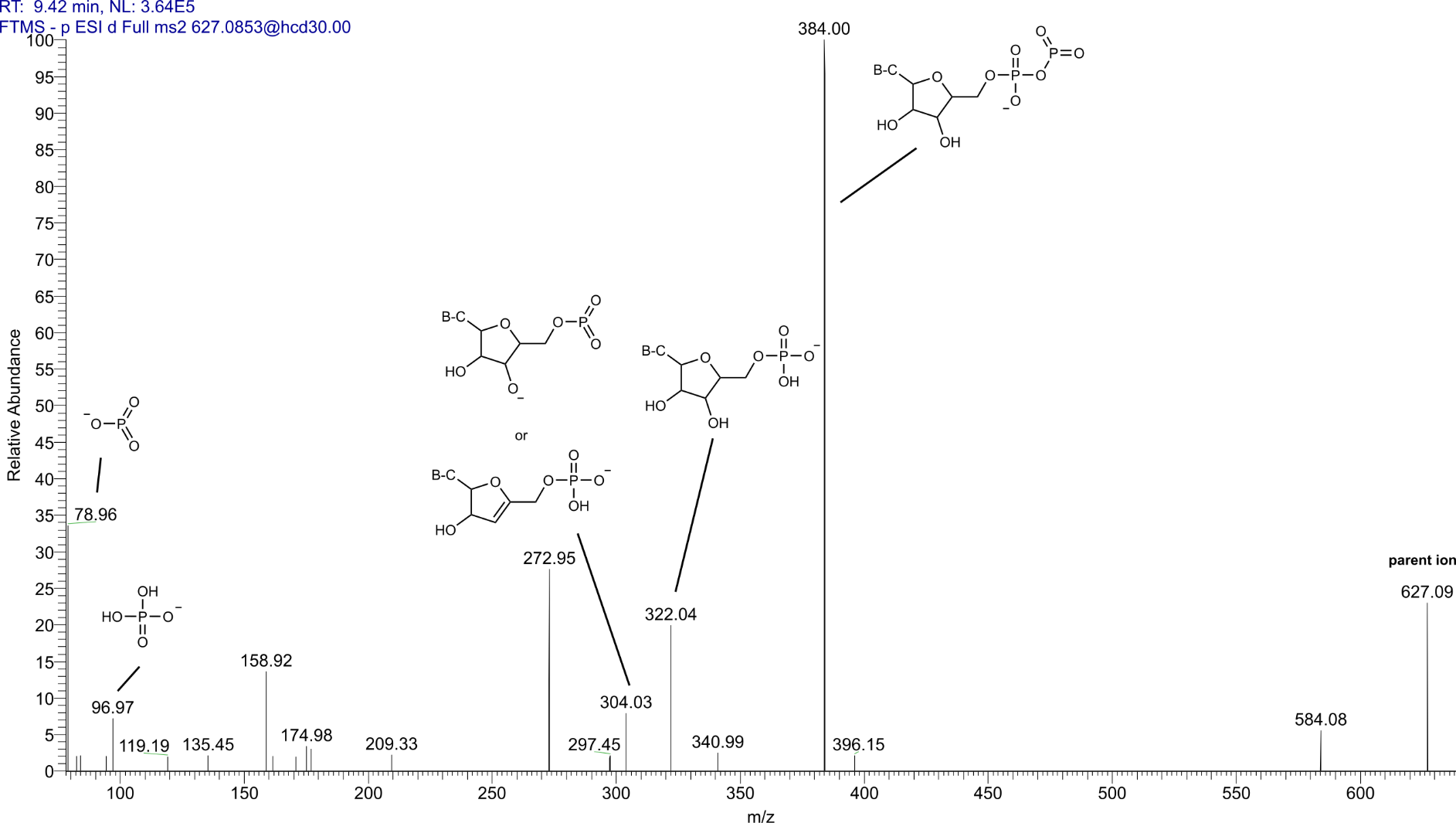


Supplementary Figure 3. MS/MS spectrum of the C based dinucleotide (m/z 627.0858) obtained from the reaction of C (100 mM) with H_3_PO_3_ (3.0 eq.) and urea (1.0 eq.) for 7 d. Collision induced dissociation (NCE 30%) of the parent ion at t_mig_ = 9.42 min after CE separation led to the assigned fragments. Displayed fragment structures have model character since there are multiple possible isomers (B-C: cytosine).


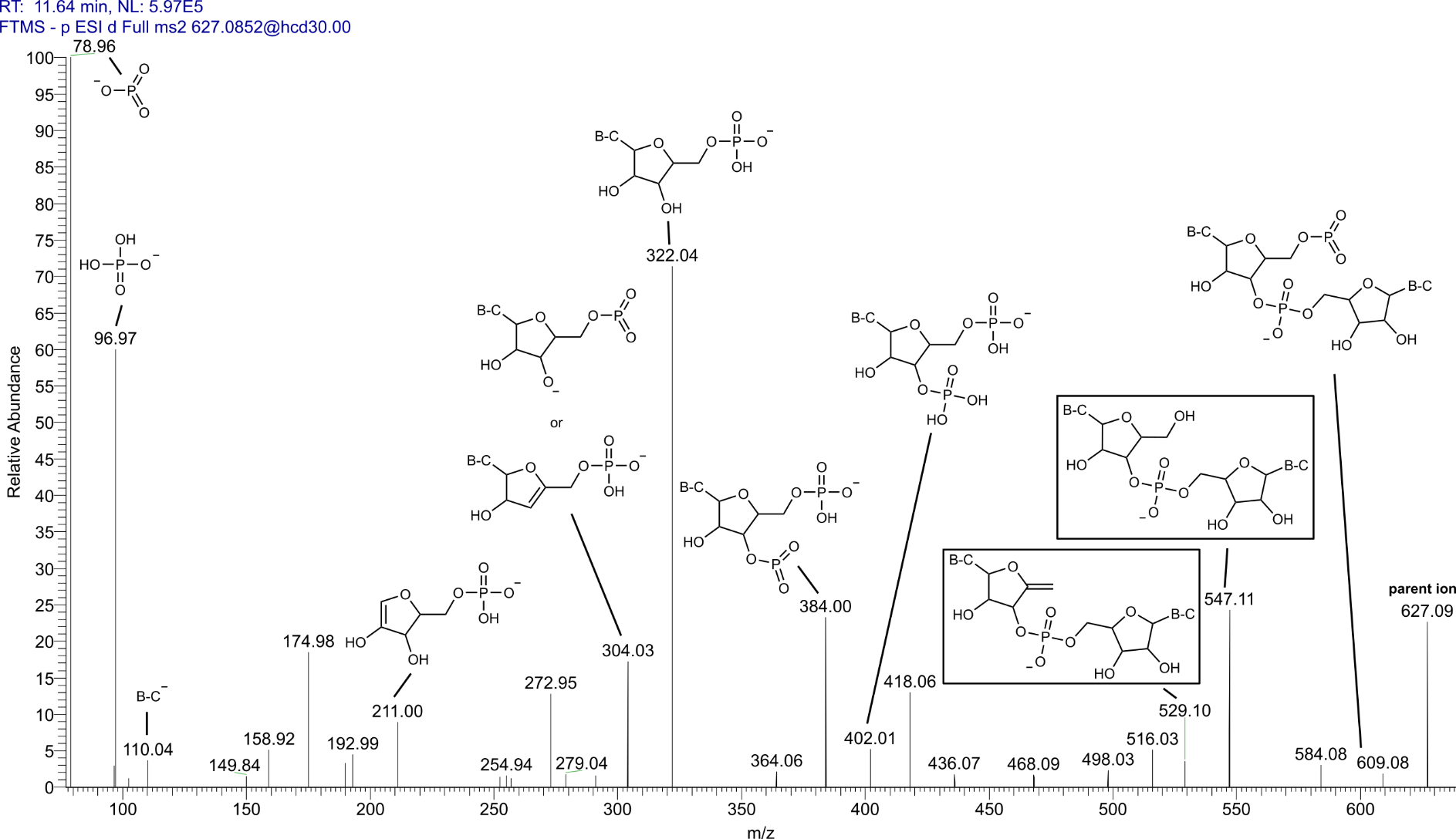


Supplementary Figure 4. MS/MS spectrum of the C based dinucleotide (m/z 627.0858) obtained from the reaction of C (100 mM) with H_3_PO_3_ (3.0 eq.) and urea (1.0 eq.) for 7 d. Collision induced dissociation (NCE 30%) of the parent ion at t_mig_ = 11.64 min after CE separation led to the assigned fragments. Fragments in boxes are characteristic for alternating C and phosphate units. Displayed fragment structures have model character since there are multiple possible isomers.


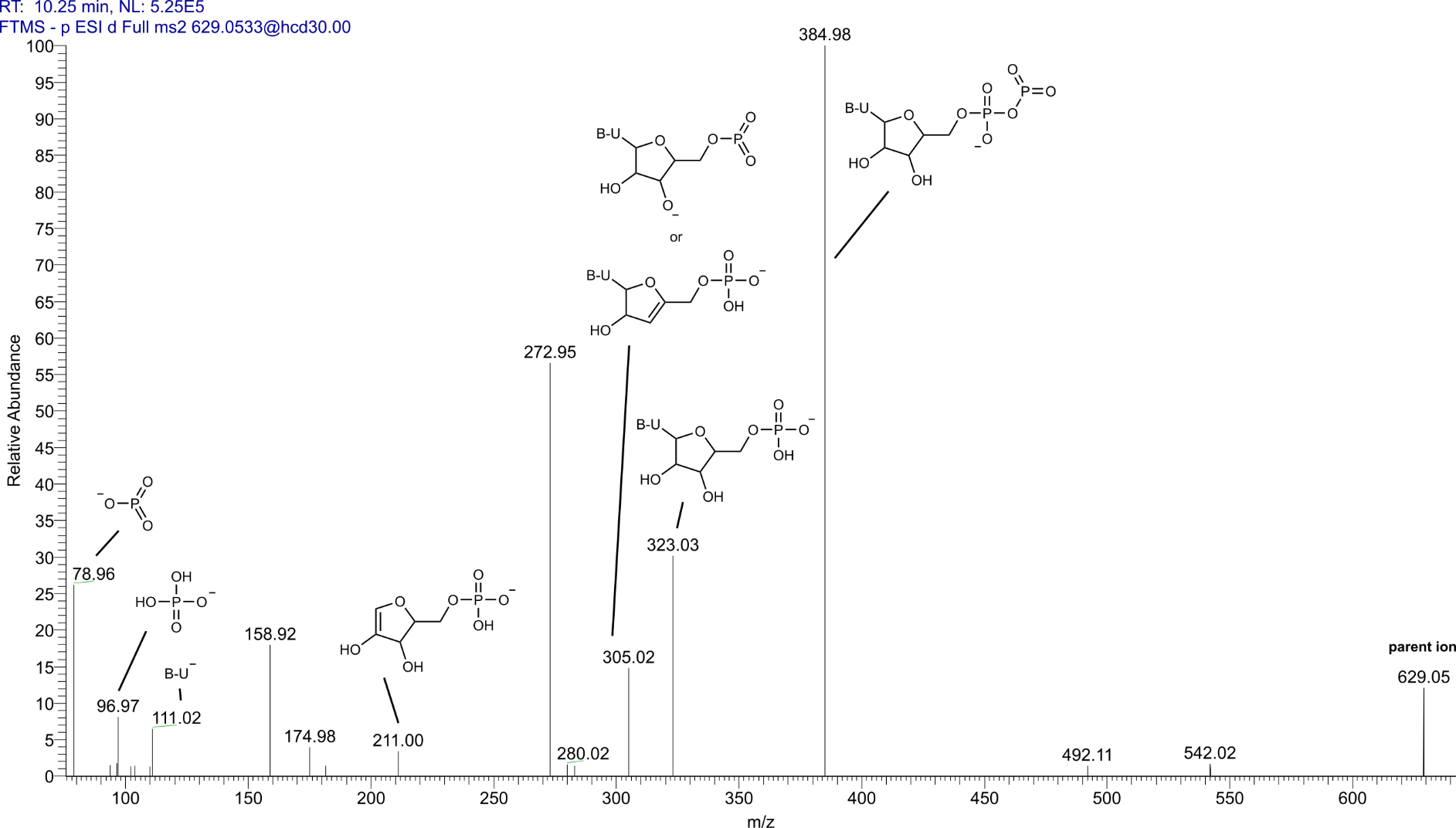


Supplementary Figure 5. MS/MS spectrum of the U based dinucleotide (m/z 629.0538) obtained from the reaction of U (100 mM) with H_3_PO_3_ (3.0 eq.) and urea (1.0 eq.) for 7 d. Collision induced dissociation (NCE 30%) of the parent ion at t_mig_ = 10.25 min after CE separation led to the assigned fragments. Displayed fragment structures have model character since there are multiple possible isomers
(B-U: uracil).


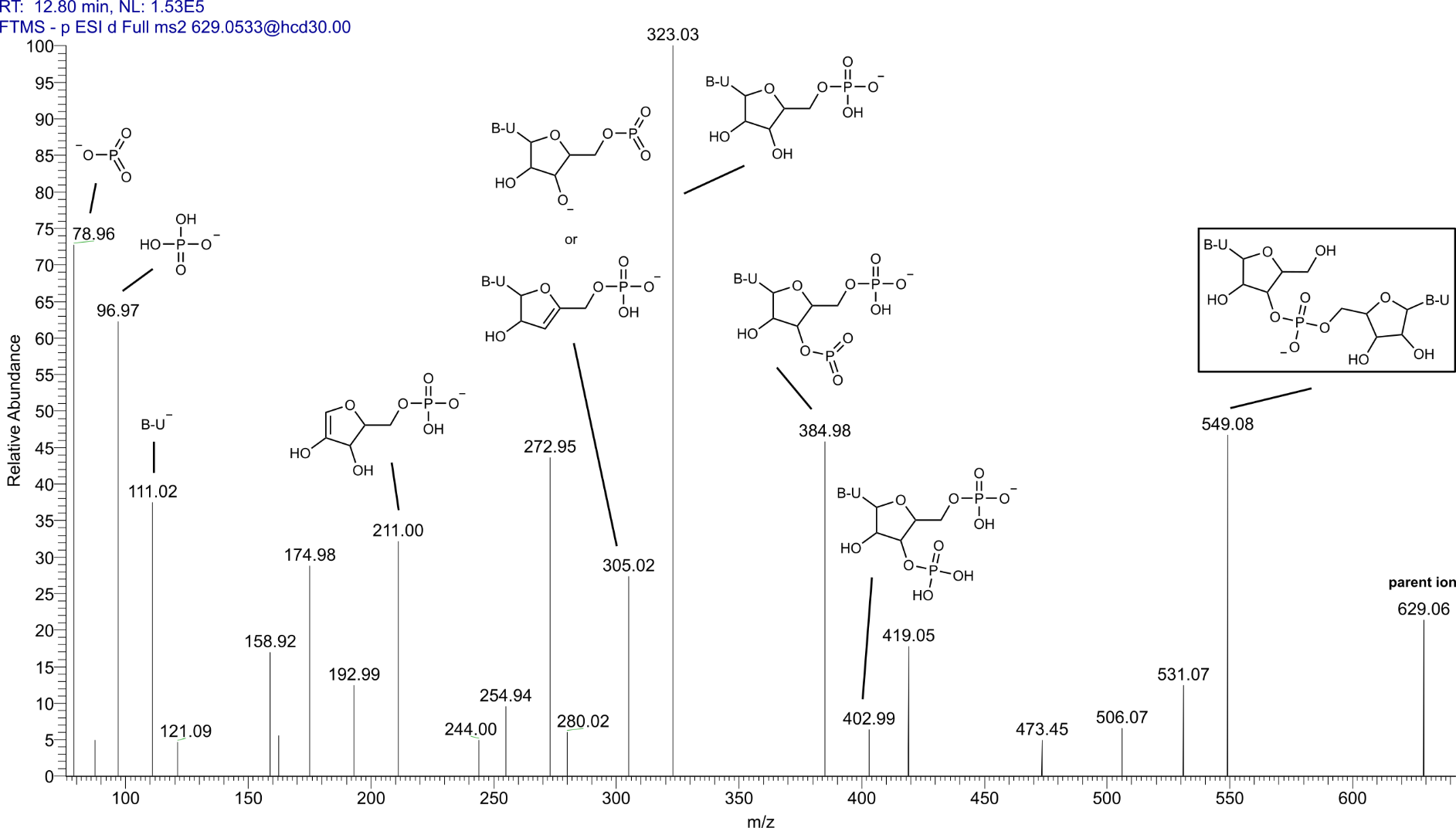


Supplementary Figure 6. MS/MS spectrum of the U based dinucleotide (m/z 629.0538) obtained from the reaction of U (100 mM) with H_3_PO_3_ (3.0 eq.) and urea (1.0 eq.) for 7 d. Collision induced dissociation (NCE 30%) of the parent ion at t_mig_ = 12.80 min after CE separation led to the assigned fragments. Fragments in boxes are characteristic for alternating U and phosphate units. Displayed fragment structures have model character since there are multiple possible isomers.


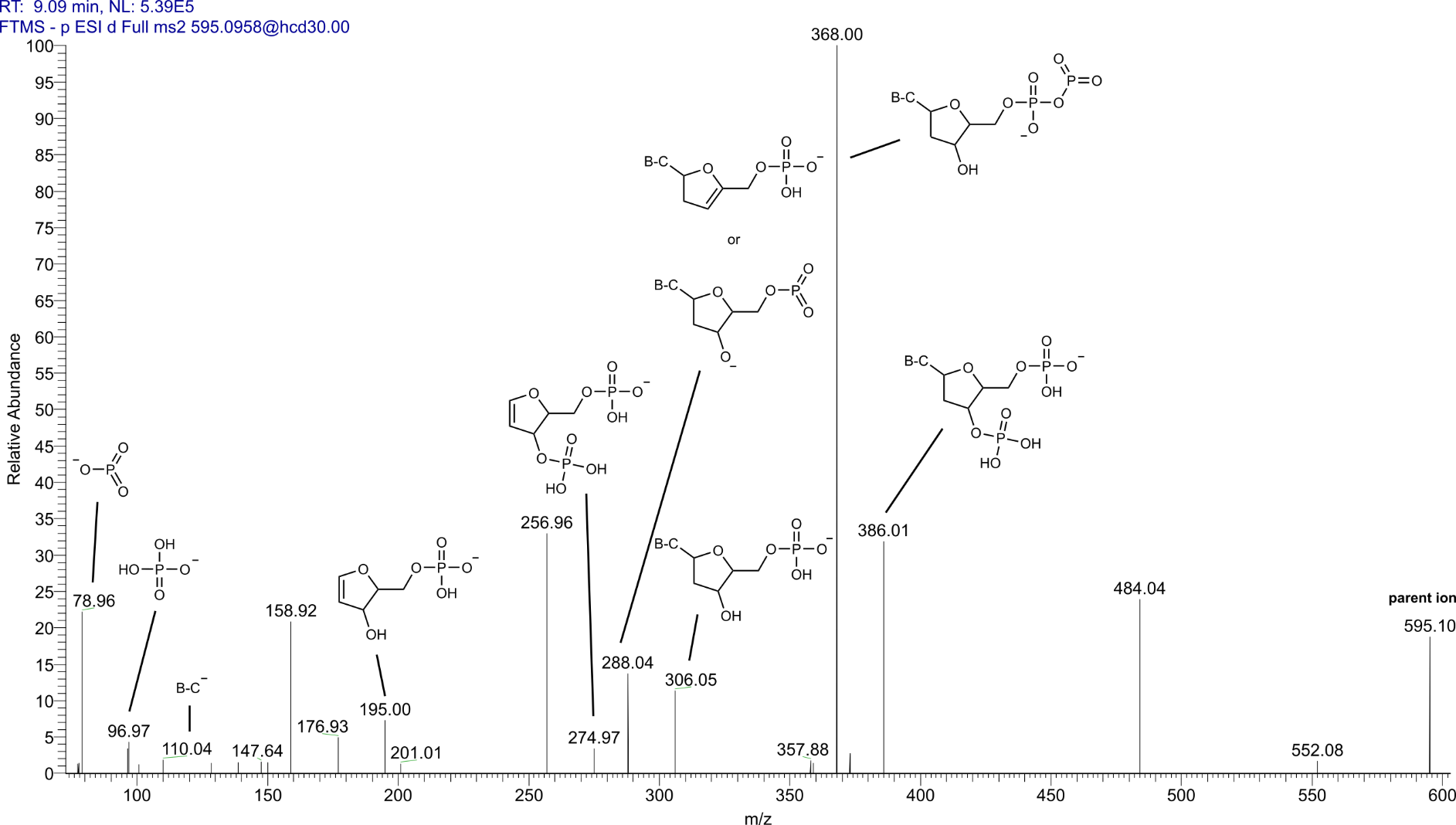


Supplementary Figure 7. MS/MS spectrum of the dC based dinucleotide (m/z 595.0960) obtained from the reaction of dC (100 mM) with H_3_PO_3_ (3.0 eq.) and urea (1.0 eq.) for 7 d. Collision induced dissociation (NCE 30%) of the parent ion at t_mig_ = 9.09 min after CE separation led to the assigned fragments. Displayed fragment structures have model character since there are multiple possible isomers.


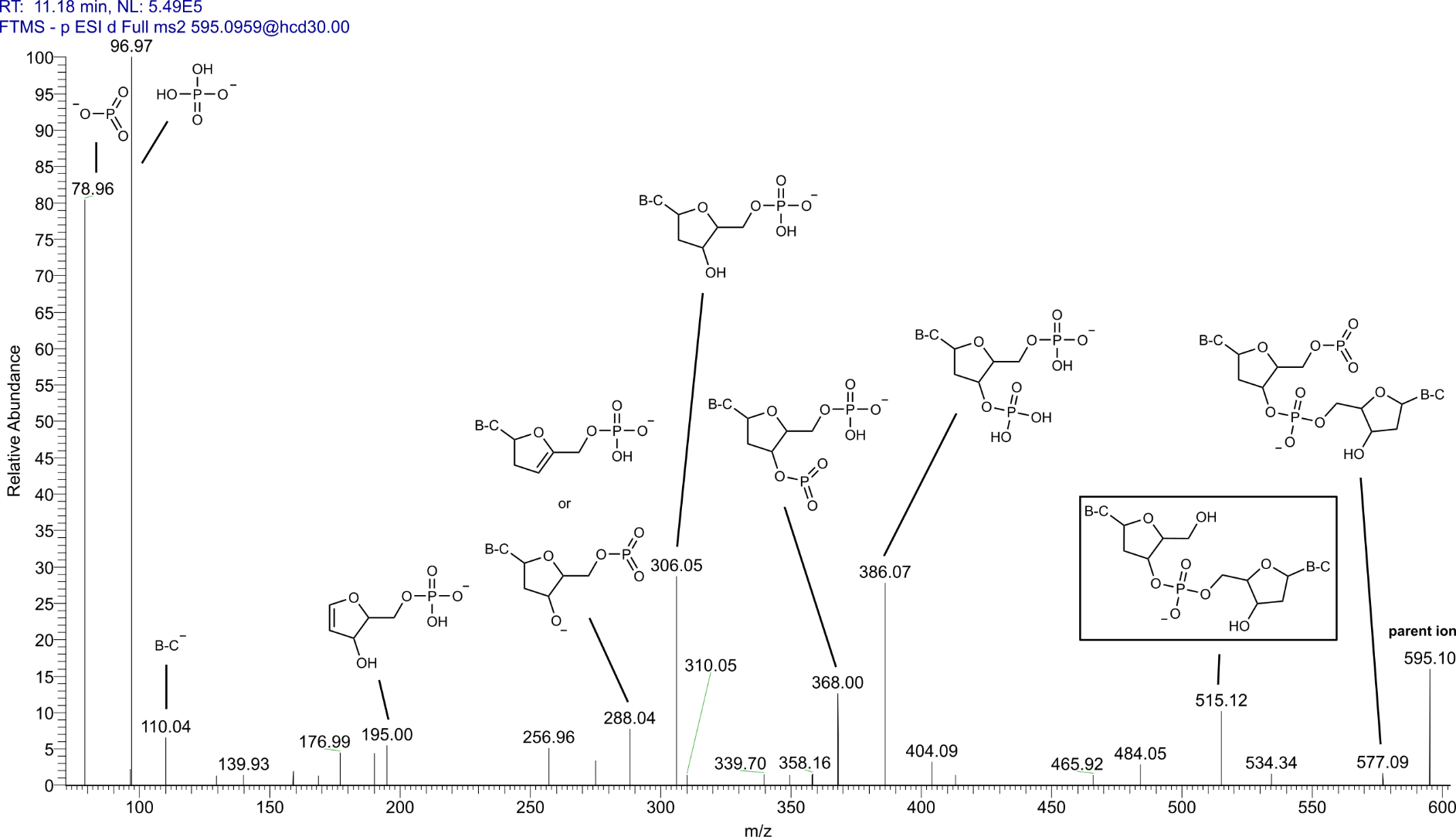


Supplementary Figure 8. MS/MS spectrum of the dC based dinucleotide (m/z 595.0960) obtained from the reaction of dC (100 mM) with H_3_PO_3_ (3.0 eq.) and urea (1.0 eq.) for 7 d. Collision induced dissociation (NCE 30%) of the parent ion at t_mig_ = 11.18 min after CE separation led to the assigned fragments. Fragments in boxes are characteristic for alternating dC and phosphate units. Displayed fragment structures have model character since there are multiple possible isomers.


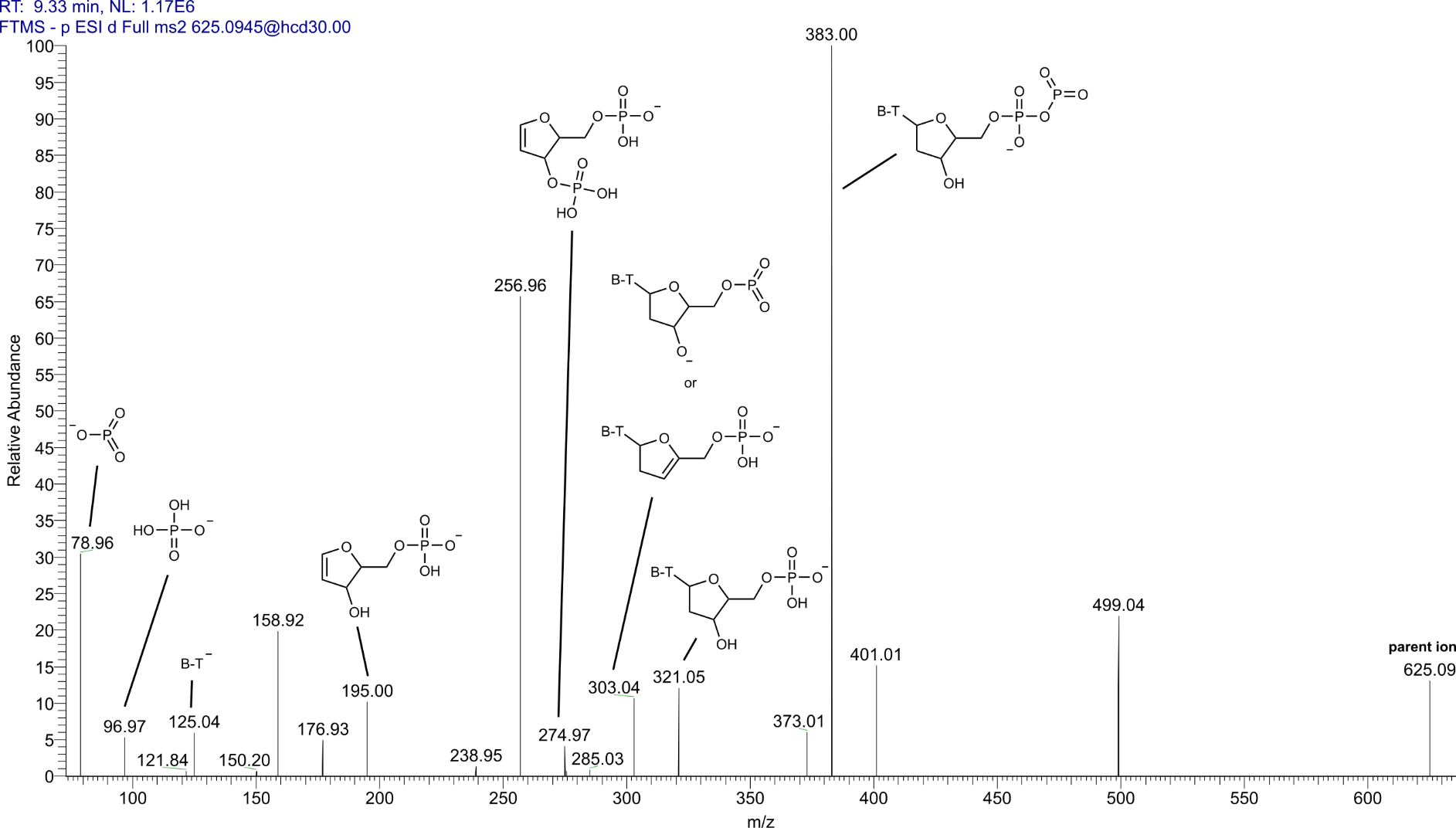


Supplementary Figure 9. MS/MS spectrum of the dT based dinucleotide (m/z 625.0953) obtained from the reaction of dT (100 mM) with H_3_PO_3_ (100 mM) and urea (1.0 eq.) for 7 d. Collision induced dissociation (NCE 30%) of the parent ion at t_mig_ = 9.33 min after CE separation led to the assigned fragments. Displayed fragment structures have model character since there are multiple possible isomers (B-T: thymine).


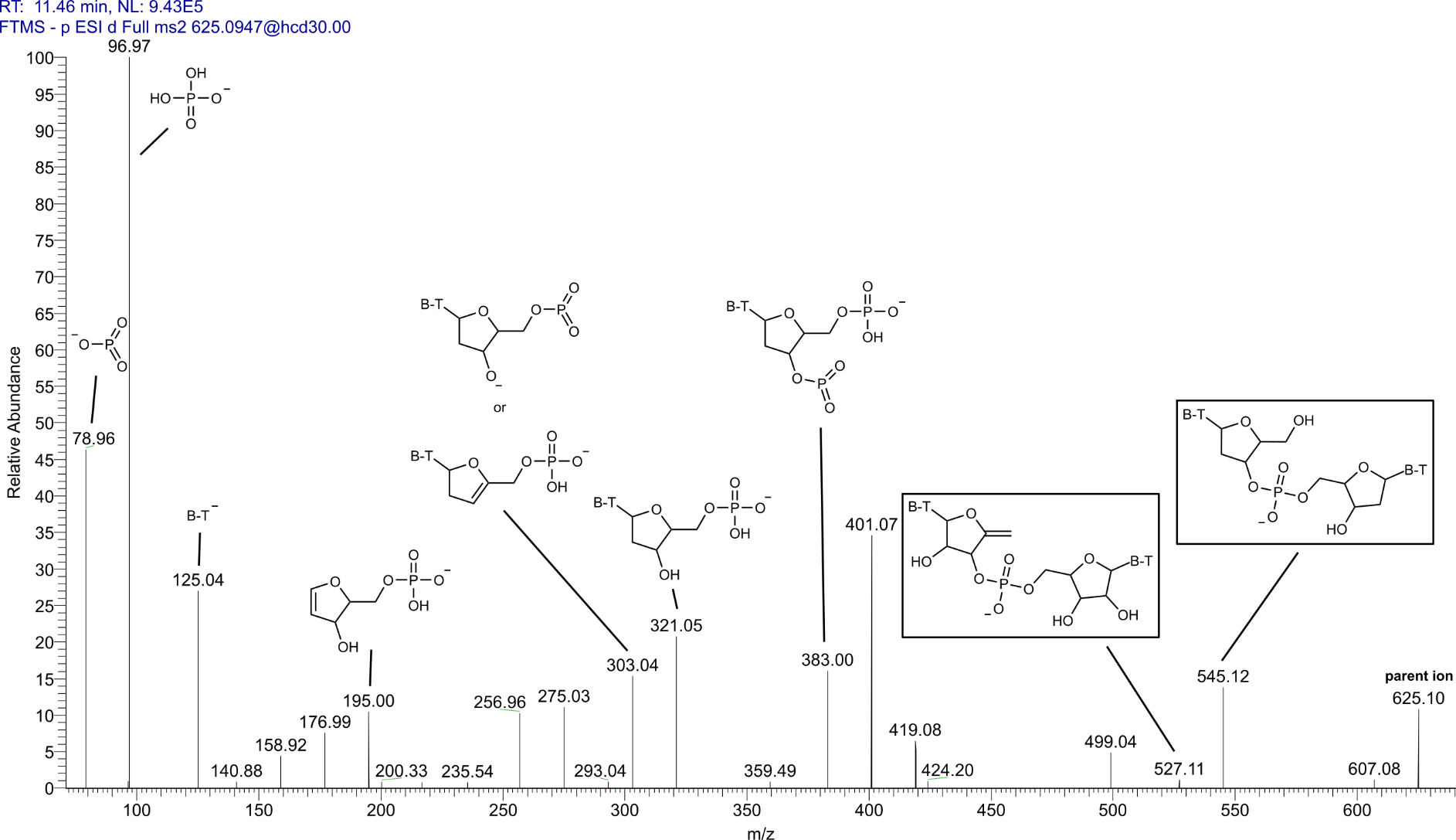


Supplementary Figure 10. MS/MS spectrum of the dT based dinucleotide (m/z 625.0953) obtained from the reaction of dT (100 mM) with H_3_PO_3_ (100 mM) and urea (1.0 eq.) for 7 d. Collision induced dissociation (NCE 30%) of the parent ion at t_mig_ = 11.46 min after CE separation led to the assigned fragments. Fragments in boxes are characteristic for alternating dT and phosphate units. Displayed fragment structures have model character since there are multiple possible isomers.
