# Supplementary material for: Phosphorylation in liquid sulfur dioxide under prebiotically plausible conditions
Source: Commun Chem. 2022 Nov 3;5:143. doi: 10.1038/s42004-022-00761-w (PMC9814524; doi:10.1038/s42004-022-00761-w)
Supplement: Supplementary file 1 — Trapp_PR File [file 42004_2022_761_MOESM1_ESM.pdf]

nature portfolio

Peer Review File

Phosphorylation in liquid sulphur dioxide under prebiotically plausible conditions

Open Access This file is licensed under a Creative Commons Attribution 4.0 International License, which permits use, sharing, adaptation, distribution and reproduction in any medium or format, as long as you give appropriate credit to the original author(s) and the source, provide a link to the Creative Commons license, and indicate if changes were made. In the cases where the authors are anonymous, such as is the case for the reports of anonymous peer reviewers, author attribution should be to 'Anonymous Referee' followed by a clear attribution to the source work. The images or other third party material in this file are included in the article’s Creative Commons license, unless indicated otherwise in a credit line to the material. If material is not included in the article’s Creative Commons license and your intended use is not permitted by statutory regulation or exceeds the permitted use, you will need to obtain permission directly from the copyright holder. To view a copy of this license, visit http://creativecommons.org/licenses/by/4.0/.

This manuscript has been previously reviewed at another Nature Portfolio journal. This document only contains reviewer comments and rebuttal letters for versions considered at Communications Chemistry.

REVIEWERS' COMMENTS:

Reviewer #1 (Remarks to the Author):

I have previously reviewed the manuscript by Professor Oliver Trapp and colleagues. This is the revised version of the same manuscript. I have also read the document specifically addressed to answer the reviewer(s)'s concerns and the list of the changes made by the authors, the addition of new references, and how the concerns of each point raised by the reviewers (including myself) have been addressed. The changes made have also been highlighted and therefore were easy to follow. The major concerns include the plausibility of liquid SO2 on the early Earth (justified by the phase diagram and the new references added), the presence of water traces/humidity and its impacts on the performance of the proposed prebiotic solvent (liquid SO2), the quantification of the inorganic phosphate as a product of the oxidation reactions of the phosphites in the liquid SO2, the rationale of adding urea in the reactions, more information regarding the presence and relevance of phosphites on the early Earth, the redox role of SO2 and formation of the elemental sulfur and phosphorothioate, etc. have been addressed.
Although there is no proof to strongly convince us that liquid SO2 could be considered a prebiotically realistic solvent on the early Earth and while the evidence of the availability of the liquid SO2 and its use as reported in the current manuscript still seem to look unrealistic from a traditional prebiotic chemistry perspective, the manuscript would be a valuable addition to the Communications Chemistry journal as the work could be applicable to various aspects of the synthetic organic chemistry and various sub-fields. Aside from the prebiotic relevancy of liquid SO2 as a (prebiotic) solvent, the authors have done a great job in supporting the claims and observations made in the manuscript with the help of sound analytical tools.
It should be noted that phosphites (or other reduced oxidation state phosphorus compounds) are known to have occurred on the early Earth and very little work has been done on their significance and direct relevance to the origin and accumulation of the biomolecules on the early Earth. Therefore, the presented work will be able to fill this gap.
In addition to the prebiotic chemistry relevancy, the manuscript also offers exciting new results, specifically the simultaneous oxidation and phosphorylation reactions of the reduced oxidation state phosphorus (P) compounds into phosphate and the phosphorylation of nucleosides.
Furthermore, the fact that the nucleosides and other organics are readily phosphorylated at room temperature is novel.
In summary, this paper has been revised satisfactorily, and therefore, I recommend its publication in the journal Communications Chemistry.

List of Changes

Phosphorylation in liquid sulphur dioxide under prebiotically plausible conditions

On behalf of all the authors, I would like to thank the competent reviewer for providing us with great feedback on our manuscript. We greatly appreciate all the helpful suggestions and valuable comments provided by the reviewer to improve the quality of the manuscript.

Reviewer #1 (Remarks to the Author):

I have previously reviewed the manuscript by Professor Oliver Trapp and colleagues. This is the revised version of the same manuscript. I have also read the document specifically addressed to answer the reviewer(s)'s concerns and the list of the changes made by the authors, the addition of new references, and how the concerns of each point raised by the reviewers (including myself) have been addressed. The changes made have also been highlighted and therefore were easy to follow. The major concerns include the plausibility of liquid SO2 on the early Earth (justified by the phase diagram and the new references added), the presence of water traces/humidity and its impacts on the performance of the proposed prebiotic solvent (liquid SO2), the quantification of the inorganic phosphate as a product of the oxidation reactions of the phosphites in the liquid SO2, the rationale of adding urea in the reactions, more information regarding the presence and relevance of phosphites on the early Earth, the redox role of SO2 and formation of the elemental sulfur and phosphorothioate, etc. have been addressed.

Although there is no proof to strongly convince us that liquid SO2 could be considered a prebiotically realistic solvent on the early Earth and while the evidence of the availability of the liquid SO2 and its use as reported in the current manuscript still seem to look unrealistic from a traditional prebiotic chemistry perspective, the manuscript would be a valuable addition to the Communications Chemistry journal as the work could be applicable to various aspects of the synthetic organic chemistry and various sub-fields. Aside from the prebiotic relevancy of liquid SO2 as a (prebiotic) solvent, the authors have done a great job in supporting the claims and observations made in the manuscript with the help of sound analytical tools.

It should be noted that phosphites (or other reduced oxidation state phosphorus compounds) are known to have occurred on the early Earth and very little work has been done on their significance and direct relevance to the origin and accumulation of the biomolecules on the early Earth. Therefore, the presented work will be able to fill this gap.

In addition to the prebiotic chemistry relevancy, the manuscript also offers exciting new results, specifically the simultaneous oxidation and phosphorylation reactions of the reduced oxidation state phosphorus (P) compounds into phosphate and the phosphorylation of nucleosides. Furthermore, the fact that the nucleosides and other organics are readily phosphorylated at room temperature is novel.

In summary, this paper has been revised satisfactorily, and therefore, I recommend its publication in the journal Communications Chemistry.

- We appreciate the reviewer’s comment. We thank the reviewers for the helpful and constructive comments during the revision process.
